# Kinetics and Mechanism of PPh_3_/Ni-Catalyzed, Zn-Mediated, Aryl Chloride Homocoupling: Antagonistic Effects of ZnCl_2_/Cl^–^

**DOI:** 10.1021/jacs.4c12088

**Published:** 2024-10-18

**Authors:** Nicole A. Fohn, Yuan Gao, Stephen Sproules, Gary S. Nichol, Colin M. Brennan, Alan J. Robinson, Guy C. Lloyd-Jones

**Affiliations:** † University of Edinburgh, Joseph Black Building, David Brewster Road, Edinburgh EH9 3FJ, U.K.; ‡ University of Glasgow, Joseph Black Building, University Avenue, Glasgow G12 8QQ, U.K.; § Jealott’s Hill International Research Centre, Berkshire, Bracknell RG42 6EY, U.K.; ∥ Syngenta Crop Protection, Research and Development Centre, Stein 4332, Switzerland

## Abstract

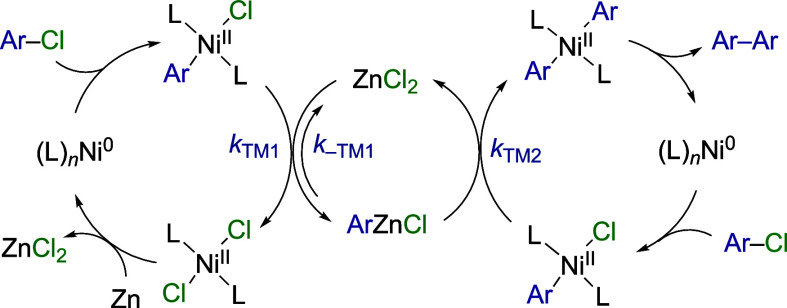

The Ni/PPh_3_-catalyzed homocoupling of aryl chlorides in DMF using Zn as the stochiometric reducing agent is one of a general class of Ni-catalyzed processes, where the mechanism has been a matter of long-standing debate. This study re-evaluates prior conclusions and insights. NMR spectroscopy is used to identify [(PPh_3_)_2_Ni^II^(Ar)Cl] as a key intermediate and to explore the indirect roles of using Zn as the reductant. The [ZnCl_2_] coproduct is responsible for several features, including a sequential transmetalation pathway involving [ArZnCl]. [ZnCl_2_] also abstracts halide from [(PPh_3_)_2_NiCl_2_] to generate [Ni^II^Cl(DMF)_5_]^+^[ZnCl_3_(DMF)]^−^, and in doing so, affects the Ni^II^ + Ni^0^ ↔ 2 Ni^I^ speciation. [ZnCl_2_] thus acts as an accelerator and inhibitor, resulting in mildly sigmoidal reaction profiles. When the [ZnCl_2_] concentration becomes too high or the phosphine ligand concentration too low, catalysis stalls. Turnover is restored by the addition of further phosphine ligand, or chloride ion. In the presence of an exogenous chloride ion, turnover is rapid, again proceeding via [(PPh_3_)_2_Ni^II^(Ar)Cl] but via dinuclear metathesis. The generation of [ZnCl_3_(DMF)]^−^ results in mutually antagonistic effects between [ZnCl_2_] and [Cl]^−^ such that turnover proceeds via one mechanism or the other, depending on which species is in excess. The intermediacy of [ArZnCl] suggests a solution to the long-standing anomaly that many other reductants were found to be much less effective than Zn in inducing turnover of Ni/PPh_3_ catalyzed aryl chloride homocoupling in DMF. The use of DMAc as a solvent in place of DMF inhibits stalling through the steric inhibition of mixed metalate generation.

## 1 Introduction

**Scheme 1:**
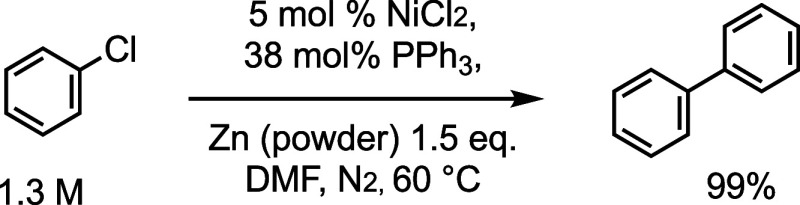
Ni/PPh_3_-Catalyzed ArCl Homocoupling in DMF with Zn as the Stoichiometric Reductant^5,6^

A biphenyl motif is a core component in applications as diverse as atropisomeric ligands, switches, sensors, light-emitting diodes, and liquid crystals. Homocoupling of aryl halides provides a simple route to symmetrical biphenyls,^1^ and in 1971, Semmelhack reported the first nickel-based methodology to achieve this.^2^ The process employed stoichiometric (0.5 equiv) [Ni^0^(COD)_2_] in DMF and efficiently converted aryl bromides into the corresponding biphenyls over a period of 20–30 h at 40–50 °C. These conditions were substantially milder than the classic Cu-mediated Ullmann coupling,^3^ making Semmelhack’s method a major advance at the time.^2^ The stochiometric process was further developed by Kende through the use of Zn powder in the presence of excess PPh_3_ to reduce the more readily handled [(PPh_3_)_2_Ni^II^Cl_2_] in situ to [(PPh_3_)_3_Ni^0^].^4^ In 1977, Kumada showed that the process can be made catalytic in Ni, again using Zn as the stoichiometric reducing agent,^5,6^ and in 1986 Colon reported a widely applicable method for the homocoupling of aryl chlorides using 5 mol % [Ni^II^Cl_2_] that is prereduced with excess Zn and PPh_3_ in DMAc or DMF, Scheme 1.^7^ The PPh_3_/Ni-catalyzed,^5−7^ Zn-mediated homocoupling, Scheme 1, has been applied as a robust route to a wide variety of symmetrical biaryls.^3a,8^

Pioneering developments over the subsequent decades have enabled enantioselective Ni-catalyzed arylhalide homocoupling^9^ and chemoselective (hetero)aryl cross-couplings.^10^ Various combinations of ligands, additives, cocatalysts, and reductants also facilitate a broad range of (enantio)selective cross-electrophile couplings with alkyl and vinyl halides.^11−15^

### 1.1 Previous Mechanistic Proposals for Ni/PPh_3_-Catalyzed Zn-Mediated Aryl Halide Homocoupling

Since its inception, the mechanism^16^ of the nominally simple Zn-mediated Ni-catalyzed homocoupling of aryl halides has been a matter of considerable interest and debate.^2,3,5−7,9,16,17^ Independent mechanistic studies by Kochi et al.,^17a^ Colon et al.,^7^ Amatore and Jutand,^17b^ and Yamamoto et al.,^17c^ and prior observations made on the homocoupling and related processes^6e,6g,17d,17e^ have led to a wide range of mechanistic possibilities.^3a,16^

**Figure 1:**
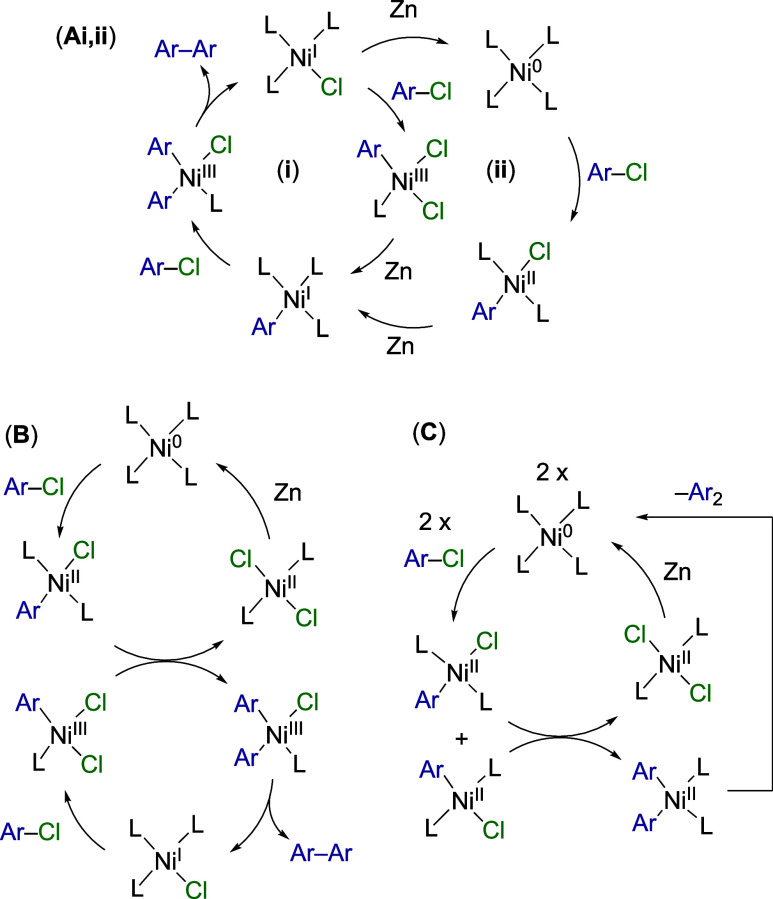
Generic mechanisms (A–C)^3a,5,6e,7a,17c,17d,17e^ previously proposed for the Ni-catalyzed, Zn-mediated, homocoupling of arylhalides, e.g. Scheme 1, when L = PR_3_. Only the key steps are shown.

The most-commonly proposed mechanisms for the process in Scheme 1 are summarized in Figure 1, L = PPh_3_, and involve complexes spanning oxidation states from Ni^0^ to Ni^III^.

In mechanism A, the biaryl is generated by reductive elimination from an (Ar)_2_Ni^III^ intermediate, accessible via two different mononuclear pathways (Ai and Aii). This mechanism is generally proposed to apply when the nickel concentration is low and the reductant is in excess.^3a^ In mechanism B, dinuclear ligand metathesis occurs between Ar–Ni^III^ and Ar–Ni^II^ complexes to access the same (Ar)_2_Ni^III^ intermediate as mechanism A, en route to the biaryl. Mechanism C is less favored,^3a^ and stems from proposals by Yamamoto et al.,^17c^ Rieke et al.,^6e^ Takagi et al.,^6g^ and Otsuka and Yamamoto,^17d^ and from recent work by Baird and Budzelaar et al.^17e^ In mechanism C, only Ni^0^ and Ar–Ni^II^ species play key roles. Dinuclear ligand metathesis, proceeding with second-order kinetics in Ar–Ni^II^, leads to the generation of the biaryl from a transient (Ar)_2_Ni^II^ intermediate.

The mechanisms shown in Figure 1 were proposed from studies of reactions conducted under conditions that diverge, in some cases considerably, from those shown in Scheme 1. For example, Koichi reported a very extensive study of the kinetics of biaryl generation from [(Et_3_P)_2_Ni^II^(Ar)X] complexes in benzene and in toluene, leading to the proposal of dinuclear mechanism B.^17a^ However, the Ni-catalyzed arylhalide homocoupling process requires a polar solvent (e.g., DMF, DMAc, and NMP) and is powerfully inhibited by trialkylphosphines.^5,7^ Kinetic studies by Amatore and Jutand on the Ni-catalyzed dimerization of PhBr involved electrogenerated [Ni^0^(dppe)] in THF/HMPA containing an electrolyte (0.1 M [Bu_4_N]^+^[BF_4_]^−^).^17b^ While this provided very detailed insight into various oxidative and reductive steps, supporting mononuclear mechanism Aii, the preparative Ni-catalyzed process, Scheme 1, is inhibited by the chelating diphosphine ligand dppe.^7a^ Yamamoto studied the reaction of [Ni^0^(COD)_2_] with two equivalents of PPh_3_ and excess PhBr in DMF at 60 °C.^17c^ The kinetics for biphenyl generation were found to be second-order in [Ni]_tot_ (*k*_obs_ = 0.16 M^–1^ s^–1^) and independent of [PhBr]_0_, supporting mechanism C. However, these experiments lacked the large excess of PPh_3_ and the Zn reductant that are present in the catalytic systems.^5−7^ Colon investigated the factors controlling the rate of Ni/PPh_3_-catalyzed Zn-mediated homocoupling of chlorobenzene in DMAc at 70 °C. Sigmoidal reaction profiles were ascribed to autocatalysis, for which control experiments “clearly demonstrated that the autocatalytic behavior was due to the formation of chloride ions; the metal cationic species play no role in the acceleration.”^7a^ Kinetic studies suggested that the process is pseudo-zero-order in aryl chloride, with rate-limiting Zn-mediated reduction of a Ni-aryl species, supporting both mechanisms Ai and Aii. However, the analysis was based on the responses to simple binary variations in reaction stoichiometry in “reactions in which the autocatalytic effect was suppressed by added sodium bromide”.^7a^

Herein, we report on the kinetics and mechanism of the Ni/PPh_3_-catalyzed Zn-mediated homocoupling of aryl chlorides under the classic conditions. The study re-evaluates the conclusions drawn by Kumada^5^ and by Colon^7^ as well as building on and contextualizing the prior mechanistic insights by Rieke et al.^6e^ Takagi et al.^6g^ Otsuka et al.,^17d^ Yamamoto et al.,^17c^ and Baird and Budzelaar et al.^17e^ on dinuclear mechanism C. A key aspect of the study, which primarily employs in situ and ex situ NMR analyses, has been the exploration of the indirect roles of the use of Zn as the reductant. As shown below, [ZnCl_2_] that is stoichiometrically cogenerated during the homocoupling is responsible for many of the counterintuitive features of the process.^18^

## 2 Results and Discussion

### 2.1 Development of the System for Analysis

**Scheme 2:**
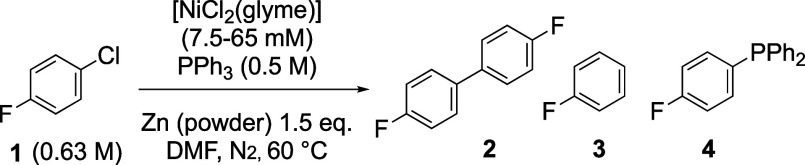
Ni/PPh_3_-Catalyzed Homocoupling of 1

The process selected for study was the homocoupling of 4-fluorochlorobenzene, Ar–Cl **1** (Ar = *p*-C_6_H_4_F) in DMF at 60 °C, to generate the corresponding biaryl **2**, Scheme 2. The reactions employed powdered-Zn as the heterogeneous reductant and were initiated by adding the ArCl (**1**) after pregenerating the homogeneous catalyst in situ from [NiCl_2_(glyme)], Zn, and PPh_3_.^7a,20^ The catalyzed process was initially monitored by periodic removal of small volumes of DMF solution for ex situ quantitative ^19^F NMR spectroscopic analysis.^19^ As noted previously,^7a^ in addition to biaryl **2**, the reactions also progressively generate traces of fluorobenzene (Ar–H, **3**)^7b^ and the mixed triaryl phosphine P(Ar)Ph_2_ (**4**) from ligand scrambling.^21^

A series of control experiments (see Supporting Information Section S4.1) confirmed that (i) the sampling process and frequency did not influence the kinetics of consumption of **1** and generation of **2**; (ii) THF-dilution then filtration of the samples through a small plug of silica-gel provided reproducible stable (quenched) solutions for analysis; (iii) the magnetic stirring speed was sufficient to ensure that mass transfer rates were consistent between experiments; (iv) reproducible kinetics were obtained when Ar–Cl **1** was added after the precatalyst mixture ([NiCl_2_(glyme)], PPh_3_ and Zn) had been activated at 60 °C for a period of >7 min to yield [(PPh_3_)_n_Ni^0^].^20^

### 2.2 Preliminary Kinetics, Inhibition by Zn^II^Cl_2_, and De-inhibition by PPh_3_

**Figure 2:**
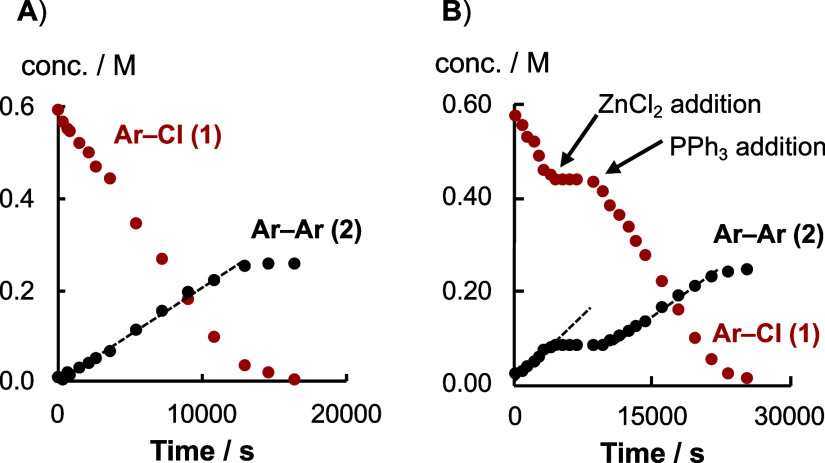
Examples of Ni-catalyzed homocoupling of Ar–Cl (**1**), Scheme 1, monitored by sampling and ex situ ^19^F NMR analysis. NiCl_2_.glyme prereduced for 30 min at 60 °C in DMF before addition of **1**. Conditions: (A) [**1**]_0_ 0.63 M, [Ph_3_P]_0_ 0.50 M, [NiCl_2_(glyme)]_0_ 35 mM, 1.5 equiv. Zn, DMF, 60 °C, N_2_. (B) Same conditions as A, except [Ph_3_P]_0_ = 0.20 M, with exogenous [ZnCl_2_] (0.44 M) added at 3450 s, and further ligand [Ph_3_P] (0.24 M) added at 8790 s.

With a reliable system in hand, the kinetics of a series of reactions conducted across a range of different initial concentrations of **1**, Ni, and PPh_3_ were analyzed, see Supporting Information Section S4.1.4–7. With a phosphine concentration that is sufficiently high to maintain stability, vide infra, the temporal–concentration profiles are very slightly sigmoidal, with approximately linear regimes in d[**2**]/d*t* sustained for the majority of the reaction evolution, as shown in Figure 2A.

The rates of consumption of Ar–Cl (**1**) and generation of biaryl (**2**) were proportional, as shown in eq 1, and were estimated by regression. The rate of product generation, d[**2**]/d*t*, was, to a first approximation, independent of Ar–Cl, [**1**]_*t*_, and [PPh_3_]_0_, with a mixed first and second order dependency on [Ni]_tot_, eq 2.^22^
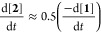
1
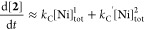
2
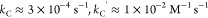

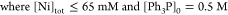


When the initial phosphine concentration, [PPh_3_]_0_, was reduced below 0.5 M, the same rates of turnover were initially observed in the dominant linear regime, but the reactions stalled before completion, see Supporting Information Section S4.1.8. Under these conditions, exogenous ZnCl_2_ (0.7 equiv) introduced during turnover also induced stalling, which was reversed by the addition of further PPh_3_ ligand, Figure 2B. The same deinhibition effect occurred on the addition of NaCl, see Supporting Information Section S4.1.8. The importance of the use of a large excess of triphenylphosphine has been widely noted,^5,7a,8g^ and Kumada proposed that this compensates for the competitive complexation of the PPh_3_ by the accumulating zinc salts.^5^ However, while ZnCl_2_ readily forms [(PPh_3_)_2_ZnCl_2_] complexes in other solvents, for example in ethanol,^23^ we detect no significant complexation of ZnCl_2_ by PPh_3_ in DMF, see the ^31^P NMR titration in the Supporting Information Section S5.2.5. Instead, the PPh_3_-induced attenuation of the ZnCl_2_ inhibition, Figure 2B, arises from the PPh_3_ reducing the extent of mixed-metalate generation; Section 2.10.

### 2.3 In Situ Identification of [(Ph_3_P)_2_Ni^II^(Ar)Cl] (**5**) in the Presence of the Zn Reductant

Ex situ ^19^F NMR spectroscopic analysis of reaction samples taken during the preliminary kinetic survey, Section 2.2 above, identified two additional minor species: [P(Ar)Ph_3_]^+^ and ArOH. Their relative proportions varied erratically between samples, and control experiments showed that both [P(Ar)Ph_3_]^+^ and ArOH are generated from [(PPh_3_)_2_Ni^II^(Ar)Cl] (**5**) during ex situ sampling (separation from Zn and then elution through silica-gel with THF). When the concentrations of the [P(Ar)Ph_3_]^+^ and ArOH are summed to report on the concentration of the Ar–Ni^II^ complex present in the heterogeneous reaction mixture, this confirms a simple relationship between [**5**] and [Ni]_0_, see Supporting Information Section S4.1.4. The maximum concentration of [(PPh_3_)_2_Ni^II^(Ar)Cl] (**5**) is established soon after the initiation of the reaction. It then falls as a function of overall conversion: 2 × Ar–Cl (**1**) + Zn → Ar–Ar (**2**) + ZnCl_2_, becoming very low in the final stages of the process, despite the rate of generation of **2** being approximately constant throughout, Figure 2A. The kinetic origins of this paradox are discussed later.

**Figure 3:**
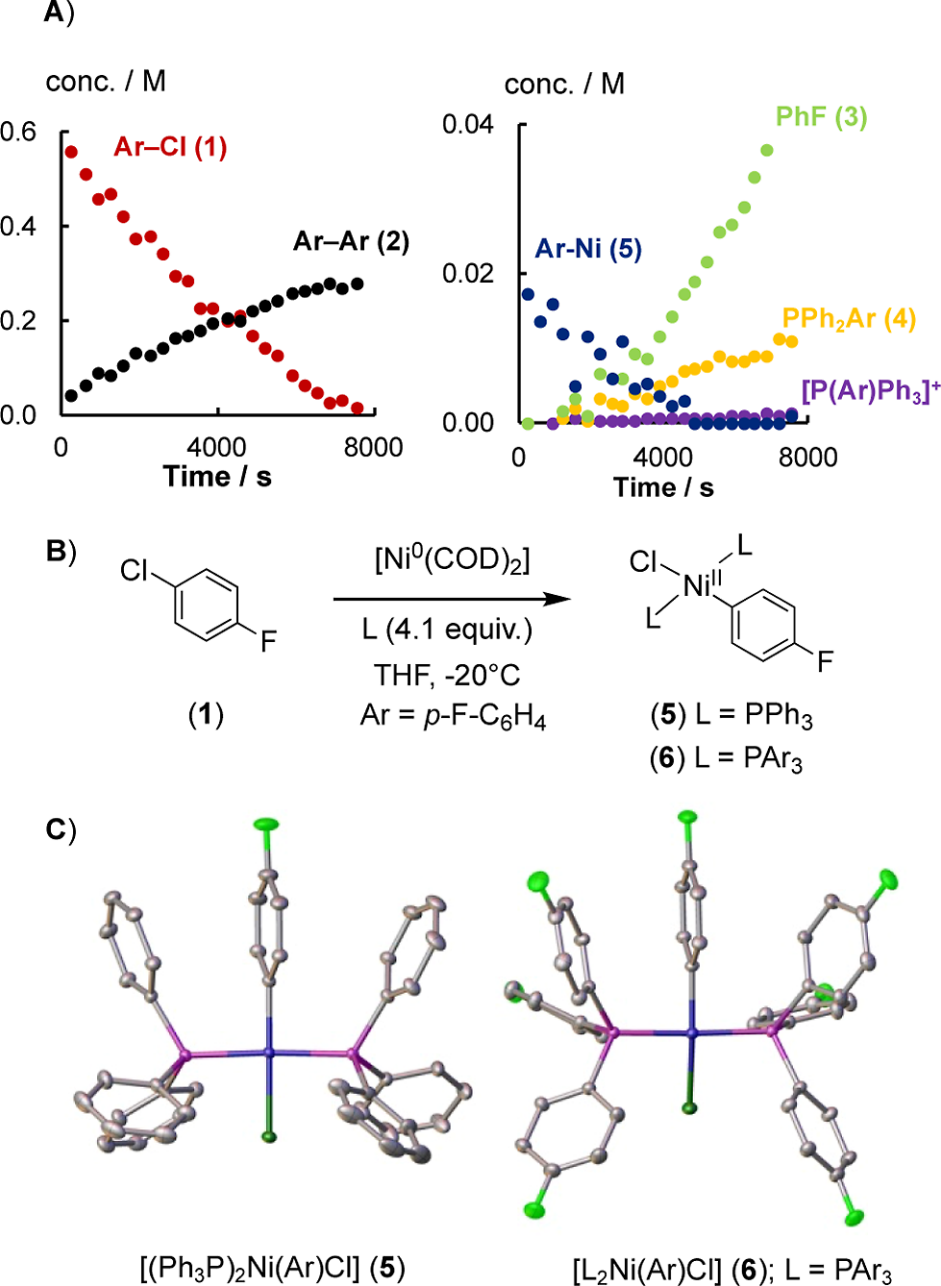
(**A**) Identification of [(PPh_3_)_2_Ni^II^(Ar)Cl] (**5**) during the in situ ^19^F NMR analysis of Ni-catalyzed homocoupling of Ar–Cl (**1**); Ar = *p*–F–C_6_H_4_. Conditions: [**1**]_0_ 0.63 M, [Ph_3_P]_0_ 0.50 M, [NiCl_2_.glyme]_0_ 44 mM, Zn powder 2.1 equiv. (**B**) Synthesis of Ar–Ni complexes **5** and **6**. (**C**) Single crystal X-ray structures of **5** and **6**, THF, omitted for clarity. There is substitutional disorder (0.7 F, 0.3 H) at the aryl-Ni para position in complex **5,** see the Supporting Information Sections S2.2.1 and S12.

By using a system for mechanical mixing of heterogeneous solution/solid-phase reactions within a 5 mm NMR tube located in the NMR spectrometer,^24^ we analyzed the homocoupling of Ar–Cl (**1**) by in situ ^19^F NMR spectroscopy, see Supporting Information Section S4.2. The analysis, Figure 3A, demonstrates that contrary to prior assumptions,^3a^ the Ar–Ni^II^ complex **5** persists in solution during productive turnover and in contact with the Zn reductant.

The analysis also confirms that neither [PPh_3_Ar]^+^ nor ArOH are present at any significant concentration during the reaction, i.e., they are artifacts from the ex situ sampling process. However, the minor coproducts of protodechlorination, Ar–H (**3**), and Ar-ligand scrambling, P(Ar)Ph_2_ (**4**), are evident in the in situ ^19^F NMR spectra and develop throughout the reaction. The Ar–Ni complex **5** was independently prepared by reaction of Ar–Cl **1** with [Ni^0^(COD)_2_] and a large excess of PPh_3_ in THF at −20 °C. Single crystal X-ray diffraction of **5** confirmed the overall structure, but with substitutional disorder at the para position in *p*-X-C_6_H_4_–Ni, Figure 3C, arising from exchange of aryl groups between Ni and the PPh_3_ ligand.^21^

### 2.4 Induction Periods in Ar–Cl (1) Homocoupling Using Ar–Ni^II^ (**5**) and Ni(COD)_2_ to Initiate Catalysis

Initiating catalysis, Section 2.2 above, by addition of the Ar–Cl substrate (**1**) at 60 °C to the deep red solutions obtained by prereduction of NiCl_2_, or the DMF-soluble complex [NiCl_2_(glyme)], results in rapid oxidative addition of **1** to [(PPh_3_)_*n*_Ni^0^]^20^ and the generation of a pale orange-brown solution. In both cases, the growth of the initial complex, [(PPh_3_)_2_Ni^II^(Ar)Cl] (**5**), is indirectly detected by ^19^F NMR spectroscopy and is accompanied by the generation of biaryl **2** through catalysis (*k*_C_) at the rate predicted by eq 2.

**Figure 4:**
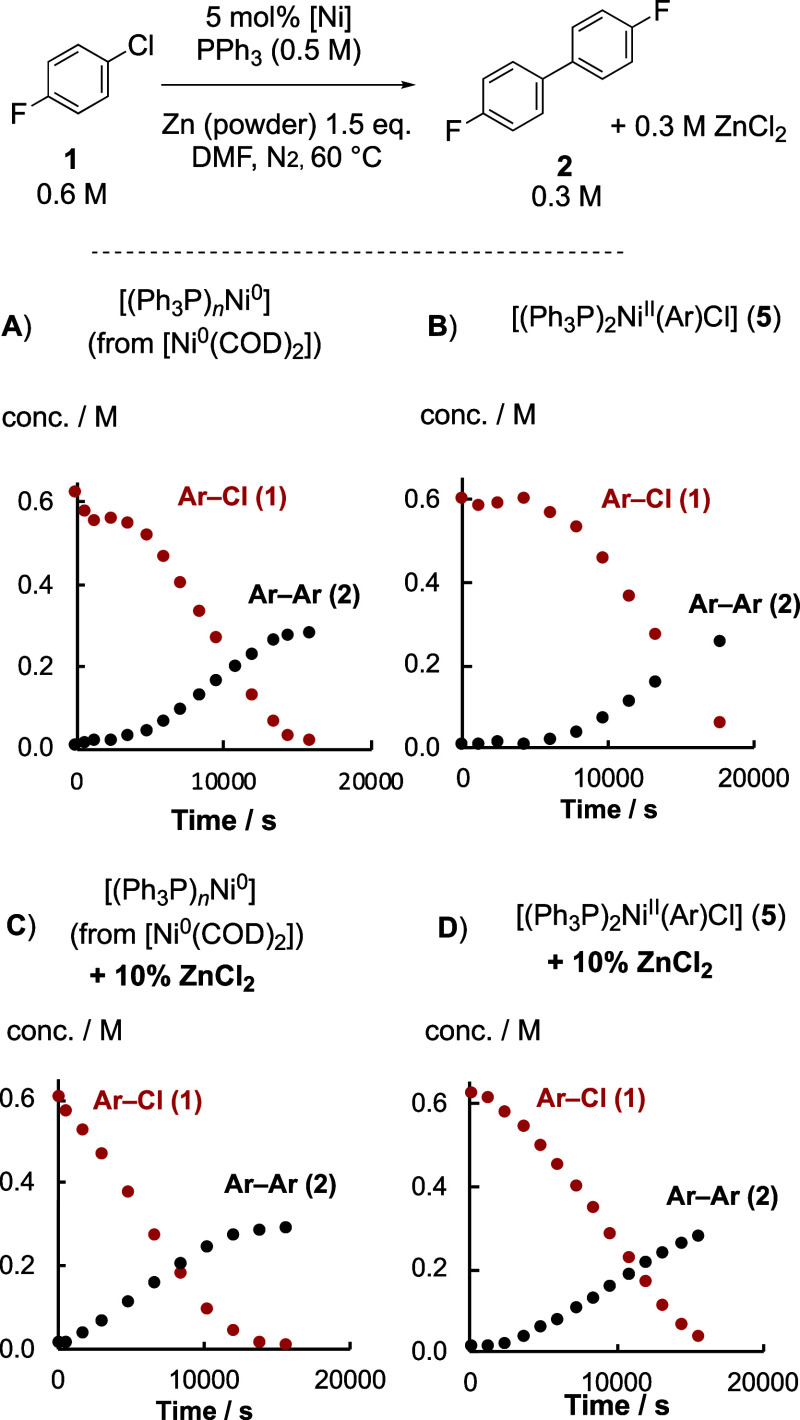
Induction period (A,B) in Ni-catalyzed homocoupling of Ar–Cl (**1**) monitored by ex situ ^19^F NMR analysis and the impact of exogenous [ZnCl_2_] (C,D). Runs (B,D) also include the initial dissolution of complex **5**. See the Supporting Information Section S4.1.11 for details.

When [Ni^0^(COD)_2_] and excess PPh_3_ were used to generate [(PPh_3_)_*n*_Ni^0^] in situ, the same red to orange-brown color changes occurred on addition of **1**, and the generation of [(PPh_3_)_2_Ni^II^(Ar)Cl] (**5**) was detected by ^19^F NMR spectroscopy. However, over a period of more than an hour, the rate of generation of **2** proceeds at least 1 order of magnitude slower than that predicted by eq 2 before accelerating to the usual turnover rate, Figure 4A, see Supporting Information Section S4.1.11. The same behavior is observed when catalysis is initiated using [(PPh_3_)_2_Ni^II^(Ar)Cl] (**5**): there is an approximately 1.5 h period of slow generation of biaryl **2** before about a 10-fold acceleration to reach the normal turnover rate, Figure 4B.

However, when 10 mol % [ZnCl_2_] was added prior to the Ar–Cl (**1**) substrate under otherwise identical conditions, the turnover immediately ensued at the standard rate, i.e., with no induction period evident, Figure 4C. The same effect is observed with the preformed Ar–Ni^II^ complex **5**, Figure 4D. These outcomes reinforce the conclusion that there are profound indirect impacts of the use of Zn as reductant, i.e., through stoichiometric cogeneration of [ZnCl_2_], the origins of which are discussed below.

### 2.5 Slow Direct Stoichiometric Biaryl Generation (*k*_D_) from [L_2_Ni^II^(Ar)Cl] Complex **6**

It has been known for decades that while [(PPh_3_)_2_Ni^II^(Ph)Cl] is stable in the solid state, it decomposes in solution to generate biphenyl and [(PPh_3_)_3_Ni^I^Cl].^ ^17c,17d^−17e^ The analogous Ar–Ni^II^ complex **5** is detected during in situ monitoring of the Ni-catalyzed homocoupling of Ar–Cl (**1**), Figure 3B, in principle providing a mechanism for turnover. However, when the homocoupling is initiated directly by [(PPh_3_)_2_Ni^II^(Ar)Cl] (**5**) Figure 4B, or from [(PPh_3_)_*n*_Ni^0^] generated without in situ reduction by Zn, Figure 4A, the rate of generation of biaryl **2** is initially very slow, resulting in a long induction period.

Repeating the procedure to synthesize the Ar–Ni^II^ complex, Figure 3B, but using P(*p*–F–C_6_H_4_)_3_ in place of PPh_3_ gave [L_2_Ni^II^(Ar)Cl] (**6**). The structure of **6** was also confirmed by single crystal X-ray diffraction, see Supporting Information Section S12, and the *p*–F–C_6_H_4_–Ni moiety was characterized by ^1^H, ^13^C, and ^19^F NMR spectroscopy in DMF containing a large excess of Ar_3_P. The kinetics of stoichiometric generation of biaryl **2** (and Ni^I^ complex **7**, see Section 2.10 below) from the pure isolated Ar–Ni^II^ complex **6** were then analyzed in the presence of excess ligand, L = PAr_3_, without complication from the now degenerate background aryl exchange.^21^

**Scheme 3:**
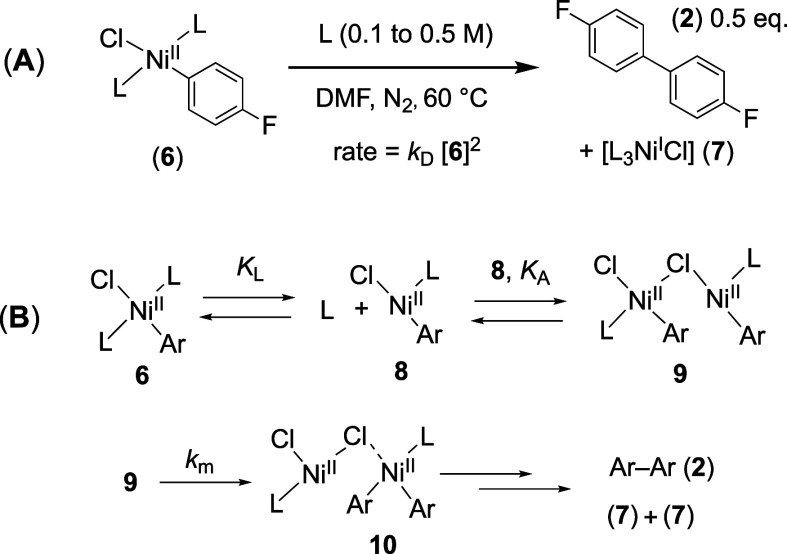
(A) Direct Biaryl (2) Generation (*k*_D_); (B) Neutral Dinuclear Metathesis; L = PAr_3_; Ar = *p*–F–C_6_H_4_

The direct generation (*k*_D_) of biaryl **2**, from **6** (∼10 mM) at 60 °C in DMF, Scheme 3A, was monitored by in situ ^19^F NMR spectroscopy. The reaction proceeds with overall second order^17c,17d^ dependency on Ni^II^-aryl complex **6**, and second-order inhibition by the phosphine ligand, eq 3. There is no evidence for the inhibition or acceleration of the process by the accumulating Ni^I^ coproduct.^25,26^ The simplest explanation for the empirical rate eq 3, is the reversible Ar_3_P dissociation (*K*_L_) from **6** to generate [LNi^II^(Ar)Cl] (**8**), or analogous DMF-solvated species,^17d^ followed by dinuclear association (*K*_A_) to yield bridged complex **9**. Internuclear aryl/Cl ligand metathesis (*k*_m_) to generate **10** then allows various reductive elimination pathways^17e^ to produce biaryl **2**, Scheme 3B. The process is thus inhibited by the phosphine, with an approximately inverse second-order dependency on excess ligand, [PAr_3_]_0_, eq 4, see Supporting Information Section S5.1.4. The generation of dinuclear complex **9** could also proceed by the kinetically indistinguishable sequential reaction of **6** with **8,** followed by ligand dissociation.
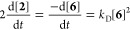
3
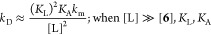
4
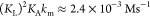
5

This general class of dinuclear Ar/Cl-metathesis mechanism is a key step in Mechanism C (Figure 1), as proposed by Rieke et al.,^6e^ Takagi et al.,^6g^ Otsuka et al.,^17d^ and Yamamoto et al.,^17c,17d^ and recently revisited by Baird and Budzelaar et al.^17e^ The latter study also employed DFT to explore the dinuclear generation of Ph–Ph from [(PPh_3_)_2_Ni^II^(Ph)Cl].^27^ However, the kinetics determined herein show that, at 60 °C in DMF containing 0.5 M PPh_3_, the dinuclear pathway (*k*_D_, eqs 3 and 5, and Figure 1C)^28^ proceeds around 2 orders of magnitude slower than required to significantly contribute to turnover. Thus, other (faster) pathways to generate biaryl **2** must be available under the conditions of (L)_*n*_Ni-catalyzed Zn-mediated homocoupling,^22^Schemes 1 and 2.

### 2.6 Chloride-accelerated (*k*_Cl_) Stoichiometric Biaryl Generation with Antagonism by [ZnCl_2_]

**Scheme 4:**
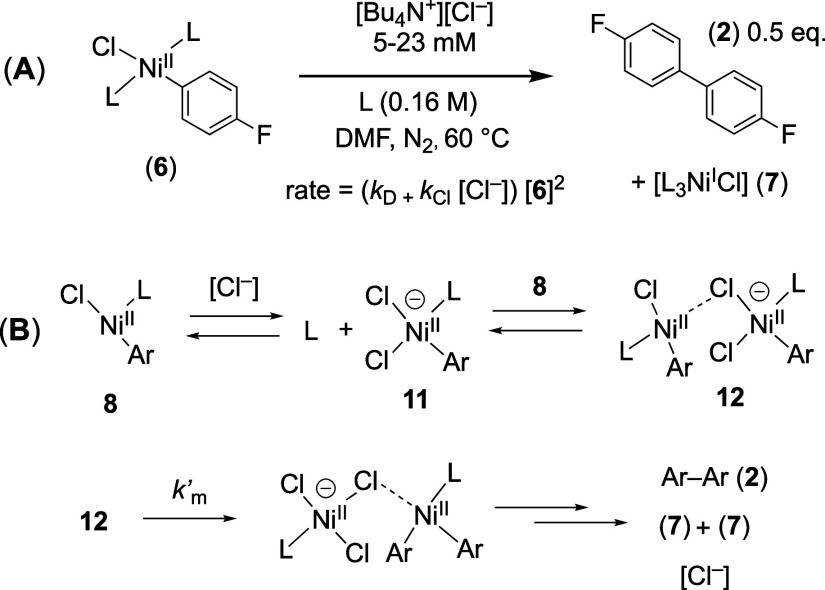
(A) Chloride-acceleration (*k*_Cl_); (B) Anionic Dinuclear Metathesis; L = PAr_3_; Ar = *p*–F–C_6_H_4_

As noted earlier, Colon proposed that Ni/PPh_3_-catalyzed Zn-mediated aryl chloride homocouplings proceed via ZnCl_2_-derived chloride-anion autocatalysis.^7a^ To explore this aspect, we measured the rate of stoichiometric generation of biaryl (**2**) from the Ar–Ni^II^ complex **6**, in the presence of [Bu_4_N^+^][Cl^–^], Scheme 4A. The process is substantially accelerated, with a first-order dependence on chloride ions [Cl^–^]. The kinetic dependency on Ar–Ni^II^ [**6**]_*t*_ remains second-order.

The simplest explanation for this is an analogous dinuclear pathway but via the corresponding nickelate (**11**), Scheme 4B. If there is a lower barrier to internuclear aryl and chloride metathesis, *k'*_m_ in **12**, than for the neutral dinuclear complex **9**, Scheme 3B, then this will lead to first-order chloride acceleration, *k*_Cl_, of the direct reaction, *k*_D_, eq 6. Again, the generation of the dinuclear species (**12**) could proceed by other kinetically indistinguishable sequences.
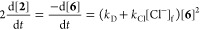
6
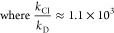


Chloride-ion-induced autoaccelerating biaryl **2** generation is, initially, an appealing explanation for the reaction profiles observed in the Ni-catalyzed aryl chloride homocoupling. However, further kinetic analyses, Section 2.9 below, show that in contrast to [ZnCl_2_] acting as a chloride source, as was proposed by Colon,^7a^ it inhibits chloride-acceleration (*k*_Cl_). This antagonism arises from the strong association of Zn^II^ with the chloride ion in DMF^29^ to generate a zincate, eqs 7 and 8.
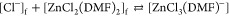
7
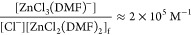
8
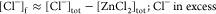
9

The net accelerating effect of chloride on the stoichiometric generation of biaryl (**2**) from the Ar–Ni^II^ complex **6**, eq 6, thus arises solely from ‘free’ chloride ions [Cl^–^]_f_, eq 9. Accordingly, endogenous chloride ion cannot significantly contribute to turnover of aryl-Ni^II^ complexes in the (PR_3_)_*n*_Ni-catalyzed, Zn-mediated, aryl chloride homocoupling, as this effect is suppressed by the accumulating [ZnCl_2_]. Conversely, for the catalyzed process to be accelerated by exogenous chloride,^7a^ the [M^+^][Cl^–^] additive needs to be present at high enough concentrations to ensure that [Cl^–^]_tot_ ≥ [ZnCl_2_] ≈ [**2**], throughout the reaction.

### 2.7 [ZnCl_2_]-Accelerated (*k*_Zn_) Stoichiometric Biaryl Generation with Antagonism by [Cl^–^]

**Scheme 5:**
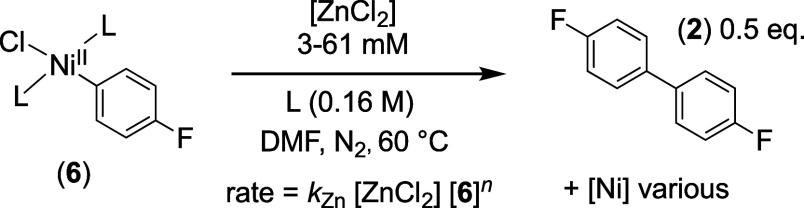
[ZnCl_2_]-Acceleration (*k*_Zn_); L = Ar_3_P; Ar = 4–F–C_6_H_4_

The kinetics above show that neither the slow direct (*k*_D_) nor chloride-accelerated (*k*_Cl_) dinuclear biaryl **2** generation can account for the rate of the Ni-catalyzed aryl chloride homocoupling, eq 2, in the absence of additives. Indeed, for the Ar–Ni^II^ complex (**5**) to be involved, there must be pathway(s) that autoaccelerate to allow escape from the induction period, Figure 4, and sustain the rate of turnover as the concentration of **5** falls, Figure 3A. We therefore explored the effect of [ZnCl_2_] (3 to 61 mM) on the rate of stoichiometric biaryl (**2**) generation from Ar–Ni^II^ complex **6**, Scheme 5.

At [ZnCl_2_] concentrations above 6 mM,^28^ biaryl generation is accelerated over the direct reaction (*k*_D_, Section 6, above). The kinetic dependency on the Ar–Ni^II^ complex (**6**) evolves from being second-order (*n* = 2, eq 10) at low [ZnCl_2_]-concentrations, through fractional order, to being predominantly first-order, *n* = 1, when [ZnCl_2_]_0_ ≥ 30 mM. There is no detectable change in the ^19^F NMR chemical shift of complex **6** (10 mM) at [ZnCl_2_]_0_ concentrations between 3 and 61 mM, suggesting that **6** is the dominant Ar–Ni complex. The temporal concentration data for the intermediate states (1 < *n* < 2) was analyzed using eq 11, in which *a* and *b* are empirical rate coefficients, see Supporting Information Section S8.2.
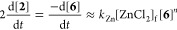
10
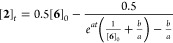
11
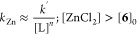
12
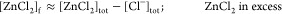
13

The acceleration is first-order dependent on [ZnCl_2_], eq 12, and inhibited by phosphine [L = PAr_3_], with increasing concentrations resulting in a progressive reversion to second-order kinetics in the Ar–Ni^II^ complex [**6**]. Addition of [Bu_4_N^+^][Cl^–^] to the stoichiometric reaction results in zincate, [ZnCl_3_(DMF)]^−^, formation and a reduction in the acceleration to that exerted by the free [ZnCl_2_]_f_*,*eq 13. Thus, the effects of [Cl]^−^ and [ZnCl_2_] on the generation of biaryl (**2**) from Ar–Ni^II^ complex **6** are mutually antagonistic, with the zincate, [ZnCl_3_(DMF)]^−^ inert, i.e., neither accelerating nor inhibiting the process. A holistic analysis of these nontrivial kinetic relationships is reported in Section 2.9 below. However, for now, it is noted that the rate of [ZnCl_2_]-accelerated biaryl (**2**) generation (*k*_Zn_) predicted for the concentrations employed in the Ni-catalyzed homocoupling, Figure 3A, more than accounts^30^ for turnover when [ZnCl_2_]_t_ ≈ [**2**]_*t*_.

**Scheme 6:**
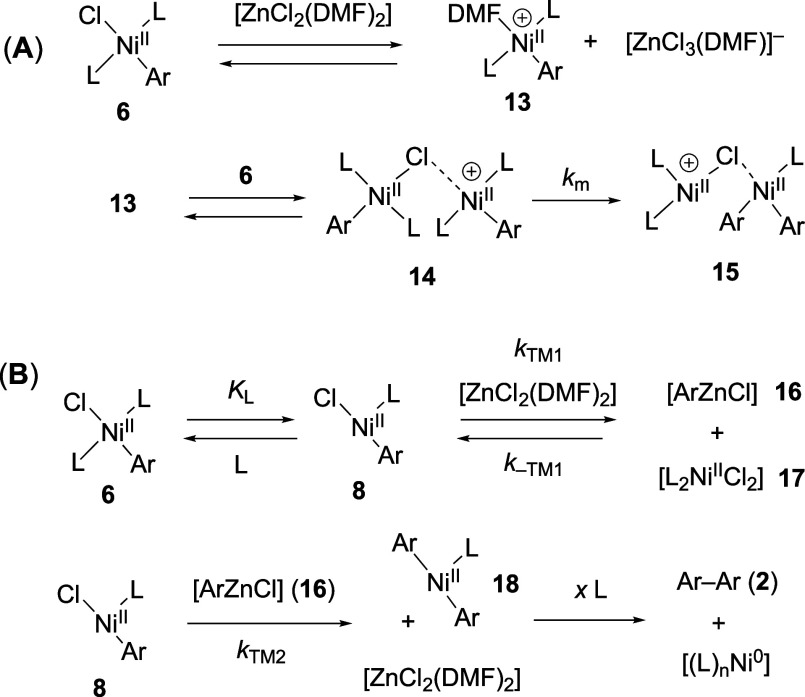
(A) Cationic Metathesis; (B) Transmetalation (*k*_TM_); L = Ar_3_P; Ar = 4–F–C_6_H_4_

Solvent-induced ionization of [L_2_Ni^II^Ar(X)] complexes was proposed by Yamamoto et al. as the origin of the substantial acceleration of the bimolecular rate of biaryl generation by the polar solvent DMF.^17c,17d^ Thus, the first process considered for the [ZnCl_2_]-acceleration of the stoichiometric process, Scheme 5, was direct or indirect chloride transfer^31^ from Ar–Ni^II^ complex **6** to [ZnCl_2_] to enable a cationic dinuclear metathesis (*k*_m_), e.g., via **13**, **14**, and **15**, Scheme 6A. However, this general pathway would be expected to result in (i) inhibition by the zincate, [ZnCl_3_(DMF)^−^], generated by addition of [Bu_4_N^+^][Cl^–^], and (ii) some rate enhancement by [Na^+^][BAr^F–^], neither of which are observed; see Supporting Information Sections S5.1.14 and S5.1.15.

An alternative mechanism to account for [ZnCl_2_]-acceleration involves Zn-transmetalation, Scheme 6B. Analogous pathways have been previously postulated^32^ for Ni/Pd cocatalyzed aryl–aryl cross couplings,^10h,30b^ albeit without explicit evidence, and more recently by Weix to account for loss in selectivity with progressive accumulation of [ZnX_2_] salts in cross-electrophile couplings.^33^ Aryl-transmetalation from Ar–Ni^II^**6**, either directly or after ligand dissociation (**7**), would generate [ArZnCl] (**16**; presumably mono or bis coordinated by DMF) and Ni^II^ complex **17**. The arylzinc intermediate **16** is then primed for aryl transmetalation to the second molecule of Ar–Ni^II^**6**, either directly or after ligand dissociation (**8**), to generate biaryl **2** via rapid reductive elimination from the (Ar)_2_Ni^II^ complex **18**.

The relative rates of the sequential aryl transmetalation steps dictate the overall order in Ar–Ni^II^ (**6**). For example, when the second transmetalation (*k*_TM2_) is more rapid than either the first (*k*_TM1_), or its reverse (*k*_–TM1_), then the overall kinetics will have a first-order dependency on [**6**]_t_ (*n* = 1, eq 10, with *k*_TM1_ rate-limiting). Conversely, when the second (*k*_TM2_) is slower than either the first (*k*_TM1_), or the reverse (*k*_–TM1_), the overall kinetics will have a second-order dependency on [**6**]_t_ (*n* = 2, eq 10, with [*K*_TM1_*k*_TM2_] rate-limiting). Intermediate kinetic regimes will result in 1 < *n* < 2. If both aryl transmetalation steps proceed via the ligand-dissociated (*K*_L_) form of the Ar–Ni^II^ complex, i.e., **8**, then increasing concentrations of [L] will attenuate both transmetalation steps (*k*_TM1_, *k*_TM2_), resulting in a progressive shift from first to second order kinetic dependency on [**6**]_*t*_ and inverse dependency on [L], as is observed, *n* = 2, eq 12.

The general mechanism in Scheme 6B also predicts that exogenous [L_2_Ni^II^Cl_2_] (**17**) will act as an inhibitor by increasing the rate of competing reverse transmetalation (*k*_–TM1_). This feature was tested by the addition of various quantities of [Ni^II^Cl_2_(glyme)] to a system run under conditions that normally elicit the first-order regime in Ar–Ni^II^**6** (*n* = 1). As the concentration of in situ generated [L_2_Ni^II^Cl_2_] (**17**) was raised from 0 to 18 mM, the kinetics progressively shifted from a first to second order dependency on [**6**]_*t*_, *n* = 2, eq 10, see Supporting Information Section S8.4 and Section 2.10 below.

### 2.8 Stoichiometric Reactions of ArZnCl (**16**) with [L_2_Ni^II^(Ar)Cl] (**6**) and [L_2_Ni^II^Cl_2_] (**17**)

**Scheme 7:**
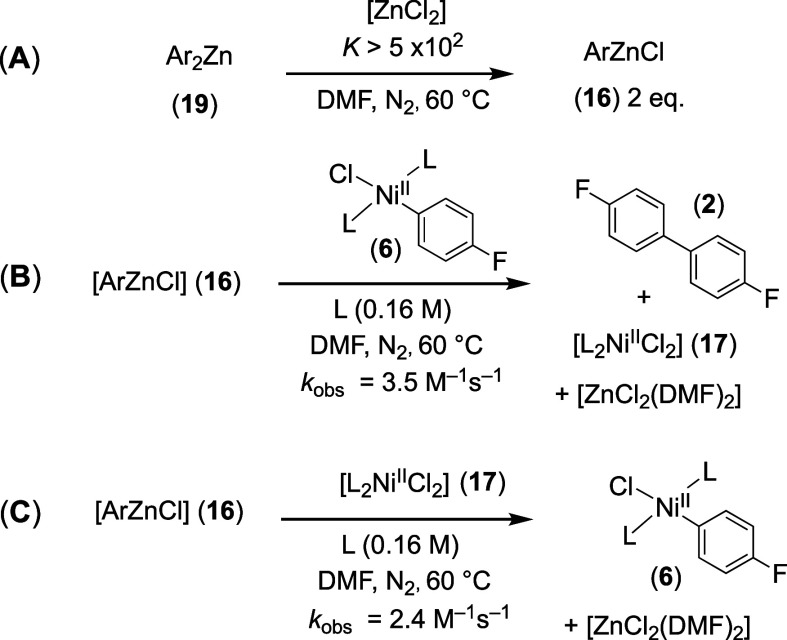
(A) Generation of Arylzinc; (B) Zn-To-Ni Transmetalation (*k*_TM2_); (C) Zn-To-Ni Transmetalation (*k*_–TM1_); L = PAr_3_; Ar = 4–F–C_6_H_4_

To gain additional evidence in support of the above conclusions, the kinetics of stoichiometric reactions of [ArZnCl] (**16**) with the two Ni^II^-complexes (**6** and **17**) were explored. The corresponding diarylzinc [Ar_2_Zn] (**19**) was prepared, sublimed, and then converted to [ArZnCl] (**16**) by addition of [ZnCl_2_] in DMF, Scheme 7A. ^19^F NMR spectroscopic analyses, as seen in Supporting Information Section S5.2.4, show that the rate of reaction of [Ar_2_Zn] with [ZnCl_2_] is close to the NMR time scale at 60 °C, with the equilibrium very strongly biased toward [ArZnCl] (**16**). Both **16** and **19** are stable for a few hours in DMF solution at 60 °C, undergoing slow protodezincation to generate fluorobenzene (**3**). With the key ^19^F chemical shifts identified, a transient low-intensity signal present during in situ ^19^F NMR monitoring of the [ZnCl_2_] (60 mM)-accelerated stoichiometric biaryl (**2**) generation, Scheme 5, was tentatively identified as [ArZnCl] (**16**), see Supporting Information Section S5.1.3.

The kinetics of the relatively rapid stoichiometric reactions of [ArZnCl] (**16**) with [L_2_Ni^II^(Ar)Cl] (**6**), Scheme 7B, and with [L_2_Ni^II^Cl_2_] (**17**), Scheme 7C, were monitored by in situ ^19^F NMR spectroscopy. Numerical methods simulations of the temporal concentrations, see Supporting Information Section S5.1.11, yielded the approximate empirical rates, Scheme 6, indicating that [ArZnCl] (**16**) reacts with [L_2_Ni^II^Cl_2_] (**17**) almost as rapidly as it does with the Ar–Ni^II^ complex **6** (via **8**).^34^

The analogous reactions using [Ar_2_Zn] (**19**) were very rapid (*k*_obs_ > 10^3^ M^–1^ s^–1^). However, the first-order dependency on [ZnCl_2_], eq 10, and the large excess of ZnCl_2_ over arylzinc species (*K* > 5 × 10^2^, Scheme 7A) suggests that [Ar_2_Zn] (**19**) is not a significant contributor to [ZnCl_2_] acceleration, Scheme 5.

### 2.9 Kinetic Model for Stoichiometric Biaryl (**2**) Generation from [L_2_Ni^II^(Ar)Cl] (**6**)

**Figure 5:**
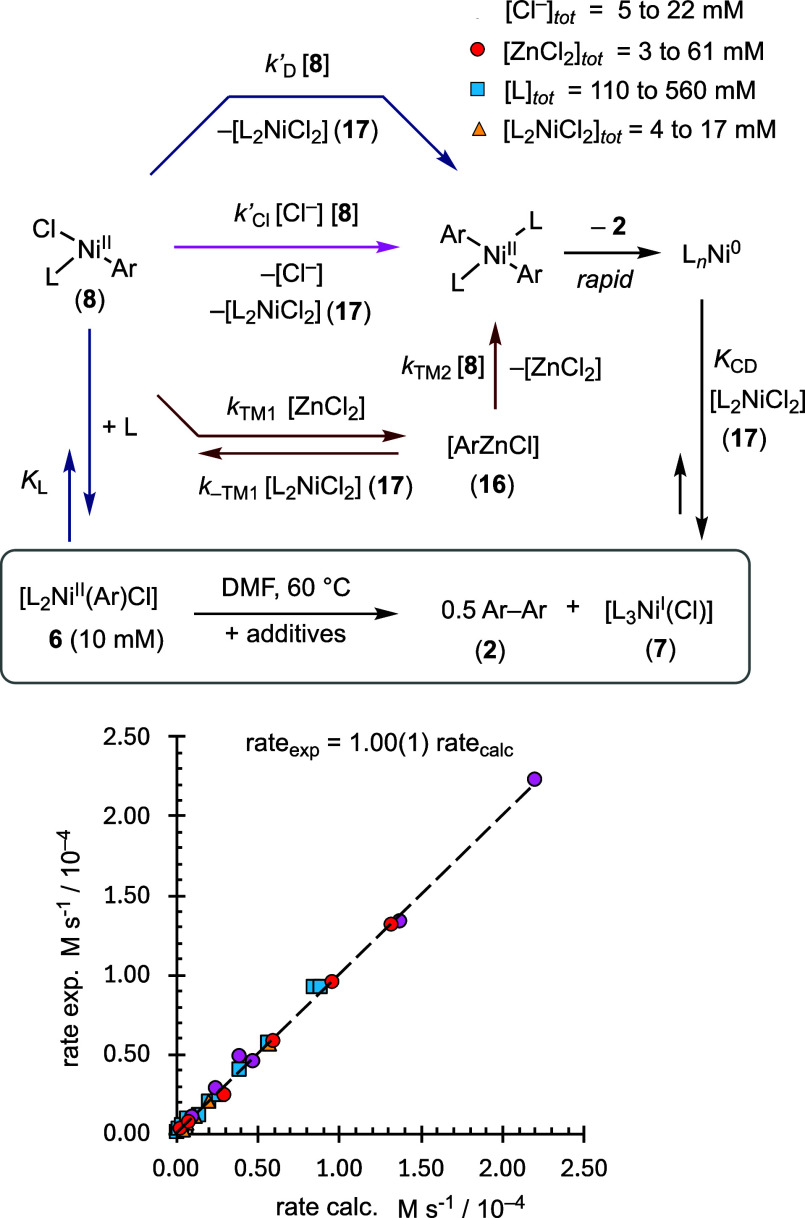
Rates of 29 stoichiometric reactions of [L_2_Ni^II^(Ar)Cl] (**6**) measured by in situ ^19^F NMR spectroscopy, at 60 °C in DMF, with varying concentrations of exogenous additives versus those calculated using eq 14 and the kinetic model shown; see Supporting Information Section S8.4. The experimental rates are normalized to [**6**] = 10 mM using eqs 6 and 11. L = PAr_3_ and Ar = *p*–F–C_6_H_4_.

As discussed in Sections 2.5 to 2.8 above, the direct generation of biaryl **2** from [L_2_Ni^II^(Ar)Cl] (**6**) proceeds readily in DMF in the absence of the Zn reductant, is accelerated by [ZnCl_2_] and by [Cl^–^] (with mutual antagonism), and is inhibited by [L_2_Ni^II^Cl_2_] (**17**) and by PAr_3_. The overall rate of stoichiometric biaryl **2** generation can be approximated by the steady-state rate eq 14, see Supporting Information Section S8.3, which allows trifurcation through direct and accelerated pathways, *k*_D_, *k*_Cl_, and *k*_Z*n*_, Figure 5. The holistic correlation of the observed versus calculated rate, (d[**2**]/d*t*) in eq 14, was obtained by fitting the data from 29 stoichiometric reactions of **6** initiated with different concentrations and combinations of the additives shown.
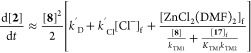
14
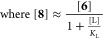

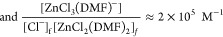


A key observation from the kinetics of stoichiometric biaryl (**2**) generation from [L_2_Ni^II^(Ar)Cl] (**6**) is that endogenous [L_2_Ni^II^Cl_2_] (**17**), common to all three pathways, does not accumulate. If it did, it would compete for [ArZnCl] (**16**), Scheme 7C, and result in deviations from the pseudo first-order kinetics that are observed in stoichiometric biaryl (**2**) generation from Ar–Ni^II^**6** with [ZnCl_2_] concentrations >30 mM. Instead, extensive comproportionation (*K*_CD_) of [L_2_Ni^II^Cl_2_] (**17**) with the nascent equimolar [(L)_*n*_Ni^0^] results in the Ni^I^ complex [L_3_Ni^I^Cl] (**7**) being the major product, as previously reported.^17e,17f,20a^

### 2.10 Impact of [ZnCl_2_] on Ni^0^, Ni^I^, and Ni^II^ Speciation and Semi-catalytic Homocoupling of ArCl (1) by [L_2_Ni^II^(Ar)Cl] (**6**)

**Figure 6:**
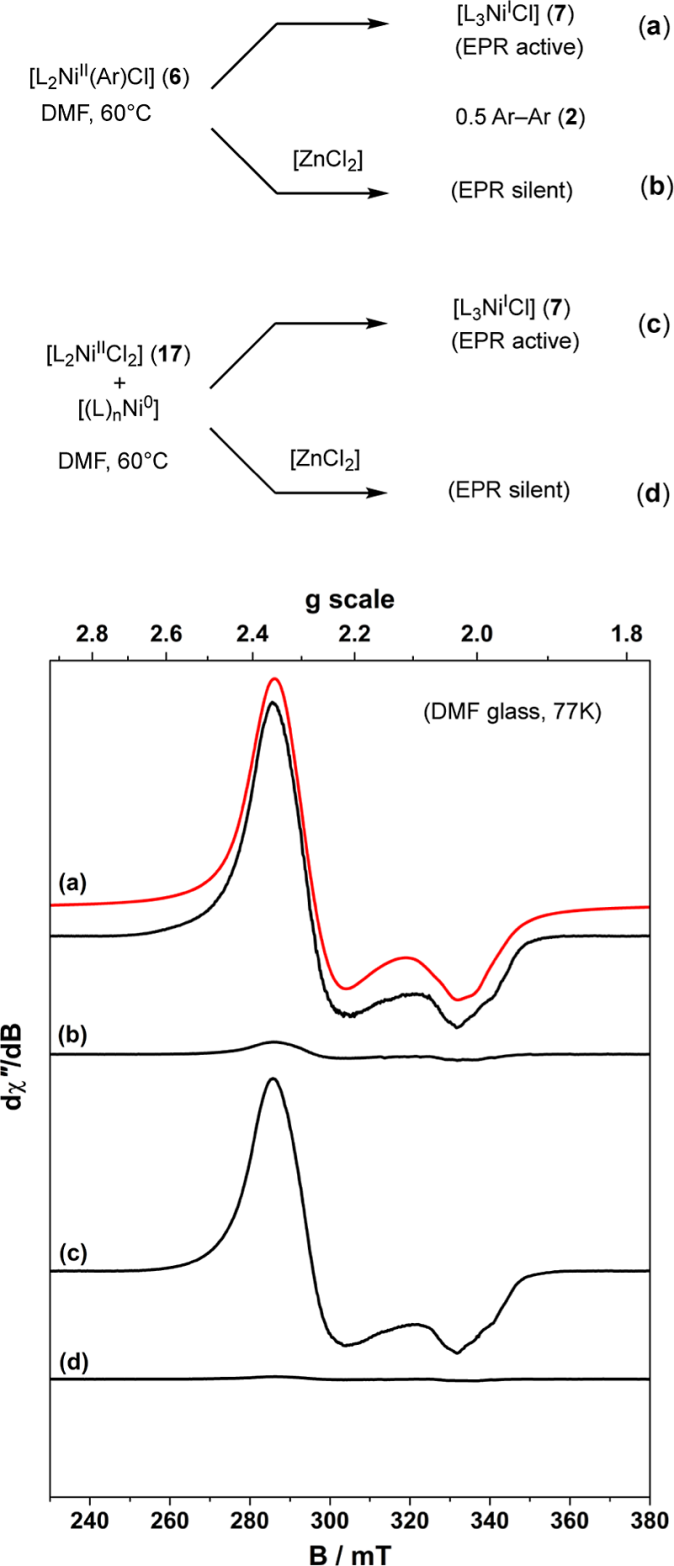
Comparison of the X-band EPR spectra (black lines) for reactions (a–d) conducted in the presence of excess ligand (PAr_3_, 95–165 mM) with or without added [ZnCl2] (47 mM) and then analyzed in DMF glass at 77 K. EPR measurement conditions: frequency, 9.4273 GHz; power, 0.63 mW; and modulation, 0.5 mT. All four spectra are plotted with the same *y*-scale intensity. See Supporting Information Section S7 for discussion and details of the simulation (red line) of the spectrum of a generic complex [(PR_3_)_3_Ni^I^X].^35^

It is clear from the above studies that [ZnCl_2_] is involved in several equilibria and processes in homocoupling catalysis. An initially counterintuitive aspect is that the [ZnCl_2_] induces both acceleration, Figure 4, and inhibition, Figure 2, with the onset of the latter being very sensitive to the phosphine ligand concentration. To analyze this aspect, a series of X-band EPR spectra were acquired of reactions (**a**) to (**d**), Figure 6, conducted at 60 °C, and then rapidly cooled to generate a DMF-glass (77 K, liquid N_2_), see Supporting Information Section S7. Direct biaryl (**2**) generation (*k*_D_) from [L_2_Ni^II^(Ar)Cl] (**6**, 12 mM) analyzed after approximately 50% conversion presents spectral qualities consistent^35^ with the expected^17e,17f,20a^ Ni^I^ complex, [L_3_Ni^I^Cl] (**7**), Figure 6a. Conducting the same reaction in the presence of an excess of [ZnCl_2_] (47 mM) results in substantial attenuation of the intensity of the Ni^I^ signal, Figure 6b.

Analogously, the coreaction of [L_2_Ni^II^Cl_2_] (**17**) with [(L)_n_Ni^0^], both 6 mM, generated in situ from [Ni^II^Cl_2_(glyme)] [Ni^0^(COD)_2_] and PAr_3_ (165 mM), yields intense EPR spectra, Figure 6c, consistent with extensive comproportionation (*K*_CD_) to generate [L_3_Ni^I^Cl] (**7**). The addition of excess [ZnCl_2_] (47 mM) again induces substantial signal attenuation, Figure 6d. The results suggest that competitive equilibria involving [ZnCl_2_]^18^ are coupled to the comproportionation equilibrium (*K*_CD_), resulting in changes to the Ni^II^, Ni^0^, and Ni^I^ speciation as the [ZnCl_2_] concentration is raised.

**Scheme 8:**
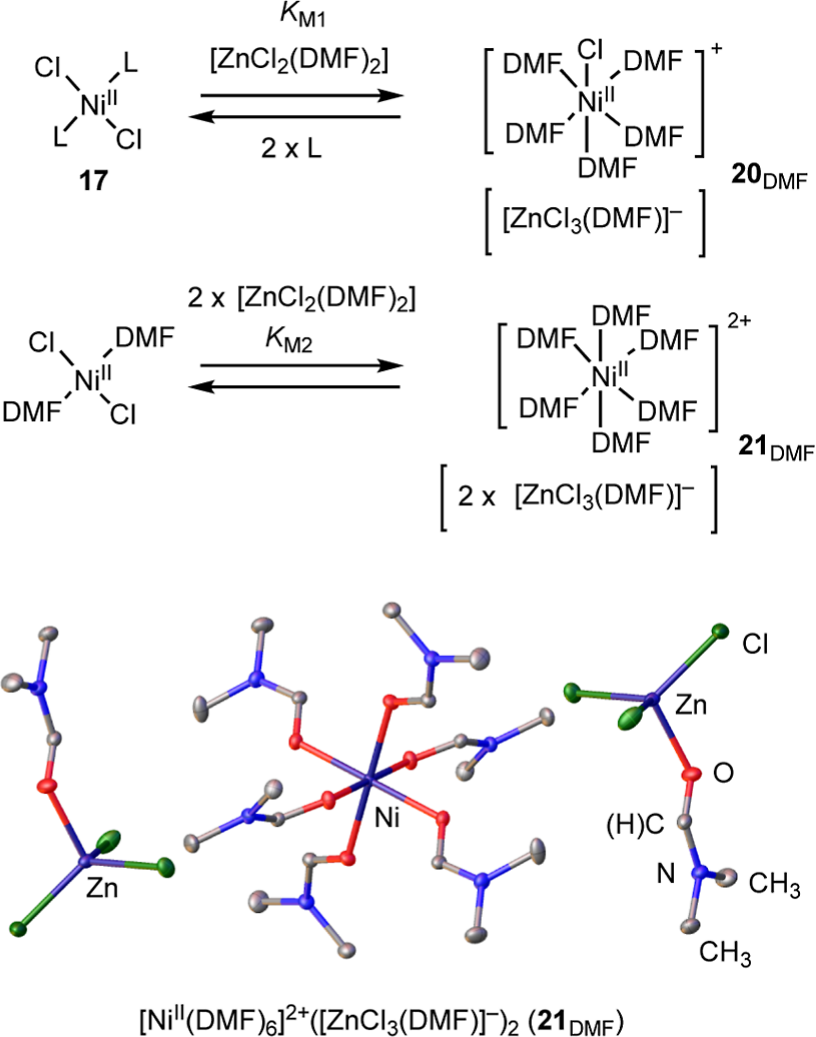
Phosphine-free Mixed Metallate Generation (*K*_M1_ ∼5 M) and Structure of 21_DMF_. L = PAr_3_ and Ar = 4–F–C_6_H_4_

To explore this, the titration of [L_2_Ni^II^Cl_2_] (**17**) with [ZnCl_2_] in the presence of varying concentrations of [L = PAr_3_] was monitored by ^19^F/^31^P NMR spectroscopy, see Supporting Information Section S5.2.1. The data indicates an overall process that results in loss of both phosphines to generate what is tentatively assigned as the mixed metalate^36^ (**20**_DMF_), *K*_M1_ ∼5 M,^37^Scheme 8. Dual chloride transfer (*K*_M2_) occurs when mixing concentrated solutions of [NiCl_2_(glyme)] and [ZnCl_2_] in DMF, yielding the double ion pair **21**_DMF_, characterized by X-ray crystallography, see Supporting Information Section S12, and UV–vis spectroscopy, see Supporting Information Section S6.1.

**Figure 7:**
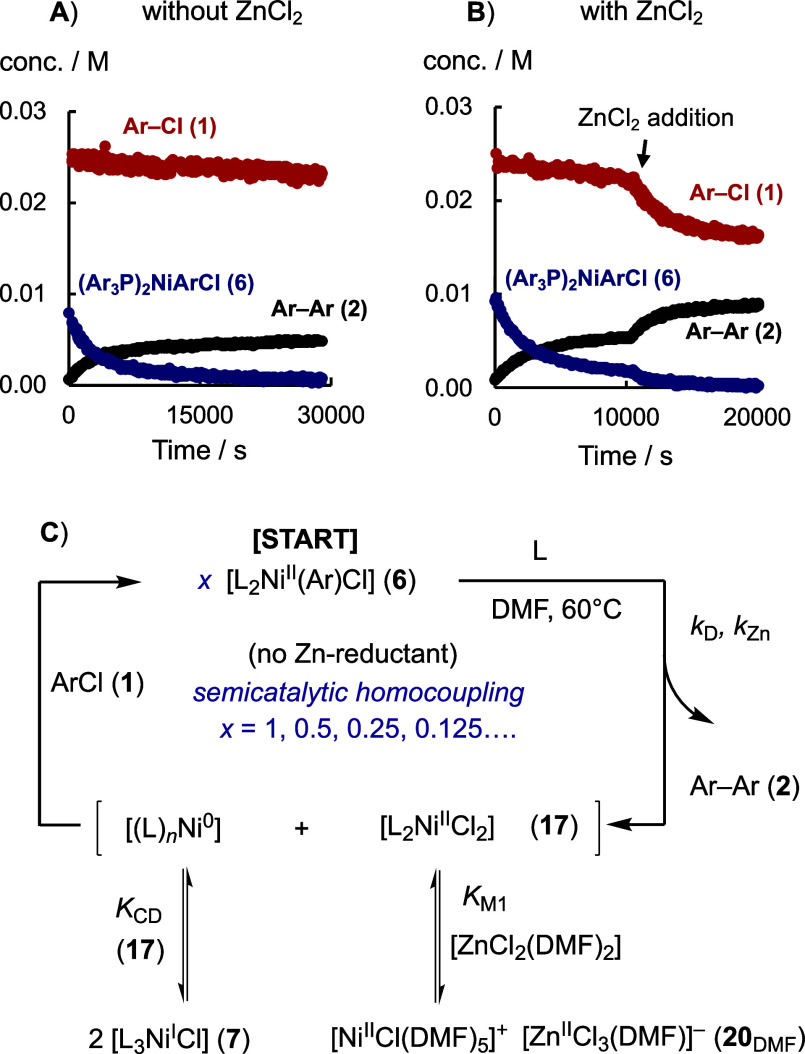
Direct (*k*_D_) and [ZnCl_2_]-accelerated (*k*_Z*n*_) biaryl (**2**) generation from [L_2_Ni^II^ArCl] (**6**, 8 mM) and Ar–Cl (**1**, 25 mM), monitored by in situ ^19^F NMR spectroscopy, see Supporting Information Section S5.1.6. (A) Standard conditions cogenerating Ni^I^ (**7**) via comproportionation (*K*_CD_). (B) With [ZnCl_2_] (30 mM) added at the time indicated to accelerate the semicatalytic^38^ homocoupling of Ar–Cl (**1**). (C) Indirect coupling of equilibria *K*_M1_ and *K*_CD_ increases the [(L)_*n*_Ni^0^] concentration for the oxidative addition of Ar–Cl (**1**).

The impact of [ZnCl_2_] on the Ni-comproportionation-disproportionation equilibrium (*K*_CD_) is further supported by the semicatalytic^38^ homocoupling of Ar–Cl (**1**, 25 mM) using [L_2_Ni^II^(Ar)Cl] (**6**) without the Zn reductant, Figure 7. In the absence of [ZnCl_2_], the slow direct generation of biaryl (**2**) over a period of about 8 h also converts a small proportion of Ar–Cl (**1**) into **2**, about 20% based on **6**, Figure 7A. In other words, there is slow follow-on turnover by oxidative addition of **1** to the low concentration of Ni^0^ present through the equilibrium *K*_CD_. Conducting the same reaction with addition of [ZnCl_2_] (30 mM) after 3 h results in substantially more consumption of Ar–Cl (**1**), ∼100% based on **6**, after addition of the [ZnCl_2_], Figure 7B. This corresponds to full repeated recycling^38^ of Ni^0^ at a concentration that is indirectly raised by depletion of [L_2_Ni^II^Cl_2_] (**17**) through the coupled equilibrium *K*_M1_, Figure 7C. This process is thus distinct from the direct stabilizing role of [ZnX_2_] in facilitating oxidative addition of Ar-X to Co^I^ complexes, for example.^39^

### 2.11 Origins of the Reduced [ZnCl_2_] Inhibition in DMAc versus DMF

Conducting the catalytic homocoupling reaction in a less polar solvent medium results in reduced [ZnCl_2_]-solubility. Adding just 20% (v/v) toluene to the coupling reaction in DMF induces a 10-fold deceleration in the rate of turnover; there was no significant turnover in toluene alone. This is consistent with the known thermal stability of [(PPh_3_)_2_Ni^II^(Ph)X] complexes in, e.g., benzene and toluene.^ ^17c,17d,17e^−17f^

Conversely, catalytic homocoupling reactions conducted in DMAc^7a^ at 60 °C proceeded at the same rates as the analogous processes in DMF but did not undergo [ZnCl_2_]-induced stalling, Figure 2B. This is not a result of the lower solubility of [ZnCl_2_] in DMAc, as the effects are apparent at concentrations substantially below the saturation limits ([ZnCl_2_]_sat_. = 1.4 M in DMAc and 2.3 M in DMF, at 60 °C; see Supporting Information Section S6.2). Moreover, the addition of [ZnCl_2_] during turnover to homocoupling catalysis in DMAc results in modest acceleration under conditions where the addition would cause stalling in DMF. ^19^F/^31^P NMR spectroscopic analysis of the titration of [L_2_Ni^II^Cl_2_] (**17**) with [ZnCl_2_] in the presence of varying concentrations of [PAr_3_], see Supporting Information Section S5.2.2, indicates a much lower propensity for metalate generation (*K*_M1_) in DMAc. This effect, which will strongly attenuate the inhibition by accumulating [ZnCl_2_], Figure 2, is presumably due to the considerable increase in steric crowding in the hexacoordinate Ni^II–^cation [Ni^II^Cl(DMAc)_5_^+^] [ZnCl_3_(DMAc)^−^] (**20**_DMAc_) compared to that in **20**_DMF_, Scheme 8.

### 2.12 Impact of Other Halide Additives

Catalytic homocoupling reactions, Section 2.2 above, conducted with LiCl, NaBr, or NaI as the additive are substantially accelerated,^7a^ see Supporting Information Section S4.1.20. Iodide is particularly effective and, in contrast to chloride, can be used at substoichiometric concentrations, with just 10 mol % inducing substantial acceleration. However, the addition of NaI has no significant effect on the rate of direct (*k*_D_) stoichiometric biaryl (**2**) generation from [L_2_Ni^II^(Ar)Cl] (**6**), nor on the [ZnCl_2_]-accelerated process (*k*_Zn_), see Supporting Information Section S5.1.12. The absence of anionic acceleration by iodide is in stark contrast to free chloride ions, where *k*_Cl_/*k*_D_ ≈10^3^. The lack of mutual antagonism, again in stark contrast to chloride, reflects the approximately 4 orders of magnitude lower affinity of Zn^II^ for iodide anion compared to chloride anion in DMF.^29^ Thus, under the catalytic homocoupling conditions, the accelerating effect of accumulating [ZnCl_2_] can proceed in parallel with an accelerating effect of iodide on other pathways.^40^

## 3 Conclusions

The mechanism of the Ni/PPh_3_-catalyzed Zn-mediated homocoupling of aryl chlorides has long been a matter of debate,^2,3,5−7,9,16,17^ with most studies proposing turnover via mononuclear Ar–Ni^I^ and Ar–Ni^III^ complexes, mechanisms A and B, Figure 1. We have employed a combination of in situ and ex situ ^19^F and ^31^P NMR spectroscopy to analyze the kinetics and equilibria involved in the catalytic process, Scheme 2, augmented by kinetic studies of the stoichiometric reactions of isolated Ar–Ni^II^ intermediates (**5** and **6**, Figures 5 and 7). The rates of the latter are unaffected by the presence of the zinc-reductant. The study has revealed that an underlying set of interdependent equilibria leads to turnover with deceptively simple phenomenological kinetics, Figure 2.

### 3.1 Key Steps (1-12) in the Overarching Mechanism

**Figure 8:**
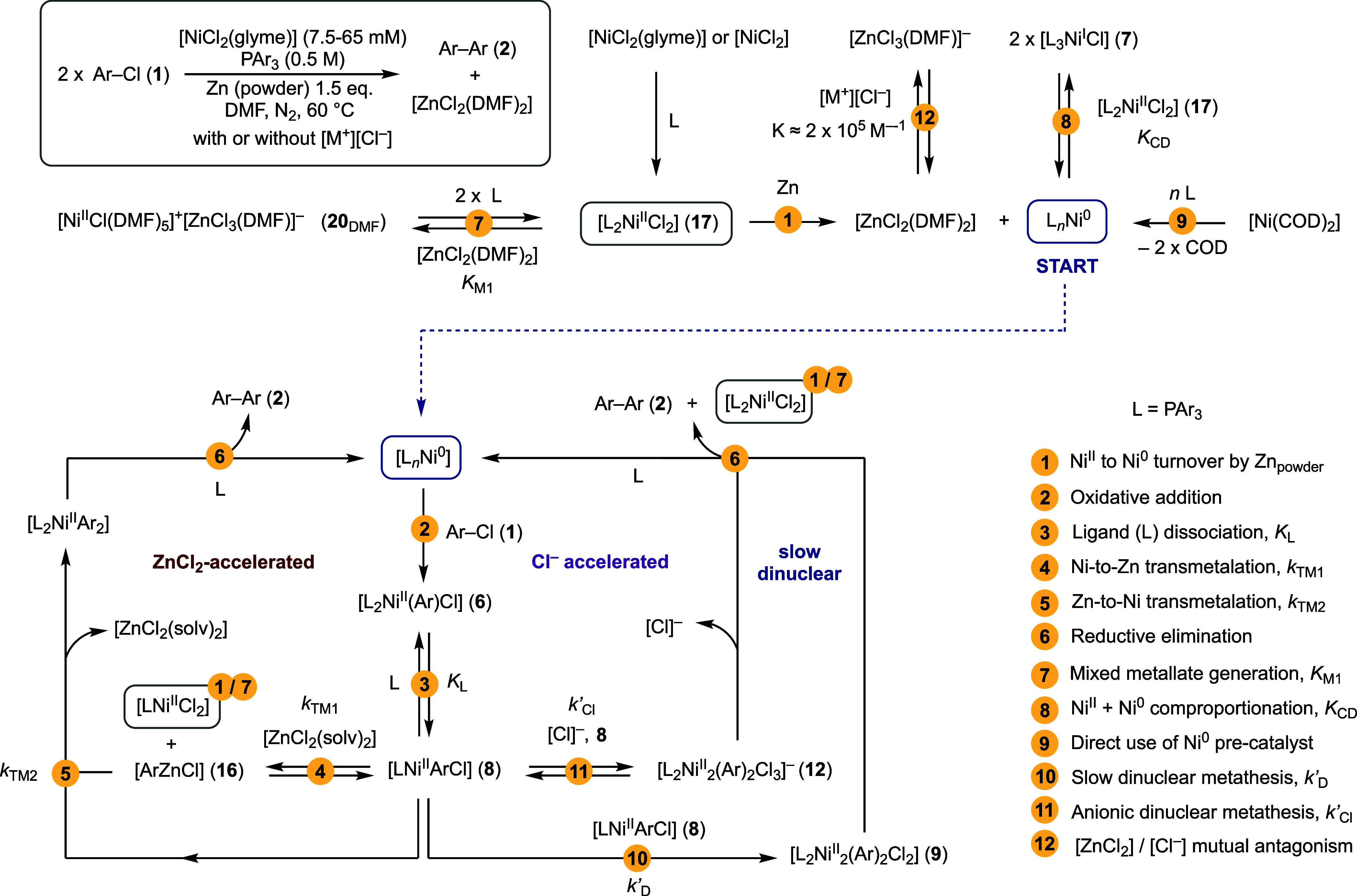
Minimal reaction network, converging at [(L)_*n*_Ni^0^], and trifurcating at Ar–Ni^II^**8**, proposed to account for biaryl generation (**2**) in the [(L)_*n*_Ni]-catalyzed homocoupling of Ar–Cl (**1**) using Zn as the reductant in DMF, with or without exogenous chloride ([M^+^][Cl^–^]). L: = PAr_3_. Some steps are “telescoped” for simplicity. The ZnCl_2_ and Cl^–^ accelerated pathways are mutually exclusive. Key processes (steps 1–12) are highlighted, see text for discussion.

Based on the collected experimental observations, an overarching mechanism can be proposed, Figure 8. Under the archetypal conditions, Scheme 1, in the absence of exogenous chloride anion, the precatalyst, [NiCl_2_(glyme)] or [NiCl_2_], is phosphine ligated and reduced in situ to generate [(L)_*n*_Ni^0^]^20^ and [ZnCl_2_], step 4. Oxidative addition of Ar–Cl (**1**), step 2, generates the Ar–Ni^II^ intermediate (**6**), which is in reversible endergonic equilibrium with traces of ligand-dissociated complex **8**, *K*_L_, step 3. Ar–Ni^II^ intermediate (**8**) undergoes [ZnCl_2_]-accelerated turnover via Ni-to-Zn Ar-transmetalation (*k*_TM1_, step 4) and then Zn-to-Ni transmetalation (*k*_TM2_, step 5) with a second Ar–Ni^II^ intermediate (**8**). Rapid reductive elimination, step 6, from an unobserved diarylnickel produces biaryl (**2**) and regenerates [(L)_*n*_Ni^0^].

The intermediate arylzinc, [ArZnCl] (**16**), has been tentatively identified by in situ ^19^F NMR, and the [L_2_NiCl_2_] (**17**) coproduct from Ni-to-Zn transmetalation, step 4, is recycled via Zn reduction, step 1. The intermediacy of arylzinc **16** also provides a route to traces of Ar–H (**3**) through adventitious water.^40^

As the reaction progresses, the [ZnCl_2_]-co-product accumulates in solution and has two major roles: it accelerates step 4 and reversibly abstracts halide (*K*_M1_, step 7) from [L_2_Ni^II^Cl_2_] (**17**) to generate a phosphine-free mixed metalate **20**_DMF_ that is presumed to be an off-cycle reservoir of Ni^II^. The combined effect is a progressive reduction in the concentration of the Ar–Ni^II^ intermediate (**6**) accompanied by its more efficient transmetalation (*k*_TM1_, step 4), resulting in a mildly sigmoidal profile for the generation of biaryl **2**.^41^ Chloride abstraction (*K*_M1_, step 7) also reduces the extent of comproportionation of Ni^II^ complex **17** with Ni^0^, (*K*_CD_, step 8). When the coupling is initiated with too low a concentration of the phosphine [PAr_3_], the rise in [ZnCl_2_] concentration results in an abrupt stalling of catalysis through off-cycle speciation, *K*_M1_, step 7. Turnover can be restored by the addition of further phosphine, Figure 2B. Catalysis conducted in DMAc instead of DMF is much less prone to this stalling due to steric crowding in the analogous mixed metalate **20**_DMAc_ that thus reduces *K*_M1_.

### 3.2 Induction and Mutual Antagonism

When catalysis is initiated without in situ Ni^II^-reduction, e.g., using [(L)_*n*_Ni^0^] generated by combination of [Ni(COD)_2_] (step 9) and Ar_3_P then [ZnCl_2_]_0_ = 0 and transmetalation (step 4) is not initially feasible. Under these conditions, Ar–Ni^II^ intermediate **6** can still slowly undergo turnover by dinuclear metathesis (*k*_D_’, step 10).^25,42^ This bypass mechanism allows the accumulation of endogenous [ZnCl_2_], step 1, required for acceleration to the normal turnover velocity,^28^Figure 4, via steps 4 and 5.

When catalysis is initiated in the presence of an exogenous chloride ion, [M^+^][Cl^–^], turnover initially proceeds via rapid anionic dinuclear metathesis (*k*_Cl_’, step 11). The chloride ion also binds strongly to the accumulating [ZnCl_2_],^29^ step 12, resulting in the shut-down of transmetalation, step 4, accompanied by a reduction in the rate of dinuclear metathesis (step 11). These mutually antagonistic effects mean that turnover can “flip” from one cycle to the other depending on the stoichiometry of the added chloride. In other words, [Cl^–^]_0_ > 0.5[**1**]_0_ is required for dinuclear metathesis (step 11) to dominate throughout. Under these conditions, the system will then also bypass the [ZnCl_2_]-induced inhibition arising from off-cycle speciation, *K*_M1_, step 7.

### 3.3 General Insights

Polar coordinating solvents, e.g., DMF, DMAc, and NMP, enable the efficient Ni/PPh_3_-catalyzed Zn-mediated homocoupling of aryl chlorides. This likely reflects several requirements, including the need to sustain substantial [ZnCl_2_] concentrations and the facilitation of Ni-to-Zn and Zn-to-Ni transmetalations, the ability of DMF to stabilize Ni-complexes that have undergone dissociation of phosphine ligand(s), e.g., **8**, or loss of halide, e.g., **20**_DMF_, and the attenuation of phosphine-[ZnCl_2_] complexation which occurs in other solvents.^23a^ When the chloride ion is in excess throughout, the homocoupling proceeds by an anionic dinuclear pathway. Features such as chemoselectivity or stereoselectivity will thus depend on which pathway(s) are followed and may change abruptly when [Cl^–^]/[ZnCl_2_] ratios invert.

The key roles of [ZnCl_2_] also suggest a solution to the long-standing anomaly that other strongly reducing metals that were tested, including Fe, Al, Na, and Ca, as well as some 2-electron organic donors, were found to be much less effective than Zn in inducing turnover of the Ni/PPh_3_-catalyzed homocoupling of aryl chlorides in DMF.^5−8,10^ It is also of note that both Mn and Mg can be used in place of Zn, as originally reported by Colon,^7a^ suggesting the possibility of analogous transmetalations (steps 4 and 5).

Whether these general findings translate to other Zn-mediated Ni-catalyzed couplings using other ligands in place of monodentate phosphines such as PPh_3_ is beyond the scope of the current study.^26^ Nonetheless, the processes and equilibria summarized in Figure 8 may be important considerations in the control of Ni-catalyzed arylhalide cross-electrophile coupling using bipyridine and related ligands.^11−13^ Aryl halide homocoupling is clearly an undesired pathway in these reactions, and radical initiators,^11^ the use of other reductants, e.g., tetrakis(dimethylamino)ethylene in non-amide solvent,^14a^ and MgCl_2_-additives can be effective in bypassing this.^11a^

It is of note that, for the phosphine system explored herein, acceleration by iodide is substantial but not mutually antagonistic with [ZnCl_2_].^29^ The use of an iodide additive in combination with exogenous chloride may therefore be a method to accelerate the desired coupling while simultaneously suppressing the aryl-transmetalation pathway.^40^

In summary, the Ni-catalyzed homocoupling originally developed by Kumada et al.,^5^ and Colon et al.,^7a^ can be viewed as an “endogenous Negishi coupling”,^43,44^ in which the Ni also formally catalyzes the generation of a transient arylzinc reagent from Zn powder and Ar–Cl. The accumulating [ZnCl_2_] acts as the autococatalyst, with the exogenous chloride ion acting to suppress this. The overarching network shown in Figure 8 accounts for the complex and initially paradoxical features observed under the catalyzed coupling conditions. The mildly sigmoidal evolution of **2** arises from increasing [ZnCl_2_] concentration exerting both acceleration of biaryl generation from **6** and reversibly sequestering Ni^II^ in the form of the phosphine-free mixed metalate **20**_DMF_ (*K*_M1_, step 7). The reaction therefore requires a high phosphine concentration to maintain the Ni^II^ that is on-cycle (∼[PPh_3_]^2^/*K*_M1_[ZnCl_2_]) and available for reduction (step 1).^10f^

## Data Availability

The data underlying this study are available in the published article and its Supporting Information.

## Supporting Information Available

Experimental details, characterization data, reaction profiles, kinetic analyses, titrations, determination of the solubility of [ZnCl_2_], NMR and EPR spectra, and X-ray diffraction data (PDF)

## References

[1] aVasconcelos S. N. S.; Reis J. S.; de Oliveira I. M.; Balfour M. N.; Stefani H. A. Synthesis of symmetrical biaryl compounds by homocoupling reaction. Tetrahedron 2019, 75, 1865–1959. 10.1016/j.tet.2019.02.001.

[2] aSemmelhack M. F.; Helquist P. M.; Jones L. D. Synthesis with Zerovalent Nickel. Coupling of Aryl Halides with Bis(1,5-Cyclooctadiene)Nickel(0). J. Am. Chem. Soc. 1971, 93, 5908–5910. 10.1021/ja00751a062.

[3] aNelson T. D.; Crouch R. D. Cu, Ni, and Pd Mediated Homocoupling Reactions in Biaryl Syntheses: The Ullmann Reaction; John Wiley and Sons, Inc, 2004; pp 265–555. 10.1002/0471264180.or Organic Reactions

[4] Kende A. S.; Liebeskind L. S.; Braitsch D. M. In Situ generation of a solvated zerovalent nickel reagent. Biaryl formation. Tetrahedron Lett. 1975, 16, 3375–3378. 10.1016/S0040-4039(00)91402-3.

[5] Zembayashi M.; Tamao K.; Yoshida J.; Kumada M. Nickel-Phosphine Complex-Catalyzed Homo Coupling of Aryl Halides in the Presence of Zinc Powder. Tetrahedron Lett. 1977, 18, 4089–4091. 10.1016/S0040-4039(01)83434-1.

[6] aTakagi K.; Hayama N.; Inokawa S. Synthesis of Biaryls from Aryl Iodides and Zinc Powder by Means of Nickel Catalyst. Chem. Lett. 1979, 8, 917–918. 10.1246/cl.1979.917.

[7] aColon I.; Kelsey D. R. Coupling of Aryl Chlorides by Nickel and Reducing Metals. J. Org. Chem. 1986, 51, 2627–2637. 10.1021/jo00364a002.

[8] aTiecco M.; Tingoli M.; Testaferri L.; Chianelli D.; Wenkert E. Total Synthesis of Orellanine: The Lethal Toxin of Cortinarius Orellanus Fries Mushroom. Tetrahedron 1986, 42, 1475–1485. 10.1016/S0040-4020(01)87367-1.

[9] aChen W.-W.; Zhao Q.; Xu M.-H.; Lin G.-Q. Nickel-Catalyzed Asymmetric Ullmann Coupling for the Synthesis of Axially Chiral Tetra-Ortho-Substituted Biaryl Dials. Org. Lett. 2010, 12, 1072–1075. 10.1021/ol1000632. 20112963

[10] aIyoda M.; Otsuka H.; Sato K.; Nisato N.; Oda M. Homocoupling of Aryl Halides Using Nickel(II) Complex and Zinc in the Presence of Et_4_NI. An Efficient Method for the Synthesis of Biaryls and Bipyridines. Bull. Chem. Soc. Jpn. 1990, 63, 80–87. 10.1246/bcsj.63.80.

[11] aWang S.; Qian Q.; Gong H. Nickel-Catalyzed Reductive Coupling of Aryl Halides with Secondary Alkyl Bromides and Allylic Acetate. Org. Lett. 2012, 14, 3352–3355. 10.1021/ol3013342. 22697415

[12] aDurandetti M.; Gosmini C.; Périchon J. Ni-catalyzed activation of α-chloroesters: a simple method for the synthesis of α-arylesters and β-hydroxyesters. Tetrahedron 2007, 63, 1146–1153. 10.1016/j.tet.2006.11.055.

[13] aEverson D. A.; Weix D. J. Cross-Electrophile Coupling: Principles of Reactivity and Selectivity. J. Org. Chem. 2014, 79, 4793–4798. 10.1021/jo500507s. 24820397 PMC4049235

[14] aAnka-Lufford L. L.; Huihui K. M.; Gower N. J.; Ackerman L. K.; Weix D. J. Nickel- Catalyzed Cross-Electrophile Coupling with Organic Reductants in Non-Amide Solvents. Chem. - Eur. J. 2016, 22, 11564–11567. 10.1002/chem.201602668. 27273457

[15] Although N,N-type ligands, such as bipyridines and bisoxazolines have dominated recent studies in Ni-catalyzed reductive couplings, there has been renewed interest in tuning the properties of monophosphines, see Wu K.; Doyle A. G. Parameterization of phosphine ligands demonstrates enhancement of nickel catalysis via remote steric effects. Nat. Chem. 2017, 9, 779–784. 10.1038/nchem.2741. 28754948 PMC5609847

[16] aTasker S. Z.; Standley E. A.; Jamison T. F. Recent Advances in Homogeneous Nickel Catalysis. Nature 2014, 509, 299–309. 10.1038/nature13274. 24828188 PMC4344729

[17] aTsou T. T.; Kochi J. K. Mechanism of Biaryl Synthesis with Nickel Complexes. J. Am. Chem. Soc. 1979, 101, 7547–7560. 10.1021/ja00519a015.

[18] aDiccianni J. B.; Hu C. T.; Diao T. Insertion of CO_2_ Mediated by a (Xantphos)Ni^I^–Alkyl Species. Angew. Chem., Int. Ed. 2019, 58, 13865–13868. 10.1002/anie.201906005. PMC8905775 31309669

[19] Ben-Tal Y.; Boaler P. J.; Dale H. J. A.; Dooley R. E.; Fohn N. A.; Gao Y.; García-Domínguez A.; Grant K. M.; Hall A. M. R.; Hayes H. L. D.; Kucharski M. M.; Wei R.; Lloyd-Jones G. C. Mechanistic Analysis by NMR Spectroscopy: A Users Guide. Prog. Nucl. Magn. Reson. Spectrosc. 2022, 129, 28–106. 10.1016/j.pnmrs.2022.01.001. 35292133

[20] aDick D. G.; Stephan D. W.; Campana C. F. The crystal and molecular structure of the coordinatively unsaturated Ni(0) species Ni(PPh_3_)_3_. Can. J. Chem. 1990, 68, 628–632. 10.1139/v90-096.

[21] aNakamura A.; Otsuka S. Pathways of Thermal Aryl Transfer from Coordinated Triarylphosphines to Nickel. Tetrahedron Lett. 1974, 15, 463–466. 10.1016/S0040-4039(01)82244-9.

[22] The reduced form of the catalyst, see ref (20), has limited solubility in DMF, with precipitation noted at about 0.07M, see Supporting Information Section S4.1.4. This constrains more detailed exploration of eq 2, and limits the rates attainable with this catalyst system.

[23] aChauhan A. K. S.; Singh N.; Srivastava R. C. Synthesis and characterization of triarylphosphine complexes of zinc(II) halides. Appl. Organometal. Chem. 2003, 17, 856–859. 10.1002/aoc.527.

[24] Gao Y.; Hall A. M. R.; Fohn N. A.; King E. J.; Mitchell L. A. L.; Steedman G. A.; Lloyd-Jones G. C. A Simple Device for Automated Mixing of Heterogeneous Solid-Liquid Reactions During In-Situ Monitoring by NMR Spectroscopy. Eur. J. Org Chem. 2024, 27, e2024400095 10.1002/ejoc.202400095.

[25] Recent studies from Stahl, Paton, and Weix, and co-workers, see ref (26), have shown that Ni^I^-to-Ni^II^ aryl transfer can be stimulated by partial reduction of [Ni^II^(bipy)(Ar)Br] to generate [Ni^I^(bipy)Ar]. The data presented herein indicates that an analogous pathway for [(Ar_3_P)_2_Ni^II^(Ar)Cl] is not stimulated by Zn-metal in DMF.

[26] Romero-Arenas A.; Popescu M.; Goetz M.; Sanders K.; Guzei I.; Rafiee M.; Weix D.; Paton R.; Stahl S. Reductively Induced Aryl Transmetalation: An Alternative Catalytically Relevant Ni-Catalyzed Biaryl Coupling Mechanism. 10.26434/chemrxiv-2024-00 PMC13214163 40493584

[27] The computed barrier in THF at 298 K (M06/cc-pVTZ PCM(THF) ≥ 31.5 kcal mol^–1^; ref (17e)) is a little higher than that experimentally determined herein in DMF at 333 K. The empirical second order rate constant, *k*_D_, predicted from eqs 4 and 5 when [PAr_3_] = 1 M is 2.4 × 10^–3^ M^–1^ s^–1^. This would correspond to a barrier of approximately 23.6 kcal mol^–1^ when [**6**] = 1 M.

[28] At 3 mM [ZnCl_2_] the rate is 2-fold supressed compared to *k*_D_, eq 3, before accelerating when [ZnCl_2_] > 6 mM. This suggests that there is catalysis (*k*_Cl_) by trace endogenous chloride ∼ 0.5 mM, possibly NiCl_2_-derived, when [ZnCl_2_] = 0; see Supporting Information Section S8.4.

[29] Ishiguro S.-I.; Miyauchi M.; Ozutumi K. Thermodynamics of Formation of Binary and Ternary Complexes of Zinc(II) with Halide and Thiocyanate Ions and 2,2’-Bipyridine in Dimethylformamide. J. Chem. Soc., Dalton Trans. 1990, 2025–2041.

[30] aThis is not a consequence of exchanging Ph_3_P for Ar_3_P: this induces a marginal increase in rate, see Supporting Information Section S4.1.12.bThe predicted turnover rate is greater than that observed, indicative that the approximation [ZnCl_2_]_t_ ≈ [**2**]_t_ overestimates the [ZnCl_2_] concentration, possibly through aggregation or autionization equilibria to generate [ZnCl_3_(DMF)]^−^[ZnCl(DMF)_3_]^+^.

[31] ZnX_2_ mediated X-abstraction from Ni-benzyl complexes has been reported, see for example: Anderson T. J.; Vicic D. A. Direct Observation of Noninnocent Reactivity of ZnBr_2_ with Alkyl Halide Complexes of Nickel. Organometallics 2004, 23, 623–625. 10.1021/om034380j.

[32] aNohira I.; Chatani N. Nickel-Catalyzed Cross-Electrophile Coupling between C(sp2)–F and C(sp2)–Cl Bonds by the Reaction of ortho-Fluoro-Aromatic Amides with Aryl Chlorides. ACS Catal. 2021, 11, 4644–4649. 10.1021/acscatal.1c01102.

[33] See footnote 62 in: Akana M. E.; Tcyrulnikov S.; Akana-Schneider B. D.; Reyes G. P.; Monfette S.; Sigman M. S.; Hansen E. C.; Weix D. J. Computational Methods Enable the Prediction of Improved Catalysts for Nickel-Catalyzed Cross-Electrophile Coupling. J. Am. Chem. Soc. 2024, 146, 3043–3051. 10.1021/jacs.3c09554. 38276910

[34] bFor comparator rate constants (*k*_obs_ in the range 0.04–0.31 M^–1^ s^–1^) see for example Jin L.; Xin J.; Huang Z.; He J.; Lei A. Transmetalation is the Rate-Limiting Step: Quantitative Kinetic Investigation of Nickel-Catalyzed Oxidative Coupling of Arylzinc Reagents. J. Am. Chem. Soc. 2010, 132, 9607–9609. 10.1021/ja1045296. 20583839

[35] aCarter E.; Murphy D. M. The Role of Low Valent Transition Metal Complexes in Homogeneous Catalysis: An EPR Investigation. Top. Catal. 2015, 58, 759–768. 10.1007/s11244-015-0417-6.

[36] aHevia E.; Chua J. Z.; Garcia-Alvarez P.; Kennedy A. R.; McCall M. D. Exposing the hidden complexity of stoichiometric and catalytic metathesis reactions by elucidation of Mg-Zn hybrids. Proc. Nat. Acad. Sci. 2010, 107, 5294–5299. 10.1073/pnas.0913307107. 20212145 PMC2851795

[37] This equilibrium constant represents the net effect of a series of complex exchanges, and the telescoped estimate that *K*_M1_ ∼ 5 M does not include the solvent (DMF) concentration.

[38] The term “semicatalytic” is used herein to describe the system evolving in the absence of Zn-reductant. The generation of biaryl (**2**) from 2 [(Ar_3_P)_2_Ni^II^(Ar)Cl] (**6**) generates 2 Ni^I^ = Ni^II^ + Ni^0^. Thus, half of the Ni-coproduct from this first step can add ArCl (**1**) to regenerate Ar-Ni^II^ (**6**). This again can generate biaryl **2**, and 0.5 Ni^0^, to be recycled, and so on. Thus, based on the initial quantity of Ar-Ni^II^ (**6**), the semicatalytic yields are 0.5, 0.25, 0.125, 0.0625, etc. In the limit, this results in an overall process in which Ar-Ni^II^ (**6**) + Ar-Cl (**1**) = Ar-Ar (**2**) + Ni^II^, corresponding to a 100% yield of **2** based on **6**.

[39] Seka S.; Buriez B.; Périchon J. Stabilization of Co^I^ by Zn^II^ in Pure Acetonitrile and its Reaction with Aryl Halides. Chem.—Eur. J. 2003, 9, 3597–3603. 12898686 10.1002/chem.200204604

[40] Our preliminary exploration of the effect of water, see Supporting Information Section S5.1.13, shows this attenuates turnover, and suggests a very large isotope effect accompanies deuterodezincation with D_2_O. This is very different to the iodide-co catalyzed process developed by Colon, see ref (7b), for which the KIE is only moderate, suggesting that iodide additives accelerate an alternative pathway that does not involve direct dinuclear or Ni-to-Zn transmetalation reaction of Ar-Ni^II^ complexes. Mechanisms A,B Figure 1, amongst other possibilities, would be consistent with this.

[41] Using eq 14 to calculate the instantaneous rate under the catalyzed homocoupling conditions based on the observed concentrations of intermediate **6**, allows estimation of the predicted temporal concentration profile for **2**. While not a perfect correlation, is does reproduce the observed mildly sigmoidal behavior and reaction lifetimes; see also ref (30b).

[42] Material balance suggests that in the absence of high [ZnCl_2_] concentrations, there may be some comproportionation of Ar-Ni^II^ complex **6** with Ni^0^ to generate undetected Ar-Ni^I^ species and, presumably, Ni^I^Cl complex **7**. However, for the phosphine-ligated system, this does not induce efficient aryl transfer, or oxidative addition-reductive elimination, to generate biaryl **2**.

[43] Kumada noted in 1977 ref (5) that “Organozinc compounds have recently been shown to couple with aryl halides in the presence of a nickel-phosphine complex as catalyst” (citing ref (44) herein) and that “Although the exact mechanism of the present coupling reaction has not yet been clarified, it seems likely that organozinc intermediates are not involved”.

[44] Negishi E.; King A. O.; Okukado N. Selective carbon-carbon bond formation via transition metal catalysis. 3. A highly selective synthesis of unsymmetrical biaryls and diarylmethanes by the nickel- or palladium-catalyzed reaction of aryl- and benzylzinc derivatives with aryl halides. J. Org. Chem. 1977, 42, 1821–1823. 10.1021/jo00430a041.

