# Supplementary material for: Kinetics and Mechanism of PPh3/Ni-Catalyzed, Zn-Mediated, Aryl Chloride Homocoupling: Antagonistic Effects of ZnCl2/Cl–
Source: J Am Chem Soc. 2024 Oct 18;146(43):29913–27. doi: 10.1021/jacs.4c12088 (PMC11528415; doi:10.1021/jacs.4c12088)
Supplement: Supplementary file 1 — ja4c12088_si_001.pdf [file ja4c12088_si_001.pdf]

Supporting Information

Kinetics and Mechanism of PPh_3/Ni-Catalyzed, Zn-Mediated, Aryl Chloride Homocoupling: Antagonistic Effects of $ZnCl_2/Cl^-$

Nicole A. Fohn†, Yuan Gao†, Stephen Sproules‡, Gary S. Nichol,† Colin M. Brennan§, Alan J. Robinson||, and Guy C. Lloyd-Jones†*

† University of Edinburgh, Joseph Black Building, David Brewster Road, Edinburgh, EH9 3FJ, UK

‡ University of Glasgow, Joseph Black Building, University Ave, Glasgow, G12 8QQ, UK

§ Jealott's Hill International Research Centre, Syngenta, Bracknell, Berkshire RG42 6EY, U.K

||Syngenta Crop Protection AG Research Centre, Syngenta, Schaffhauserstrasse, Muenchwillen, 4334 Switzerland

Email: guy.lloyd-jones@ed.ac.uk

Contents

S1 General Procedures 4

S1.1 Chemicals and Solvents 4

S1.2 Instruments 4

S1.2.1 NMR Spectroscopy 4
S1.2.2 EPR Spectroscopy 4
S1.2.3 IR Spectroscopy 4
S1.2.4 MALDI Spectrometry 4
S1.2.5 UV-VIS Spectroscopy 4

S2 Synthetic Procedures 5

S2.1.1 Synthesis of $L_2NiArCl$ (6) 5
S2.1.2 Synthesis of di(4-fluorophenyl)zinc (19) 7
S2.1.3 Synthesis of mixed metallate 21 8
S2.1.4 Synthesis of *N*-(2-hydroxy-5-methylphenyl)salicylideneimine 9

S3 In situ Analysis of Catalyst Activation 10

S4 Catalytic Experiments 11

S4.1 Ex-situ Monitoring 11

S4.1.2 Standard Procedure for the Nickel Catalysed Homocoupling 12
S4.1.3 Control Experiments 14
S4.1.4 Impact of Catalyst Concentration on the Rate of Homocoupling 15
S4.1.5 Control experiment for Internal Standard (1-fluoronaphthalene) 17
S4.1.6 Variation in Substrate Concentration 18
S4.1.7 Variation of the Zinc Loading 19
S4.1.8 Reduction in the PPh_3 Concentration 20
S4.1.9 Homocoupling with Endogenous $ZnCl_2$ 22
S4.1.10 Homocoupling using $NiCl_2$ as Pre-catalyst. 24
S4.1.11 Homocoupling Initiated using other Nickel Pre-Catalysts 25
S4.1.12 Homocoupling with tri(para-fluorophenyl)phosphine (PAr_3) 27
S4.1.13 Effect of Reduction in Overall Concentration on the Homocoupling 28
S4.1.14 Homocoupling at 80 °C 29
S4.1.15 Homocoupling without Pre-activation 30
S4.1.16 Addition of Further Substrate after Completion of the Homocoupling 32
S4.1.17 Homocoupling of 1-chloro-3-fluorobenzene 33
S4.1.18 Homocoupling with Deliberate Exposure to Air 34
S4.1.19 Homocoupling in the Presence of H_2O and D_2O 36
S4.1.20 Homocoupling in the Presence of Sodium Halide Salts 38
S4.1.21 Homocoupling in the Presence of Chloride Additives (MCl) 40
S4.1.22 Homocoupling in Toluene 41
S4.1.23 Homocoupling in DMAc 42
S4.1.24 Effect of reduced ex situ Sampling Frequency on the Homocoupling 44
S4.1.25 Effect of Reduced Stirring Rate on the Homocoupling 45

S4.2 Homocoupling with In Situ Reaction Monitoring using a Mixing Device[S6] *..... 46*

S4.3 Alternating-Agitation-Heating Experiments 49

S4.3.2 Standard Procedure 49

S5 Stoichiometric Homocoupling Experiments 52

S5.1 In-situ Monitoring ... *52*
S5.1.2 Standard Procedure ... 52
S5.1.3 Homocoupling of $L_2NiArCl$ (6) at Various $ZnCl_2$ Concentrations ... 56
S5.1.4 Direct Homocoupling of $L_2NiArCl$ (6) at Various PAr_3 Concentrations ... 60
S5.1.5 $ZnCl_2$ Catalysed Homocoupling of $L_2NiArCl$ (6) at Various PAr_3 Concentrations ... 62
S5.1.6 Semicatalytic Homocoupling of $L_2NiArCl$ (6) and ArCl ... 64
S5.1.7 Semicatalytic homocoupling of ArCl (1) by $[Ni(PAr_3)_n(DMF)_m]$ and $[(PAr_3)_3NiCl]$. ... 66
S5.1.8 Inhibition of the $ZnCl_2$-accelerated Homocoupling of $L_2NiArCl$ (6) by $[(PAr_3)_2NiCl_2]$ at raised PAr_3 concentration. ... 68
S5.1.9 Direct Homocoupling of $L_2NiArCl$ (6) in the Presence of $[(PAr_3)_2NiCl_2]$. ... 70
S5.1.10 Inhibition of the $ZnCl_2$-accelerated Homocoupling of $L_2NiArCl$ (6) by $[(PAr_3)_2NiCl_2]$ ... 72
S5.1.11 Reaction of $L_2NiArCl$ (6) and $[(PAr_3)_2NiCl_2]$ (17) with ZnArCl (16) and $ZnAr_2$ (19) ... 74
S5.1.12 Homocoupling of $L_2NiArCl$ (6) in the presence of NaI ... 76
S5.1.13 $ZnCl_2$ and Cl^- accelerated Homocoupling of $L_2NiArCl$ (6) in the presence of H_2O ... 78
S5.1.14 Effect of added $NaBAr^F$ on the Stoichiometric Homocoupling of $L_2NiArCl$ (6) ... 80
S5.1.15 Cl^- accelerated Homocoupling of $L_2NiArCl$ (6) ... 82

S5.2 NMR Titrations and Equilibria ... *85*
S5.2.1 Titration of $NiCl_2$·glyme and PAr_3 with $ZnCl_2$ in DMF ... 85
S5.2.2 Titration of $NiCl_2$·glyme and PAr_3 with $ZnCl_2$ in DMAc ... 88
S5.2.3 Titration of $[NiCl_2(glyme)]$ with PAr_3 ... 90
S5.2.4 Equilibration of $ZnAr_2$ (19) + $ZnCl_2$ with ArZnCl (16) ... 92
S5.2.5 Titration of $ZnCl_2$ with PAr_3 ... 94
S5.2.6 Titration of LiCl with $ZnCl_2$ ... 96

S5.3 Low Temperature ^{31}P NMR Spectroscopic Studies ... *98*

S6 UV-Vis Spectroscopic Analyses ... **100**

S6.1 UV-Vis Analysis of the Mixed Metallate 21_{DMF} ... *100*
S6.1.2 UV-VIS Measurements ... 100

S6.2 Estimation of the limiting solubility of $ZnCl_2$ in DMF and DMAc. ... *102*
S6.2.2 Solubility of $ZnCl_2$ in DMF ... 102
S6.2.3 Solubility of $ZnCl_2$ in DMAc ... 102
S6.2.4 UV-VIS Measurements ... 103
S6.2.5 Calculation and Plots ... 104

S7 EPR Spectroscopic Analysis of Ni Speciation ... **106**

S7.1.2 Reaction 1: Stoichiometric Homocoupling of $L_2NiArCl$ (6) without $ZnCl_2$ ... 106
S7.1.3 Reaction 2: Stoichiometric Homocoupling of $L_2NiArCl$ (6) with $ZnCl_2$ ... 106
S7.1.4 Reaction 3: $[Ni(COD)_2]$ and $[NiCl_2(glyme)]$ Comproportionation without $ZnCl_2$ ... 106
S7.1.5 Reaction 4: $[Ni(COD)_2]$ and $[NiCl_2(glyme)]$ Comproportionation with $ZnCl_2$ ... 106

S8 Analyses of kinetic data. ... **109**

S9 IR Spectra ... **115**

S10 NMR Spectra ... **119**

S11 MALDI ... **136**

S12 Single Crystal X-ray Diffraction ... **138**

S13 References ... **144**

S1 General Procedures

S1.1 Chemicals and Solvents

All reagents were purchased commercially (Fluorochem, SigmaAldrich, AlphaAesar). DMF, DMAc and 1-fluoronaphthalene (used as an internal standard, IS, for ^{19}F NMR spectroscopy) were distilled under vacuum, and 1-chloro-4-fluorobenzene (**1**) was cryo-degassed to remove oxygen from solution. Solvents and substrates were stored in the glovebox (N_2-filled) over molecular sieves. Triphenylphosphine (PPh_3) and tris(4-fluorophenyl)phosphine (PAr_3) were dried under vacuum for several days before they were stored in the glovebox. Zn powder (mesh 325), Ni(COD)$_2$ and zinc chloride ($ZnCl_2$) were supplied under an inert atmosphere and directly introduced into the glovebox for storage.

S1.2 Instruments

S1.2.1 NMR Spectroscopy

^{1}H, ^{19}F, ^{31}P, ^{7}Li and ^{13}C NMR spectra were acquired on a Bruker Avance III HD 400 MHz spectrometer with a 5 mm BBO CryoProbe Prodigy (LN2). The low temperature NMR experiments (S5.3) were acquired on a Brucker Avance III 400 MHz spectrometer with a BBFO+ probe. Chemical shifts are reported in parts per million (ppm) and splitting patterns given as multiplicities (s for singlet, d for doublet, t for triplet, q for quartet, quint for quintet and m for multiplet). ^{1}H, ^{2}D and ^{13}C NMR spectra were referenced to residual solvent peaks; ^{19}F NMR spectra were referenced to 1-chloro-4-fluorobenzene (**1**) or 1-fluoronaphthalene, and employed a 30 degree pulse angle in combination with a 10 second interscan relaxation delay; ^{31}P NMR spectra were referenced to triphenylphosphine oxide. NMR tubes were cleaned with aqua regia, then quenched with aqueous $NaHCO_3$, and washed with water and acetone before being dried in an oven and/or under vacuum. Spectra were processed with MestreNova.

S1.2.2 EPR Spectroscopy

EPR spectra were acquired using a Bruker ELEXSYS E500 spectrometer and simulations were using the Bruker's Xsophe software package.[S1]

S1.2.3 IR Spectroscopy

IR spectra were acquired using a Bruker ALPHA™ ATR-FTIR spectrometer.

S1.2.4 MALDI Spectrometry

MS spectra were acquired using a Bruker ultrafleXtreme MALDI-TOF-TOF.

S1.2.5 UV-VIS Spectroscopy

UV-VIS spectra were acquired on OceanOptics USB4000 and Flame Spectrometers. The system was equipped with a thermostatic cuvette holder, a DH2000-BALUV lamp and solarised resistant grade optical fibres. Data were processed in Kinetics Studio.

S2 Synthetic Procedures

S2.1.1 Synthesis of $L_2NiArCl$ (6)

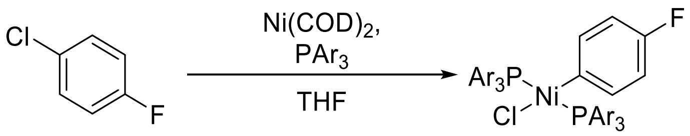

1 **6**

*Scheme S1: Synthesis of $L_2NiArCl$ (**6**) from **1**, $Ni(COD)_2$ and PAr_3.*

The synthesis was adapted from a literature procedure for the preparation of $[(PPh_3)_2NiCl(Ph)]$.[S2]

This procedure was conducted in the glovebox.

$Ni(COD)_2$ (145 mg, 0.53 mmol), tris(4-fluorophenyl)phosphine (PAr_3, 655 mg, 2.2 mmol) and THF (5 mL) were added to a vial equipped with a stirring bar. The solution was dark red. After 1h the reaction mixture was cooled to -20 °C and 1-chloro-4-fluorobenzene (**1**, 60 µL, 0.56 mmol) was added. The reaction was stirred overnight. The stirring was stopped, a layer of hexane (20 mL) was carefully added, and the solution was left for 2 days while crystallisation occurred. The resulting orange crystals of $[(PAr_3)_2NiCl(Ar)]$.THF were isolated by filtration, washed with hexane, and dried under N_2. Yield: 250 mg (53 %)

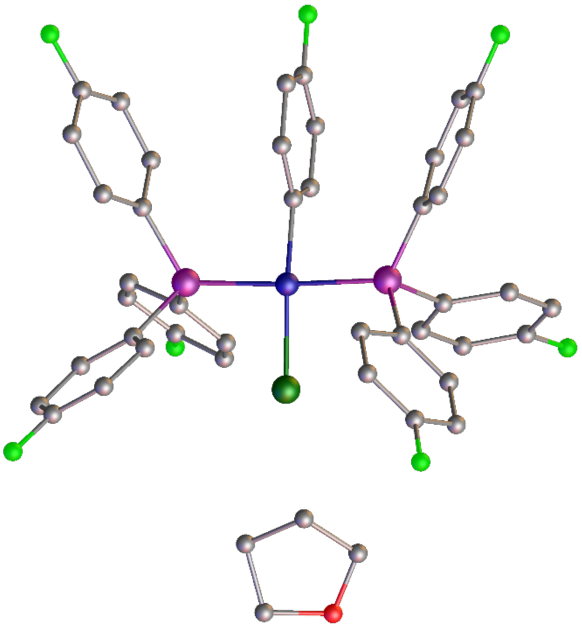

*Figure S1: X-ray crystal structure of complex (**6**). THF is present as a solvent of crystallisation in 1:1 ratio with the Ni. For coordinates and details see: CCDC 2373479*

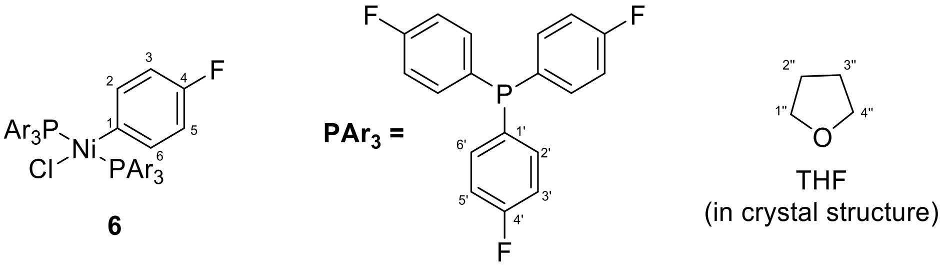

Scheme S2: Numeration of the carbon atoms in complex 6 used in the NMR data below.

^{1}H NMR (400 MHz, d_7-DMF) δ 7.62 – 7.10 (m, 24H, PAr_3, CH), 6.76 (t, J_{HH} = 8.5 Hz, 2H, Ni-*p*-PhF, CH (2,6)), 6.13 (t, J_{HH} = 8.5 Hz, 2H, Ni-*p*-PhF, CH (3,5)), 3.63 (t, J_{HH} = 6.2 Hz, 4H, THF, CH_2 (2,5)), 1.78 (quint, J_{HH} = 6.2 Hz, 4H, THF, CH_2 (3,4)). ^{19}F NMR (377 MHz, d_7-DMF) δ -110.74 (br, PAr_3), -125.72 – -126.89 (m, Ni-*p*-PhF). ^{13}C NMR (101 MHz, d_7-DMF) δ 163.95 (d, J_{CF} = 247.6 Hz, PAr_3, C-F (4')), 160.65 (d, J_{CF} = 238.52 Hz, (Ni-*p*-PhF, C-F (4)), 142.24 (br, Ni-*p*-PhF, C-Ni (1)), 137.50 (d, J_{CF} = 5.7 Hz, Ni-*p*-PhF, CH (2,6)), 136.44 (br, PAr_3, CH (2',6')), 133.61 (br, PAr_3, C-P (1')), 116.46 (d, J_{CF} = 22.3 Hz, PAr_3, CH (3',5')), 113.99 (d, J_{CF} = 19.1 Hz, Ni-*p*-PhF, CH (3,5)), 67.76 (s, THF, CH_2 (1'',4'')), 25.80 (s, THF, CH_2 (2'',3'')). ^{31}P NMR (162 MHz, d_7-DMF) δ 20.11 (br).

All NMR spectra were recorded with a large excess of PAr_3 in solution to stabilise (**6**) for long enough to conduct the characterisation. In the absence of added ligand, the complex decomposes within minutes. Co-plotted NMR spectra of complex **6** and ligand PAr_3 are provided in Section 10 section, and the IR spectrum of **6** (solid state, ATR spectrum) in Section 9. All attempts to obtain a MS spectrum (ESI, FAB, MALDI) resulted in decomposition to polynuclear Ni/Cl species and $[PAr_4]^+$; the MALDI spectrum is provided in Section 11.

The same procedure was followed for the preparation of complex **5** (for coordinates and details see CCDC 2373478) using triphenylphosphine as a ligand. Approximately 30 % ligand aryl scrambling was evident from the crystal structure. Tri(para-fluorophenyl)phosphine was used in further experiments.

S2.1.2 Synthesis of di(4-fluorophenyl)zinc (19)

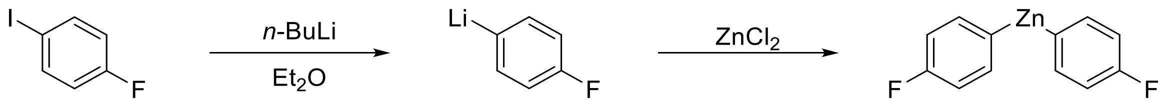

19

*Scheme S3: Synthesis of $ZnAr_2$ (**19**) from 4-fluoroiodobenzene, n-BuLi and $ZnCl_2$.*

A literature procedure was employed for the synthesis of **19**.[S3]

The reaction was set up under a nitrogen atmosphere using standard Schlenk techniques.
1-Fluoro-4-iodobenzene (1.15 mL, 10.0 mmol) was dissolved in Et_2O (30 mL) in a Schlenk flask. The reaction mixture was cooled to -60 °C and *n*-BuLi (1.5 M in hexane, 6.5 mL, 9.8 mmol) was added dropwise. The reaction was allowed to warm to 0 °C over a period of 3-4 hours. $ZnCl_2$ (680 mg, 5.0 mmol) was dissolved in Et_2O (10 mL) and added dropwise to the reaction at 0 °C. The mixture was stirred at room temperature for 4 days. The solution turned slightly yellow, and a white precipitate formed. The suspension was filtered over celite using Schlenk techniques and the filtrate collected. The Et_2O and hexane were removed under reduced pressure, and then the residue heated to 60 °C under vacuum for 4 hours, then held under vacuum overnight at room temperature. Benzene (10 mL) was added to the solid and the solution was refluxed for 1h. The solvent was removed at -5°C under vacuum. The solid was then sublimed at 90-120 °C under vacuum. The solid started to melt at 100 °C. A white powder was isolated and stored in the glovebox. Yield: 209.9 mg (8 %).

^{1}H NMR (400 MHz, benzene-d_6) δ: 6.91-7.02 ppm (m, 4H). ^{19}F NMR (377 MHz, benzene-d_6) δ: -111.84 (m). ^{13}C NMR (101 MHz, benzene-d_6) δ: 163.92 (d, J_{CF} = 246.1 Hz), 142.97 (d, J_{CF} = 4.4 Hz), 139.18 (d, J_{CF} = 6.1 Hz), 114.92 (d, J_{CF} = 18.3 Hz).

The NMR data are consistent with those reported by Hevia and co-workers.[S3]

S2.1.3 Synthesis of Mixed Metallate 21

$$NiCl_2{\cdot}glyme \; + \; ZnCl_2 \xrightarrow[DMF]{} [Ni(DMF)_6][Zn(DMF)Cl_3]_2$$

*Scheme S4: Synthesis of Ni-Zn complex (**21**) from $NiCl_2$·glyme and $ZnCl_2$ in DMF.*

A suspension of [$NiCl_2$(glyme)] (527 mg, 2.49 mmol) was prepared in 2 mL DMF. A suspension of $ZnCl_2$ (136 mg, 4.99 mmol) were prepared in 2 mL DMF in the glovebox. Both suspensions were transferred out of the glovebox, under nitrogen, and heated to 70 °C until they became homogeneous. 1 mL of each solution were taken and mixed in a small vial under argon flow. The solution immediately turned green and a white precipitate formed. The mixture was filtered using a syringe filter and the filtrate cooled in the freezer at -20 °C. A seed crystal from an unfiltered solution prepared using the same procedure was used to initiate crystallisation. The crystals were separated by decantation, carefully washed with cold DMF, then dried under vacuum, to afford **21**, 190.95 mg (32%).

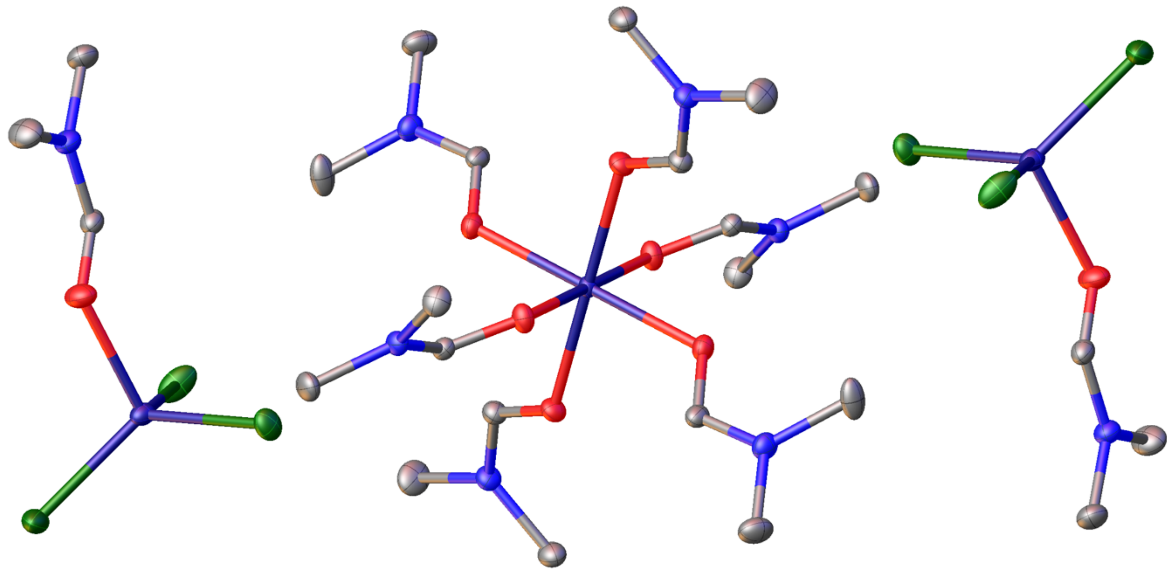

*Figure S2: Single crystal X-ray diffraction structure of **21**. For coordinates and details see CCDC 2373480*

^{1}H NMR (400 MHz, $CDCl_3$, 278 K) δ 8.07 (s, 1H, CH), 3.08 (s, 3H, N-CH_3), 2.83 (s, 3H, N-CH_3). The data indicate rapid exchange of DMF leading to time-averaged signals across all sites.

S2.1.4 Synthesis of *N*-(2-hydroxy-5-methylphenyl)salicylideneimine

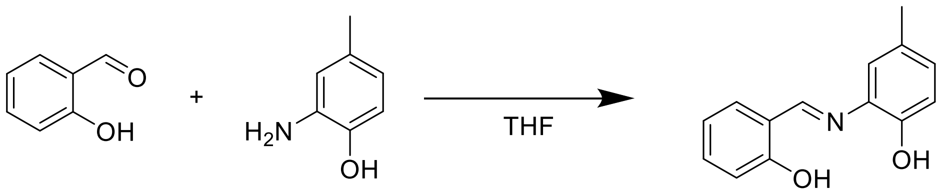

Scheme S5: Synthesis of N-(2-hydroxy-5-methylphenyl)salicylideneimine from 3-amino-4-hydroxytoluene and salicylaldehyde.

The synthesis of N-(2-hydroxy-5-methylphenyl)salicylideneimine was conducted by adaptation of a literature procedure.[S4]

3-Amino-4-hydroxytoluene (1.0 g, 8.2 mmol) was dissolved in dry THF (100 mL) in a round bottom flask. $MgSO_4$ (4 g, 33.2 mmol) and salicylaldehyde (720 µL, 6.9 mmol) were added. The reaction was magnetically stirred overnight, then filtered through a silica-gel plug, and the solvent removed under vacuum. The resulting red solid was recrystalised from EtOH at -20 °C, separated by filtration and then dried, 1.1 g (73 %).

^{1}H NMR (400 MHz, d_6-DMSO) δ: 13.81 (s, 1H), 9.46 (s, 1H), 8.95 (s, 1H), 7.59 (dd, J_{HH} = 7.5, 1.8 Hz, 1H), 7.37 (ddd, J_{HH} = 8.2, 7.5, 1.8 Hz, 1H), 7.17 (d, J_{HH} = 1.8 Hz, 1H), 7.00 – 6.87 (m, 3H), 6.84 (d, J_{HH} = 8.2 Hz, 1H), 2.24 (d, J_{HH} = 0.7 Hz, 3H).

The NMR data are consistent with those reported by Öztürk et al.[S5]

S3 In situ Analysis of Catalyst Activation

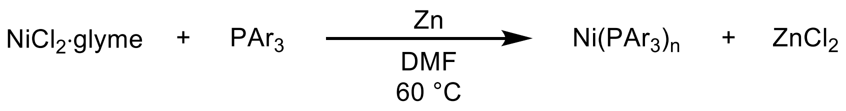

Scheme S6: Formation of $Ni(PAr_3)_n$ by reducing $NiCl_2$·glyme with Zn in the presence of PAr_3.

S3.1.1.1 Ni stock solution

In the glovebox, a solution of [$NiCl_2$(glyme)] (26.08 mg, 119 µmol), 1-fluoronaphthalene (10 µL, 77.5 µmol) and tris(4-fluorophenyl)phosphine (PAr_3, 184.14 mg, 582 µmol) was prepared in DMF (2 mL).

S3.1.1.2 Shimming and Tuning of the Spectrometer

The spectrometer was shimmed to a sample containing Zn (50 mg, 765 µmol) and Ni stock solution (0.6 mL) at 60 °C and tuned to ^{19}F.

S3.1.1.3 Reaction Mixture

This *in situ* monitoring was performed using an automated mixing device.[S6] Zn powder (50 mg, 765 µmol) and the Ni stock solution (0.6 mL) were added to an NMR tube, transferred out of the glovebox and connected to the device under argon. The device was inserted into the spectrometer and a reference spectrum acquired. Mixing was initiated. Concentrations are in Table S1.

S3.1.1.4 Spectrometer Settings

Nucleus: ^{19}F
Pulse angle: 90 degrees.
Number of scans: 1
Temperature: 333 K
Mixing time: 50 s
Settling time: 10 s
Plunger speed: 100 mm/s

Table S1: Concentrations and equivalents of reaction components for the catalyst activation.

$[IS]_0$	$[Ni]_0$	Zn_0 / eq	$[PAr_3]_0$
85.2 mM	59.3 mM	6.44 (Zn/Ni)	291.1 mM

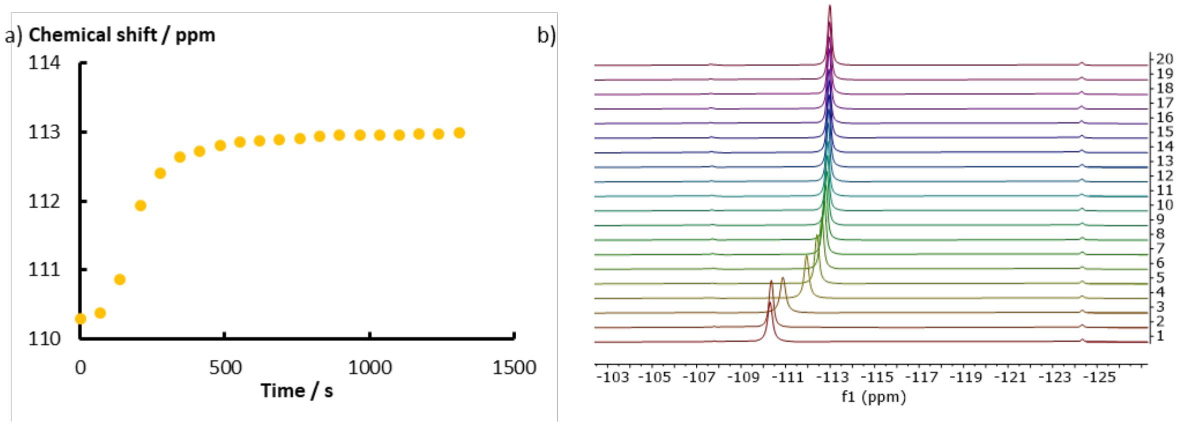

Figure S3: a) Chemical shift of the PAr_3 signal in ^{19}F NMR spectrum plotted against the reaction time for the in situ monitoring of the reduction of $NiCl_2$ in DMF. The chemical shift stabilised after 7 minutes. b) ^{19}F NMR spectra (DMF, 60 °C) showing the migration of the time-averaged chemical shift of free and Ni-complexed PAr_3.

S4 Catalytic Experiments

S4.1 *Ex-situ* Monitoring

S4.1.1.1 General Procedure

Unless stated otherwise, all reactions were set up in the glovebox in a Schlenk flask (12 cm high, 2.5 cm diameter) with a 1.5 cm stirring bar. The reactions were then transferred out of the glove box into the fume hood and further processed performed using standard Schlenk techniques (N_2). All glassware was dried in a 200 °C oven before use.

S4.1.1.2 Sampling Procedure

Unless otherwise stated, 50-100 µL were taken from the reaction and filtered through a short plug of silica-gel, washing through with 1.1 mL THF. The solution was then analysed via ^{19}F NMR. Reaction samples were taken every 10 minutes for the first 1-2 hours then every 30 minutes until the reaction was completed.

S4.1.1.3 Shimming and Tuning of the Spectrometer

The spectrometer was shimmed to each sample and tuned to ^{19}F.

S4.1.1.4 Spectrometer Settings

Nucleus: ^{19}F
Pulse sequence: zg30
Relaxation delay: 10 s
Number of scans: 8
Temperature: 300 K
For all other parameters, standard settings were used.

S4.1.1.5 Generation of temporal concentration data

Quenching studies have shown that $L_2NiArCl$ (**6**) which is present as an intermediate during the reaction decomposes to ArOH and $[PPh_3Ar]^+[X]^-$ during the quenching process (see NMR spectra in section S10). The concentration of **6** was thus estimated from the combined concentrations of the 4-fluorophenol (ArOH) and (4-fluorophenyl) triphenylphosphonium species. ArOH was identified by spiking commercial 4-fluorophenol into a quenched reaction sample. $[PPh_3Ar]^+[X]^-$ (where X is assumed, without further investigation, to be Cl) was identified by comparison to the ^{31}P and ^{19}F NMR shifts of similar compounds[S7] and by detection in MALDI mass-spectrometry (see section S11 MALDI). Fluorobenzene (**3**) was identified by spiking commercial fluorobenzene in a reaction sample and (4-fluorophenyl) diphenylphosphine (PPh_2Ar) was identified by its similarity to the ^{19}F NMR signal of commercial sample of tri(4-fluorophenyl)phosphine (PAr_3).

For all reactions with additives introduced during the monitoring, the changes in volume due to prior sampling was taken in consideration when calculating the subsequent concentrations of species.

S4.1.2 Standard Procedure for the Nickel Catalysed Homocoupling

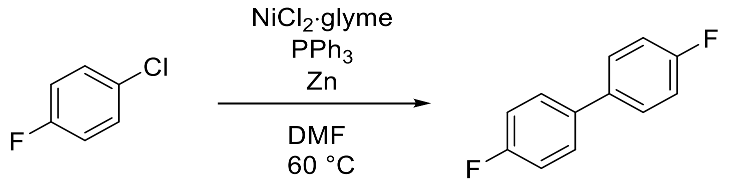

Scheme S7: Standard conditions for the Ni catalysed Ullman coupling.

The procedure was slightly adapted from the original procedure of Colon and Kelsey[S8] in that the more conveniently handled DMF-soluble complex [$NiCl_2$(glyme)] was employed instead of $NiCl_2$. The kinetics are not detectably affected by the trace concentrations of glyme that are liberated, see section S4.1.10.

[$NiCl_2$(glyme)] (27.0 mg, 123 µmol), Zn powder (250 mg, 3.82 mmol) and PPh_3 (500 mg, 1.91 mmol) were added to a Schlenk flask with a stirring bar in the glove box. DMF (3 mL) was added, resulting in a blue coloured solution. A stock solution of 1-chloro-4-fluorobenzene (**1**) (540 µL, 4.07 mmol) and 1-fluoronaphthalene (IS) (480 µL, 3.72 mmol) in DMF was made in a volumetric Schlenk flask (2 mL). Both flasks were sealed, transferred out of the glovebox and connected to the Schlenk line. The flask containing the catalyst was immersed in a preheated oil bath at 60 °C and the mixture stirred at 715 rpm. During this activation period the solution turned red. After 30 minutes, 1 mL of the stock solution was added and the solution turned orange. An aliquot was taken every 10 minutes within the first hour and then every 30 minutes until completion (see sampling procedure). After consumption of the substrate (**1**) the reaction mixture became deep red again, and was then quenched with ice-water. The samples were analysed by ^{19}F NMR spectroscopy.

The rate was estimated by calculating the slope of the product concentrations (see Figure S4).

The initial concentrations are shown in Table S2.

Table S2: Concentrations and equivalents of reaction components under standard conditions.

Concentrations are in mM, Zn is in equivalents (eq) relative to the ArCl (1)					
**$[IS]_0$**	**$[1]_0$**	**$[Ni]_0$**	**$[PPh_3]_0$**	**Zn_0 / eq**	**Rate / mM·s^{-1}**
464.8	633.9	34.3	479.7	1.54	2.04E-02

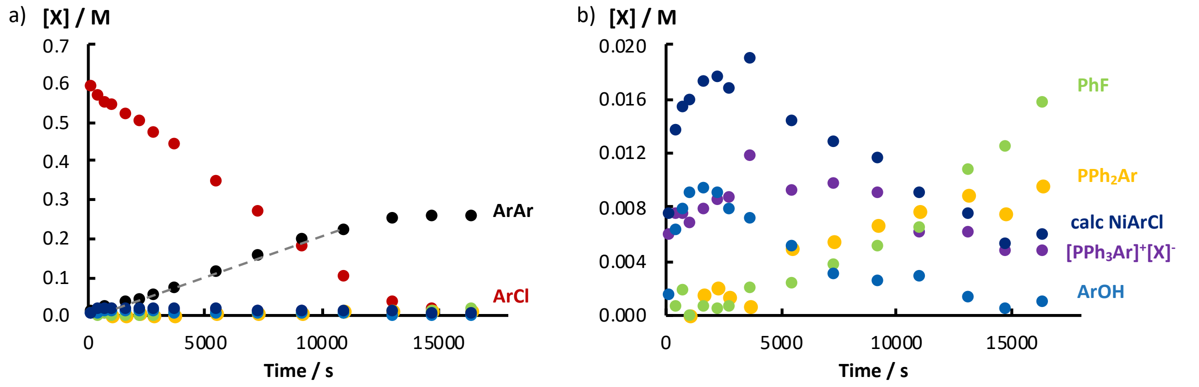

*Figure S4: a) Concentration vs time plot of the Ni catalysed homocoupling using the standard procedure. ArCl (**1**) consumption and ArAr (**2**) production are approximately linear. b) Concentration vs time plot of the intermediates and side products under standard reaction conditions. The concentration of L_2NiArCl (**5**) was calculated from the concentrations of $[PPh_3Ar]^+[X]^-$ (X is assumed to be chloride) and ArOH.*

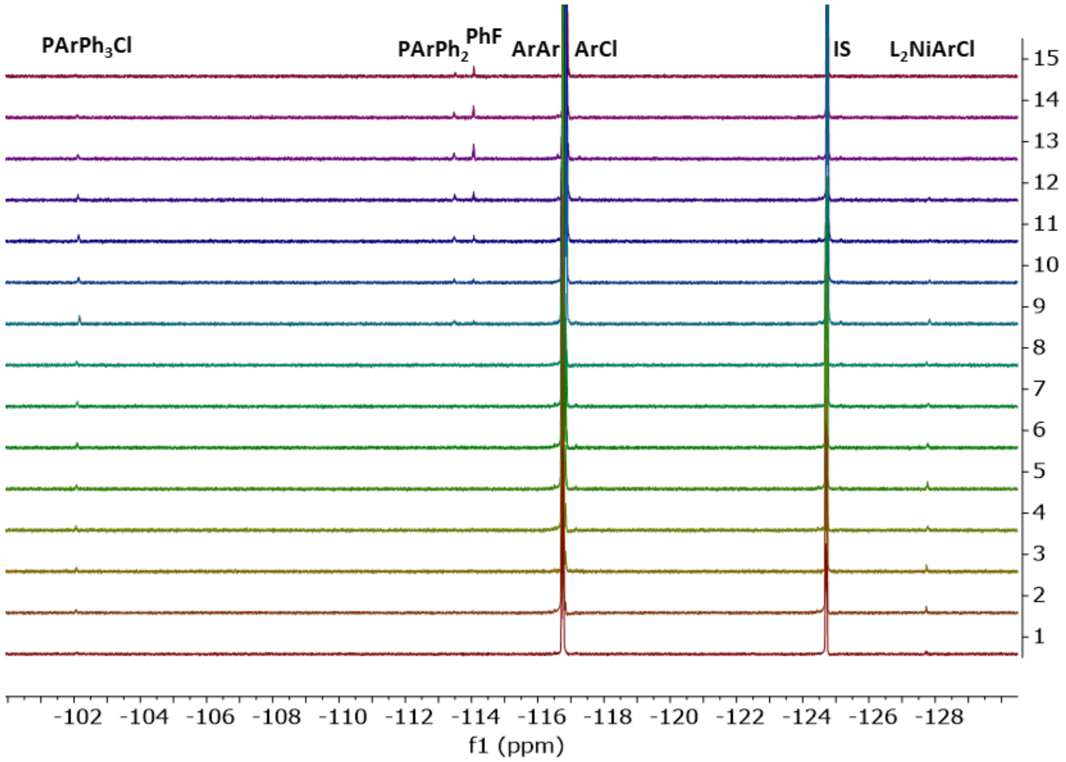

Figure S5: Ex situ ^{19}F NMR monitoring of the homocoupling under the standard conditions in DMF.

S4.1.3 Control Experiments

In this set of experiments the standard procedure was followed but in entries A, B, C, one of the reaction components was omitted. The concentrations are shown in Table S3.

Table S3: Concentrations and equivalents of reaction components for the control experiments.

All concentrations are in mM, Zn is in equivalents relative to ArCl (1)						
**Run**	**$[IS]_0$**	**$[1]_0$**	**$[Ni]_0$**	**$[PPh_3]_0$**	**Zn_0 / eq**	**Rate / $mM{\cdot}s^{-1}$**
A	464.8	633.9	28.4	471.9	0	0
B	464.8	633.9	0	480.1	1.49	0
C	464.8	633.9	30.6	0	1.50	0

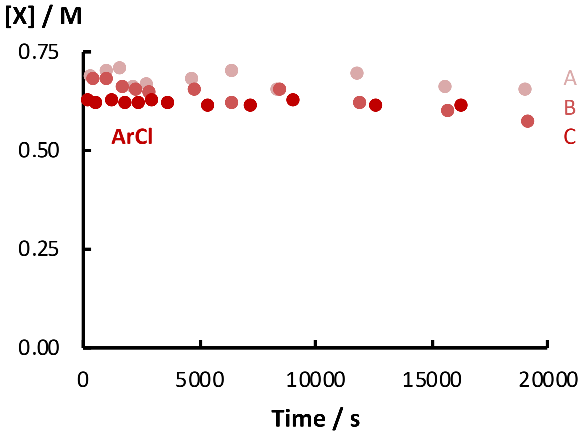

*Figure S6: Control experiments (runs A, B, C, Table S3). The concentration of ArCl (**1**) did not significantly change over a period of 5 hours.*

S4.1.4 Impact of Catalyst Concentration on the Rate of Homocoupling

For these experiments the standard procedure was followed, but the quantities of pre-catalyst, [$NiCl_2$(glyme)], varied between 6.6 mg, 29.9 µmol and 128 mg, 582 µmol, and PPh_3 varied between 502 mg, 1.92 mmol and 1.25 g, 4.79 mmol, see Table S4. The catalyst loading for runs K and L was too high and exceeds the solubility of [$Ni(PPh_3)_n(DMF)_m$] (*n* and *m* were not determined) and significant quantities precipitated resulting in unreliable stirring in run K and no stirring in run L (Figure S7a, see grey box). The rates in these runs are neither reliable nor reproducible and are excluded from the overall analysis, Figure S7d.

The rates of the reaction mixtures were calculated as described in the standard procedure and plotted against the Ni catalyst concentration, Figure S7 and Table S4.

Table S4: Concentrations and equivalents of all reaction components. The catalyst concentrations were varied in each run. The Ni concentration in runs K and L are nominal due to extensive precipitation, see discussion above, and the data not employed for analysis of rate versus [Ni]$_0$.

All concentrations are in mM, Zn amounts are in equivalents to ArCl (1)

Run	$[IS]_0$	$[1]_0$	$[Ni]_0$	$[PPh_3]_0$	Zn_0 / eq	Rate / mM·s^{-1}
A	464.8	633.9	7.5	499.9	1.46	7.67E-04
B	464.8	633.9	14.4	478.9	1.58	3.99E-03
C	464.8	633.9	16.7	469.2	1.54	6.86E-03
D	464.8	633.9	23.2	482.4	1.54	1.07E-02
E	464.8	633.9	32.3	479.8	1.54	1.89E-02
F	464.8	633.9	34.3	479.7	1.54	2.04E-02
G	464.8	633.9	38.9	487.5	1.51	2.66E-02
H	464.8	633.9	43.8	478.4	1.49	3.02E-02
I	464.8	633.9	50.3	549.4	1.50	4.04E-02
J	464.8	633.9	64.4	614.9	1.51	5.72E-02
K	464.8	633.9	110.4	957.4	1.50	7.20E-02
L	464.8	633.9	145.6	1197.0	1.50	4.55E-02

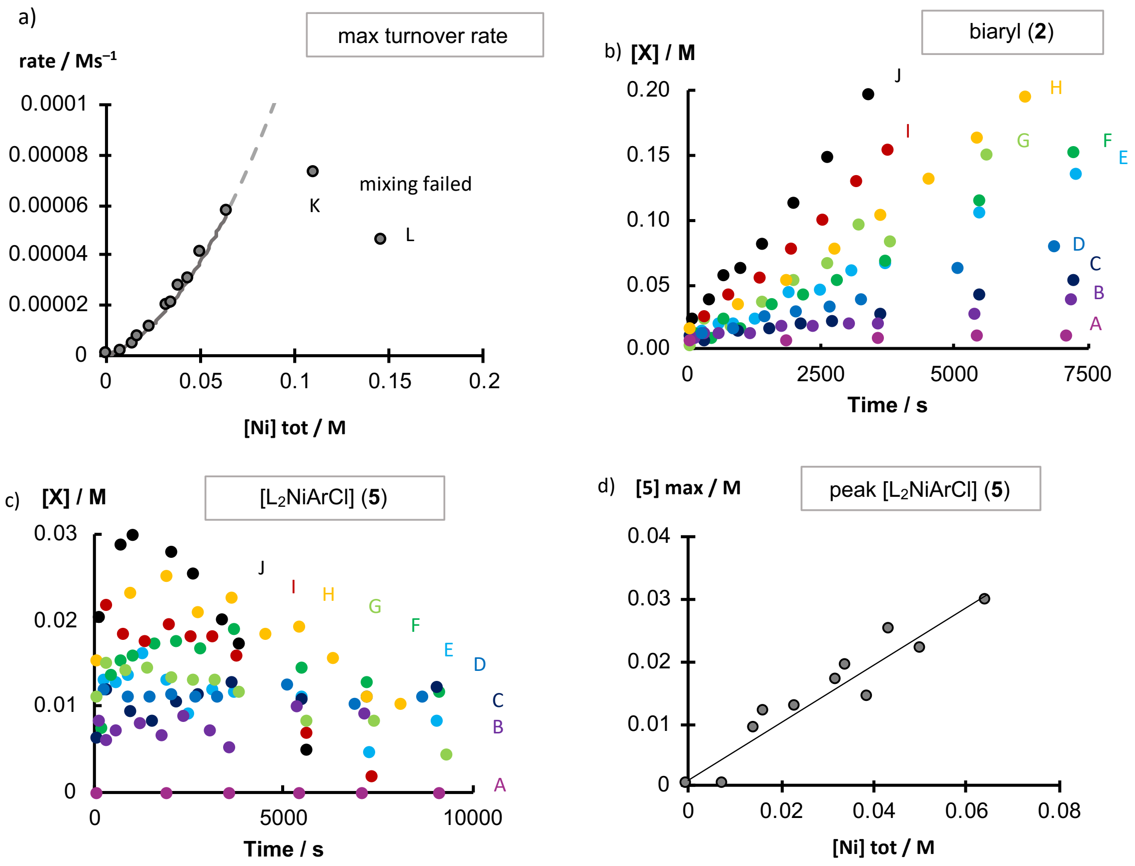

*Figure S7: a) Maximum turnover rate (d[**2**]/dt) plotted against the catalyst concentration. The dashed line indicates $\frac{d[2]}{dt} = k_C[Ni]_{tot}^1 + k_C'[Ni]_{tot}^2, where\ k_C \approx 3\times10^{-4}\ s^{-1}, k_C' \approx 1\times10^{-2}\ M^{-1}s^{-1}$. In runs K and L the activated catalyst precipitated and the reactions were not able to be stirred at sufficient rates - these two points are not included in the fitting. Reaction rates were calculated by linear regression analysis of ArAr (**2**), see (b). In graph (c) the approximate L_2NiArCl (**5**) concentrations, estimated from the sum of ArOH and [PPh_3Ar]$^+$ concentrations in ex situ samples, are plotted against time. d) Maximum concentration of Ar-Ni complex 5 (see graph c) against total Ni concentration.*

S4.1.5 Control experiment for Internal Standard (1-fluoronaphthalene)

For these experiments the standard procedure was followed, but the concentrations of 1-fluoronaphthalene (240 µL, 1.86 mmol and 300 µL, 2.32 mmol) were reduced, as shown in Table S5. There was no detectable impact on the rate, Figure S8.

Table S5: Concentrations and equivalents of reaction components, with variation in the internal standard from the standard procedure (Table S4).

All concentrations are in mM, Zn is in equivalents to ArCl (1)

Run	$[IS]_0$	$[1]_0$	$[Ni]_0$	$[PPh_3]_0$	Zn_0 / eq	Rate / mM·s^{-1}
A	290.5	633.9	32.1	481.2	1.54	2.01E-02
B	232.4	633.9	36.3	465.5	1.53	2.34E-02

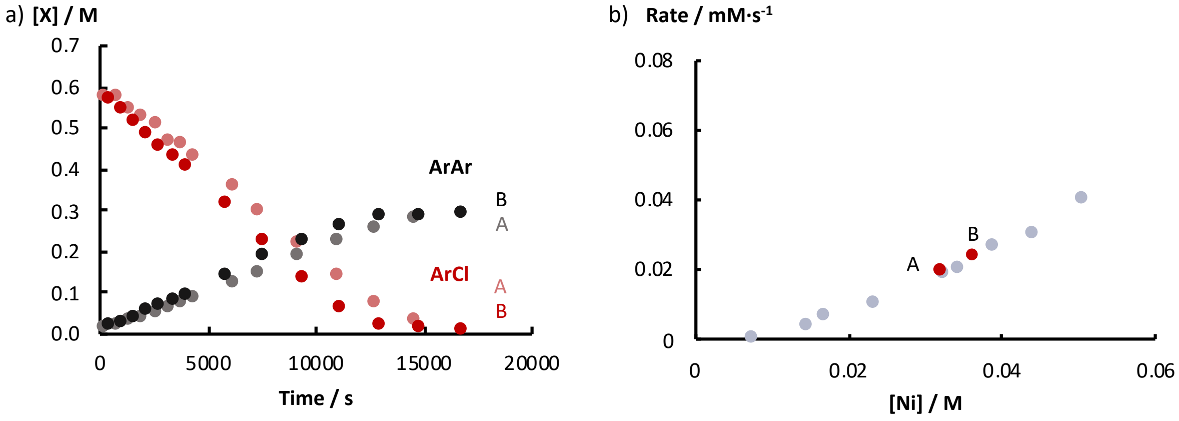

Figure S8: a) Concentration vs time plot of the homocoupling with lower internal standard concentrations. The mildly sigmoidal profiles the standard conditions are again observed. b) Rate vs catalyst loading plot, with runs A and B highlighted. The internal standard concentration has no significant influence on the reaction rate.

S4.1.6 Variation in Substrate Concentration

For these experiments the standard procedure was followed, but the concentrations of 1-chloro-4-fluorobenzene (**1**) (270 µL, 2.54 mmol and 450 µL, 4.23 mmol) were varied, as shown in Table S6.

*Table S6: Concentrations and equivalents of reaction components, with variation in the concentration of ArCl (**1**) from the standard conditions (Table S4).*

All concentrations are in mM, Zn amounts are in equivalents to ArCl (1)

Run	$[IS]_0$	$[1]_0$	$[Ni]_0$	$[PPh_3]_0$	Zn_0 / eq	Rate / $mM{\cdot}s^{-1}$
A	464.8	316.9	34.9	463.0	3.02	2.31E-02
B	464.8	528.2	35.9	481.7	1.86	2.57E-02

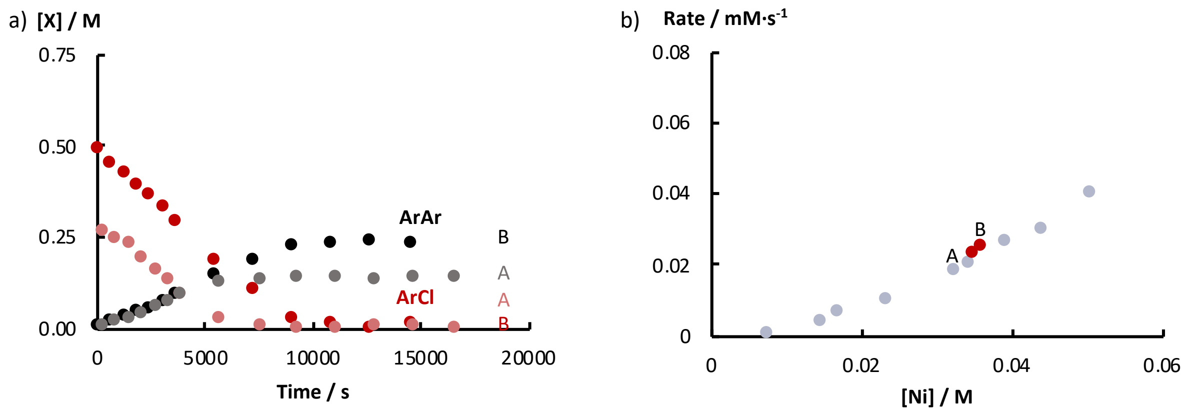

Figure S9: a) Concentration vs time plots of the Ni catalysed homocoupling at initial substrate concentrations of 0.32 M and 0.53 M, that vary from the standard conditions. b) Rate vs Ni concentration plot of the coupling under standard conditions and the couplings at reduced substrate concentrations: there is no significant influence on the maximum reaction rates attained in the central sections of the mildly sigmoidal product evolution profiles. This indicates that the main phase of the reaction has a pseudo-zero order kinetic dependency on the Ar-Cl substrate concentration.

S4.1.7 Variation of the Zinc Loading

For these experiments the standard procedure was followed, but the quantity of Zn powder (371 mg, 5.67 mmol and 506 mg, 7.73 mmol) was varied, as shown in Table S7, leading to changes in the surface area available for reduction of Ni-species.

Table S7: Concentrations and equivalents of reaction components, with variation in the equivalents of Zn powder employed.

All concentrations are in mM, Zn is in equivalents to ArCl (1)

Run	$[IS]_0$	$[1]_0$	$[Ni]_0$	$[PPh_3]_0$	Zn_0 / eq	Rate / $mM{\cdot}s^{-1}$
A	503.1	633.9	39.9	477.5	3.05	3.74E-02
B	464.8	633.9	38.1	479.8	2.24	3.83E-02

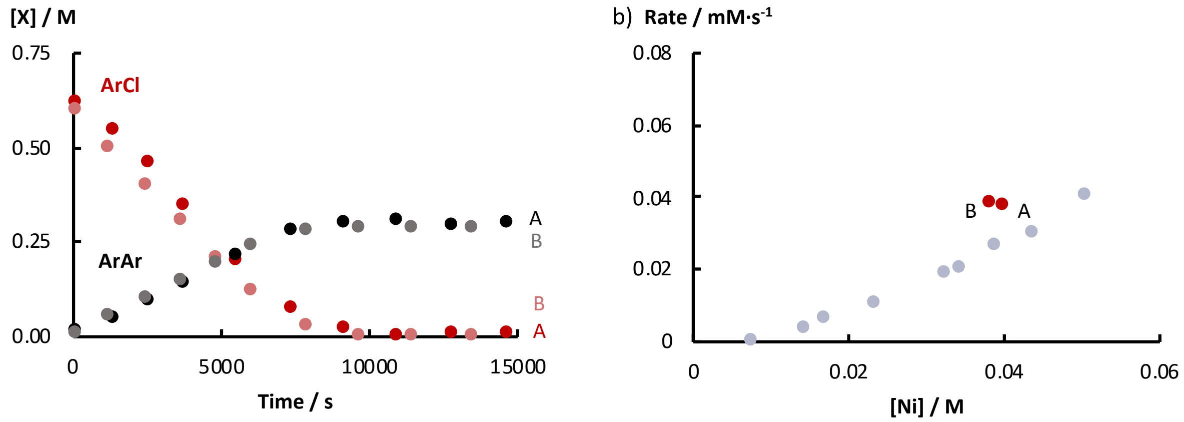

Figure S10: a) Concentration vs time plot of the homocoupling at various initial Zn loadings (2.24 – 3.05 eq.). b) Rate vs Ni concentration plot of the reaction under standard conditions coplotted with coupling at the two raised Zn loadings. The reaction rate increases, in direct proportion to the loading for run B, and less than proportionally in run A; it is noted however that the impact of stirring rate was not explored for these experiments.

S4.1.8 Reduction in the PPh_3 Concentration

For these experiments the standard procedure was followed, but the quantity of PPh_3 was reduced to between 209 mg, 795 µmol and 254 mg, 967 µmol.

For run B the reaction was initiated at a low phosphine concentration (209 mg, 795 µmol) and the reaction stalled. Further PPh_3 (326 mg, 1.24 mmol) was added after 3h 5 min, resulting in the phosphine concentration being raised to the value indicated in the [additive] column.

For run C the reaction was initiated at a low phosphine concentration (254 mg, 967 µmol) and after the reaction stalled, NaI (394 mg, 2.63 mmol) was added after 3h 45 min.

For run D the reaction was initiated at a low phosphine concentration (251 mg, 957 µmol) and after the reaction stalled, NaCl (161 mg, 2.75 mmol) was added after 4h 28 min.

The concentrations are shown in Table S8.

Table S8: Concentrations and equivalents of the reaction components, with the PPh_3 concentration reduced from that in the standard procedure, and additives introduced in runs B-D.

All concentrations are in mM, Zn is in equivalents to ArCl (1)

Run	$[IS]_0$	$[1]_0$	$[Ni]_0$	$[PPh_3]_0$	Zn_0 / eq	$[Additive]_t$	Additive	Rate / $mM{\cdot}s^{-1}$
A	464.8	633.9	30.3	241.1	1.49	-	-	1.57E-02
B	464.8	633.9	32.1	198.8	1.50	613.0[i]	PPh_3	1.68E-02
C	464.8	633.9	28.3	241.7	1.52	905.5[ii]	NaI	1.35E-02
D	464.8	633.9	38.2	239.1	1.47	915.5[iii]	NaCl	2.68E-02

i. PPh_3 was added to the reaction mixture after 11100 s (3h 5 min). $[Additive]_t$ was calculated from the amount of PPh_3 which was added while the reaction was set up and the amount added during the reaction. The volume reduction due to sampling is considered in this calculation. Each sample volume is approximately 100 µL.

ii. NaI was added to the reaction mixture after 13500 s (4 h 45 min). $[Additive]_t$ was calculated from the amount of NaI which was added during the reaction. The volume reduction due to sampling is considered in this calculation. Each sample volume is approximately 100 µL.

iii. NaCl was added to the reaction mixture after 16060 s (4 h 28 min). $[Additive]_t$ was calculated from the amount of NaCl which was added during the reaction. The volume reduction due to sampling is considered in this calculation. Each sample volume is approximately 100 µL.

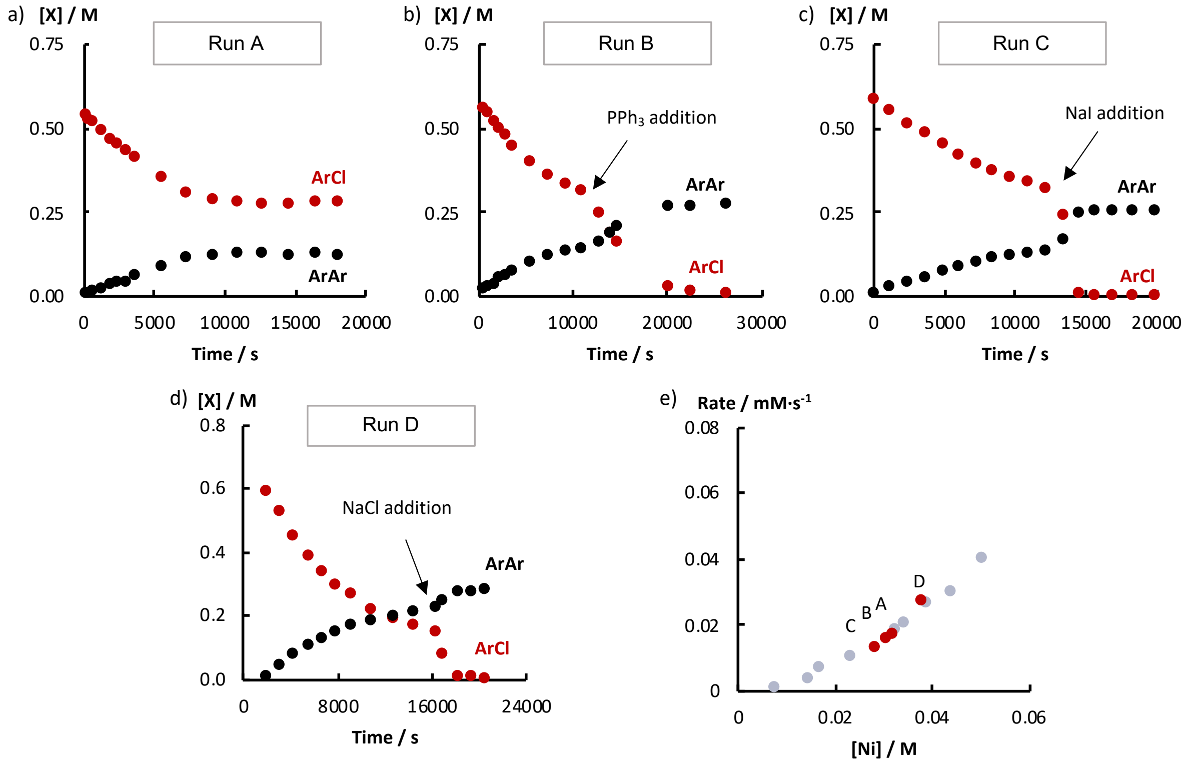

Figure S11: a) Concentration vs time plot with the phosphine concentration at approximately half that of the standard conditions. The reaction stalls at around 40 % conversion. b) Same as (a) but reactivated by addition of phosphine at the time indicated. c) Same as (a) but reactivated by addition of NaI at the time indicated. d) Same as (a) but reactivated by addition of NaCl at the time indicated. e) Initial rates of the reactions plotted against the Ni catalyst loading prior to stalling and additives. The two-fold reduction in phosphine concentration has no significant effect on the initial rate.

S4.1.9 Homocoupling with Endogenous $ZnCl_2$

For these experiments the standard procedure was followed but $ZnCl_2$ (between 155 mg, 1.13 mmol and 267 mg, 1.96 mmol) was added to the reaction mixture.

For run A, the $ZnCl_2$ (267 mg, 1.96 mmol) was present from the start.

For run B, the $ZnCl_2$ (263 mg, 1.93 mmol) was added after 1 hour and 37 minutes.

Run C was initiated with a lower initial quantity of PPh_3 than the standard procedure (254 mg, 967 µmol), and the $ZnCl_2$ (155 mg, 1.13 mmol) added after 57 minutes. Further PPh_3 (517 mg, 1.97 mmol) was introduced after 2 hours 27 minutes to give the total phosphine concentration indicated as $[PPh_3]_{TOT}$ in Table S9.

The concentrations are shown in Table S9.

Table S9: Concentrations and equivalents of all reaction components. $ZnCl_2$ and PPh_3 were added during the reaction.

All concentrations are in mM, Zn is in equivalents to ArCl (1)								
**Run**	**$[IS]_0$**	**$[1]_0$**	**$[Ni]_0$**	**$[PPh_3]_0$**	**Zn_0 / eq**	**$[ZnCl_2]_{TOT}$**	**$[PPh_3]_{TOT}$**	**Rate / mM·s^{-1}**
A	464.8	633.9	35.3	479.5	1.50	488.8	-	3.47E-04
B	464.8	633.9	32.8	478.9	1.52	739.2[i]	-	1.59E-02
C	464.8	633.9	33.4	241.7	1.49	437.1[ii]	946.3[ii]	1.72E-02

i. $ZnCl_2$ was added to the reaction mixture after 5778 s (1 h 36 min 18 s). $[ZnCl_2]_{TOT}$ is calculated from the quantity of $ZnCl_2$ initially added to the reaction mixture, plus the $ZnCl_2$ produced during the reaction up to the last data point before the addition of exogenous $ZnCl_2$, and included the $ZnCl_2$ produced from Ni^{II} pre-catalyst reduction. The volume reduction due to sampling is also considered in these calculations. Each sample volume is approximately 100 µL.

ii. $ZnCl_2$ was added to the reaction mixture after 3450 s (57 min 30 s) and PPh_3 was added to the reaction mixture after 8790 s (2 h 26 min 30 s). $[ZnCl_2]_{TOT}$ is calculated from the quantity of $ZnCl_2$ initially added to the reaction mixture, plus the $ZnCl_2$ produced during the reaction up to the last data point before the addition of exogenous $ZnCl_2$, and included the $ZnCl_2$ produced from Ni^{II} pre-catalyst reduction. $[PPh_3]_{TOT}$ was calculated from the quantity of PPh_3 added initially, plus that subsequently added to the reaction. The volume reduction due to sampling is considered in both calculations. Each sample volume is approximately 100 µL.

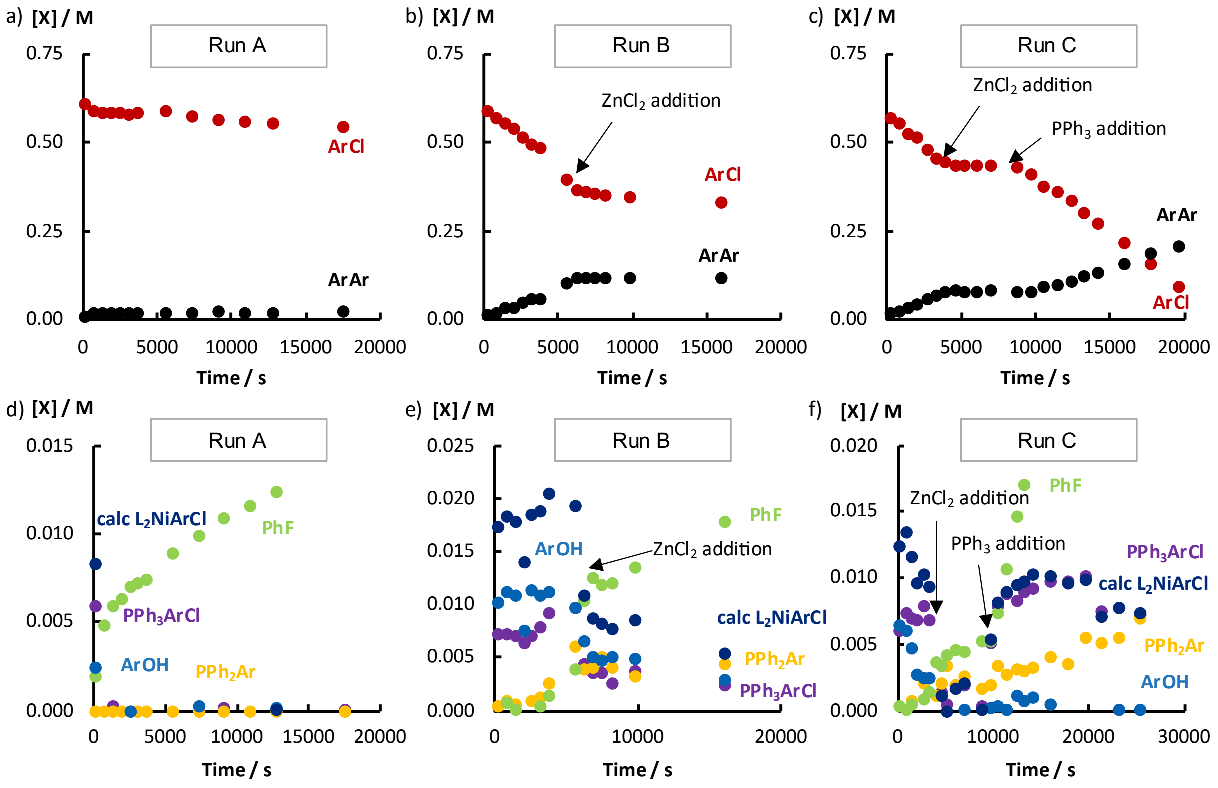

*Figure S12: a) Concentration vs time plot of the homocoupling in which $ZnCl_2$ was added at the beginning of the reaction. The reaction stalls after approximately one turnover. b) Concentration vs time plot of the homocoupling in which the $ZnCl_2$ was added after 1 h 36 min. The reaction stalls immediately after the addition. c) Concentration vs time plot in which $ZnCl_2$ was added after 57 minutes. The reaction stalls immediately after the addition. After 2h 26 minutes further phosphine was added which reactivates the reaction. d) Concentration vs time plot of the side products and intermediates of run A. $L_2NiArCl$ (**5**) formation was only detected (indirectly from the ex situ sampling quenching products) in the first data point. e) Concentration vs time plot of the side products and intermediates of run B. The estimated $L_2NiArCl$ (**5**) concentration decreases significantly after the addition of the $ZnCl_2$. f) Concentration vs time plot of the intermediates and side products. The estimated $L_2NiArCl$ (**5**) concentration decreases after $ZnCl_2$ addition and is restored on addition of further phosphine.*

S4.1.10 Homocoupling using $NiCl_2$ as Pre-catalyst.

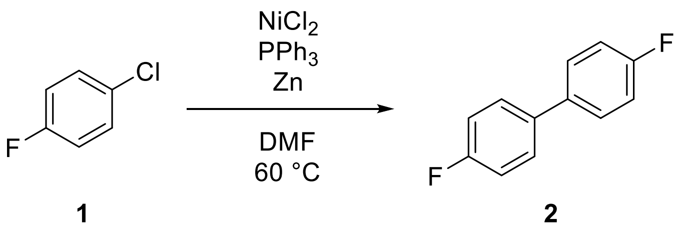

Scheme S8: Reaction scheme of the Ni catalysed Ullmann coupling using $NiCl_2$ as a Ni source.

The experiment was set up as described in the standard procedure, but $NiCl_2$ (20.0 mg, 154 µmol) employed instead of [$NiCl_2$(glyme)]. The concentrations are shown in Table S10.

Table S10: Concentrations and equivalents of all reaction components. $NiCl_2$ was used as Ni source.

All concentrations are in mM, Zn is in equivalents to ArCl (1)

Run	$[IS]_0$	$[1]_0$	$[Ni]_0$	$[PPh_3]_0$	Zn_0 / eq	Rate / mM·s^{-1}
A	464.8	633.9	38.5	486.4	1.20	2.29E-02

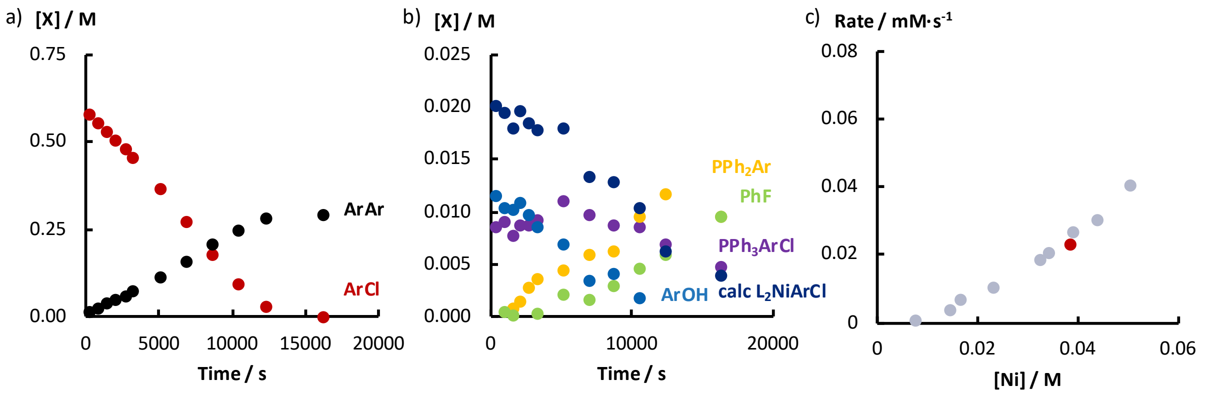

*Figure S13: a) Concentration vs time plot of the Ni catalysed homocoupling using $NiCl_2$ as pre-catalyst, with the usual mildly sigmoidal profile. b) Concentration vs time plot of the side products and intermediates, and estimated concentration of $L_2NiArCl$ (**5**). c) Reaction rate vs the catalyst loading, co-plotted against data obtained using [$NiCl_2$(glyme)], indicative that there is no significant impact from the glyme that is released on pre-catalyst reduction.*

S4.1.11 Homocoupling Initiated using other Nickel Pre-Catalysts

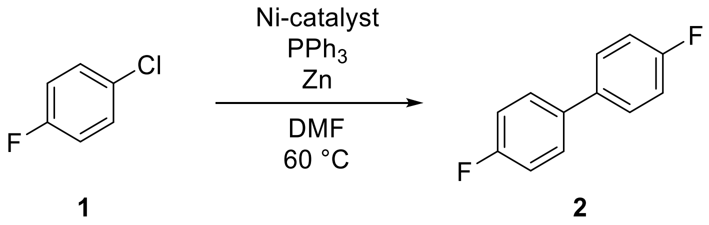

Scheme S9: Reaction scheme of the Ni catalysed Ullmann coupling with various Ni catalysts.

For these experiments the standard procedure was followed, but the Ni source was varied.

In runs A and E, the $L_2NiArCl$ complex **5** (93.6 mg, 131 µmol) was employed instead of [$NiCl_2$(glyme)].

In runs B-D, $Ni(COD)_2$ (41.8 mg, 152 µmol) was employed instead of [$NiCl_2$(glyme)].

In run B, the $Ni(COD)_2$ and PPh_3 were dissolved in 2 mL DMF (instead of 3 mL) and 2 mL of the ArCl (**1**) stock solution were added after the activation period.

In run C NaCl (30.2 mg, 516 µmol) was added at the same time as $Ni(COD)_2$ (41.4 mg, 151 µmol), phosphine and Zn powder.

In run D and E, $ZnCl_2$ (31.9 mg, 234 µmol in run D and 49.0 mg, 359.5 µmol in run E) was added at the same time as the nickel catalyst, phosphine and Zn powder.

The concentrations are shown in Table S11.

Table S11: Concentrations and equivalents of the reaction components, with variation in the Ni source employed.

All concentrations are in mM, Zn is in equivalents to ArCl (1)

Run	$[IS]_0$	$[1]_0$	$[Ni]_0$	Ni source	$[PPh_3]_0$	Zn_0 / eq	[Additive]	Additive
A	464.8	633.9	32.8	**5**	478.0	1.49	0.0	-
B	464.8	633.9	37.1	$Ni(COD)_2$	474.2	1.56	0.0	-
C	464.8	633.9	37.6	$Ni(COD)_2$	483.2	1.55	129.0	NaCl
D	464.8	633.9	35.8	$Ni(COD)_2$	481.8	1.55	58.5	$ZnCl_2$
E	464.8	633.9	34.8	**5**	482.7	1.48	89.9	$ZnCl_2$

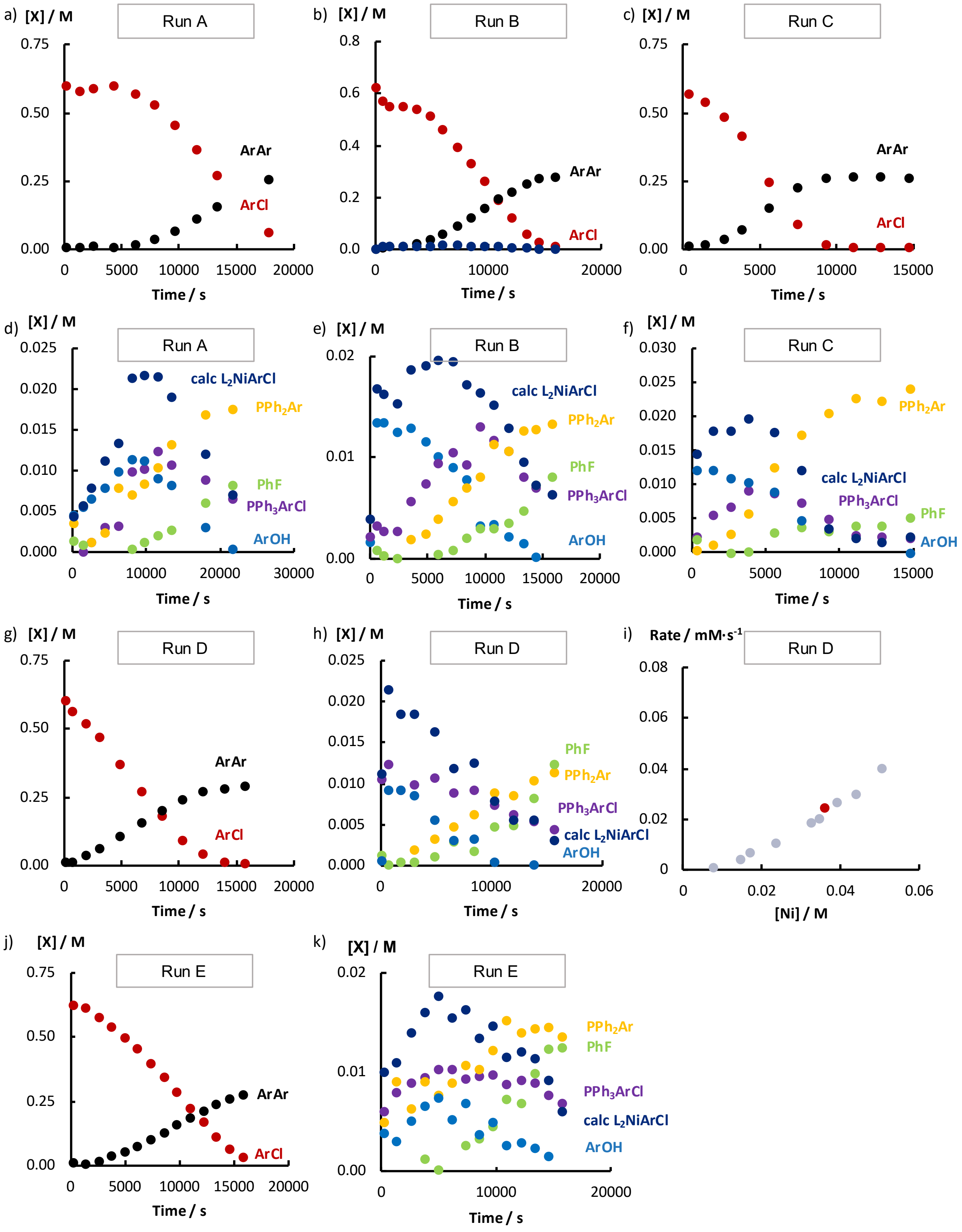

*Figure S14: a) Homocoupling using $L_2NiArCl$ (**5**) as a catalyst. b) Homocoupling using $Ni(COD)_2$ as a catalyst. In both (a) and (b) the reactions have a significant activation period. c) Homocoupling using $Ni(COD)_2$ as a catalyst with NaCl as an additive. The induction period was reduced and the reaction profile changes. d), e) and f) Concentration vs time plot of the side products and intermediates of runs A to C. During the activation periods in A and B, the $L_2NiArCl$ (**5**) accumulates. In run C, the reaction profile of the side products and intermediates is similar to that under standard conditions. g) Homocoupling using $Ni(COD)_2$ in the presence of $ZnCl_2$, with no notable catalyst activation period. h) Side products and intermediates in run D, again with no significant difference to the standard conditions. i) The reaction rate of run D co-plotted against the Ni catalyst concentration. j) Homocoupling using $L_2NiArCl$ (**5**) in the presence of $ZnCl_2$. k) Concentrations vs time plot of side products and intermediates in run E. It is noted that there is an initial delay while the $L_2NiArCl$ (**5**) dissolves in the DMF at 60 °C.*

S4.1.12 Homocoupling with tri(para-fluorophenyl)phosphine (PAr_3)

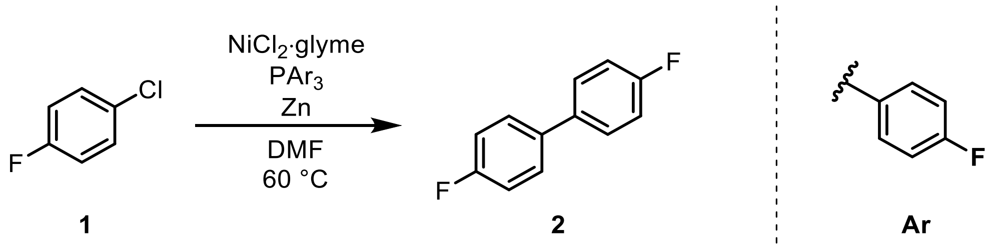

Scheme S10: Reaction scheme of the Ni catalysed Ullmann coupling with PAr_3 as a phosphine ligand.

For these experiments the standard procedure was followed, but tri(para-fluorophenyl)phosphine (PAr_3, 612 mg, 1.94 mmol) was used instead of PPh_3. The concentrations are shown in Table S12.

S4.1.12.1 Calculations and Plots

Due to the change in ligand, the Ar-ligand scrambling with the phosphine is degenerate. Phosphine oxidation ($OPAr_3$) was identified by spiking an indepently prepared sample into a reaction sample.

Table S12: Concentrations and equivalents of the reaction components. PAr_3 was used instead of PPh_3.

All concentrations are in mM, Zn is in equivalents to ArCl (1)

Run	$[IS]_0$	$[1]_0$	$[Ni]_0$	$[PAr_3]_0$	Zn_0 / eq	Rate / mM·s^{-1}
A	464.8	633.9	28.5	484.0	1.51	1.76E-02

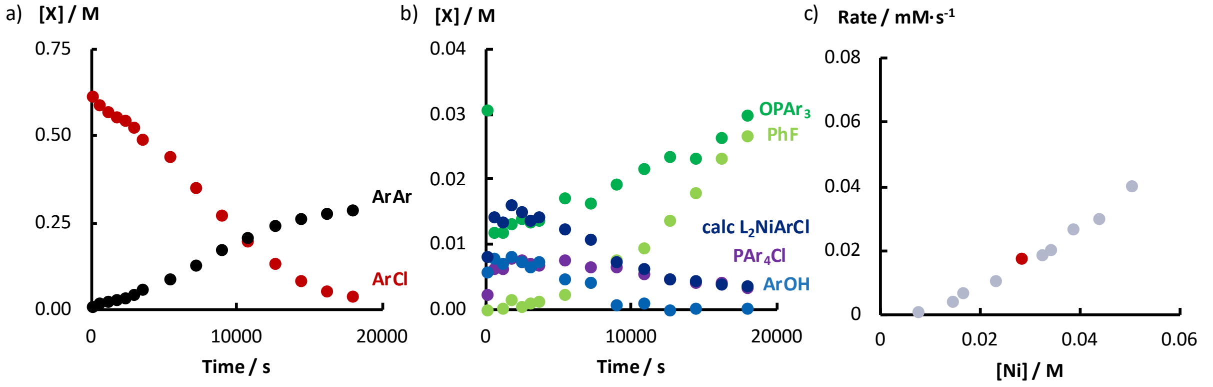

*Figure S15: a) Concentration vs time plot of the homocoupling using PAr_3 as a ligand. The reaction profile is similar to the standard conditions. b) Concentration vs time plot of the side products and intermediates. Phosphine oxide is produced during the quenching process. Control experiments indicate it arises from $Ni(PAr_3)_n$. $L_2NiArCl$ (**6**) was estimated from ArOH and $[PAr_4][Cl]$ in quenched samples. c) Rate of the reaction using PAr_3, normalised by catalyst loading, and showing it to be similar to the rate of the standard reaction using PPh_3 as a ligand.*

S4.1.13 Effect of Reduction in Overall Concentration on the Homocoupling

For this experiment the standard procedure was followed but the reaction diluted by a factor of approximately two. The stock solution was made with ArCl (**1**, 270 µL, 2.54 mmol) and 1-fluoronaphthalene (240 µL, 1.86 mmol), and the quantities of [$NiCl_2$(glyme)] (22.1 mg, 100 µmol) and PPh_3 (250 mg, 953 µmol) reduced. The quantity of Zn powder (242 mg, 3.70 mmol) was kept similar to the standard procedure to minimise the impact of mixing. The concentrations are in Table S13.

Table S13: Concentrations and equivalents of reaction components.

All concentrations are in mM, Zn is in equivalents to ArCl (1)

Run	$[IS]_0$	$[1]_0$	$[Ni]_0$	$[PPh_3]_0$	Zn_0 / eq	Rate / mM·s^{-1}
A	232.4	316.9	25.1	238.2	2.92	1.62E-02

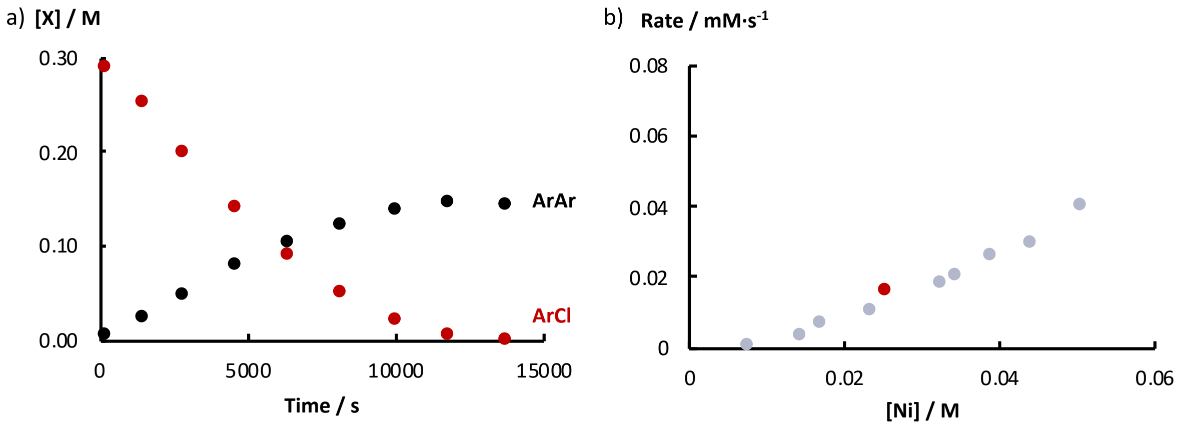

Figure S16: a) Concentration vs time plot of the homocoupling at two-fold dilution. The reaction profile is similar to that of the using standard conditions. b) Reaction rate plotted against the catalyst loading to allow comparison with the standard conditions.

S4.1.14 Homocoupling at 80 °C

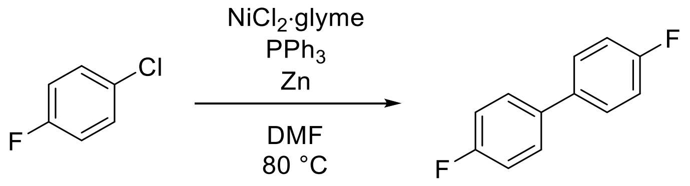

1 **2**

Scheme S11: Ni catalysed homocoupling at 80 °C.

The experiments were set up as described in the standard procedure, but the reaction at 80 °C instead of 60 °C. The concentrations are shown in Table S14.

Table S14: Concentrations and equivalents of reaction components. The reaction was run at 80 °C.

All concentrations are in mM, Zn is in equivalents to ArCl (1)						
**Run**	**$[IS]_0$**	**$[1]_0$**	**$[Ni]_0$**	**$[PPh_3]_0$**	**Zn_0 / eq**	**Rate / mM·s^{-1}**
A	464.8	633.9	29.9	488.0	1.49	7.93E-02

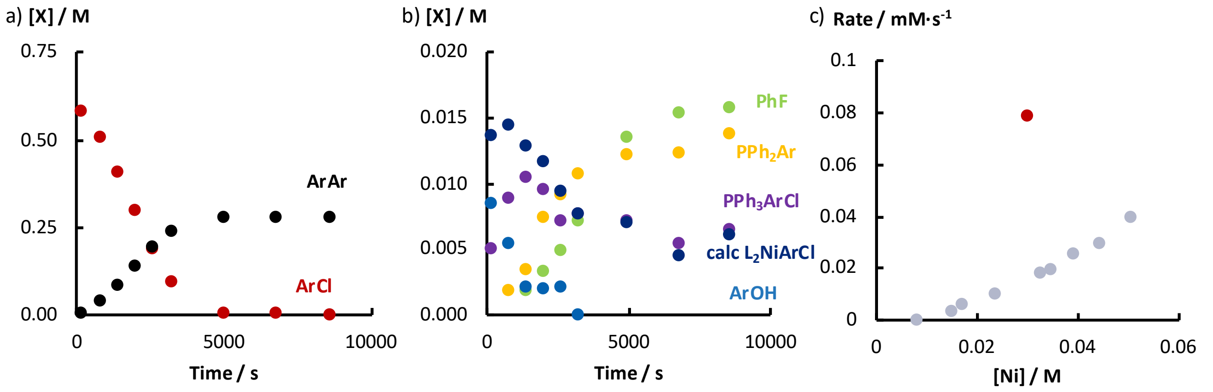

Figure S17: a) Concentration vs time plot of the homocoupling at 80 °C. The reaction profile is similar to the standard conditions. b) Concentration vs time plot of the side reactions and intermediates. The kinetic profile is similar to the standard conditions. c) Reaction rate at 80 °C plotted against the catalyst loading and compared to the reaction rate under standard conditions at 60 °C. The rate is significantly greater at 80 °C compared to 60 °C.

S4.1.15 Homocoupling without Pre-activation

For runs A and B the standard procedure was followed but without the 30 minutes activation period. All reagents and the stock solution were added at the start of the reaction.

For run B $ZnCl_2$ (20.3 mg, 149 µmol) was added at the same time as the Ni catalyst, phosphine (PPh_3) and Zn powder.

For run C the standard procedure was followed with the usual activation period, but and additional 1 ml of a [$NiCl_2$(glyme)] stock solution was added after approximately 45 minutes.

S4.1.15.1 [$NiCl_2$(glyme)] stock solution

A stock solution of $NiCl_2$·glyme (37.42 mg, 170 µmol) was made in a volumetric flask in DMF (2 mL).

The concentrations are in Table S15.

Table S15: Concentrations and equivalents of reaction components.

All concentrations are in mM, Zn is in equivalents to ArCl (1)

Run	$[IS]_0$	$[1]_0$	$[Ni]_0$	$[PPh_3]_0$	Zn_0 / eq	$[ZnCl_2]_0$	$[Ni]_{TOT}$	Rate / $mM{\cdot}s^{-1}$
A	464.8	633.9	39.1	478.0	1.56	0.0	-	2.18E-02
B	464.8	633.9	40.1	478.8	1.52	37.3	-	2.35E-02
C	464.8	633.9	32.1	481.7	1.51	0.0	53.4[i]	1.53E-02 (before addition)<br>4.20E-02 (after addition)

i. [$NiCl_2$(glyme)] was added to the reaction mixture after around 2700 s (45 minutes). $[Ni]_0$ was calculated from the quantity of [$NiCl_2$(glyme)] added before the start of the reaction and the amount added during the reaction. The volume reduction due to sampling is considered in this calculation. Each sample volume is approximately 100 µL.

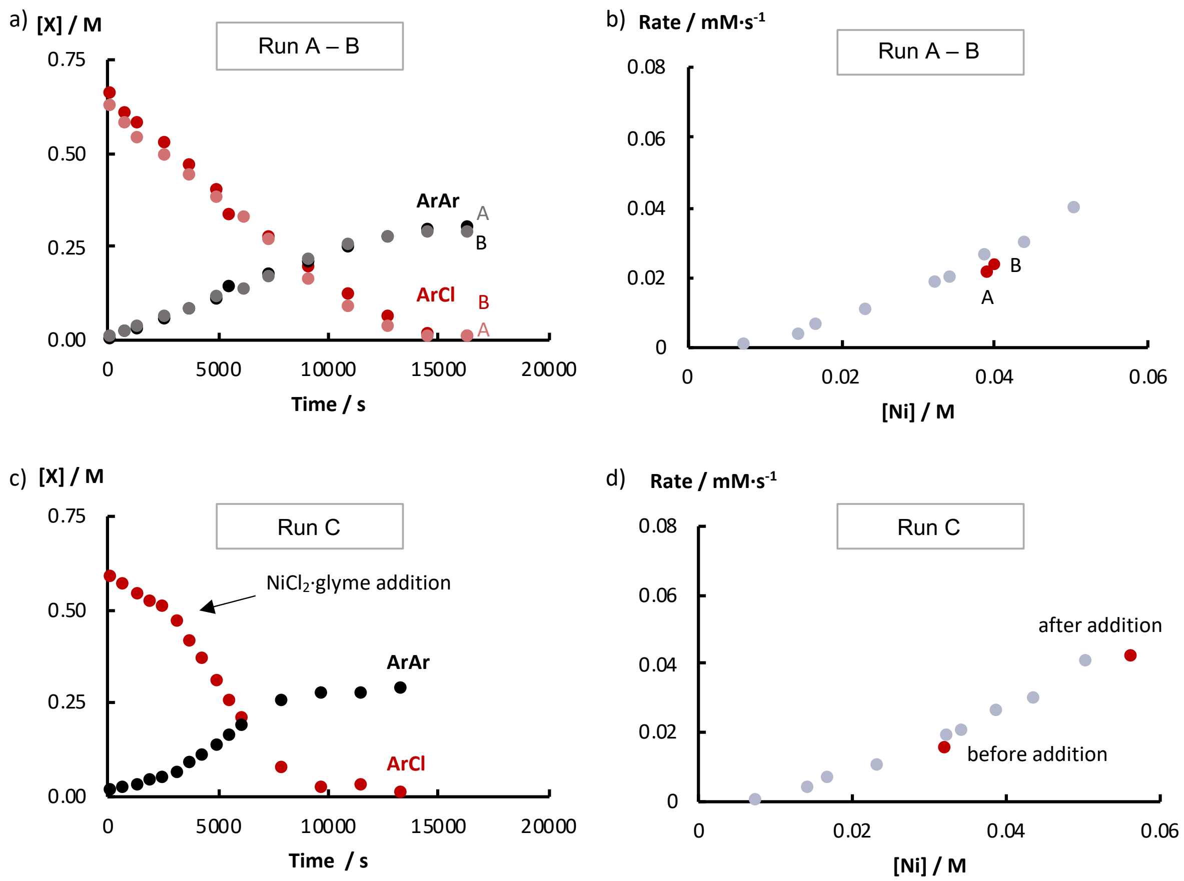

Figure S18: a) Concentration vs time plot of homocouplings without activation period. In run B, $ZnCl_2$ was added at the beginning of the reaction as an additive. Neither profile is significantly different to the standard conditions. b) Reaction rates of the two reactions without activation period plotted against the Ni catalyst loading. The rates are similar to the standard conditions. c) Concentration vs time plot of a reaction in which additional [$NiCl_2$(glyme)] is added during the reaction. The reaction rate increases after the addition. d) Rate of the run C before and after the addition of [$NiCl_2$(glyme)].

S4.1.16 Addition of Further Substrate after Completion of the Homocoupling

The standard procedure was followed but the stock solution of ArCl (**1**, 1.35 mL, 12.7 mmol) and internal standard (1.20 mL, 9.30 mmol) was made on a larger scale (5 mL). Further ArCl (**1**) stock solution (1 ml) was added to the reaction after 5h 3 min. The concentrations are shown in Table S16.

Table S16: Concentrations and equivalents of reaction components.

All concentrations are in mM, Zn amounts are in equivalents to ArCl (1)						
**Run**	**$[IS]_0$**	**$[1]_0$**	**$[Ni]_0$**	**$[PPh_3]_0$**	**Zn_0 / eq**	**$[1]_{TOT}$**
A	464.8	633.9	33.4	493.8	1.45	652.4[i]

i. 1 mL of ArCl (**1**) stock solution (**1** and IS) was added after 18180 s (5h 3 min). $[\mathbf{1}]_{TOT}$ was calculated from the amount added at the start of the reaction, the amount added during the reaction and the amount that reacted during the reaction. The volume reduction due to sampling is considered in this calculation. Each sample volume is assumed to be 100 µL.

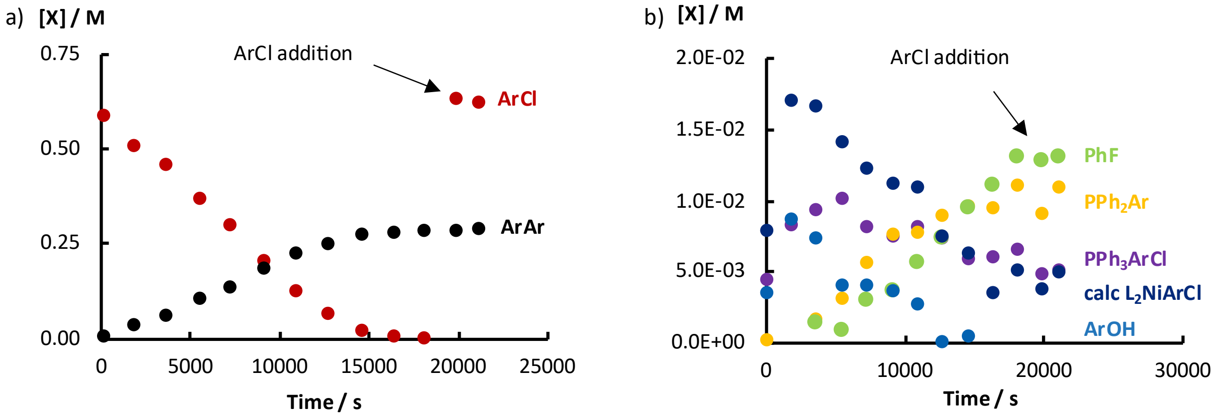

*Figure S19: a) Concentration vs time plot of the coupling in which further ArCl (**1**) is added after the reaction reached completion. No significant turnover was induced after addition of the second equivalent of ArCl (**1**). b) Concentration vs time plot of the side products and intermediates of the reaction. The concentration of $L_2NiArCl$ (**5**) does not increase after the addition of ArCl (**1**).*

S4.1.17 Homocoupling of 1-chloro-3-fluorobenzene

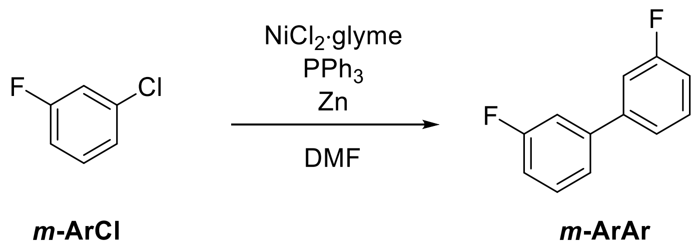

Scheme S12: Reaction Scheme of the coupling using the 1-chloro-3-fluorobenzene.

The experiment was performed using the standard procedure, but with the *meta* substrate (1-chloro-3-fluorobenzene, *m*-ArCl) (540 µL, 5.04 mmol). The concentrations are shown in Table S17.

Table S17: Concentrations and equivalents of reaction components.

All concentrations are in mM, Zn amounts are in equivalents to ArCl (1)						
Run	$[IS]_0$	[*m*-ArCl]$_0$	$[Ni]_0$	$[PPh_3]_0$	Zn_0 / eq	Rate / mM·s^{-1}
A	464.8	630.3	37.0	478.9	1.52	1.70E-02

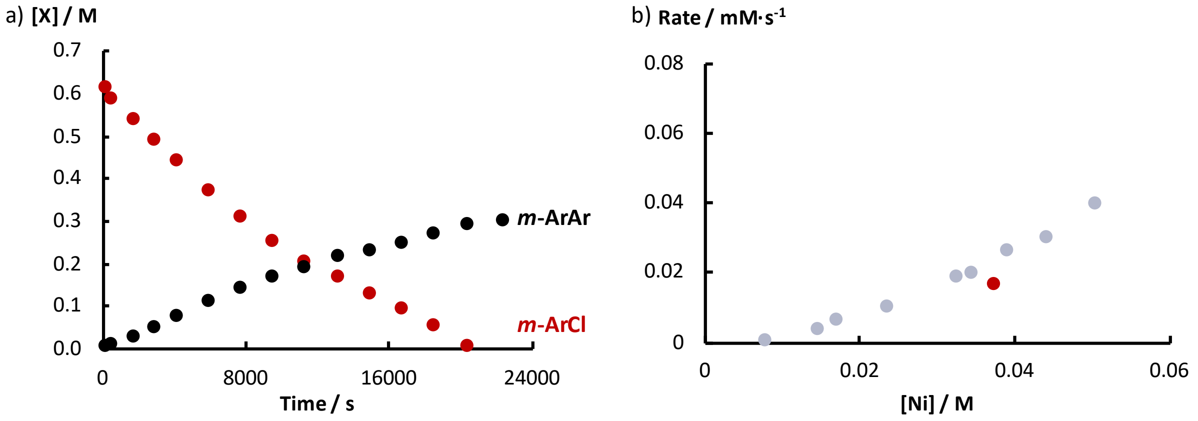

Figure S20: a) Concentration vs time plot of the homocoupling of the meta-fluoro analogue. The reaction profile shows a slight curvature and possibly a different rate law. b) The maximum reaction rate of the meta substrate is slightly lower than the one of the para substrate under the standard conditions.

S4.1.18 Homocoupling with Deliberate Exposure to Air

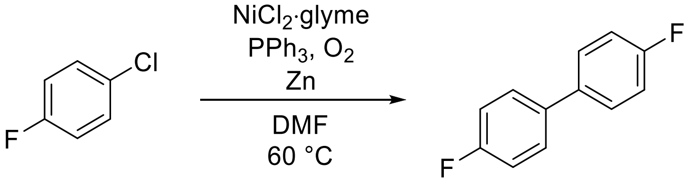

Scheme S13: Reaction scheme of the Ni catalysed Ullmann coupling with deliberate oxygen exposure.

The experiments were set up as described for the standard procedure.

For run A, the Schlenk flask and volumetric flask were opened to air to allow brief exposure to O_2, CO_2, etc. The reaction flask was closed again but not connected to the Schlenk line.

For run B, the DMF was transferred out of the glovebox and air was purged through the solution for 10-30 minutes and the solvent was dried over molecular sieves. The reaction flask and stock solution were prepared in the glovebox and the solvent was added outside of the glovebox without using Schlenk techniques.

The concentrations are shown in Table S18.

Table S18: Concentrations and equivalents of all reaction components. The reactions were deliberately exposed to air.

All concentrations are in mM, Zn is in equivalents to ArCl (1)

Run	$[IS]_0$	$[1]_0$	$[Ni]_0$	$[PPh_3]_0$	Zn_0 / eq	air exposure	Rate / $mM{\cdot}s^{-1}$
A	464.8	633.9	33.9	478.9	1.54	atmosphere	2.01E-02
B	464.8	633.9	33.1	454.9	1.51	DMF pre-exposed	1.64E-02

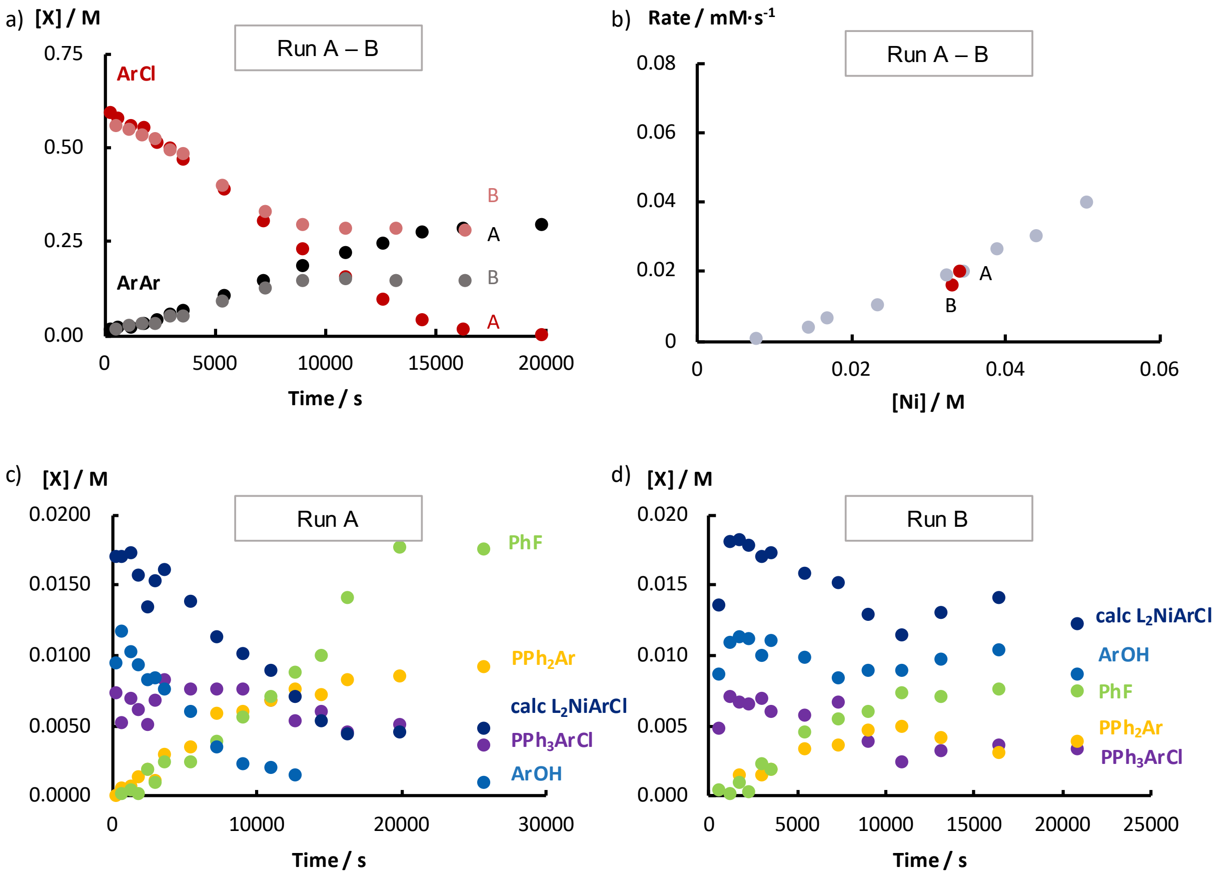

*Figure S21: a) Concentration vs time plot of two homocouplings with deliberate exposure to air. The reaction in which the atmosphere was briefly exposed to air (run A) showed a similar profile to the reaction without exposure. The reaction in which there was initial air exposure in solution started to stall after around 50 % conversion. b) The reaction rates of the reactions plotted against catalyst loading. The rate of run B was calculated from the data points prior to stalling. There was no significant rate difference in comparison to standard conditions. c) Concentration vs time plot of the intermediates and side products of run A. d) Concentration vs time plot of the intermediates and side products of run B. After the reaction stalls the concentration of PPh_2Ar and PhF (**3**) remain constant. The estimated concentration of $L_2NiArCl$ (**5**) is based on ex situ quenched samples which generate ArOH and $[PPh_3Ar]^+$ - these species may also be present in the reaction mixture at the point of air-exposure and stalling; i.e. the real $L_2NiArCl$ (**5**) concentration may be much lower.*

S4.1.19 Homocoupling in the Presence of H_2O and D_2O

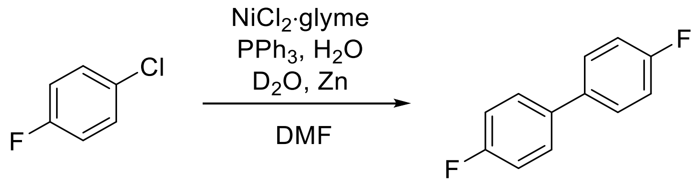

1 **2**

Scheme S14: Reaction Scheme of the Ni catalysed Ullmann coupling in the presence of H_2O and D_2O.

The experiments were performed according to the standard procedure, but before the addition of the ArCl (**1**) stock solution, small volumes of H_2O (between 4.5 µL, 249 µmol and 70 µL, 3.87 mmol) and D_2O (between 44 µL, 2.43 mmol and 70 µL, 3.87 mmol) were added with a micro syringe. The concentrations are shown in Table S19.

Table S19: Concentrations and equivalents of reaction components. H_2O und D_2O were added in various amounts to the reaction mixtures.

All concentrations are in mM, Zn is in equivalents to ArCl								
**Run**	**$[IS]_0$**	**$[1]_0$**	**$[Ni]_0$**	**$[PPh_3]_0$**	**Zn_0 / eq**	**$[H_2O]_0$**	**$[D_2O]_0$**	**Rate / $mM \cdot s^{-1}$**
A	464.8	633.9	35.2	476.9	1.53	62.3	0	2.44E-02
B	464.8	633.9	33.1	489.5	1.50	332.1	0	1.49E-02
C	464.8	633.9	35.1	481.9	1.49	968.5	0	6.40E-03
D	464.8	633.9	32.8	480.4	1.54	0	622.1	1.92E-02
E	464.8	633.9	29.7	478.1	1.50	0	967.3	1.50E-02
F	464.8	633.9	34.3	480.9	1.53	608.0	608.8	1.12E-02

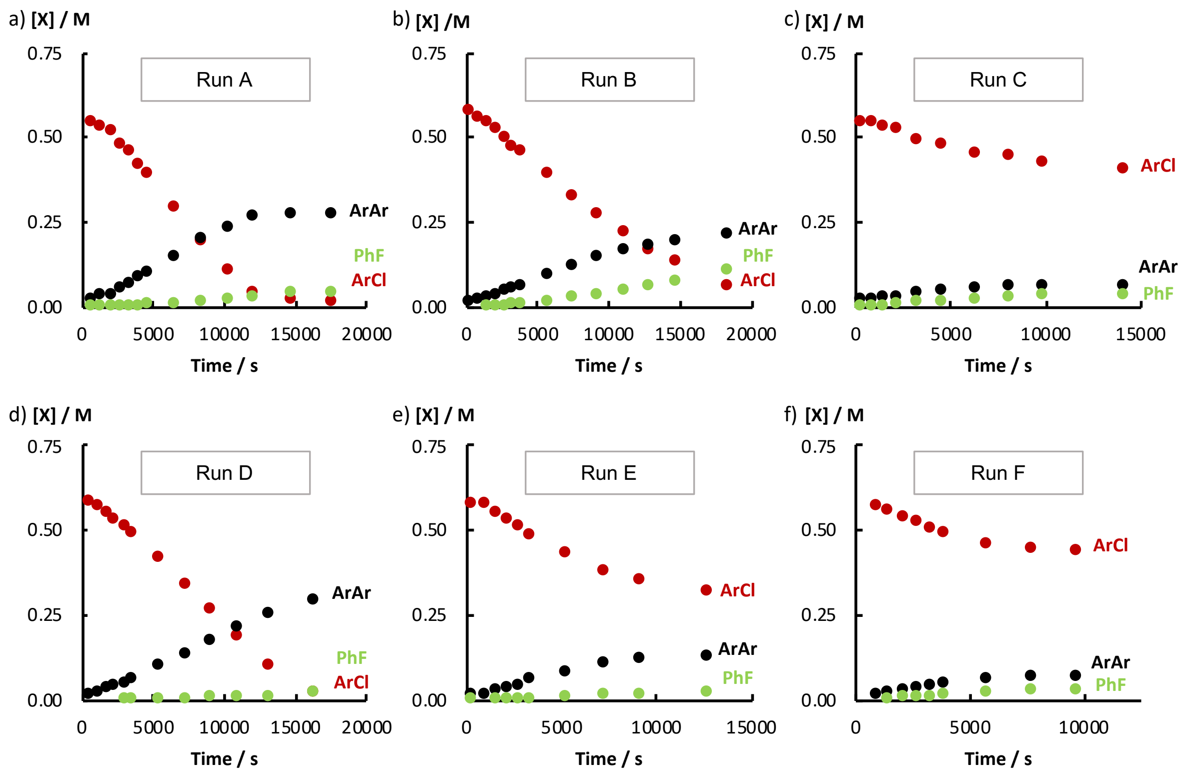

*Figure S22: a – b) Concentration vs time plots of the homocoupling in the presence of various amounts of water. The amount of PhF (**3**) formed during the reaction increased with the amount of added water. c) Concentration vs time plot of the reaction with 1.5 equivalents of H_2O present. The reaction stalls after a few turnovers. d-e) Concentration vs time plots of the reaction in the presence of various amounts of D_2O. There is no significant quantity of p-F-C_6H_4-H (**3**) or p-F-C_6H_4-D (**3**-D) formed indicative that there is a large primary kinetic isotope effect. The reaction stalls in Run E at around 50 % conversion. f) Concentration vs time plot of the reaction with both H_2O and D_2O present. The reaction stalls after a few turnovers. PhF (**3**) is detected before the reaction stalls.*

S4.1.20 Homocoupling in the Presence of Sodium Halide Salts

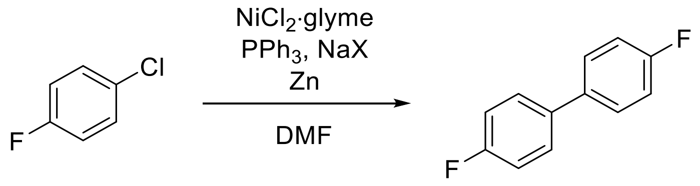

Scheme S15: Homocoupling with sodium salts as additives.

The experiments were performed according to the standard procedure, but with NaCl (39.8 mg, 681 µmol), NaBr (61.5 mg, 598 µmol and 67.0 mg, 651 µmol) and NaI (between 36.6 mg, 244 µmol and 97.1 mg, 648 µmol) added to the reaction flask along with the Ni catalyst, phosphine and Zn powder.

For runs A, B, C and G the quantity of phosphine (around 250 mg, 953 µmol) added at the beginning of the reaction was lower.

For run G, 0.5 mL of the ArCl (**1**) stock solution was added. The concentrations are shown in Table S20.

Table S20: Concentrations and equivalents of reaction components.

All concentrations are in mM, Zn is in equivalents to ArCl (1)

Run	$[IS]_0$	$[1]_0$	$[Ni]_0$	$[PPh_3]_0$	Zn_0 / eq	$[NaX]_0$	NaX
A	464.8	633.9	32.7	245.7	1.52	170.3	NaCl
B	464.8	633.9	31.6	244.5	1.50	149.5	NaBr
C	464.8	633.9	37.0	242.4	1.49	161.9	NaI
D	464.8	633.9	31.3	480.5	1.51	627.3	NaI
E	464.8	633.9	35.5	479.3	1.51	117.4	NaI
F	464.8	633.9	36.7	479.5	1.51	61.1	NaI
G	232.4	316.9	33.8	239.9	3.08	162.8	NaBr

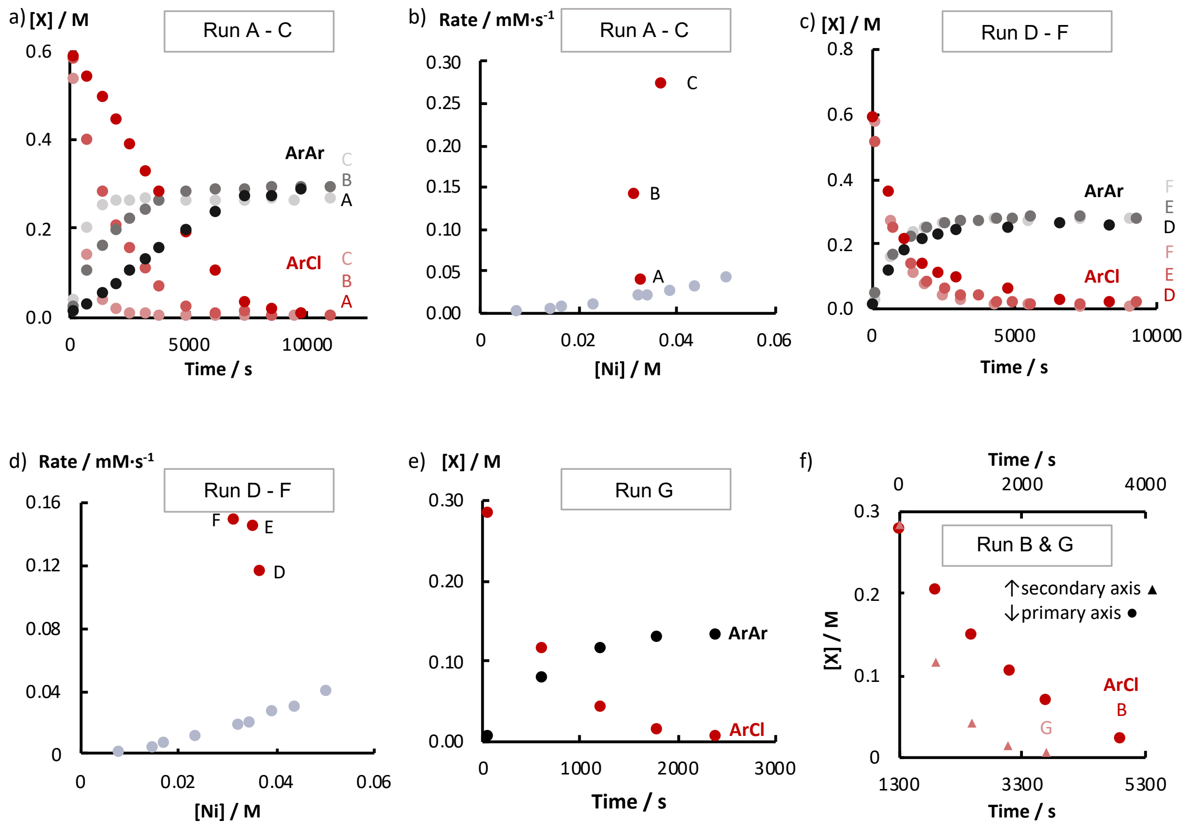

*Figure S23: a) Concentration vs time plot of the homocoupling with NaCl, NaBr and NaI additives b) Reaction rates with NaCl, NaBr and NaI additives vs the catalyst concentration. The reaction rate increases in the presence of the salts. This increase was most apparent with NaI, followed by NaBr. c) Concentration vs time plots of the homocoupling with varied quantity of NaI. The reaction rates only increase slightly with increasing NaI. d) The reaction rates of the reactions with NaI were plotted against the catalyst loading. e) Concentration vs time plot of the reaction in the presence of NaBr but with a lower ArCl (**1**) concentration. f) Comparison of the concentration vs time plot of the reaction with NaBr and 633.9 mM ArCl (**1**) versus 316.9 mM. This comparison, in which one time axis is deliberately shifted to allow this to be visualised, shows that the chloride that is produced by turnover competes with the added bromide, leading to slower rates as the reaction progresses.*

S4.1.21 Homocoupling in the Presence of Chloride Additives (MCl)

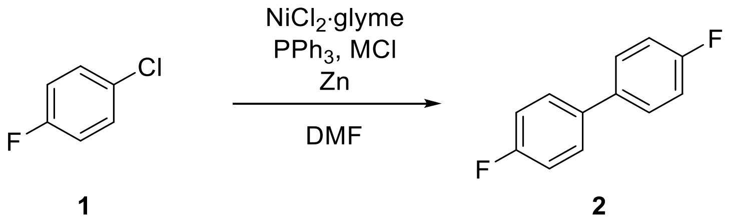

Scheme S16: Reaction Scheme of the Ni catalysed Ullmann coupling with various salts.

The experiments were performed according to the standard procedure, except that LiCl (between 56.5 mg, 675 µmol and 111 mg, 2.62 mmol), NaCl (39.8 mg, 681 µmol) and CsCl (102 mg, 604 µmol) were added to the reaction flask with the Ni catalyst, phosphine (PPh_3) and Zn powder. The quantity of phosphine (around 250 mg, 953 µmol) added at the beginning of the reaction was lower in all runs. The concentrations are shown in Table S21.

Table S21: Concentrations and equivalents of reaction components.

All concentrations are in mM, Zn is in equivalents to ArCl (1)

Run	$[IS]_0$	$[1]_0$	$[Ni]_0$	$[PPh_3]_0$	Zn_0 / eq	$[MCl]_0$	MCl
A	464.8	633.9	31.6	238.6	1.58	168.8	LiCl
B	464.8	633.9	38.9	241.1	1.50	333.3	LiCl
C	464.8	633.9	38.9	241.8	1.54	655.6	LiCl
D	464.8	633.9	32.7	245.7	1.52	170.3	NaCl
E	464.8	633.9	30.3	240.9	1.51	150.9	CsCl

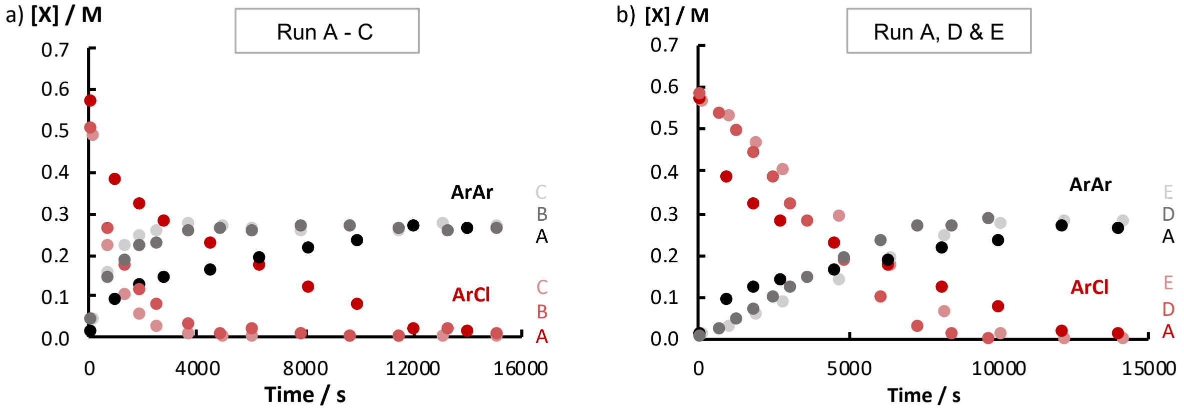

Figure S24: a) Concentration vs time plots of the homocoupling with various LiCl concentrations. The reaction rate increases with increasing concentrations of LiCl. The reaction profile changes significantly in comparison to the standard conditions. b) Concentration vs time plots of the reaction with LiCl, NaCl and CsCl additives. The most notable effect is observed with LiCl, which has an initial burst that levels off to linear behaviour. The kinetic profiles with the NaCl and CsCl additives have a small initiation period.

S4.1.22 Homocoupling in Toluene

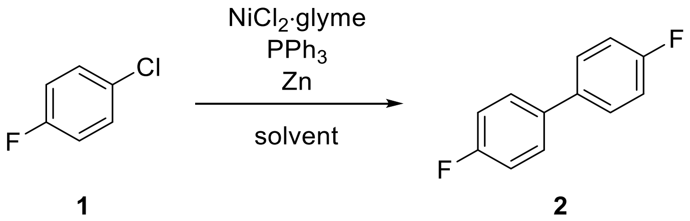

Scheme S17: Ni catalysed homocoupling in toluene and a toluene/DMF mixture.

These experiments were set up according to the standard procedure but with different solvents. Concentrations and solvent mixtures are given in Table S22.

Table S22: Concentrations and equivalents of reaction components. The reactions were run in toluene or toluene/DMF mixtures.

All concentrations are in mM, Zn is in equivalents to ArCl (1)

Run	$[IS]_0$	$[1]_0$	$[Ni]_0$	$[PPh_3]_0$	Zn_0 / eq	Solvent	Rate / mM·s^{-1}
A	464.8	633.9	32.8	478.4	1.51	DMF/Toluene = 4/1	2.28E-03
B	464.8	633.9	33.2	241.4	1.50	Toluene	0

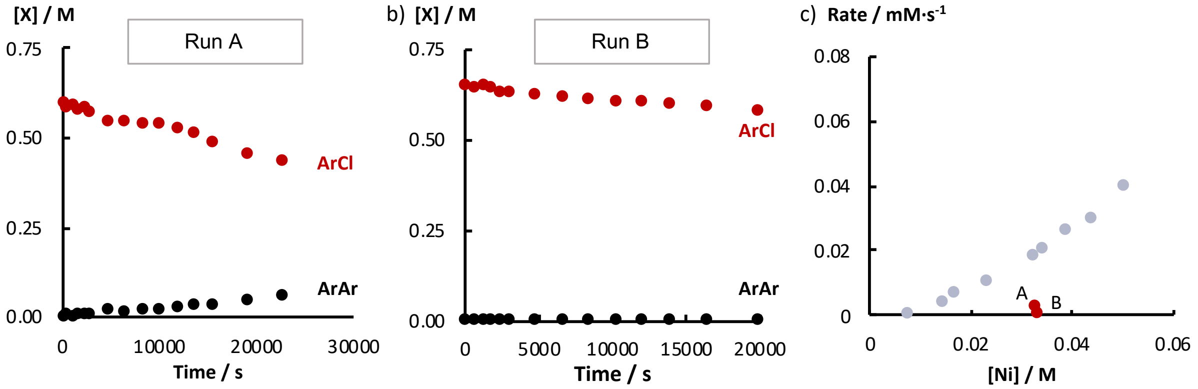

*Figure S25: a) Concentration and time plot of the homocoupling in DMF/toluene. The reaction rate is significantly slower. b) Concentration vs time plot of the reaction in toluene. Very little biaryl (**2**) generation is detected over a period of several hours. c) Reaction rates in toluene and toluene/DMF plotted against the catalyst concentration. Both reactions are significantly slower than the reaction in DMF under standard conditions.*

S4.1.23 Homocoupling in DMAc

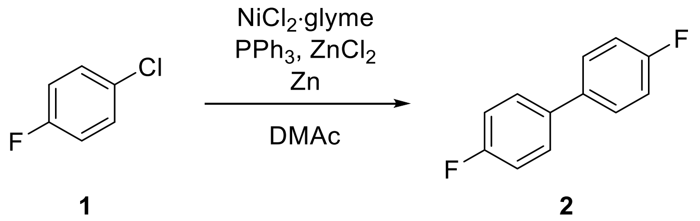

Scheme S18: Ni catalysed homocoupling in DMAc.

These experiments were set up according to the standard procedure but in DMAc instead of DMF. Run B was heated to 80 °C. For run C, $ZnCl_2$ (281 mg, 2.06 mmol) was added after 1h 47 min 30 s under an inert atmosphere. The concentrations and temperatures are given in Table S23.

Table S23: Concentrations and equivalents of reaction components. All reactions are run in DMAc.

All concentrations are in mM, Zn is in equivalents to ArCl (1)

Run	[IS]	[1]	$[Ni]_0$	$[PPh_3]$	Zn_0 / eq	$[ZnCl_2]$	T/ °C	Rate / $mM·s^{-1}$
A	464.8	633.9	30.8	480.8	1.52	0	60	1.93E-02
B	464.8	633.9	30.7	478.6	1.51	0	80	4.16E-02
C	371.8	633.9	36.3	479.4	1.50	749.9[i]	60	1.61E-02
D	371.8	633.9	34.3	243.6	1.51	0	60	2.22E-02
E	464.8	633.9	41.5	482.0	2.98	0	60	2.92E-02

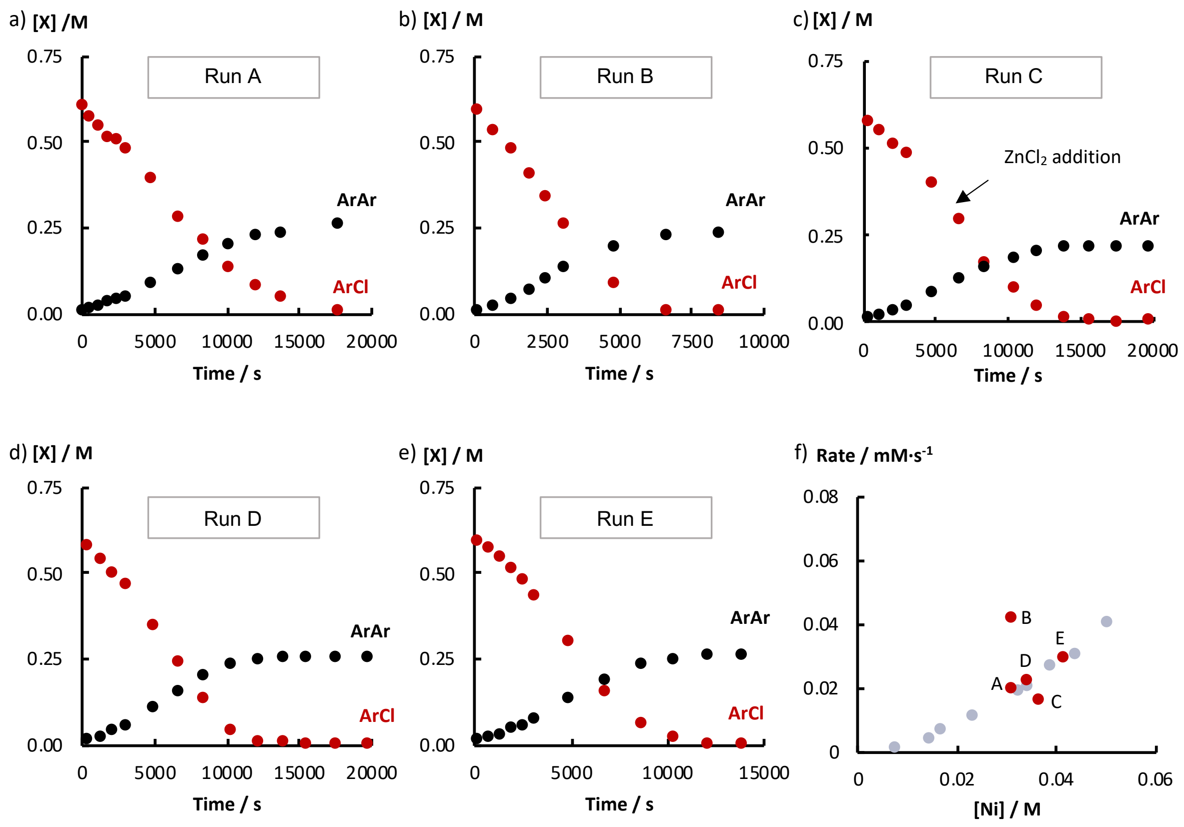

Figure S26: a) Concentration vs time plot of the homocoupling in DMAc. The kinetic profile is similar to those in DMF under standard conditions. b) Concentration vs time plot of the reaction in DMAc at 80 °C; the reaction rate is significantly faster. c) Concentration vs time plot of the reaction in DMAc, with $ZnCl_2$ added during the reaction; unlike reactions in DMF, this induces no stalling or significant change in the kinetic profile. d) Concentration vs time plot of the reaction in DMAc with half the usual phosphine concentration. The reaction profile was comparable to the one of the reactions under standard conditions. Again this is in stark contrast to the reaction in DMF at lower phosphine concentrations which stalls. e) Concentration vs time plot of the reaction in DMAc with an increased quantity of Zn powder. The reaction profile has a small initiation period. f) Reaction rates of the reactions in DMAc plotted against the catalyst concentrations. All reactions have comparable rates as the ones using standard conditions in DMF except run B (at 80 °C) which is faster.

S4.1.24 Effect of reduced ex situ Sampling Frequency on the Homocoupling

For this experiment the standard procedure was followed but less samples were taken. The concentrations are shown in Table S24.

Table S24: Concentrations and equivalents of reaction components.

All concentrations are in mM, Zn is in equivalents to ArCl (1)

Run	$[IS]_0$	$[1]_0$	$[Ni]_0$	$[PPh_3]_0$	Zn_0 / eq	Rate / $mM \cdot s^{-1}$
A	464.8	633.9	28.8	477.7	1.50	1.34E-02

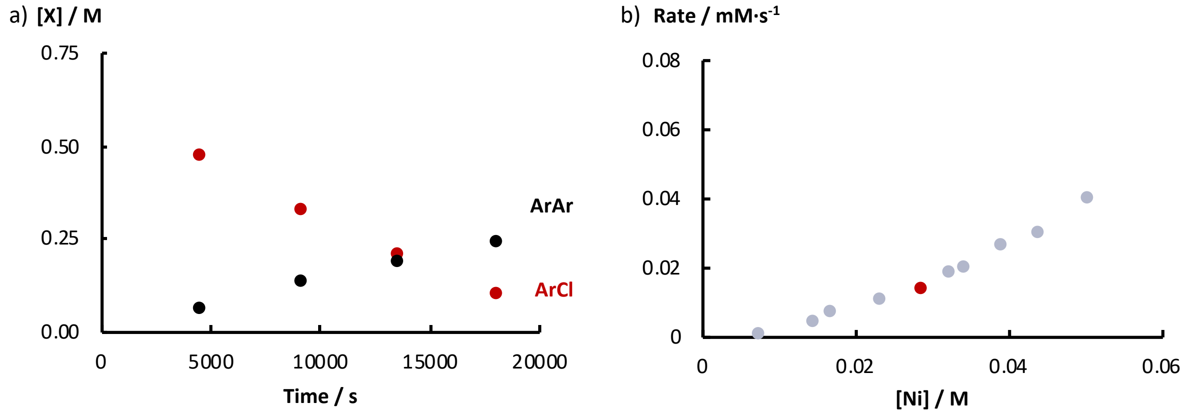

Figure S27: a) Concentration vs time plot of the reaction with a lower sampling frequency. The kinetic profile is similar to the one using the standard sampling frequency. b) Reaction rate at a lower sampling frequency plotted against the catalyst loading. The reaction rate is similar to that using the normal sampling frequency.

S4.1.25 Effect of Reduced Stirring Rate on the Homocoupling

For this experiment the standard procedure was followed but lower stirring rates were employed. The concentrations are shown in Table S25.

Table S25: Concentrations and equivalents of reaction components. The stirring rate was varied.

All concentrations are in mM, Zn is in equivalents to ArCl (1)							
Run	$[IS]_0$	$[1]_0$	$[Ni]_0$	$[PPh_3]_0$	Zn_0 / eq	Stirring Rate / RPM	Rate / mM·s^{-1}
A	464.8	633.9	29.5	474.7	1.53	300	1.07E-02
B	464.8	633.9	39.2	484.7	1.51	500	2.46E-02

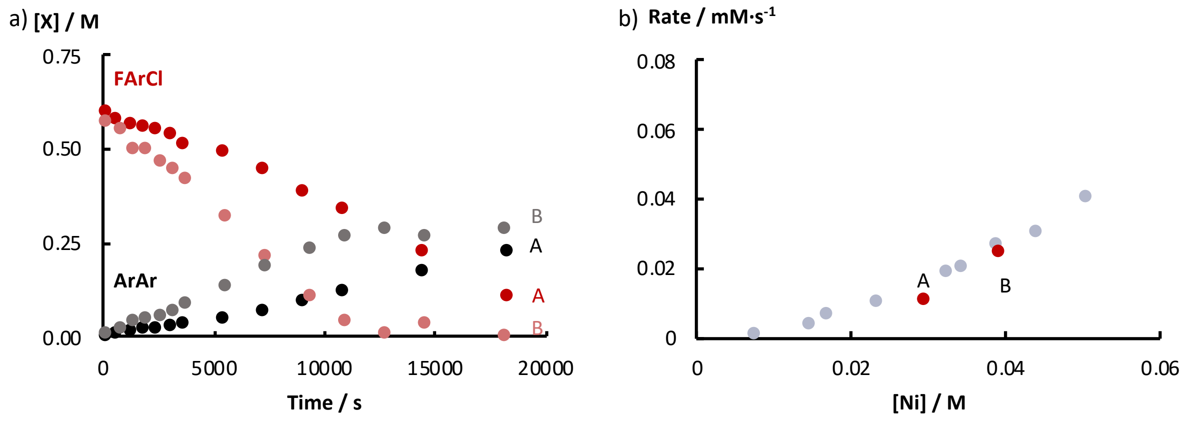

Figure S28: a) Concentration vs time plot of the reactions with lower stirring rates. The reaction profiles were slightly less linear, possibly indicating mass transfer is influential at the start of the reaction. b) Reactions at reduced stirring rates are marginally slower than those at the stirring rate of 715 RPM employed in the standard procedure.

S4.2 Homocoupling with In Situ Reaction Monitoring using a Mixing Device[S6]

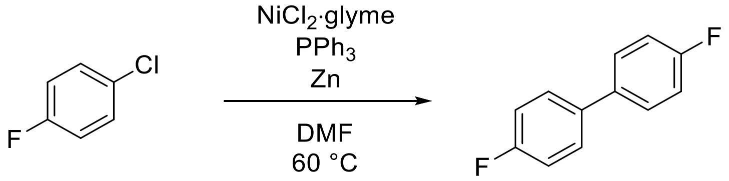

1 **2**

Scheme S19: Reaction scheme of the Ni catalysed homocoupling in the mixing device.

All stock solutions were prepared in a glovebox under nitrogen atmosphere.

S4.2.1.1 [$NiCl_2$(glyme)] stock solution

A stock solution of [$NiCl_2$(glyme)] (22.5 mg, 102 µmol) and PPh_3 (299 mg, 1.1 mmol) in DMF was prepared in a volumetric flask (2 mL).

S4.2.1.2 ArCl stock solution

A stock solution of 1-chloro-4-fluorobenzene (**1**) (940 µL, 8.8 mmol) and 1-fluoronapthalene (IS) (840 µL, 6.51 mmol) in DMF was prepared in a volumetric flask (2 mL).

S4.2.1.3 Shimming and Tuning of the Spectrometer

The spectrometer was shimmed to a sample containing Zn powder (50 mg, 765 µmol) and DMF (700 µL).

S4.2.1.4 Reaction Mixture

Zn powder (59.8 mg, 914 µmol) was added to an NMR tube together with the [$NiCl_2$(glyme)] stock solution (600 µL). The NMR tube was connected to the mixing device[S6] and purged with argon. The catalyst was activated at 60 °C for 30 minutes, by using a plunger speed that ensures distribution of the zinc powder throughout the reaction volume. Then ArCl (**1**) stock solution (100 µL) was added, and the system again purged with argon. The mixing device was transferred into the spectrometer and the reaction was monitored. The concentrations are shown in Table S26.

S4.2.1.5 Spectrometer Settings

Nucleus: ^{19}F
Pulse sequence: personalized sequence, 90 degrees pulse angle
Number of scans: 1
Temperature: 333 K
Mixing time: 300 s
Settling time: 30 s
Plunger speed: 100 mm/s

Table S26: Concentrations and equivalents of reaction components. The reaction was conducted in an in situ NMR mixing device.

All concentrations are in mM, Zn is in equivalents to ArCl (1)

Run	[IS]	[1]	$[Ni]_0$	$[PPh_3]$	Zn_0 / eq	Rate / $mM{\cdot}s^{-1}$
A	464.8	630.5	43.8	488.0	2.07	3.22E-02

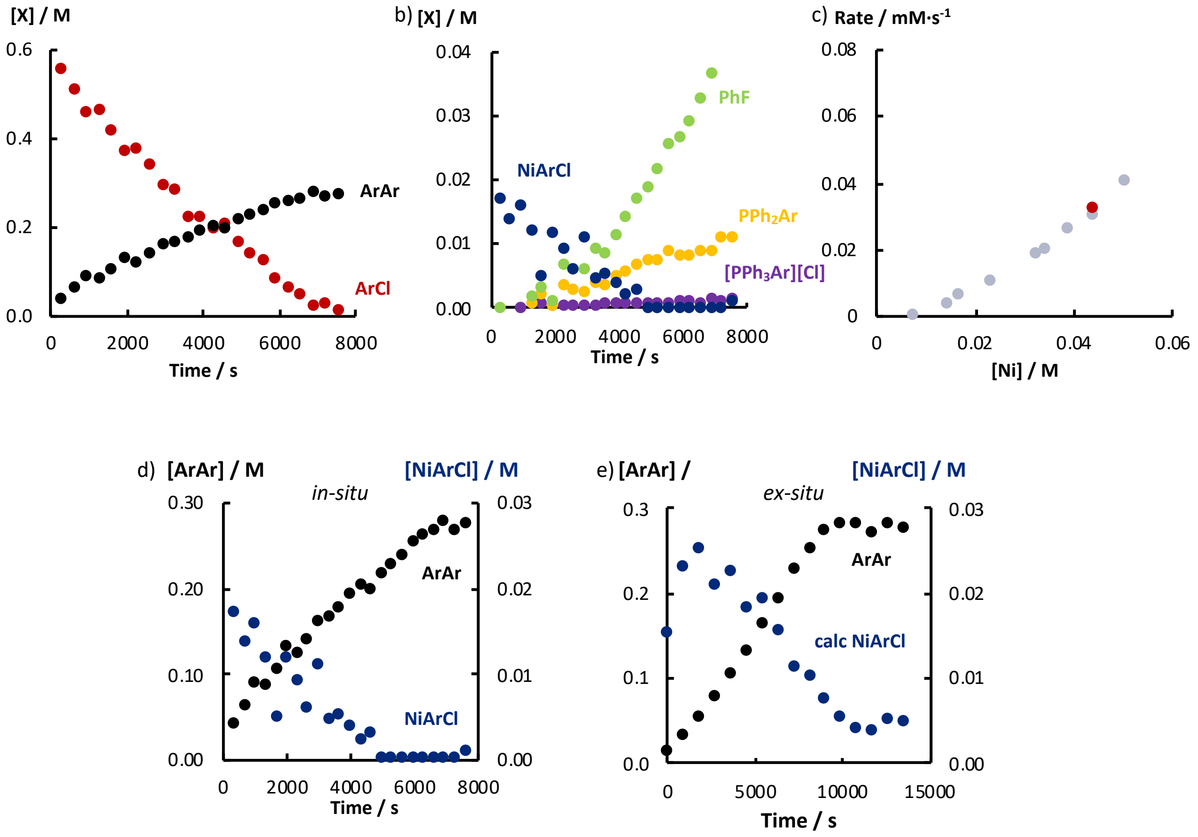

*Figure S29: a) Concentration vs time plot of the homocoupling in the mixing device. There is a significant deadtime between the reaction being set up at ambient temperature and the device being inserted into the spectrometer for monitoring at 60 °C, resulting in some biaryl product (**2**) evident in the first spectrum. b) Concentration vs time plot of the intermediate and side products. The accumulation of the L_2NiArCl (**5**) intermediate occurs during the deadtime between the reaction set up and the monitoring. The heterogenous mixture results in magnetic inhomogeneity and broad signals. c) Rate vs Ni concentration plot of the reaction monitored using the mixing device and reaction using ex-situ monitoring techniques. The reaction rate of the Ullmann coupling in the mixing device compared to the standard ex-situ sampling method. d) and e) Concentration vs time plots of L_2NiArCl (**5**) and ArAr (**2**) using the mixing device and ex situ monitoring. Minor discrepancies may arise from longer deadtime, poorer shimming, and estimation of the theoretical L_2NiArCl (**5**) in the ex-situ monitoring.*

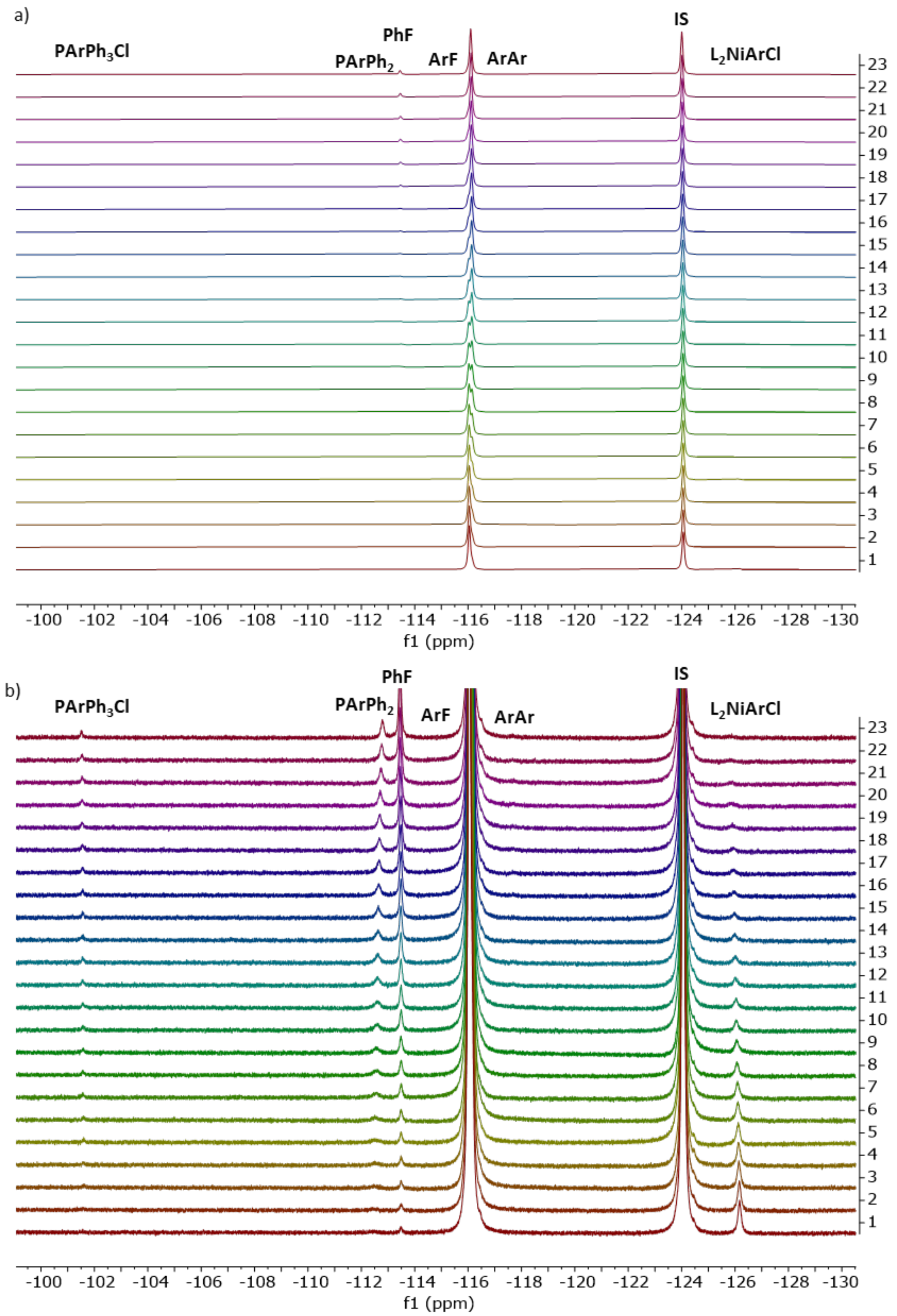

Figure S30: a) and b) ^{19}F NMR spectra in DMF of homocoupling monitored in-situ using a mixing device.

S4.3 Alternating-Agitation-Heating Experiments

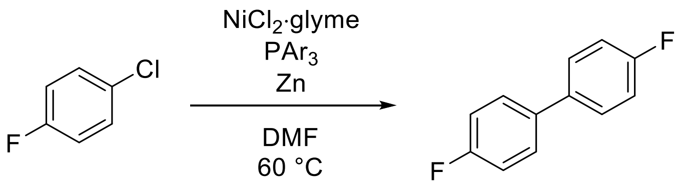

Scheme S20: Ni catalysed homocoupling - alternated-agitation-heating experiment.

S4.3.1.1 General Procedure

J Young valve equipped NMR tubes were dried overnight in an 80 °C vacuum oven and allowed to cool to room temperature in the glove box.

S4.3.2 Standard Procedure

A stock solution of [$NiCl_2$(glyme)] (26.5 mg, 121 µmol) and tri(para-fluorophenyl)phosphine (PAr_3) (351 mg, 1.1 mmol) in DMF (2 mL) was prepared in the glovebox. A second stock solution of 1-chloro-4-fluorobenzene (**1**) (120 µL, 1.1 mmol) and 1-fluoronaphthalene (IS) (110 µL, 852 µmol) in DMF (2 mL) was prepared in the glovebox. The Ni stock solution (0.5 mL) was transferred to an NMR tube equipped with a gas-tight J Young valve and ^{1}H, ^{19}F and ^{31}P NMR spectra acquired. Zn powder (27.1 mg, 415 µmol) and Ni stock solution (0.4 mL) were placed in a second NMR tube equipped with a gas-tight J Young valve. The NMR tube was then heated to 60° C. Every 5 minutes the NMR tube was shaken 20 times. After 30 minutes, 1-chloro-4-fluorobenzene (**1**) stock solution (0.1 mL) was added to the NMR tube. The NMR tube was shaken vigorously and ^{1}H, ^{19}F and ^{31}P NMR spectra were acquired. The NMR tube was heated for 15 minutes and ^{1}H, ^{19}F and ^{31}P NMR spectra were acquired. Then the NMR tube was shaken 20 times and ^{1}H, ^{19}F and ^{31}P NMR spectra were acquired. This process of heating, shaking and acquiring spectra was repeated several times. Afterwards the reaction mixture was quenched with ice water.

S4.3.2.1 Shimming and Tuning of the Spectrometer

The spectrometer was shimmed to the sample at each stage before taking the spectra.

S4.3.2.2 Spectrometer Settings

Nucleus: ^{19}F
Pulse sequence: zg30
Number of scans: 8
Temperature: 300 K
Relaxation Delay: 10 s

Nucleus: ^{31}P
Pulse sequence: zg30
Number of scans: 128
Temperature: 300 K
Relaxation Delay: 2 s

Table S27: Concentrations and equivalents of reaction components. The reaction was set up in NMR tube equipped with a gas-tight J Young valve.

All concentrations are in mM, Zn is in equivalents to ArCl

Run	$[1]_0$	$[IS]_0$	$[Ni]_0$	Zn_0 / eq	$[PAr_3]_0$
A	112.7	85.2	48.3	7.36	443.7

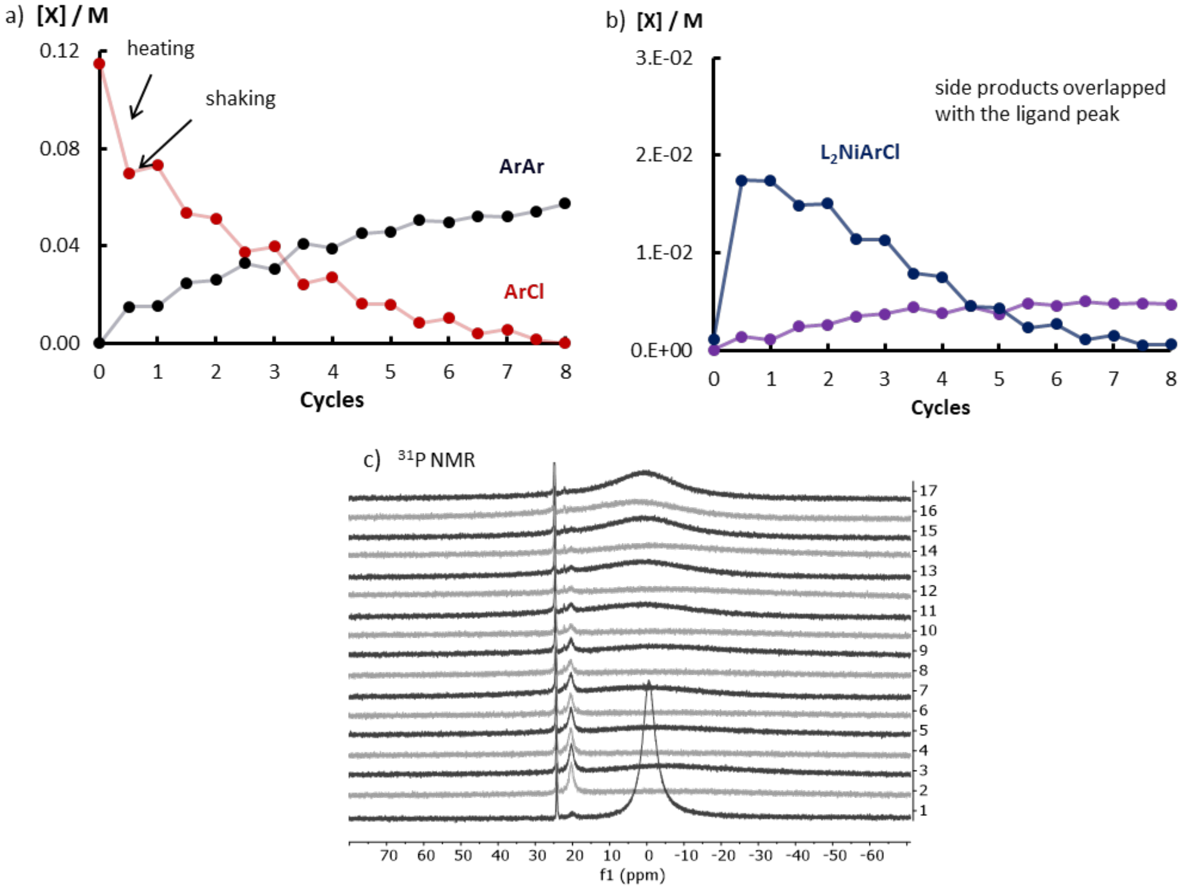

*Figure S31: a) Concentration of ArCl (**1**) and ArAr (**2**) plotted against the agitation-heating cycles. Each cycle started with a heating period followed by an agitation/shaking period. The ArCl (**1**) is consumed in a stepwise manner. b) Concentration of the intermediate and side products in the reaction plotted against the heating-agitation cycles. Some side products and intermediates overlapped with the signals from the free PAr_3 and could therefore not be clearly detected. c) ^{31}P NMR spectra in DMF after each heating and shaking period in the reaction. The signals are broader after the heating period, indicative of changes in exchange rates, Ni-speciation / oxidation states, and relaxation rates.*

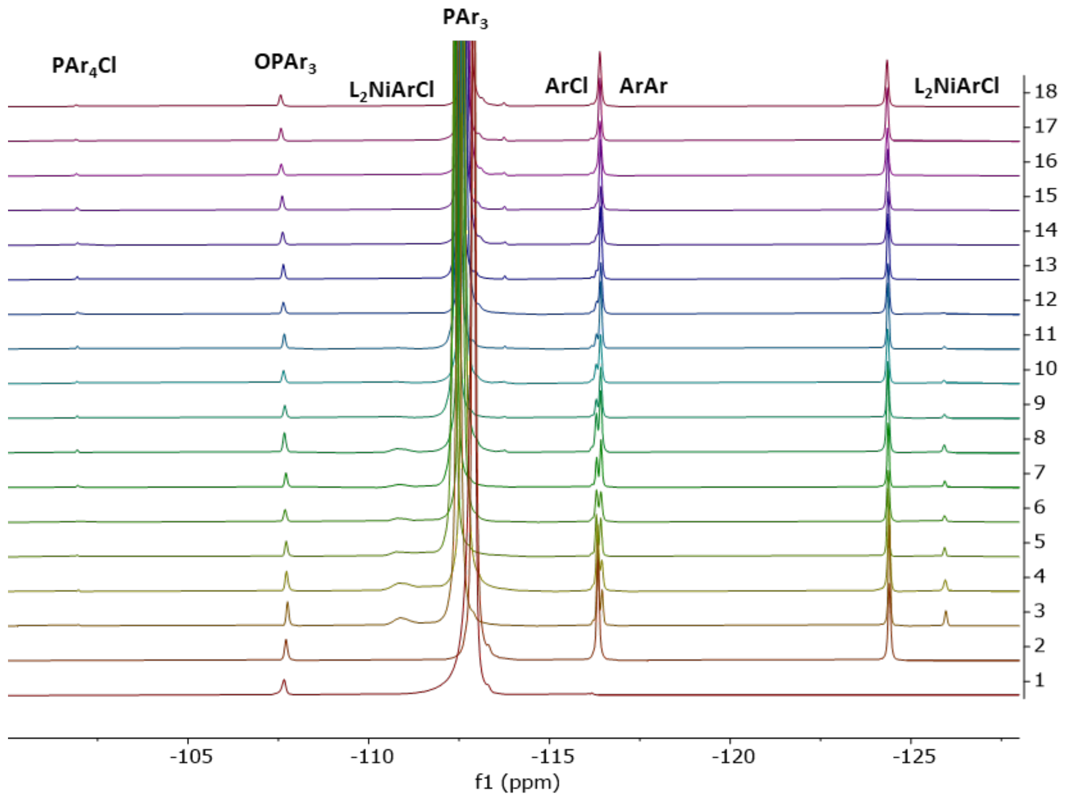

Figure S32: ^{19}F NMR spectra in DMF during the alternated-agitation-heating experiment.

S5 Stoichiometric Homocoupling Experiments

S5.1 *In situ* Monitoring

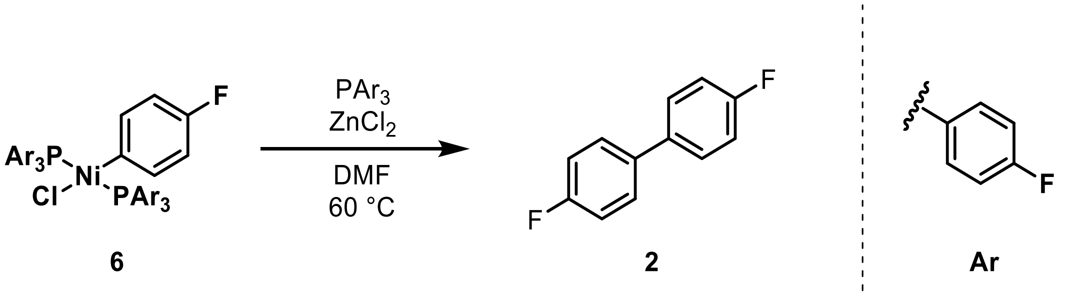

Scheme S21: Standard procedure for in-situ monitoring of stoichiometric homocoupling.

S5.1.1.1 General Procedure

All NMR tubes for *in-situ* monitoring techniques were dried overnight in an 80 °C vacuum oven and allowed to cool down to room temperature in the glove box. Either NMR tubes equipped with gas-tight J Young valves or rubber septa were used. All stock solutions were prepared in the glovebox. All reactions were run at 60 °C unless stated otherwise.

S5.1.2 Standard Procedure

S5.1.2.1 Ni-stock solution

L_2NiArCl (**6**) was carefully ground to a fine powder, in the glove box. A stock solution of L_2NiArCl (**6**), 1-fluoronaphthalene (IS) and tris(4-fluorophenyl)phosphine (PAr_3) was prepared in DMF. The mixture was shaken vigorously to dissolve complex.

S5.1.2.2 $ZnCl_2$ stock solution:

A stock solution of $ZnCl_2$ was prepared in DMF in a volumetric flask. The solution was transferred in a vial with septum.

S5.1.2.3 Shimming and Tuning of the Spectrometer

500 µL of the Ni stock solution was transferred into an NMR tube with a septum and the spectrometer shimmed to ^{1}H and tuned to ^{19}F at 60 °C.

S5.1.2.4 Reaction mixture

A second sample of the Ni-stock solution (500 µL) was added to an NMR tube with septum. The NMR tube and $ZnCl_2$ stock solution were transferred out of the glovebox. The NMR tube was heated to 60 °C for 1 minute to dissolve L_2NiArCl (**6**). A ^{19}F NMR spectrum was acquired and the concentration of L_2NiArCl (**6**) calculated. The amounts of $ZnCl_2$ stock solution and DMF (0 -100 µL) added to the NMR tube were varied to result in the concentrations given in Table S28. The total volume in the NMR tube was 600 µL.

S5.1.2.5 Spectrometer Settings

Nucleus: ^{19}F
Pulse sequence: zg30
Relaxation delay: 10 s
Number of scans: 1
Temperature: 333 K (unless otherwise stated)
Inter scan delay: adapted to fit the length of the reaction (between 0s and 180 s)
For all other parameters standard settings were used.

S5.1.2.6 Calculations and Plots

PAr_4Cl and $OPAr_3$ were identified by their similarity in chemical shifts in the *ex-situ* experiments. A ^{19}F NMR signal assigned to a minor unidentified [NiL_n] species, was confirmed as not be from the aryl group of $L_2NiArCl$ (**6**), by mass balance calculations. This NMR signal is assumed to belong to a PAr_3 group which is coordinated to Ni, (PAr_3 does not coordinate to $ZnCl_2$ under these conditions and the species is observed in reactions with and without $ZnCl_2$ additive). For the concentration calculation it is nominally assumed that a single ligand is coordinated.

The initial concentration of the Ar-Ni complex, [**6**]$_0$, was calculated in each run from the signals in the first NMR spectrum. This avoids errors caused by the reaction starting during transfer from the glove-box at room temperature into the spectrometer, and from some $L_2NiArCl$ (**6**) precipitation from the stock solution as it cools.

Table S28: Concentrations of reagents under standard conditions.

All concentrations are in mM

Run	$[6]_0$	$[IS]_0$	$[PAr_3]_0$	$[ZnCl_2]_0$	k_{obs}
A	7.9	8.1	160.0	0.0	7.73E-02 $M^{-1}\cdot s^{-1}$
B	7.1	8.1	160.0	30.4	5.11E-03 s^{-1}

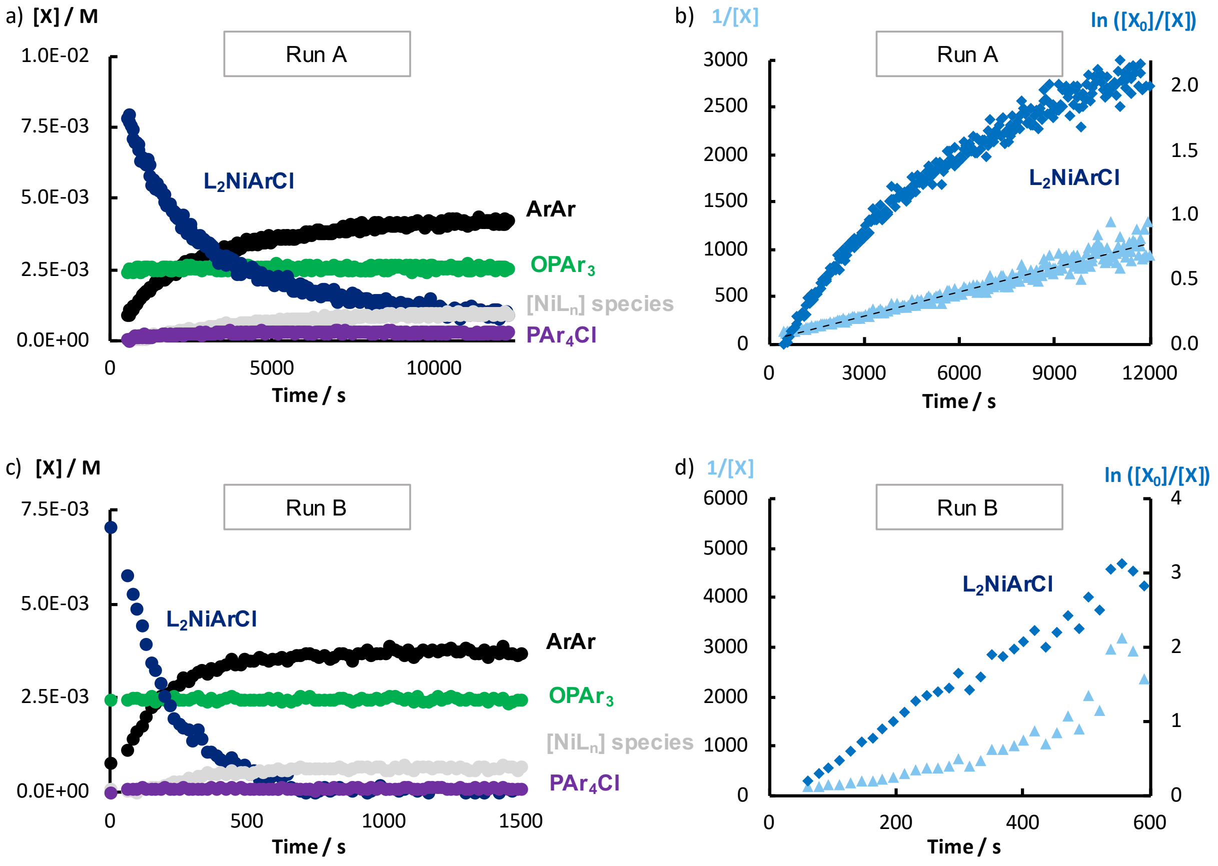

*Figure S33: a) Concentration vs time plot of the coupling using standard procedure without the addition of $ZnCl_2$. b) Plots to determine that the reaction is second order in $L_2NiArCl$ (**6**): 1/[X] axis gives a linear trend. c) Concentration vs time plot of the reaction under standard conditions, but with $ZnCl_2$ in solution. D) Plots to determine that the reaction is first or pseudo-first order in $L_2NiArCl$ (**6**): ln([X_0]/[X]) gives a linear trend.*

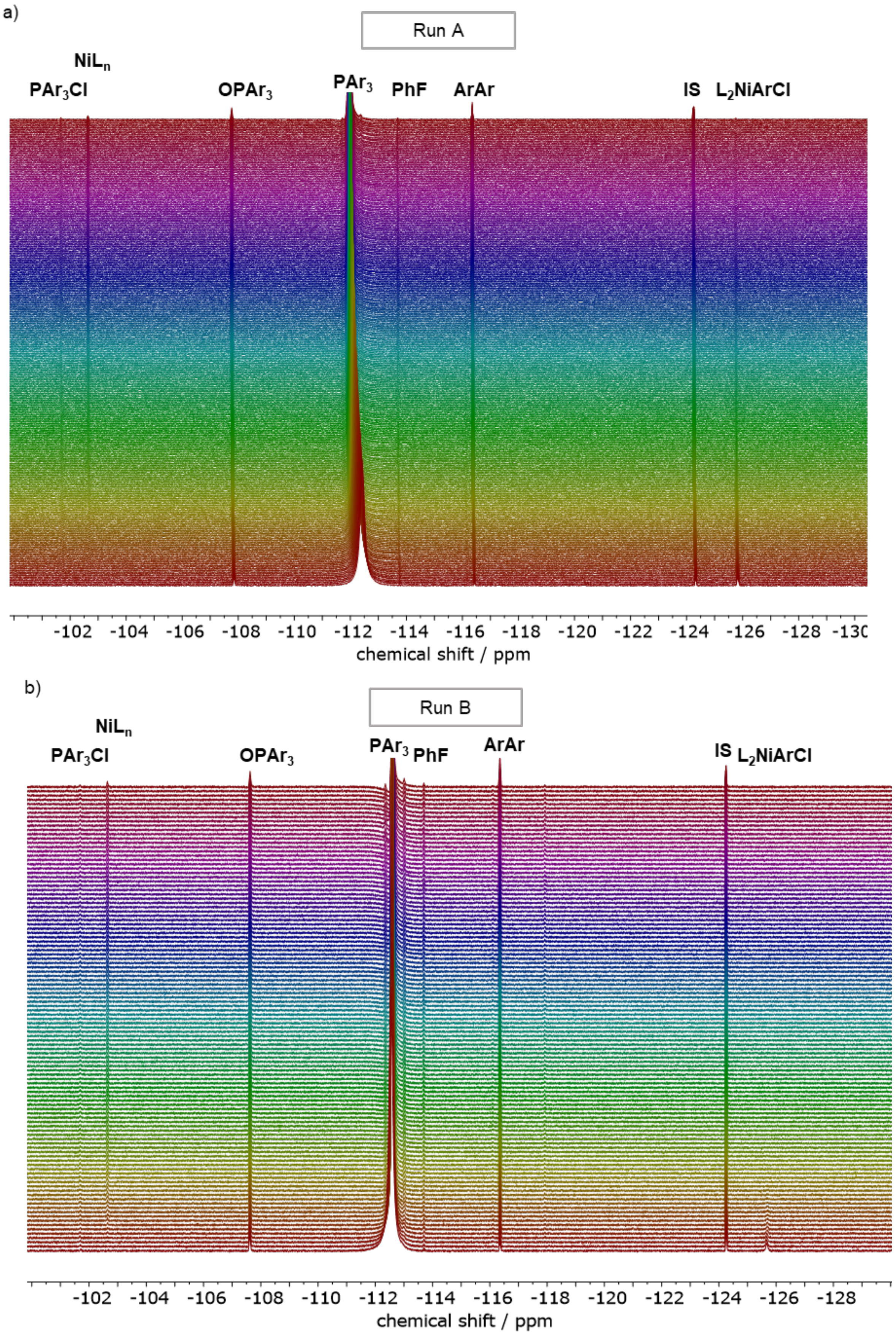

*Figure S34: ^{19}F spectrum of the stoichiometric reaction (a) without $ZnCl_2$ b) with $ZnCl_2$ in solution) of $L_2NiArCl$ (**6**) monitored using in-situ NMR methods.*

S5.1.3 Homocoupling of $L_2NiArCl$ (6) at Various $ZnCl_2$ Concentrations

The reactions were set up according to the standard procedure. The quantities of $ZnCl_2$ in DMF added were varied between runs. The total volume in each NMR tube was 600 µL. The concentrations for each run are given in Table S29.

S5.1.3.1 Ni stock solution (see standard procedure)

$L_2NiArCl$ (**6**, 60.65 mg, 73.8 µmol), PAr_3 (306.43 mg, 969 µmol) and 1-fluoronaphthalene (IS) (6.5 µL, 50.4 µmol) in DMF (5mL).

S5.1.3.2 $ZnCl_2$ stock solutions (see standard procedure)

$ZnCl_2$ (100.79 mg, 739 µmol) in DMF (2 mL).

*Table S29: Concentrations of reactants in stoichiometric homocoupling of **6** with varying $ZnCl_2$ concentrations.*

All concentrations are in mM

Run	$[6]_0$	$[IS]_0$	$[PAr_3]_0$	$[ZnCl_2]_0$
A	8.45	8.39	161.43	3.08
B	8.84	8.39	161.43	6.16
C	8.89	8.39	161.43	15.41
D	9.19	8.39	161.43	30.81
E	9.46	8.39	161.43	30.81
F	8.85	8.39	161.43	46.22
G	9.01	8.39	161.43	61.63

All ^{19}F NMR spectra of run G were summed up using the topspin command fidadd to give a new spectrum. All spectra between 0 and around 90 % conversion were summed up as well as all spectra between around 90 - 100% conversion. All three spectra are shown in Figure S12. A small peak in the chemical shift region of ZnArCl (**16**) is detected. This peak is more prominent in the last 10 % of the reaction which fits with the proposed mechanism as the ZnArCl (**16**) reacts quickly with $L_2NiArCl$ (**6**). At low $L_2NiArCl$ (**6**) concentration the rate of this transmetalation is slower than at high concentration and the signal from ZnArCl (**16**) can be detected. The high affinity of $ZnCl_2$ (in this case 62 mM) for redistribution of $ZnAr_2$ (**19**) to ZnArCl (**16**), see Section S5.2.4, suggests that the signal is not from the diarylzinc.

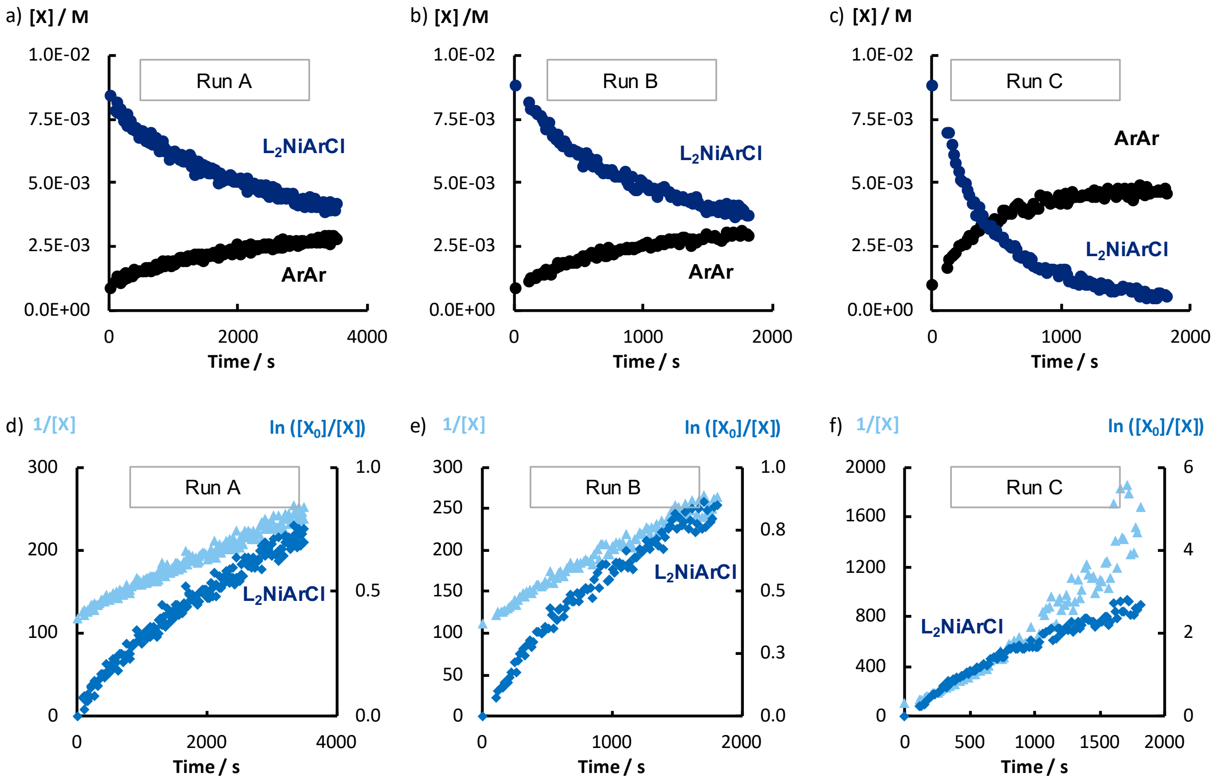

*Figure S35: a-c) Concentration vs time plots of the stoichiometric homocoupling of **6** at various $ZnCl_2$ concentrations. The reaction rate increases with increasing $ZnCl_2$ concentration, and the kinetic profiles progress from second to mixed order (d-f).*

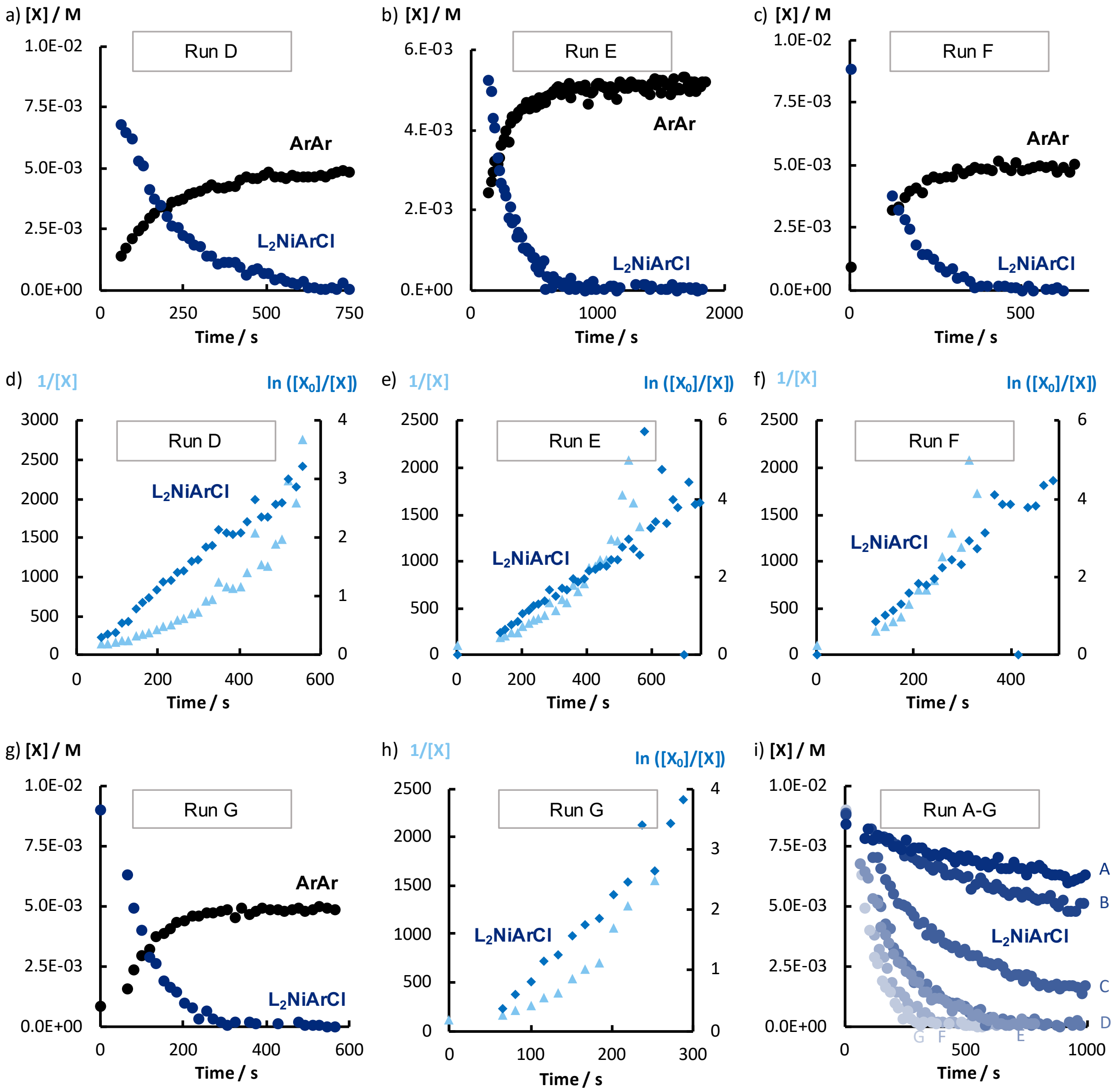

*Figure S36: a-c) Concentration vs time plots of the stoichiometric homocoupling of **6** at various $ZnCl_2$ concentrations. The reaction rate increases with increasing $ZnCl_2$ concentration, and the kinetic profiles progress (d-f) from mixed second-first to first order. g) Concentration vs time plot of run G. h) Plot to determine the first-order kinetics for $L_2NiArCl$ (**6**). i) Concentration vs time plot of $L_2NiArCl$ (**6**) of runs A to G. An increase in reaction rate and change in kinetic profiles is apparent on increasing $ZnCl_2$ concentration.*

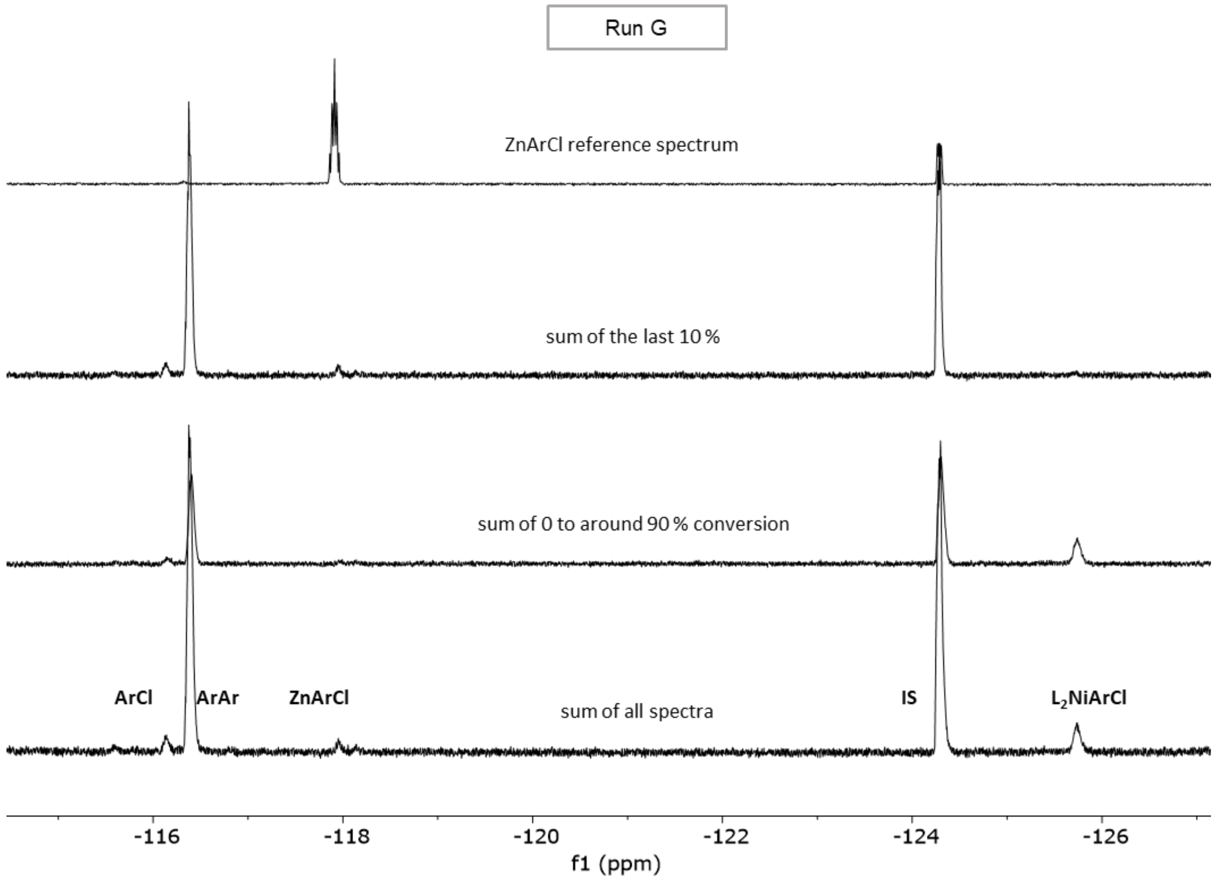

*Figure S37: Sum of all spectra from run G, the first 90 % and last 10 % conversion, and a reference sample of ZnArCl (**16**). A low-intensity signal at -118 ppm is observed at the same chemical shift as ZnArCl (**16**) in DMF. This supports the conclusion from the kinetic studies that ZnArCl (**16**) is an intermediate in the homocoupling of **6** catalysed by $ZnCl_2$.*

S5.1.4 Direct Homocoupling of $L_2NiArCl$ (6) at Various PAr_3 Concentrations

S5.1.4.1 Ni stock solution (see standard procedure)

For run A, B and E:
$L_2NiArCl$ (**6**, 25.7 mg, 31.3 µmol), PAr_3 (80.3 mg, 254 µmol) and 1-fluoronaphthalene (IS) (2.5 µL, 19.4 µmol) in DMF (2 mL).

For run C:
$L_2NiArCl$ (**6**, 25.4 mg, 30.9 µmol), PAr_3 (164 mg, 518 µmol) and 1-fluoronaphthalene (IS) (2.5 µL, 19.4 µmol) in DMF (2 mL).

For run D:
$L_2NiArCl$ (**6**, 24.2 mg, 19.4 µmol), PAr_3 (122 mg, 384 µmol) and 1-fluoronaphthalene (IS) (2.5 µL, 19.4 µmol) in DMF (2 mL).

S5.1.4.2 Reaction Mixture

Runs C, D and E were prepared according to the standard procedure: Ni stock solution (0.5 mL) and DMF (100 µL). For runs A and B, the Ni stock (0.5 mL) was mixed with DMF (100 µL) and PAr_3 (run A: 85.1 mg PAr_3, run B: 40.5 mg) in the glovebox.

*Table S30: Concentration of reactants in the stoichiometric homocoupling of **6** at with various PAr_3 concentrations*

All concentrations are in mM

Run	$[6]_0$	$[IS]_0$	$[PAr_3]_0$	k_{obs} / $M^{-1}s^{-1}$
A	10.3	8.1	554.3	9.50E-03
B	9.6	8.1	319.3	1.98E-02
C	7.8	8.1	215.8	4.17E-02
D	7.9	8.1	160.0	8.40E-02
E	6.5	8.1	105.8	1.87E-01

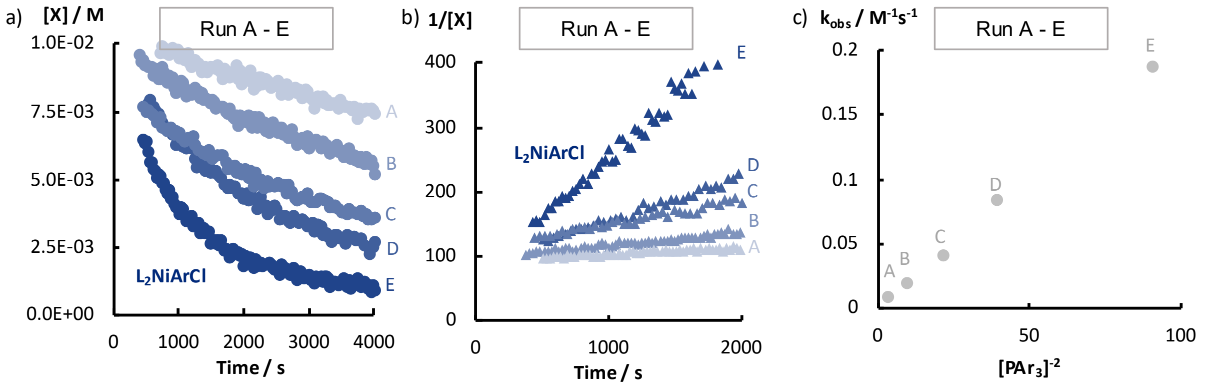

Figure S38: a) Concentration vs rate plot of runs A-E. The rate of the reaction decreases with increasing phosphine concentrations. b) Reciprocal concentration plot of runs A-E. The empirical second order rate constants (k_{obs}) decrease with increasing phosphine concentration. C) Analysis of the empirical rate constants as a function of the reciprocal of the squared phosphine concentration. This preliminary analysis indicates an inverse second order dependency on PAr_3; $k_{obs} = 2.3 \times 10^{-3} / [L]^2$. See Section S8 for a more holistic analysis.

S5.1.5 $ZnCl_2$ Catalysed Homocoupling of L_2NiArCl (6) at Various PAr_3 Concentrations

S5.1.5.1 Ni stock solution (see standard procedure)

For run A, B and E:

L_2NiArCl (**6**, 26.3 mg, 32.0 µmol), PAr_3 (84.8 mg, 268 µmol) and 1-fluoronaphthalene (IS) (2 µL, 15.5 µmol) in DMF (2 mL)

For run C, D and F:

L_2NiArCl (**6**, 27.5 mg, 33.4 µmol), PAr_3 (81.8 mg, 259 µmol) and 1-fluoronaphthalene (IS) (2 µL, 15.5 µmol) in DMF (2 mL)

S5.1.5.2 $ZnCl_2$ stock solutions (see standard procedure)

$ZnCl_2$ (99.4 mg, 730 µmol) in DMF (2 mL)

S5.1.5.3 PAr_3 stock solutions

A stock solution PAr_3 (412 mg, 316 µmol) in DMF was prepared in a volumetric flask (1 mL).

S5.1.5.4 Reaction Mixture

Run C, D, E and F were prepared according to the standard procedure. (C&D: Ni stock solution (0.5 mL), $ZnCl_2$ stock solution (50 µL) and PAr_3 stock solution (50 µL); E&F: Ni stock (0.5 mL), $ZnCl_2$ stock solution (50 µL) and DMF (50 µL)).

For run A and B: 0.5 mL Ni stock were mixed with 50 µL DMF, 50 µL $ZnCl_2$ and PAr_3 (run A: 39.4 mg PAr_3, run B: 84.8 mg) in the glovebox.

*Table S31: Concentration of reactants in the $ZnCl_2$ catalysed homocoupling of **6** at varied PAr_3 concentrations.*

All concentrations are in mM

Run	$[6]_0$	$[IS]_0$	$[PAr_3]_0$	$[ZnCl_2]_0$
A	9.2	6.5	555.5	30.4
B	9.1	6.5	316.3	30.4
C	9.8	6.5	216.3	30.4
D	9.2	6.5	162.1	30.4
E	7.5	6.5	108.8	30.4
F	8.4	6.5	107.8	30.4

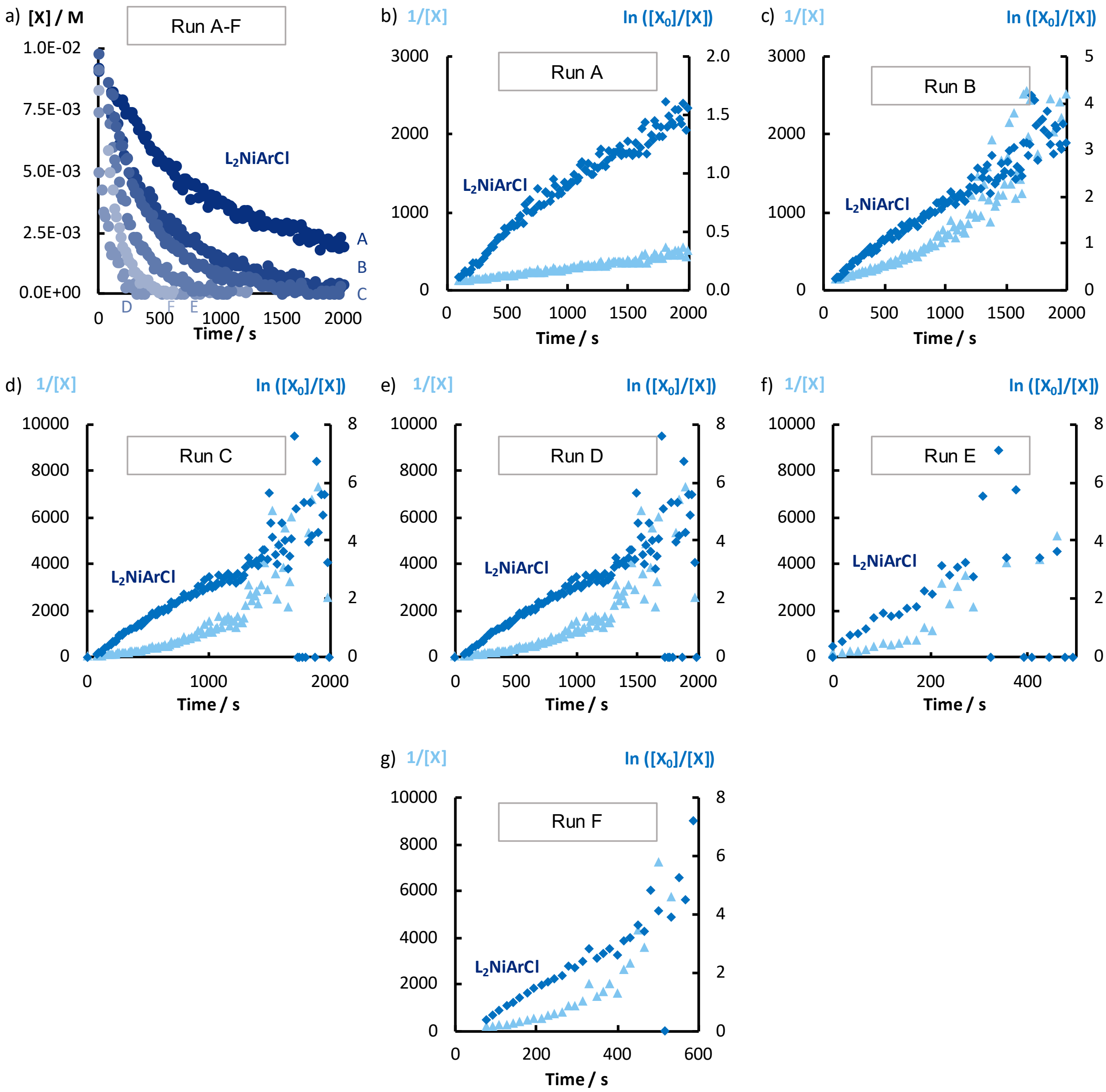

*Figure S39: a) Concentration vs time plot of runs A-F. The rate increases at lower PAr_3 concentrations. b-g) Plots to determine the apparent reaction orders as second through mixed to first order in $L_2NiArCl$ (**6**) as the phosphine concentration is reduced.*

S5.1.6 Semicatalytic Homocoupling of $L_2NiArCl$ (6) and ArCl

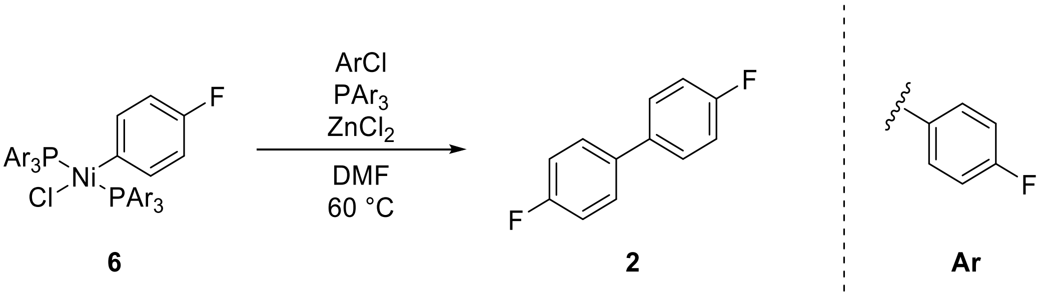

*Scheme S22: Semicatalytic homocoupling of $L_2NiArCl$ (**6**) and ArCl.*

The reaction mixture was prepared according the to standard procedure with the following changes:

In all runs 50 µL of ArCl (**1**) stock solution was added before the NMR tube was transferred out of the glovebox.

In run B, 50 µL of $ZnCl_2$ stock solution was added to the NMR tube to initiate the reaction immediately before monitoring, in run A, 50 µL of DMF was added to the NMR tube.

In run C, 50 µL of $ZnCl_2$ stock solution was added to the NMR tube after 10215s (2h 50 min) of monitoring.

S5.1.6.1 ArCl stock solution

A stock solution of 1-chloro-4-fluorobenzene (**1**, 65 µL, 610 mM) in DMF was prepared in a volumetric flask (2 mL).

S5.1.6.2 Ni stock solution (see standard procedure)

For run A and B:

$L_2NiArCl$ (**6**, 26.7 mg, 32.5 µmol), PAr_3 (122.4 mg, 386 µmol) and 1-fluoronaphthalene (IS) (2 µL, 15.5 µmol) in DMF (2mL)

For run C:

$L_2NiArCl$ (**6**, 28.58 mg, 34.8 mM), PAr_3 (125.3 mg, 396 µmol) and 1-fluoronaphthalene (2 µL, 14.4 µmol) in DMF (2 mL)

S5.1.6.3 $ZnCl_2$ stock solutions (see standard procedure)

For run A and B:

$ZnCl_2$ (50.9 mg, 37.3 µmol) in DMF (1 mL)

For run C:

$ZnCl_2$ (51.0 mg, 37.4 µmol) in DMF (1 mL)

*Table S32: Concentration of reactants in the semicatalytic homocoupling reactions with ArCl (**1**).*

All concentrations are in mM

Run	$[6]_0$	$[IS]_0$	$[1]_0$	$[PAr_3]_0$	$[ZnCl_2]_0$
A	8.0	6.5	25.4	161.2	0.0
B	7.8	6.5	25.4	161.2	31.1
C	9.3	6.5	25.4	165.0	31.2[i]

i) $ZnCl_2$ was added to the reaction after around 10350 s (2h 52 min 30 s).

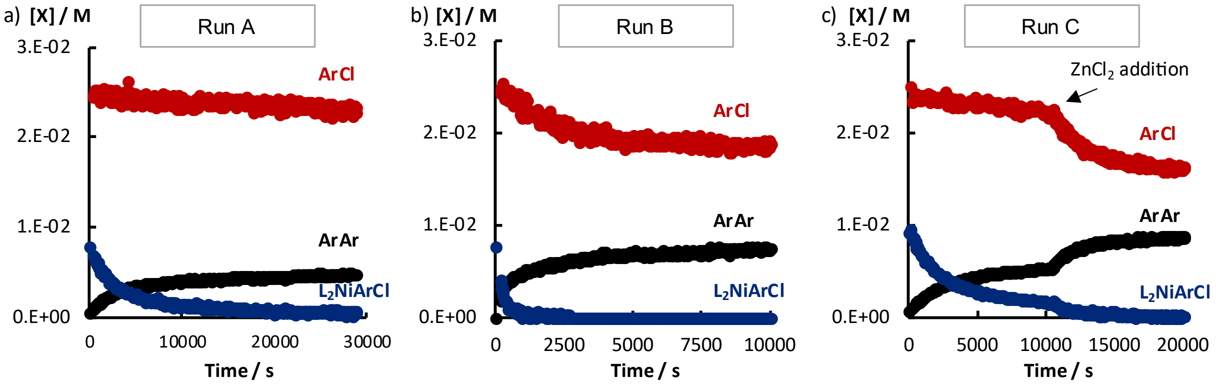

*Figure S40: a) Concentration vs time plot of the semicatalytic homocoupling of $L_2NiArCl$ (**6**) and ArCl (**1**), without $ZnCl_2$ present. The ArCl (**1**) consumption is very slow, indicating that ArCl (**1**) only undergoes very slow, or no, addition to the Ni^I coproduct, resulting in very slow turnover. b) Concentration vs time plot of the stoichiometric reaction with $ZnCl_2$ in solution. The quantity of ArCl (**1**) consumed corresponds to full repeated recycling of the Ni^0. c) Concentration vs time plot of the stoichiometric reaction with ArCl (**1**) when $ZnCl_2$ is added during the reaction. On the addition of $ZnCl_2$, the rate of ArCl (**1**) is markedly increased, consistent with $ZnCl_2$ biasing against comproportionation equilibrium of Ni^0 + Ni^{II} with Ni^I. The ArCl (**1**) that is consumed corresponds to full repeated recycling of the Ni^0.*

S5.1.7 Semicatalytic homocoupling of ArCl (1) by [Ni(PAr$_3$)$_n$(DMF)$_m$] and [(PAr$_3$)$_3$NiCl].

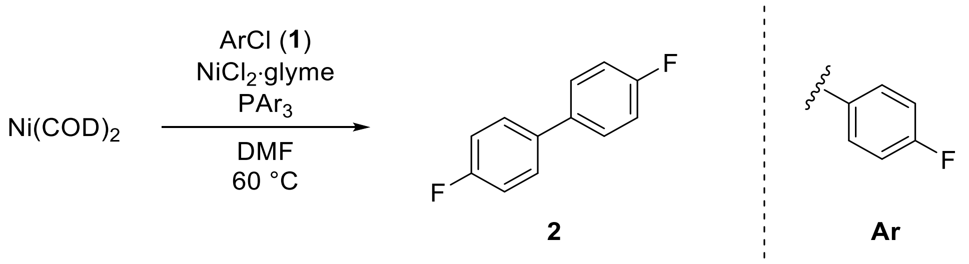

*Scheme S23: Stoichiometric reaction of Ni(COD)$_2$, NiCl$_2$·glyme and ArCl (**1**).*

S5.1.7.1 Ni(COD)$_2$ stock solution
A stock solution of [Ni(COD)$_2$] (8.8 mg, 31.9 µmol) and PAr$_3$ (140 mg, 442 µmol) in DMF (2 mL) was prepared in the glovebox.

S5.1.7.2 [NiCl$_2$(glyme)] stock solution
A stock solution of [NiCl$_2$(glyme)] (8.3 mg, 37.7 µmol) and PAr$_3$ (141 mg, 445 µmol) in DMF (2 mL) was prepared in the glovebox.

S5.1.7.3 ArCl stock solution
A stock solution of 1-chloro-4-fluorobenzene (**1**, 65 µL, 610 µmol) in DMF was made in a volumetric flask (2 mL).

S5.1.7.4 ZnCl$_2$ stock solution (see standard procedure)
ZnCl$_2$ (54.7 mg, 401 µmol) in DMF (1 mL).

S5.1.7.5 Reaction Mixture, shimming and tuning of the spectrometer
For run A: the Ni(COD)$_2$ stock solution (500 µL) solution and DMF (50 µL) were added to an NMR tube which was sealed with a septum in the glovebox and then removed. The spectrometer was shimmed and tuned to ^{19}F using this sample. The ArCl (**1**) stock solution (50 µL) was added, and the reaction monitored by ^{19}F NMR.

For run B: the Ni(COD)$_2$ stock solution (500 µL) solution and the ZnCl$_2$ stock solution (50 µL) were added to an NMR tube which was sealed with a septum in the glovebox and then removed. The spectrometer was shimmed and tuned to ^{19}F using this sample. The ArCl (**1**) stock solution (50 µL) was added, and the reaction monitored by ^{19}F NMR.

For run C: the Ni(COD)$_2$ stock solution (250 µL), the [NiCl$_2$(glyme)] stock solution (250 µL), and DMF (50 µL) were added to an NMR tube which was sealed with a septum in the glovebox and then removed. The spectrometer was shimmed and tuned to ^{19}F using this sample. The ArCl (**1**) stock solution (50 µL) was added, and the reaction was monitored by ^{19}F NMR.

For run D: the Ni(COD)$_2$ stock solution (250 µL), the NiCl$_2$·glyme stock solution (250 µL) and ZnCl$_2$ stock solution (50 µL) were added to an NMR tube which was sealed with a septum in the glovebox and then removed. The spectrometer was shimmed and tuned to ^{19}F using this sample. The ArCl (**1**) stock solution (50 µL) was added, and the reaction was monitored by ^{19}F NMR.

The total volume of each sample was 600 µL.

*Table S33: Concentration of reactants in the stoichiometric reactions of ArCl (**1**) with Ni^0.*

All concentrations are in mM

Run	$[Ni(COD)_2]_0$	$[IS]_0$	$[PAr_3]_0$	$[1]_0$	$[ZnCl_2]_0$	$[NiCl_2]_0$
A	13.3	9.7	184.3	25.4	0.0	0.0
B	13.3	9.7	184.3	25.4	33.4	0.0
C	6.6	4.8	184.9	25.4	0.0	7.9
D	6.6	4.8	184.9	25.4	33.4	7.9

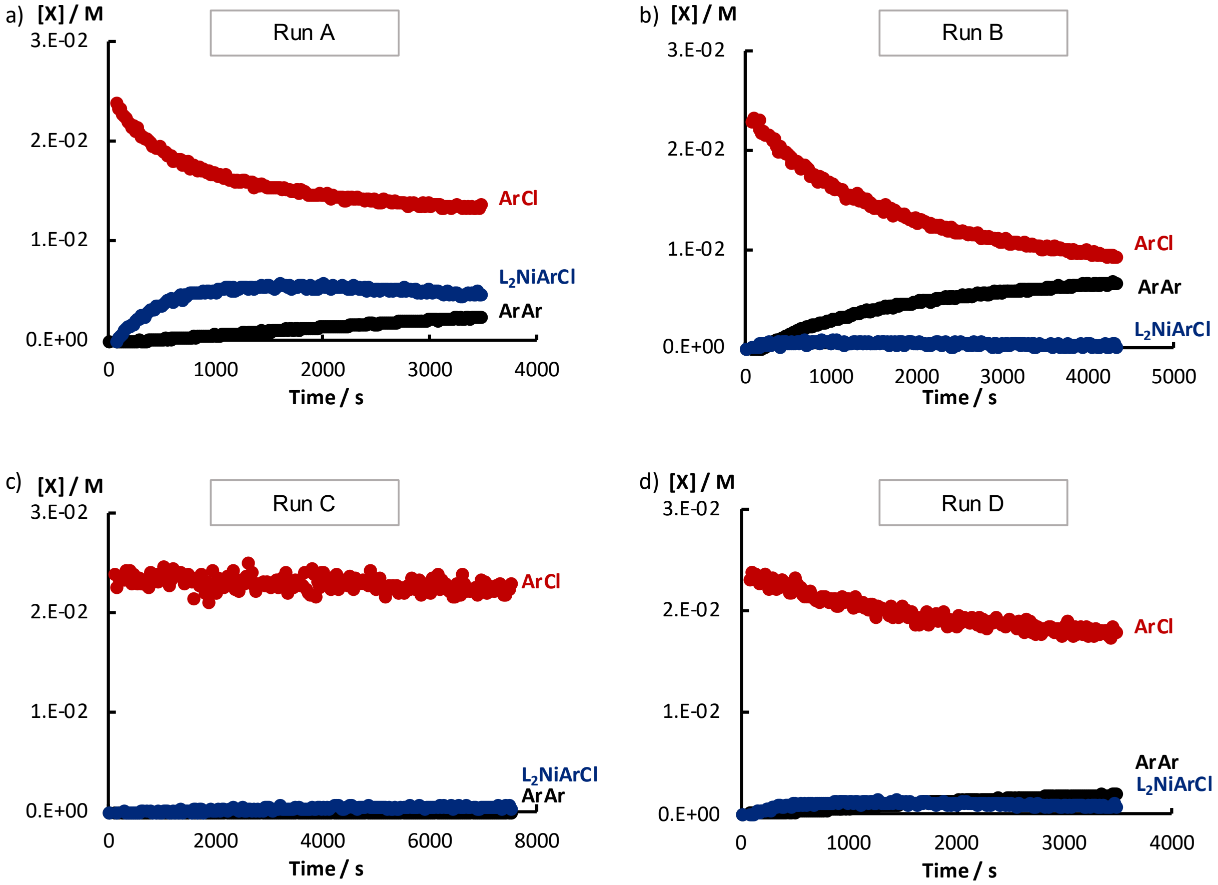

*Figure S41: a) Concentration vs time plot of the reaction of $[Ni^0(PAr_3)_n(DMF)_m]$ (prepared in situ from $Ni(COD)_2$ + PAr_3) with ArCl (**1**). The reaction slows significantly after one turnover. The intermediate $[L_2NiArCl]$ (**6**) is generated in significant proportions. b) Concentration vs time plot of the reaction of $[Ni^0(PAr_3)_n(DMF)_m]$ with ArCl (**1**) in the presence of $ZnCl_2$. The reaction undergoes semicatalytic turnover, i.e. more ArCl (**1**) is consumed than $[Ni(COD)_2]$ initially added. The generation of ArAr (**2**) is faster and there is significantly less accumulation of the $L_2NiArCl$ (**6**) intermediate. c) Concentration vs time plot of the reaction of $[(PAr_3)_3Ni^ICl]$ (prepared in situ by comproportionation of $[Ni^0(PAr_3)_n(DMF)_m]$ and $[NiCl_2(glyme)]$) with ArCl (**1**). There is very slow ArCl (**1**) consumption and $L_2NiArCl$ (**6**) generation. d) Concentration vs time plot of the reaction $[(PAr_3)_3Ni^ICl]$ with ArCl (**1**) in the presence of $ZnCl_2$. ArCl (**1**) is consumed, and $L_2NiArCl$ (**6**) and Ar-Ar (**2**) generated, consistent with $ZnCl_2$ indirectly biasing the disproportionation of $[(PAr_3)_3Ni^ICl]$, by chloride abstraction from $[(PAr_3)_2Ni^{II}Cl_2]$.*

S5.1.8 Inhibition of the $ZnCl_2$-accelerated Homocoupling of $L_2NiArCl$ (6) by $[(PAr_3)_2NiCl_2]$ at raised PAr_3 concentration.

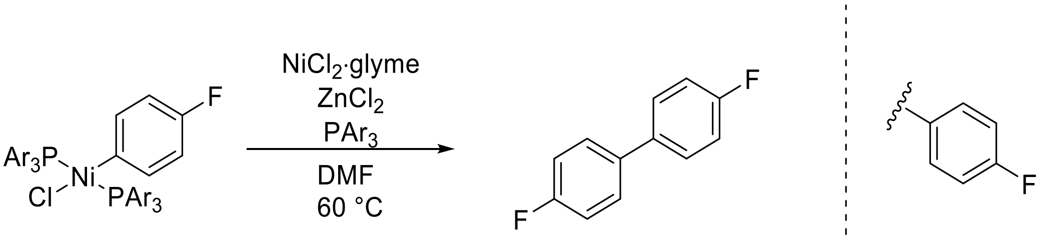

6 **2** **Ar**

*Scheme S24: Stoichiometric $ZnCl_2$-accelerated homocoupling of $L_2NiArCl$ (**6**) with added $[NiCl_2(glyme)]$.*

The Ni and $ZnCl_2$ stock solutions were prepared as described in the standard procedure.

S5.1.8.1 Ni stock solution (see standard procedure)

$L_2NiArCl$ (**6**, 26.3 mg, 32.0 µmol), PAr_3 (419.1 mg, 1.32 mmol) and 1-fluoronaphthalene (IS) (2 µL, 15.5 µmol) in DMF (2 mL).

S5.1.8.2 $ZnCl_2$ stock solution (see standard procedure)

$ZnCl_2$ (48.1 mg, 353 µmol) in DMF (1 mL).

S5.1.8.3 $NiCl_2$·glyme stock solution

A stock solution of $NiCl_2$·glyme (36.9 mg, 168 µmol) in DMF was made in a volumetric flask (1 mL).

S5.1.8.4 Shimming and Tuning of the spectrometer

The spectrometer was shimmed to a sample containing Ni stock solution (500 µL) and DMF (50 µL) and then tuned to ^{19}F.

S5.1.8.5 Reaction Mixture

The reaction mixtures were prepared according to the standard procedure. (500 µL Ni stock solution, 50 µL $ZnCl_2$) The volumes of DMF (0-50 µL) and $NiCl_2$·glyme stock solution (0-50 µL) were varied to afford the concentrations in Table S34. The total volume of each sample was 600 µL.

Table S34: Concentration of reactants in the $ZnCl_2$-accelerated homocoupling of $L_2NiArCl$ (6) in the presence of $[(PAr_3)_2NiCl_2]$.

All concentrations are in mM

Run	$[6]_0$	$[IS]_0$	$[PAr_3]_0$	$[ZnCl_2]_0$	$[NiCl_2]_0$
A	8.7	6.5	552.0	29.4	0.0
B	10.5	6.5	552.0	29.4	7.0
C	10.7	6.5	552.0	29.4	14.0

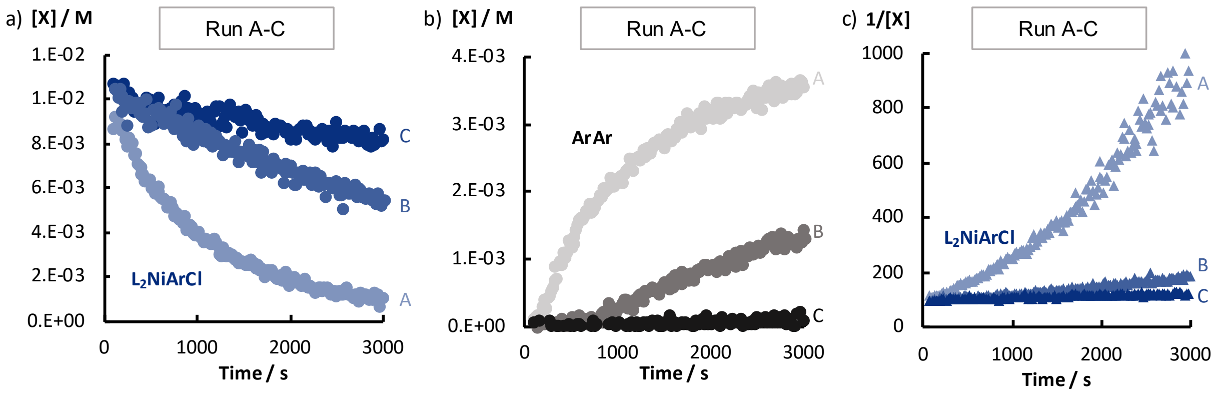

*Figure S42: a) Concentration vs time plot of the $ZnCl_2$-accelerated stoichiometric homocoupling of L_2NiArCl (**6**), at high phosphine concentration, in the absence (A) and presence of [$(PAr_3)_2NiCl_2$] (B, C). The rate decreases with increasing concentration of [$(PAr_3)_2NiCl_2$] b) Concentration of ArAr (**2**) vs time plot for the same reactions (A,B,C). c) Reciprocal concentration plot of L_2NiArCl (**6**), run A has a curved kinetic profile indicating that the reaction is not second order in L_2NiArCl (**6**). With increasing [$(PAr_3)_2NiCl_2$] concentration the profiles become more linear indicating progressive transition to second order kinetics.*

S5.1.9 Direct Homocoupling of $L_2NiArCl$ (6) in the Presence of $[(PAr_3)_2NiCl_2]$.

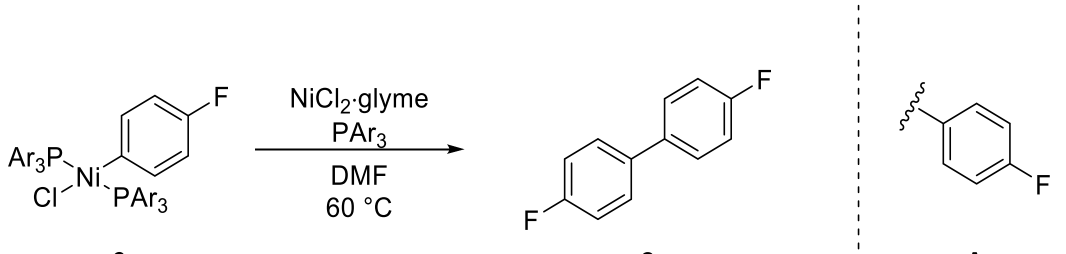

*Scheme S25: Reaction scheme of the stoichiometric reaction of $L_2NiArCl$ (**6**) with $NiCl_2$·glyme without $ZnCl_2$.*

The Ni stock solution was prepared as described in the standard procedure.

S5.1.9.1 Ni stock solution (see standard procedure)

$L_2NiArCl$ (**6**, 26.9 mg, 32.7 µmol), PAr_3 (130 mg, 409 µmol) and 1-fluoronaphthalene (IS) (2 µL, 15.5 µmol) in DMF (2 mL)

S5.1.9.2 $NiCl_2$·glyme stock solution

A stock solution of $NiCl_2$·glyme (38.9 mg, 177 µmol) in DMF was made in a volumetric flask (1 mL).

S5.1.9.3 Shimming and Tuning of the spectrometer

The spectrometer was shimmed to a sample containing Ni stock solution (500 µL), DMF (50 µL) and $NiCl_2$·glyme stock solution (50 µL) and then tuned to ^{19}F.

S5.1.9.4 Reaction Mixture

The reaction mixtures were prepared according to the standard procedure. (500 µL Ni stock solution) The volumes of DMF (50-100 µL) and $NiCl_2$·glyme stock solution (0-50 µL) were varied to generate the concentrations shown in Table S35. The total volume of each sample was 600 µL.

Table S35: Concentration of reactants in the direct homocoupling of $L_2NiArCl$ (6) in the presence of $[(PAr_3)_2NiCl_2]$.

All concentrations are in mM

Run	$[6]_0$	$[IS]_0$	$[PAr_3]_0$	$[NiCl_2(glyme)]_0$
A	9.7	6.5	170.6	0.0
B	8.8	6.5	170.6	7.4
C	8.5	6.5	170.6	14.8

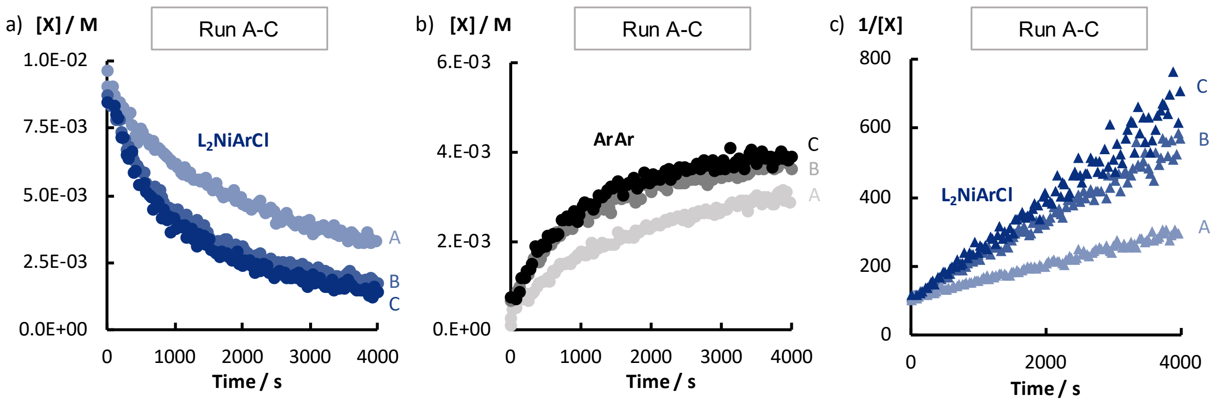

*Figure S43: a) Concentration vs time plot of the stoichiometric reaction of $L_2NiArCl$ (**6**) in the presence of $[(PAr_3)_2NiCl_2]$. The initial rate increases as the $[(PAr_3)_2NiCl_2]$ concentration is increased. The increase in rate is greater than that predicted by the reduction in concentration of free PAr_3 caused by the in situ generation of $[(PAr_3)_2NiCl_2]$ from $[NiCl_2(glyme)]$. The $[(PAr_3)_2NiCl_2]$ may possibly act as a chloride anion source by generation of $[NiCl(DMF)_5]^+$ b) Concentration of ArAr (**2**) vs time plots of the same reactions. c) Reciprocal concentration plots of $L_2NiArCl$ (**6**) vs time. All three runs are indicative of second order kinetics in $L_2NiArCl$ (**6**).*

S5.1.10 Inhibition of the $ZnCl_2$-accelerated Homocoupling of $L_2NiArCl$ (6) by $[(PAr_3)_2NiCl_2]$

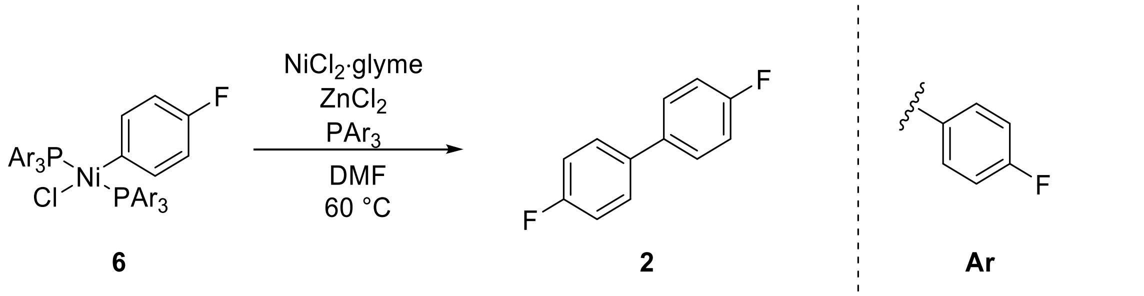

*Scheme S26: Stoichiometric $ZnCl_2$-accelerated homocoupling of $L_2NiArCl$ (**6**) with added $[NiCl_2(glyme)]$.*

S5.1.10.1 Ni stock solution (see standard procedure)
For run A:
$L_2NiArCl$ (**6**, 24.4 mg, 29.7 µmol), PAr_3 (123 mg, 389 µmol) and 1-fluoronaphthalene (IS) (2 µL, 15.5 µmol) in DMF (2mL).

For runs B-G:
$L_2NiArCl$ (**6**, 49.3 mg, 60.0 µmol), PAr_3 (246 mg, 777 µmol) and 1-fluoronaphthalene (IS) (5 µL, 38.7 µmol) in DMF (4 mL).

S5.1.10.2 $NiCl_2$·glyme stock solution
For run A:
A stock solution of $NiCl_2$·glyme (92.2 mg, 420 µmol) in DMF was made in a volumetric flask (2 mL).

For B-G:
A stock solution of $NiCl_2$·glyme (94.7 mg, 431 µmol) in DMF was made in a volumetric flask (2 mL).

S5.1.10.3 $ZnCl_2$ stock solution (see standard procedure)
For run A:
$ZnCl_2$ (50.9 mg, 374 µmol) in DMF (1 mL).

For runs B-G:
$ZnCl_2$ (100.3 mg, 736 µmol) in DMF (2 mL).

S5.1.10.4 Shimming and Tuning of the spectrometer
For run A:
The spectrometer was shimmed to a sample containing Ni stock solution (500 µL), $ZnCl_2$ stock solution (50 µL) and $NiCl_2$·glyme stock solution (50 µL) at 60 °C and then tuned to ^{19}F.

For runs B-G:
The spectrometer was shimmed to a sample containing Ni stock solution (500 µL) and DMF (50 µL) at 60 °C and then tuned to ^{19}F.

S5.1.10.5 Reaction Mixture
The reaction mixtures were prepared according to the standard procedure (Ni stock solution (500 µL) and $ZnCl_2$ stock solution (50 µL)). The volumes of DMF (0-50 µL) and $NiCl_2$·glyme stock solution (0-50 µL) were varied to generate the concentrations shown in Table S36. The total volume of each sample was 600 µL.

Table S36: Concentration of reactants in the $ZnCl_2$-accelerated homocoupling of $L_2NiArCl$ (6) in the presence of $[(PAr_3)_2NiCl_2]$.

All concentrations are in mM

Run	$[6]_0$	$[IS]_0$	$[PAr_3]_0$	$[ZnCl_2]_0$	$[NiCl_2(glyme)]_0$
A	9.6	6.5	162.2	31.1	17.5
B	9.2	8.1	161.9	30.7	18.0
C	9.6	8.1	161.9	30.7	14.4
D	15.2	8.1	161.9	30.7	10.8
E	9.4	8.1	161.9	30.7	7.2
F	13.0	8.1	161.9	30.7	3.6
G	7.6	8.1	161.9	30.7	0.0

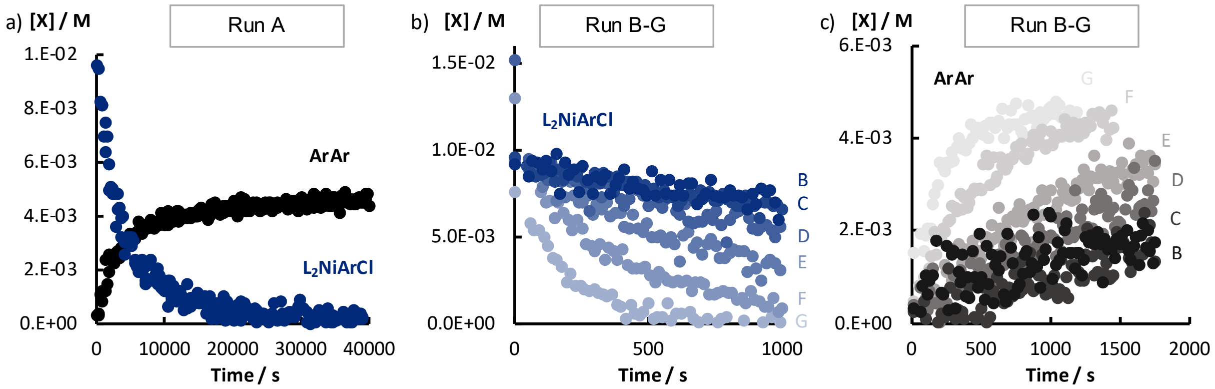

*Figure S44: a) Kinetic profile of the $ZnCl_2$-accelerated stoichiometric reaction of $L_2NiArCl$ (**6**) with added $[NiCl_2(glyme)]$. b) Concentration vs time plot for runs B-G. The rates decrease with increasing $NiCl_2$·glyme concentration. c) Concentration of ArAr (**2**) vs time plot of runs B-G. The rates decrease with increasing $NiCl_2$·glyme concentration through the acceleration of reverse transmetallation (Zn-to-Ni; k_{-TM1}) further modulated by the interaction of $[(PAr_3)_2NiCl_2]$ with $[ZnCl_2]$.*

S5.1.11 Reaction of $L_2NiArCl$ (6) and $[(PAr_3)_2NiCl_2]$ (17) with ZnArCl (16) and $ZnAr_2$ (19)

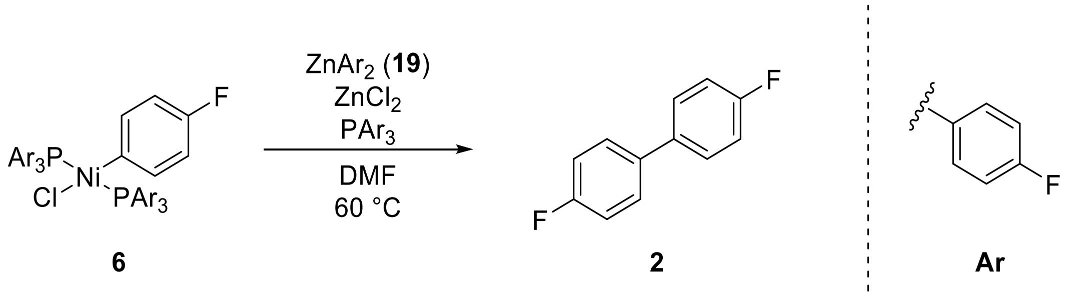

*Scheme S27: Reaction Scheme of the stoichiometric reaction of $L_2NiArCl$ (**6**), $NiCl_2$·glyme, $ZnAr_2$ (**19**) and $ZnCl_2$.*

S5.1.11.1 Ar-Ni stock solution (see standard procedure)
$L_2NiArCl$ (**6**, 26.6 mg, 32.4 µmol), PAr_3 (123.7 mg, 391 µmol) and 1-fluoronaphthalene (IS) (7.8 mg, 53.4 µmol) in DMF (2 mL).

S5.1.11.2 $[(PAr_3)_2NiCl_2]$ stock solution
A stock solution of $NiCl_2$·glyme (8 mg, 36.4 µmol), PAr_3 (127 mg, 401 µmol) and 1-fluoronaphthalene (IS) (11.4 mg, 78.0 µmol) in DMF was prepared in a volumetric flask (2 mL).

*S5.1.11.3 $ZnAr_2$ (**19**) stock solution*
A stock solution of $ZnAr_2$ (**19**, 21.1 mg, 82.6 µmol) in DMF was prepared in a volumetric flask (1 mL).

*S5.1.11.4 ZnArCl (**16**) stock solution*
A stock solution of $ZnCl_2$ (7.6 mg, 52.1 µmol) and $ZnAr_2$ (**19**, 0.5 mL $ZnAr_2$ stock solution) was prepared in DMF (0.5 mL) (total volume 1 mL).

S5.1.11.5 Shimming and Tuning of the spectrometer
For runs A, B, D & E:
The NMR was shimmed to a sample containing $L_2NiArCl$ (**6**) stock solution (500 µL) at 300 K. The NMR was then tuned to ^{19}F.

For run C:
The NMR was shimmed to a sample containing $[(PAr_3)_2NiCl_2]$ solution (500 µL) at 300 K. The spectrometer was then tuned to ^{19}F. The shimming was poorer than in runs A, B, D, E.

S5.1.11.6 Reaction Mixture
For runs A & B:
The reaction mixtures were prepared using Ni stocks solution (500 µL) and either ZnArCl (**16**, A) or $ZnAr_2$ (**19**, B) stock solutions (100 µL). The reaction was monitored at 300 K.

For runs C-E:
The reaction mixtures were prepared using $NiCl_2$·glyme stocks solution (500 µL) and either ZnArCl (**16**, A) or $ZnAr_2$ (**19**, B) stock solutions (100 µL). Reactions C and D were monitored at 300 K, reaction E was monitored at 333 K.

*Table S37: Concentrations of reactants in reactions of $[(PAr_3)_2NiCl_2]$ (17) and $L_2NiArCl$ (**6**) with ZnArCl (**16**) and $ZnAr_2$ (**19**).*

All concentrations are in mM

Run	$[L_2NiArCl]$ $[6]_0$	$[IS]_0$	$[PAr_3]_0$	ZnArCl $[16]_0$	$ZnAr_2$ $[19]_0$	$[NiCl_2]_0$
A	8.31	22.2	162.9	13.8	0.0	0.0
B	10.5	22.2	162.9	0.0	15.0	0.0
C	0.0	32.5	167.3	12.0	0.0	15.2
D	0.0	32.5	167.3	0.0	13.8	15.2
E	0.0	32.5	167.3	0.0	13.8	15.2

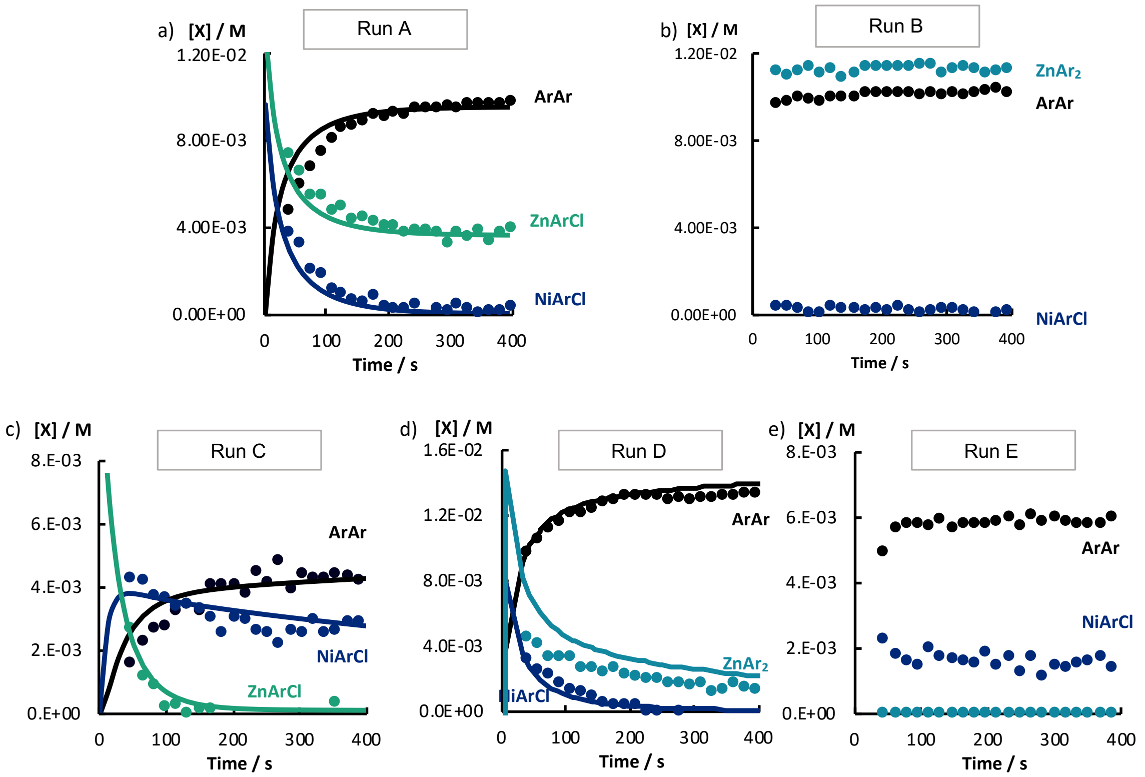

*Figure S45: In situ ^{19}F NMR spectroscopic analyses of the stoichiometric reactions of a) $L_2NiArCl$ (**6**) with ZnArCl (**16**) at 300 K. ZnArCl (**16**), supporting the hypothesis that ZnArCl (**16**) can be an intermediate in the catalytic homocoupling reaction at 333 K. b) $L_2NiArCl$ (**6**) with $ZnAr_2$ (**19**). The reaction is almost completed after mixing: $ZnAr_2$ (**19**) reacts faster than ZnArCl (**16**). c) $[(PAr_3)_2NiCl_2]$ (**17**) with ZnArCl (**16**), to generate $L_2NiArCl$ (**6**) and thus Ar-Ar (**2**). d) $[(PAr_3)_2NiCl_2]$ (**17**) with $ZnAr_2$ (**19**) at 300 e) $[(PAr_3)_2NiCl_2]$ (**17**) with $ZnAr_2$ (**19**) at 333 K. Lines through data in Runs A, C and D are based on the following simple model:*

$$[\mathrm{L_2NiCl_2}]\ (\mathbf{17})\ +\ \mathrm{Ar_2Zn}\ (\mathbf{19}) \rightarrow [\mathrm{L_2Ni(Ar)Cl}]\ (\mathbf{6})\ +\ \mathrm{ArZnCl}\ (\mathbf{18});\ 1.3\times10^4\ \mathrm{M^{-1}s^{-1}}$$

$$[\mathrm{L_2Ni(Ar)Cl}](\mathbf{6}) + \mathrm{Ar_2Zn}\ (\mathbf{19}) \rightarrow \mathrm{Ar{-}Ar}\ (\mathbf{2}) + \mathrm{ArZnCl}\ (\mathbf{18})\ +\ [\mathrm{Ni^0}];\ 0.7\times10^4\ \mathrm{M^{-1}s^{-1}}$$

$$[\mathrm{L_2NiCl_2}](\mathbf{17}) + \mathrm{ArZnCl}\ (\mathbf{18}) \rightarrow [\mathrm{L_2Ni(Ar)Cl}](\mathbf{6}) + \mathrm{ZnCl_2}\ ;\ \ 2.4\ \mathrm{M^{-1}s^{-1}}$$

$$[\mathrm{L_2Ni(Ar)Cl}](\mathbf{6}) + \mathrm{ZnCl_2}\ \rightarrow [\mathrm{L_2NiCl_2}](\mathbf{17}) + \mathrm{ArZnCl}\ (\mathbf{18});\ \ 7\times10^{-2}\ \mathrm{M^{-1}s^{-1}}$$

$$[\mathrm{L_2Ni(Ar)Cl}](\mathbf{6}) + \mathrm{ArZnCl}\ (\mathbf{18}) \rightarrow \mathrm{Ar{-}Ar}\ (\mathbf{2}) + \mathrm{ZnCl_2}\ +\ [\mathrm{Ni^0}];\ 3.5\ \mathrm{M^{-1}s^{-1}}$$

$$[\mathrm{L_2NiCl_2}]\ (\mathbf{17})\ +\ [\mathrm{L}_n\mathrm{Ni^0}] \rightarrow 2\ [\mathrm{L_3NiCl}]\ (\mathbf{7});\ K\ \geq\ 10;\ >60\ \mathrm{M^{-1}s^{-1}}$$

S5.1.12 Homocoupling of L_2NiArCl (6) in the presence of NaI

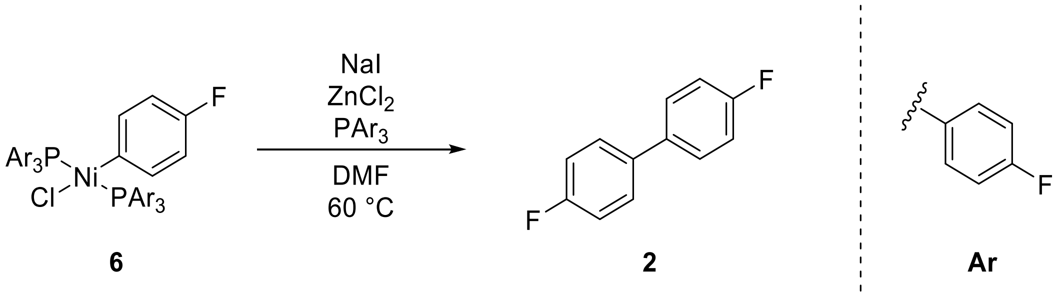

*Scheme S28: Stoichiometric homocoupling of L_2NiArCl (**6**) in the presence of NaI, with and without $ZnCl_2$.*

S5.1.12.1 Ni stock solution (see standard procedure)

L_2NiArCl (**6**, 64.1 mg, 78.0 µmol), PAr_3 (299 mg, 946 µmol) and 1-fluoronaphthalene (IS) (6 µL, 46.5 µmol) in DMF (5 mL).

S5.1.12.2 $ZnCl_2$ stock solution (see standard procedure)

$ZnCl_2$ (103 mg, 759 µmol) in DMF (2 mL).

S5.1.12.3 NaI stock solution

A stock solution of NaI (39.5 mg, 264 µmol) was prepared in DMF in a volumetric flask (1 mL).

S5.1.12.4 Shimming and Tuning of the spectrometer

The spectrometer was shimmed and tuned to a sample containing Ni stock solution (500 µL) at 60 °C and then tuned to ^{19}F.

S5.1.12.5 Reaction Mixture

For run A:
Ni stock solution (500 µL), DMF (50 µL) and NaI stock solution (50 µL) were added to an NMR tube.

For run B:
Ni stock solution (500 µL), $ZnCl_2$ stock solution (50 µL) and NaI stock solution (50 µL) were added to an NMR tube.

Table S38: Concentration of all reagents in the stoichiometric reactions with NaI as an additive.

All concentrations are in mM

Run	$[6]_0$	$[IS]_0$	$[PAr_3]_0$	$[ZnCl_2]_0$	$[NaI]_0$
A	9.1	7.7	157.7	0.0	22.0
B	9.3	7.7	157.7	31.6	22.0

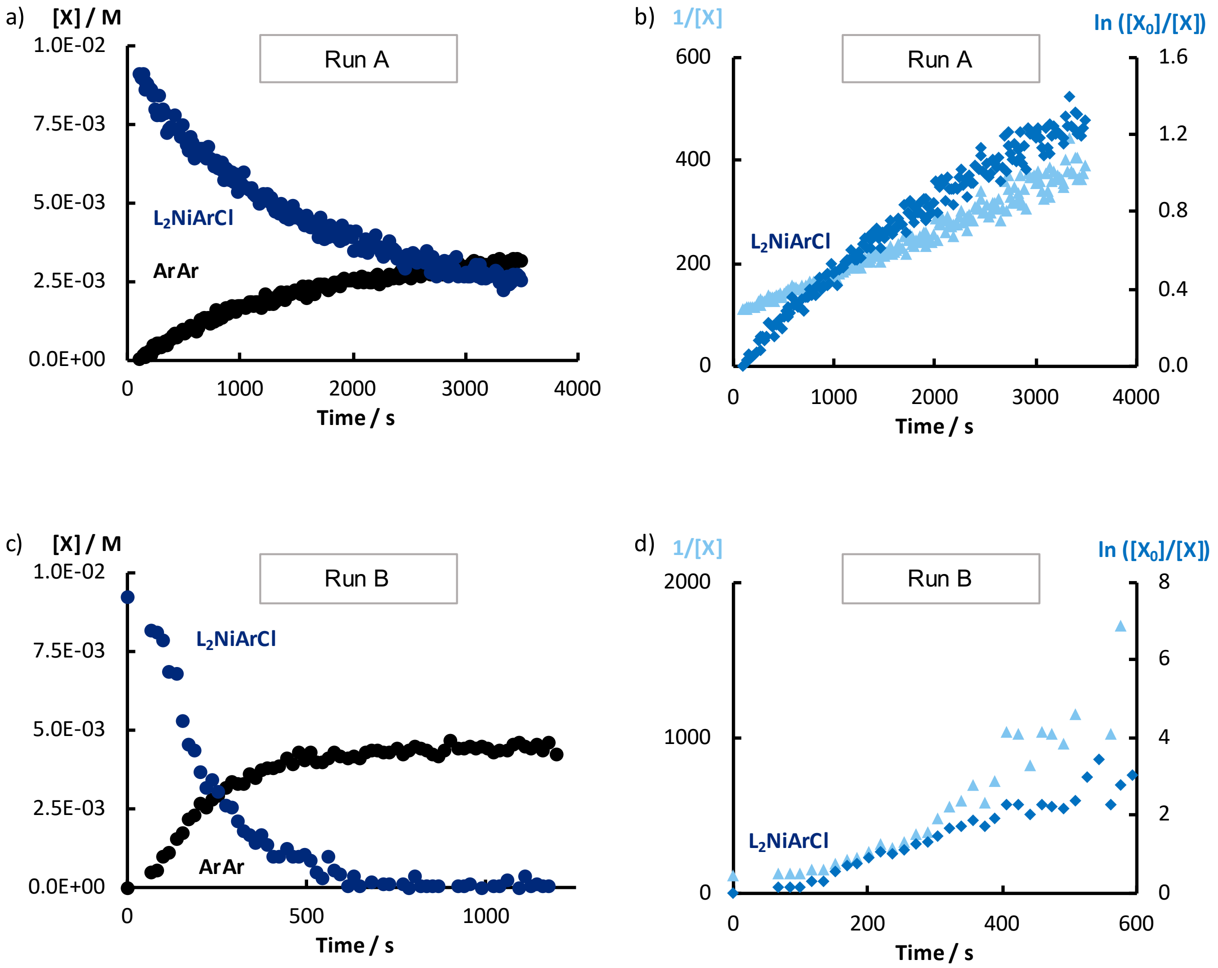

*Figure S46: a) Concentration vs time plot of stoichiometric homocoupling of $L_2NiArCl$ (**6**) in the presence of NaI. b) The process proceeds with second order kinetic dependency on $L_2NiArCl$ (**6**). The rate is not accelerated by NaI. c) Concentration vs time plot of the $ZnCl_2$-accelerated homocoupling of $L_2NiArCl$ (**6**) in the presence of NaI. d) The process proceeds with approximately pseudo first-order dependency on $L_2NiArCl$ (**6**).*

S5.1.13 $ZnCl_2$ and Cl^- accelerated Homocoupling of $L_2NiArCl$ (6) in the presence of H_2O

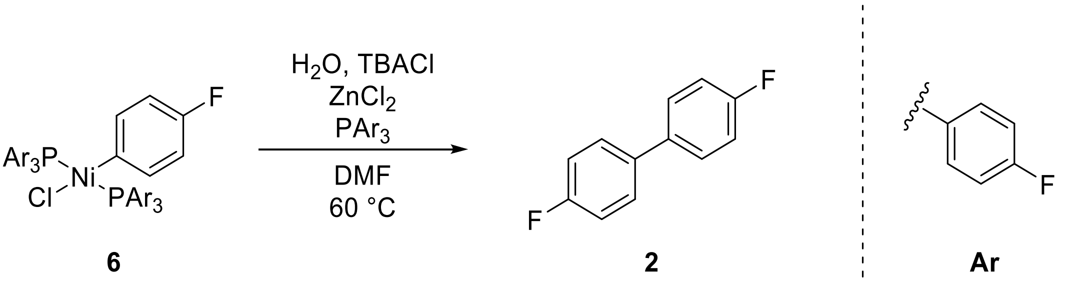

Scheme S29: Reaction scheme of the stoichiometric reaction of $L_2NiArCl$ (6) with H_2O, $ZnCl_2$ and TBACl.

S5.1.13.1 Ni stock solution (see standard procedure)
$L_2NiArCl$ (**6**, 64.1 mg, 78.0 µmol), PAr_3 (299.4 mg, 946 µmol) and 1-fluoronaphthalene (IS) (6 µL, 46.5 µmol) in DMF (5 mL).

S5.1.13.2 $ZnCl_2$ stock solution (see standard procedure)
$ZnCl_2$ (103.4 mg, 759 µmol) in DMF (2 mL).

S5.1.13.3 H_2O stock solution 1
A stock solution of H_2O (5 µL, 277 µmol) was prepared in DMF in a volumetric flask (1 mL).

S5.1.13.4 H_2O stock solution 2
A stock solution of H_2O (20 µL, 1.1 mmol) was prepared in DMF in a volumetric flask (1 mL).

S5.1.13.5 TBACl stock solution
A stock solution of TBACl (76.3 mg, 275 µmol) was prepared in DMF in a volumetric flask (1 mL).

S5.1.13.6 Shimming and Tuning of the spectrometer
The spectrometer was shimmed and tuned to a sample containing Ni stock solution (500 µL) at 60 °C and then tuned to ^{19}F.

S5.1.13.7 Reaction Mixture
For runs A & B:
Ni stock solution (500 µL), DMF (50 µL) and H_2O stock solution (50 µL; A sol 1, B sol 2) were added to an NMR tube.

For runs C & D:
Ni stock solution (500 µL), $ZnCl_2$ stock solution (50 µL) and H_2O stock solution (50 µL; A sol 1, B sol 2) were added to an NMR tube.

For run E:
Ni stock solution (500 µL), TBACl (50 µL) and H_2O stock solution 2 (50 µL) were added to an NMR tube.

*Table S39: Concentration of reactants in the stoichiometric homocoupling of ArNi (**6**) with H_2O as an additive.*

All concentrations are in mM

Run	$[6]_0$	$[IS]_0$	$[PAr_3]_0$	$[ZnCl_2]_0$	$[H_2O]_0$	[TBACl]
A	9.1	7.8	157.7	0.0	23.1	0.0
B	9.1	7.8	157.7	0.0	92.3	0.0
C	8.0	7.8	157.7	31.6	23.1	0.0
D	7.9	7.8	157.7	31.6	92.3	0.0
E	8.5	7.8	157.7	0.0	92.3	22.9
F	8.6	7.8	157.7	31.6[i]	92.3	0.0

i) $ZnCl_2$ was added to the reaction mixture after around 1350 s (22 min 30 s)

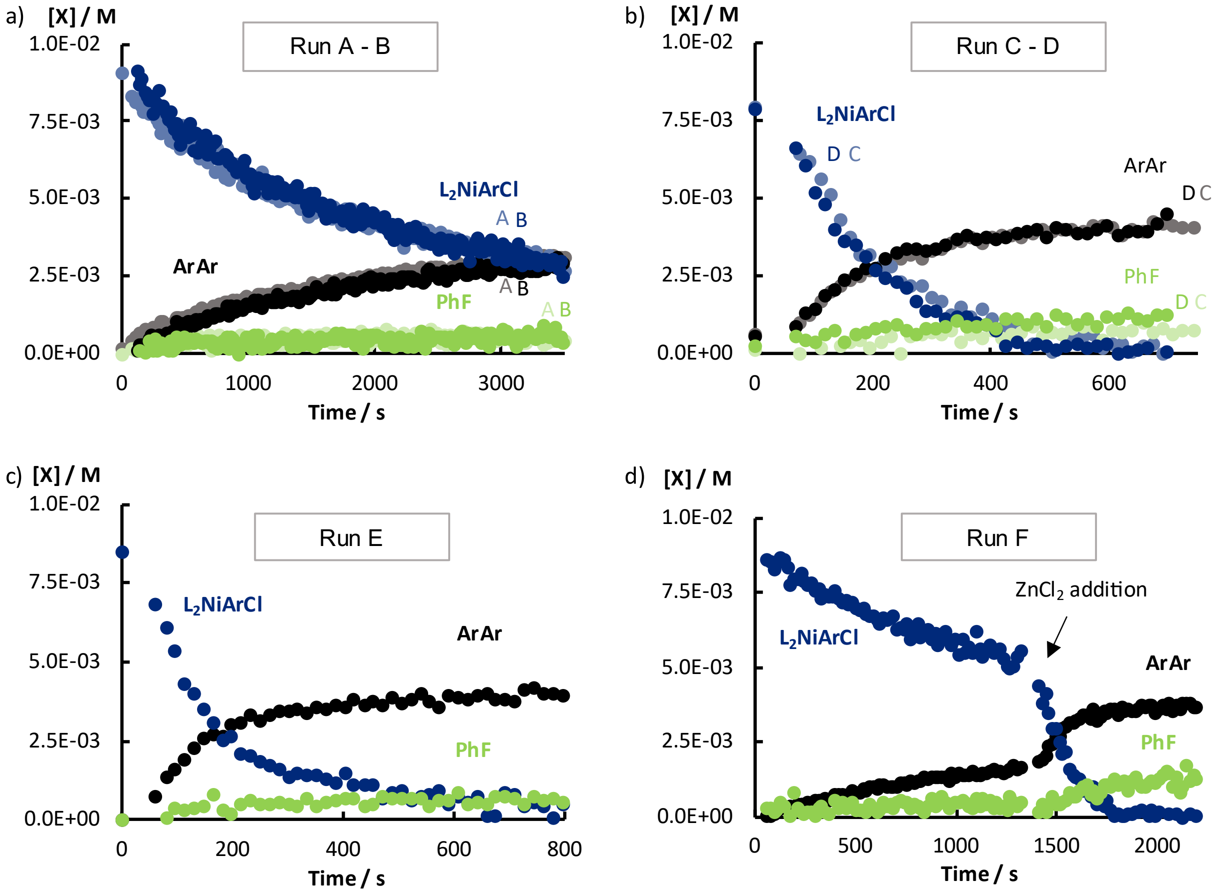

*Figure S47: a) Concentration vs time plot of two stoichiometric reactions of $L_2NiArCl$ (**6**) with different water concentrations. The quantity of PhF (**3**) produced does not significantly differ. b) Concentration vs time plot of two $ZnCl_2$-accelerated stoichiometric homocouplings of $L_2NiArCl$ (**6**) at two different water concentrations. The quantity of PhF (**3**) generated is higher in the reaction with higher concentrations of water, but not directly proportional to the water concentration. c) Concentration vs time plot of the chloride-accelerated stoichiometric homocoupling of $L_2NiArCl$ (**6**) with added water. The quantity of PhF (**3**) generated is marginally increased by the presence of water. d) Concentration vs time plot of the stoichiometric homocoupling of $L_2NiArCl$ (6) with water in which $ZnCl_2$ is added during the reaction. The reaction rate and formation of PhF (**3**) increased upon the addition of $ZnCl_2$. The reactions with $ZnCl_2$ present form more PhF (**3**) than the reactions without $ZnCl_2$ present.*

S5.1.14 Effect of added NaBAr^F on the Stoichiometric Homocoupling of $L_2NiArCl$ (6)

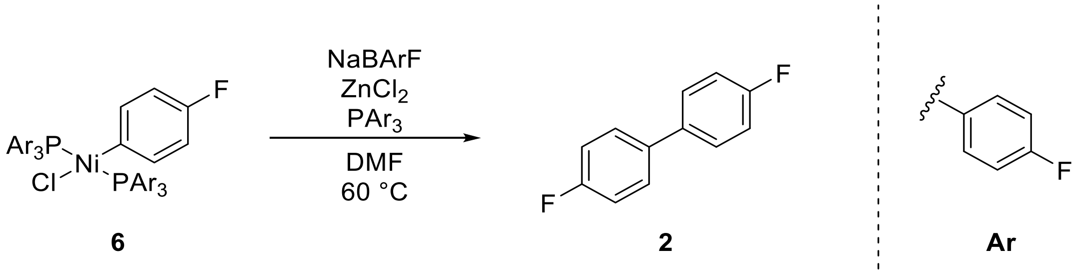

Scheme S30: $ZnCl_2$-accelarted stoichiometric homocoupling of $L_2NiArCl$ (6) with added NaBAr^F.

S5.1.14.1 Ni stock solution (see standard procedure)

For runs A & B:

$L_2NiArCl$ (**6**, 25.11 mg, 30.6 µmol), PAr_3 (127.5 mg, 403 µmol) and 1-fluoronaphthalene (IS) (2 µL, 15.5 µmol) in DMF (2 mL).

For runs C & D:

$L_2NiArCl$ (**6**, 24.8 mg, 30.1 µmol), PAr_3 (123 mg, 388 µmol) and 1-fluoronaphthalene (IS) (2 µL, 15.5 µmol) in DMF (2 mL).

S5.1.14.2 $ZnCl_2$ stock solution (see standard procedure)

$ZnCl_2$ (103.8 mg, 762 µmol) in DMF (2 mL).

S5.1.14.3 NaBAr^F stock solution

A stock solution of NaBAr^F (442, 498 µmol) was prepared in DMF (2 mL).

S5.1.14.4 Shimming and Tuning of the Spectrometer

The spectrometer was shimmed to a sample containing $L_2NiArCl$ (**6**, 500 µL) at 60 °C and then tuned to ^{19}F.

S5.1.14.5 Reaction Mixture

For run A:

Ni stock solution (500 µL) was mixed with DMF (100 µL).

For run B:

Ni stock solution (500 µL) was mixed with DMF (50 µL) and NaBArF stock solution (50 µL).

For run C:

Ni stock solution (500 µL) was mixed with DMF (50 µL) and $ZnCl_2$ stock solution (50 µL).

For run D:

Ni stock solution (500 µL) was mixed with NaBArF stock solution (50 µL) and $ZnCl_2$ stock solution (50 µL).

*Table S40: Concentration of reactants in the stoichiometric homocoupling of ArNi (**6**) with NaBAr^F as an additive.*

All concentrations are in mM

Run	$[6]_0$	$[IS]_0$	$[PAr_3]_0$	$[ZnCl_2]_0$	[NaBArF]
A	8.3	6.5	127.5	0.0	0.0
B	8.3	6.5	127.5	0.0	20.8
C	7.9	6.5	161.6	31.7	0.0
D	7.7	6.5	161.6	31.7	20.8

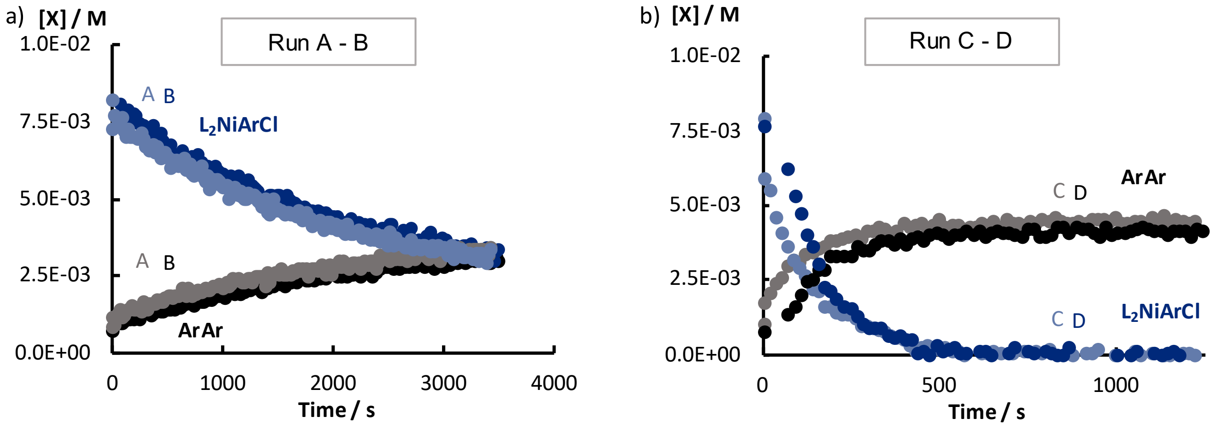

*Figure S48: a) Concentration vs time plot of two direct homocoupling reactions of L_2NiArCl (**6**): the presence of NaBAr^F has no effect on the kinetic profile. b) Concentration vs time plot of two $ZnCl_2$-accelerated homocoupling reactions of L_2NiArCl (**6**). The presence of NaBAr^F causes a slight induction period, after which the rate is similar to the reaction in the absence of NaBAr^F.*

S5.1.15 Cl⁻ accelerated Homocoupling of $L_2NiArCl$ (6)

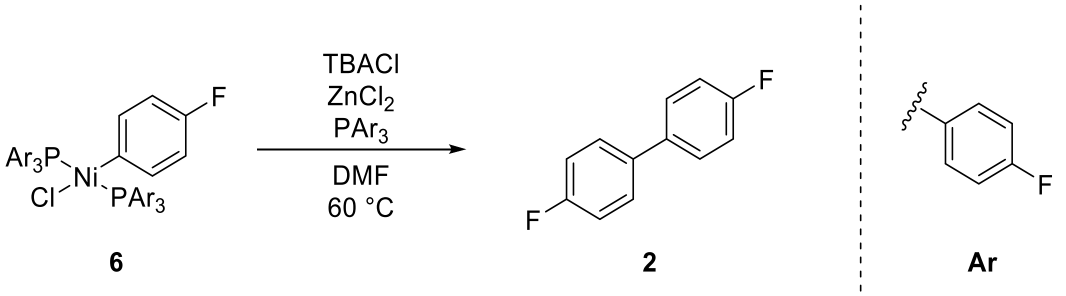

Scheme S31: Reaction scheme of the stoichiometric reaction of $L_2NiArCl$ (6) with TBACl in solution, with and without $ZnCl_2$

S5.1.15.1 Ni stock solution (see standard procedure)
For runs A, B, D, E, F &H:
$L_2NiArCl$ (**6**, 62.4 mg, 76.0 µmol), PAr_3 (300 mg, 950 µmol) and 1-fluoronaphthalene (IS) (6 µL, 46.5 µmol) in DMF (5 mL).

For run C:
$L_2NiArCl$ (**6**, 25.11 mg, 30.6 µmol), PAr_3 (128 mg, 403 µmol) and 1-fluoronaphthalene (IS) (2 µL, 15.5 µmol) in DMF (2 mL).

For run G:
$L_2NiArCl$ (**6**, 24.8 mg, 30.1 µmol), PAr_3 (123 mg, 388 µmol) and 1-fluoronaphthalene (IS) (2 µL, 15.5 µmol) in DMF (2 mL).

S5.1.15.2 $ZnCl_2$ stock solution (see standard procedure)
For run G:
$ZnCl_2$ (104 mg, 762 µmol) in DMF (2 mL).

For runs E, F & H:
$ZnCl_2$ (99.4 mg, 270 µmol) in DMF (2 mL).

S5.1.15.3 TBACl stock solution
For run C:
A stock solution of TBACl (139 mg, 502 µmol) was prepared in DMF (2 mL).

For run G:
A stock solution of TBACl (137 mg, 493 µmol) was prepared in DMF (2 mL).

For runs E, F & H:
A stock solution of TBACl (75.1 mg, 270 µmol) was prepared in DMF (1 mL).

S5.1.15.4 Shimming and Tuning of the Spectrometer
The spectrometer was shimmed to a sample containing $L_2NiArCl$ (**6**, 500 µL) at 60 °C and then tuned to ^{19}F.

S5.1.15.5 Reaction Mixture
For runs A-D:
Ni stock solution (500 µL) was mixed with DMF (50-90 µL) and TBACl stock solution (10-50 µL).

For runs A-D:
Ni stock solution (500 µL) was mixed with $ZnCl_2$ stock solution (50 µL), DMF (0-40 µL) and TBACl stock solution (10-50 µL).

Table S41: Concentration of reactants in the stoichiometric homocoupling reactions with TBACl as an additive.

All concentrations are in mM

Run	$[6]_0$	$[IS]_0$	$[PAr_3]_0$	$[ZnCl_2]_0$	$[TBACl]_0$
A	7.1	7.8	158.2	0.0	4.5
B	7.1	7.8	158.2	0.0	13.5
C	8.2	6.5	167.9	0.0	20.9
D	6.8	7.8	158.2	0.0	22.5
E	7.4	7.8	158.2	30.4	4.5
F	7.0	7.8	158.2	30.4	13.5
G	8.7	6.5	161.6	31.7	20.5
H	6.8	7.8	158.2	30.4	22.5

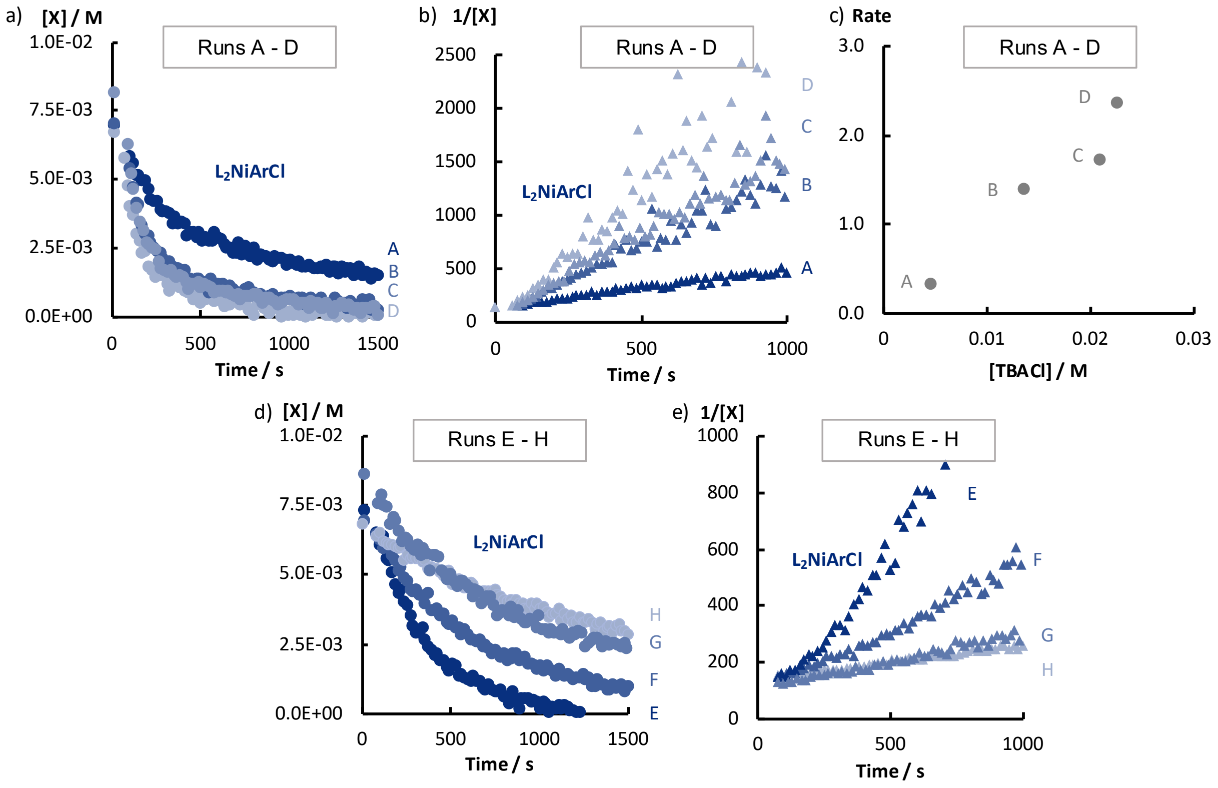

*Figure S49: a) Concentration vs time plot of the stoichiometric homocoupling of $L_2NiArCl$ (**6**) with TBACl in solution. b) Reciprocal concentration of $L_2NiArCl$ (**6**) vs time of runs A-D. The reaction rates were calculated by linear regression. c) The reaction rates plotted against the TBACl concentration, indicative of a first order dependency on TBACl. Run C has a higher PAr_3 concentration. d) Concentration vs time plot of the stoichiometric stoichiometric homocoupling of $L_2NiArCl$ (**6**) with TBACl and $ZnCl_2$ in solution. d) Reciprocal concentration plots of runs E-H. The reaction rate reduces and changes from first to second order on raising the TBACl concentration.*

S5.2 NMR Titrations and Equilibria

S5.2.1 Titration of $NiCl_2$·glyme and PAr_3 with $ZnCl_2$ in DMF

$$[\mathrm{L_2NiCl_2}](\mathbf{17}) + [\mathrm{ZnCl_2}] \overset{K_{M1}}{\rightleftarrows} [\mathrm{NiCl(DMF)_5}]^{+}[\mathrm{ZnCl_3(DMF)}]^{-}\ (\mathbf{20_{DMF}}) + 2 \times \mathrm{L}$$

Scheme S32: Titration of [$(PAr_3)_2NiCl_2$] with $ZnCl_2$ in DMF.

All stock solutions were prepared in the glove box under nitrogen atmosphere.

S5.2.1.1 $NiCl_2$·glyme stock solution
A stock solution of $NiCl_2$·glyme (46.7 mg, 213 µmol) was prepared in DMF in a volumetric flask (5 mL).

S5.2.1.2 $ZnCl_2$ stock solution
A stock solution of $ZnCl_2$ (49.9 mg, 366 µmol) in DMF was prepared in a volumetric flask (2 mL).

S5.2.1.3 PAr_3 stock solution
A stock solution of PAr_3 (203 mg, 642 µmol) in DMF was prepared (5 mL).

S5.2.1.4 Shimming and Tuning of the Spectrometer
The spectrometer was shimmed to sample A at 60 °C and then tuned to ^{31}P and ^{19}F.

S5.2.1.5 Reaction Mixtures
All samples were prepared with $NiCl_2$·glyme stock solution (250 µL), PAr_3 stock solution (170 µL for runs A-D and 250 µL for samples D-G) $ZnCl_2$ stock solution (0-100 µL) and DMF (0-180 µL).

The final concentrations are given in Table S42.

Before each measurement the samples were allowed to equilibrate at the temperature for 5 minutes.

^{31}P NMR spectra were acquired at 5 °C and ^{19}F NMR spectra were acquired 21 °C and 60 °C.

S5.2.1.6 Spectrometer Settings
Nucleus: ^{19}F
Pulse sequence: zg30
Number of scans: 8
Temperature: 333 K
Relaxation Delay: 10 s

Nucleus: ^{31}P
Pulse sequence: zg30
Number of scans: 1
Temperature: 294 K
Relaxation Delay: 6 s

*Table S42: Concentration of reactants in each sample for the titration of [$(PAr_3)_2NiCl_2$] (**17**) with $ZnCl_2$ in DMF.*

	**All concentrations are in mM**				
**Run**	**$[NiCl_2]_0$**	**$[PAr_3]_0$**	**$[ZnCl_2]_0$**	**^{19}F / ppm 294 K**	**^{19}F / ppm 333 K**
A	17.7	36.4	0.0	-108.89	-109.6
B	17.7	36.4	7.6	-110.61	-110.75
C	17.7	36.4	15.3	-111.9	-111.71
D	17.7	36.4	22.9	-112.44	-112.38
E	17.7	36.4	30.5	-112.46	-112.47
F	17.7	53.5	0.0	-109.44	-109.17
G	17.7	53.5	7.6	-110.89	-110.48
H	17.7	53.5	15.3	-111.96	-111.66
I	17.7	53.5	22.9	-112.43	-112.41
J	17.7	53.5	30.5	-112.45	-112.51

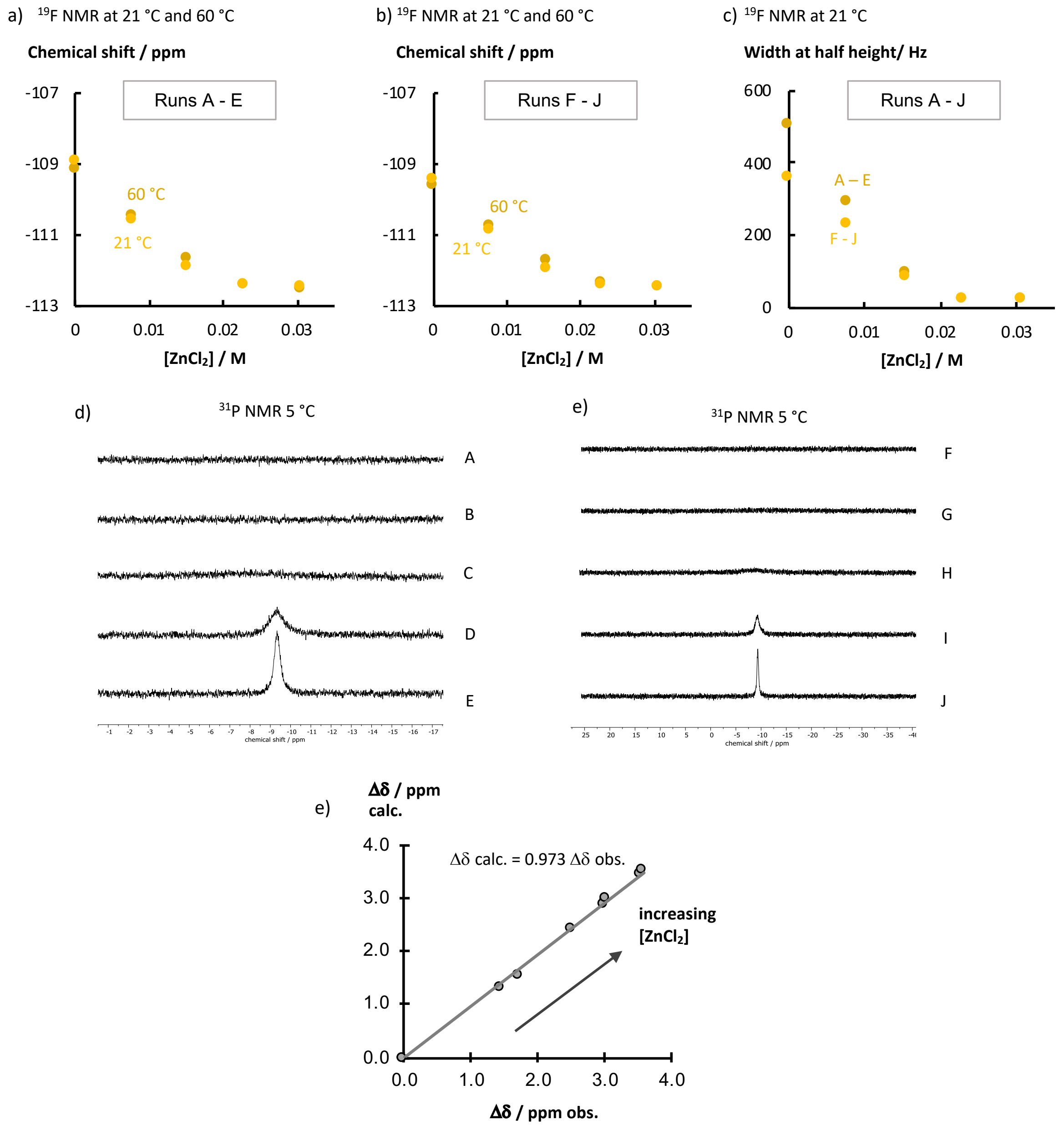

Figure S50: a) and b) Time-average ^{19}F NMR chemical shift of the free and coordinated phosphine ligand plotted against the $ZnCl_2$ concentration, at 21 °C and at 60 °C. The chemical shift asymptotes to that of the free ligand with increasing $ZnCl_2$ concentration. Runs A-E have a phosphine concentration of 36.4 mM and runs F-J had a phosphine concentration of 53.5 mM. There is no significant difference between the equilibria at 21 °C and 60 °C. c) Width at half height for the time-average ^{19}F NMR chemical signals in runs A-J at 21 °C. The signals became sharper with increasing $ZnCl_2$ concentration. d) and e) ^{31}P NMR time-average signals of the ligand. The signals at higher phosphine concentrations (53.5 mM) are sharper than the ones at lower phosphine concentrations (36.4 mM). The signals become sharper at higher $ZnCl_2$ concentrations. f) Combined fit of data from Runs A to J using simplified single step mono-cation equilibrium model (Scheme S32), with K_{M1} = 5 M (this does not include the concentration of DMF which is effectively constant). $\Delta\delta$ / ppm is defined as ({^{19}F ppm without $ZnCl_2$} – {^{19}F ppm with $ZnCl_2$}).

S5.2.2 Titration of $NiCl_2$·glyme and PAr_3 with $ZnCl_2$ in DMAc

$$[L_2NiCl_2](\mathbf{17}) + [ZnCl_2] \rightleftarrows [NiCl(DMAc)_n]^+[ZnCl_3(DMF)_m]^- (\mathbf{20_{DMAc}})$$

Scheme S33: Reaction scheme of the titration of $NiCl_2$·glyme with $ZnCl_2$ in DMAc.

All stock solutions were prepared in the glove box under nitrogen atmosphere.

S5.2.2.1 $NiCl_2$·glyme stock solution
A stock solution of $NiCl_2$·glyme (46.2 mg, 210 µmol) was prepared in DMAc in a volumetric flask (5 mL).

S5.2.2.2 $ZnCl_2$ stock solution
A stock solution of $ZnCl_2$ (51.0 mg, 374 µmol) in DMAc was prepared in a volumetric flask (2 mL).

S5.2.2.3 PAr_3 stock solution
A stock solution of PAr_3 (195 mg, 617 µmol) in DMAc was prepared (5 mL).

S5.2.2.4 Shimming and Tuning of the Spectrometer
The spectrometer was shimmed and tuned with each sample.

S5.2.2.5 Reaction Mixtures
All samples were prepared with $NiCl_2$·glyme stock solution (250 µL), PAr_3 stock solution (170 µL for runs A-D and 250 µL for samples D-G) $ZnCl_2$ stock solution (0-100 µL) and DMAc (0-180 µL).

The concentrations are given in Table S43Table S42.

Before each measurement the samples were allowed to equilibrate at the temperature for 5 minutes.

^{31}P NMR spectra were acquired at 5 °C and ^{19}F NMR spectra were acquired 21 °C and 60 °C.

S5.2.2.6 Spectrometer Settings
Nucleus: ^{19}F
Pulse sequence: zg30
Number of scans: 8
Temperature: 333 K
Relaxation Delay: 10 s

Nucleus: ^{31}P
Pulse sequence: zg30
Number of scans: 1
Temperature: 294 K
Relaxation Delay: 6 s

S5.2.2.7 Calculations and Plots
The equilibria in DMF and DMAc are intrinsically different, as evident by comparison of Figure S50 and Figure S51. There is no significant difference in chemical shift at 21 °C and 60 °C in DMF, but a notable difference in DMAc. The data in DMAc could not be fitted to a simple model and was not further explored.

*Table S43: Concentration of reactants in each sample of the titration [$(PAr_3)_2NiCl_2$] (**17**) with $ZnCl_2$ in DMAc.*

All concentrations are in mM

Run	$[NiCl_2]_0$	$[PAr_3]_0$	$[ZnCl_2]_0$
A	17.5	35.0	0.0
B	17.5	35.0	7.8
C	17.5	35.0	15.6
D	17.5	35.0	23.4
E	17.5	35.0	31.2
F	17.5	51.4	0.0
G	17.5	51.4	7.8
H	17.5	51.4	15.6
I	17.5	51.4	23.4
J	17.5	51.4	31.2

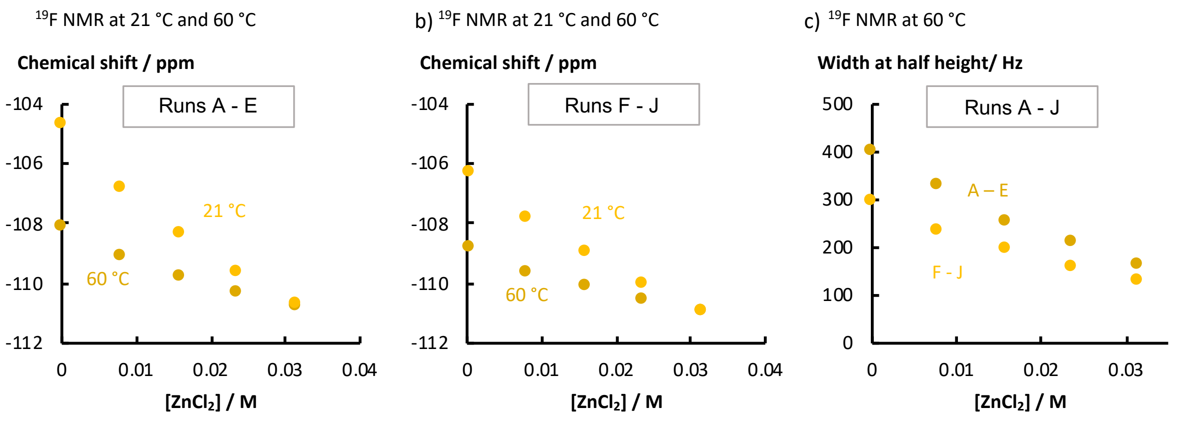

Figure S51: a) and b) Time-average ^{19}F NMR chemical shift of the free and coordinated phosphine ligand plotted against the $ZnCl_2$ concentration, at 21 °C and at 60 °C. The chemical shift asymptotes to that of the free ligand with increasing $ZnCl_2$ concentration. Runs A-E have a phosphine concentration of 35.0 mM and runs F-J had a phosphine concentration of 51.4 mM. There is a significant difference between the equilibria at 21 °C and 60 °C. c) Width at half height for the time-average ^{19}F NMR chemical signals in runs A-J at 21 °C and at 60 °C. The signals became sharper with increasing $ZnCl_2$ concentration.

S5.2.3 Titration of [$NiCl_2$(glyme)] with PAr_3

$$[\mathrm{NiCl_2(glyme)}] + 2\ \mathrm{L} \rightleftarrows [\mathrm{LNiCl_2}] + \mathrm{L} \rightleftarrows [\mathrm{L_2NiCl_2}](\mathbf{17})$$

Scheme S34: Titration of $NiCl_2$·glyme with PAr_3.

All stock solutions were prepared in the glove box under nitrogen atmosphere.

S5.2.3.1 $NiCl_2$·glyme stock solution
A stock solution of $NiCl_2$·glyme (19.7 mg, 89.7 µmol) was prepared in DMF in a volumetric flask (2 mL).

S5.2.3.2 PAr_3 stock solution
A stock solution of PAr_3 (52.4 mg, 166 µmol) in DMF was prepared (2 mL).

S5.2.3.3 Shimming and Tuning of the Spectrometer
The spectrometer was shimmed to ^{1}H and tuned to ^{19}F with each sample.

S5.2.3.4 Reaction Mixtures
All samples were prepared with $NiCl_2$·glyme stock solution (0-250 µL), PAr_3 stock solution (100-400 µL) and DMF (0-400 µL).

The exact concentrations are given in Table S44Table S42.

S5.2.3.5 Spectrometer Settings
Nucleus: ^{19}F
Pulse sequence: zg30
Number of scans: 8
Temperature: 333 K
Relaxation Delay: 10 s

Table S44: Concentrations of all reagents in each sample of the titration of $NiCl_2$ with PAr_3

All concentrations are in mM

Run	$[Ni]_0$	$[PAr_3]_0$
A	0	27.6
B	14.9	13.8
C	14.9	27.6
D	14.9	55.2

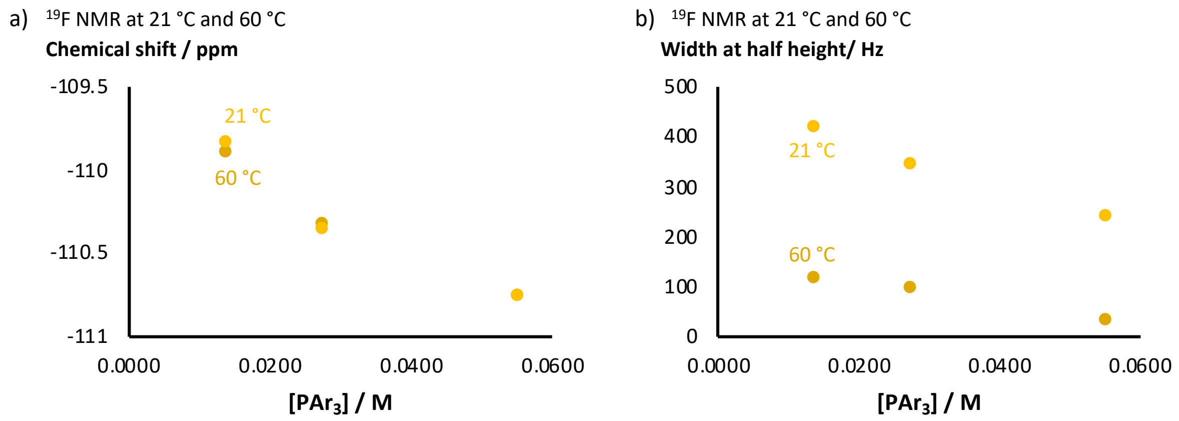

Figure S52: a) Time average of the ^{19}F chemical shift of the free and complexed phosphine ligand plotted against the total PAr_3 concentration. The chemical shifts decrease with increasing total phosphine concentration, and towards the signal of the free ligand. There was no significant difference between the shifts at 60 °C versus 21 °C. b) Width at half height of the signal plotted against the total phosphine concentration in the titration at 60 °C and at 21 °C.

S5.2.4 Equilibration of $ZnAr_2$ (19) + $ZnCl_2$ with ArZnCl (16)

$$\underset{\textbf{19}}{ZnAr_2} + ZnCl_2 \underset{DMF}{\rightleftharpoons} \underset{\textbf{16}}{2\ ZnArCl}$$

*Scheme S35: Titration of $ZnAr_2$ (**19**) with $ZnCl_2$.*

All stock solutions were prepared in the glove box under nitrogen atmosphere.

S5.2.4.1 $ZnCl_2$ stock solution

A stock solution of $ZnCl_2$ (11.2 mg, 82.2 µmol) in DMF was prepared in a volumetric flask (1 mL).

*S5.2.4.2 $ZnAr_2$ (**19**) stock solution*

A stock solution of $ZnAr_2$ (**19**, 22.9 mg, 89.6 µmol) and 1-fluoronaphthalene (IS) (7 µL, 54.2 µmol) in DMF was prepared in a volumetric flask (1 mL).

S5.2.4.3 Shimming and Tuning of the Spectrometer

The spectrometer was shimmed to sample C at 60 °C and tuned to ^{19}F.

S5.2.4.4 Reaction Mixtures

All samples were prepared with $ZnAr_2$ (**19**) stock solution (200 µL), DMF (200 - 400 µL) and $ZnCl_2$ stock solution (0 - 200 µL).

Runs A, C & D were monitored at 60 °C while run B was monitored at 5 °C.

Sample A was then cooled to 5 °C and another NMR spectrum was acquired. The solution was then spiked with the $ZnCl_2$ stock solution to identify the peaks.

S5.2.4.5 Spectrometer Settings

Nucleus: ^{19}F
Pulse sequence: zg30
Number of scans: 1
Temperature: 278 and 333 K
Relaxation Delay: 0 s
Interscan Delay: >10 s

Table S45: Concentration of reactants in Runs A-D for the titration of $ZnAr_2$ with $ZnCl_2$.

All concentrations are in mM

Run	$[IS]_0$	$[19]_0$	$[ZnCl_2]_0$
A	18.1	29.9	13.7
B	18.1	29.9	20.5
C	18.1	29.9	27.4
D	18.1	29.9	0.0

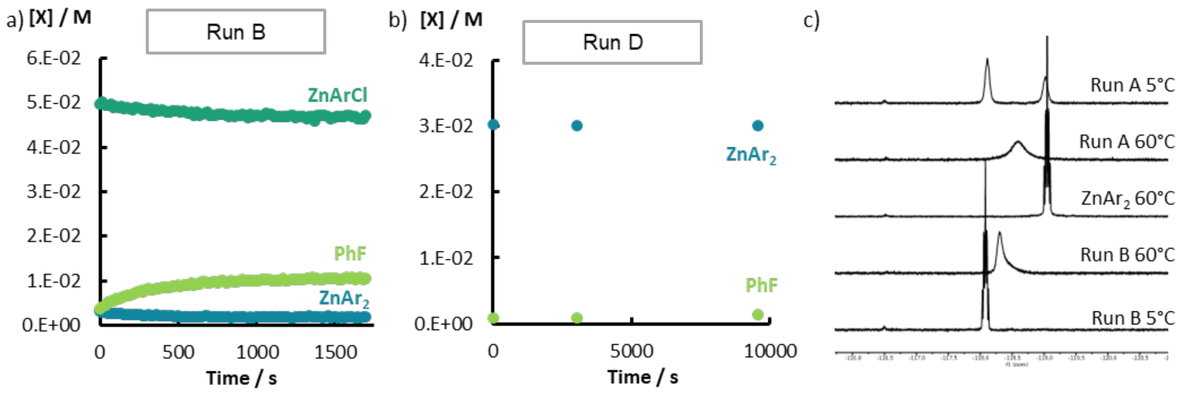

*Figure S53: a) Concentration vs time plot after rapid equilibration of $ZnAr_2$ (**19**) + $ZnCl_2$ with ZnArCl (**16**) to slowly generate PhF (**3**) by reaction with residual bound water released from $ZnCl_2$. b) Concentration vs time plot to demonsrate the stability of $ZnAr_2$ (**19**) in DMF at 60 °C; only a small amount of PhF (**3**) is produced over a period of hours. c) Example ^{19}F NMR spectra of Runs A and B, with equilibrium at the NMR scale at 60 °C, plus a spectrum of a reference sample of $ZnAr_2$ (**19**).*

S5.2.5 Titration of $ZnCl_2$ with PAr_3

$$\mathrm{L} + [\mathrm{ZnCl_2}] \rightleftarrows [(\mathrm{L})_2\mathrm{ZnCl_2}]\ (complexation\ not\ detected\ in\ DMF)$$

Scheme S36: Reaction of $ZnCl_2$ with PAr_3.

S5.2.5.1 PAr_3 stock solution
A stock solution of PPh_3 (393 mg, 1.24 mmol) in DMF (5 mL) was prepared.

S5.2.5.2 $ZnCl_2$ stock solution
A stock solution of $ZnCl_2$ (200 mg, 1.47 mmol) in DMF (5 mL) was prepared.

S5.2.5.3 Reaction mixtures
$ZnCl_2$ stock solution (100 µL), PAr_3 stock solution (500 µL – 800 µL) and DMF (100 µL – 400 µL) were added to a vial. The total volume in all vials was 1 mL. The concentrations are shown in Table S46. All solutions were analysed by ^{19}F and ^{31}P and NMR spectroscopy.

S5.2.5.4 Shimming and Tuning of the Spectrometer
The spectrometer was shimmed and tuned to each sample.

S5.2.5.5 Spectrometer Settings
Nucleus: ^{19}F
Pulse sequence: zg30
Number of scans: 8
Temperature: 333 K
Relaxation Delay: 10 s

Nucleus: ^{31}P
Pulse sequence: zg30
Number of scans: 256
Temperature: 333 K
Relaxation Delay: 2 s

Table S46: Concentration of species in the titration of $ZnCl_2$ with PAr_3.

All concentrations are given in mM

Run	$[PAr_3]$	$[ZnCl_2]$	δ (^{19}F)/ ppm
A	198.8	29.4	-113.18
B	173.9	29.4	-113.18
C	149.1	29.4	-113.18
D	124.2	29.4	-113.18

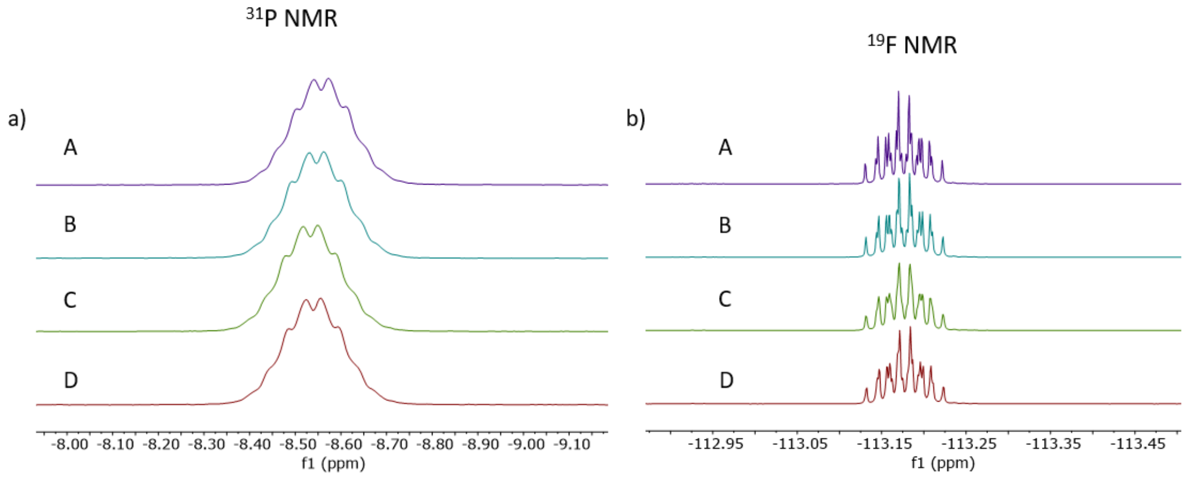

Figure S54: a) ^{31}P NMR signal of PAr_3 on titration with $ZnCl_2$. b) ^{19}F NMR signal of PAr_3 on titration with $ZnCl_2$. In neither (a, b) do the peak shapes or chemical shifts detectably change, indicative of no significant interaction between $ZnCl_2$ and PAr_3 in DMF at 60 °C.

S5.2.6 Titration of LiCl with $ZnCl_2$

$$\mathrm{LiCl} + [\mathrm{ZnCl_2}] \rightleftarrows [\mathrm{Li(DMF)}_n]^+[\mathrm{ZnCl_3(DMF)}]^-$$

Scheme S37: Titration of LiCl with $ZnCl_2$.

S5.2.6.1 LiCl stock solution

A stock solution of LiCl (52.9 mg, 1.25 mmol) in DMF (5 mL) was prepared.

S5.2.6.2 $ZnCl_2$ stock solution

A stock solution of $ZnCl_2$ (49.5 mg, 363 µmol) in DMF (5 mL) was prepared.

S5.2.6.3 Reaction mixtures

$ZnCl_2$ stock solution (100 µL - 1 mL), LiCl stock solution (100 µL – 1 mL) and DMF (0 mL – 1.40 mL) were added to a vial. The total volume in all vials was 2 mL. The concentrations are shown in Table S47. All solutions were analysed by ^{7}Li NMR spectroscopy.

S5.2.6.4 Shimming and Tuning of the Spectrometer

The spectrometer was shimmed and tuned to each sample for entries A-D. For entries E-H the spectrometer was shimmed and tuned to sample H.

S5.2.6.5 Spectrometer Settings

Nucleus: ^{7}Li
Pulse sequence: zg30
Number of scans: 64 (for samples A-D), 16 (for samples E-H)
Temperature: 300 K (for samples A-D), 294 K (for samples E-H)
Relaxation Delay: 6 s

Table S47: Concentration of reactants in each sample of the titration of LiCl with $ZnCl_2$.

All concentrations are given in mM

**Run**	**[LiCl]**	**[$ZnCl_2$]**	**δ (^{7}Li) / ppm**	**Whh / Hz**
A	118.0	36.7	1.23	0.54
B	59.0	36.7	1.20	0.70
C	29.0	36.7	1.17	0.83
D	12.0	36.7	1.16	1.34
E	65.0	14.6	0.69	1.54
F	65.0	43.8	0.70	1.39
G	65.0	72.9	0.74	1.38
H	65.0	102.1	0.79	1.04

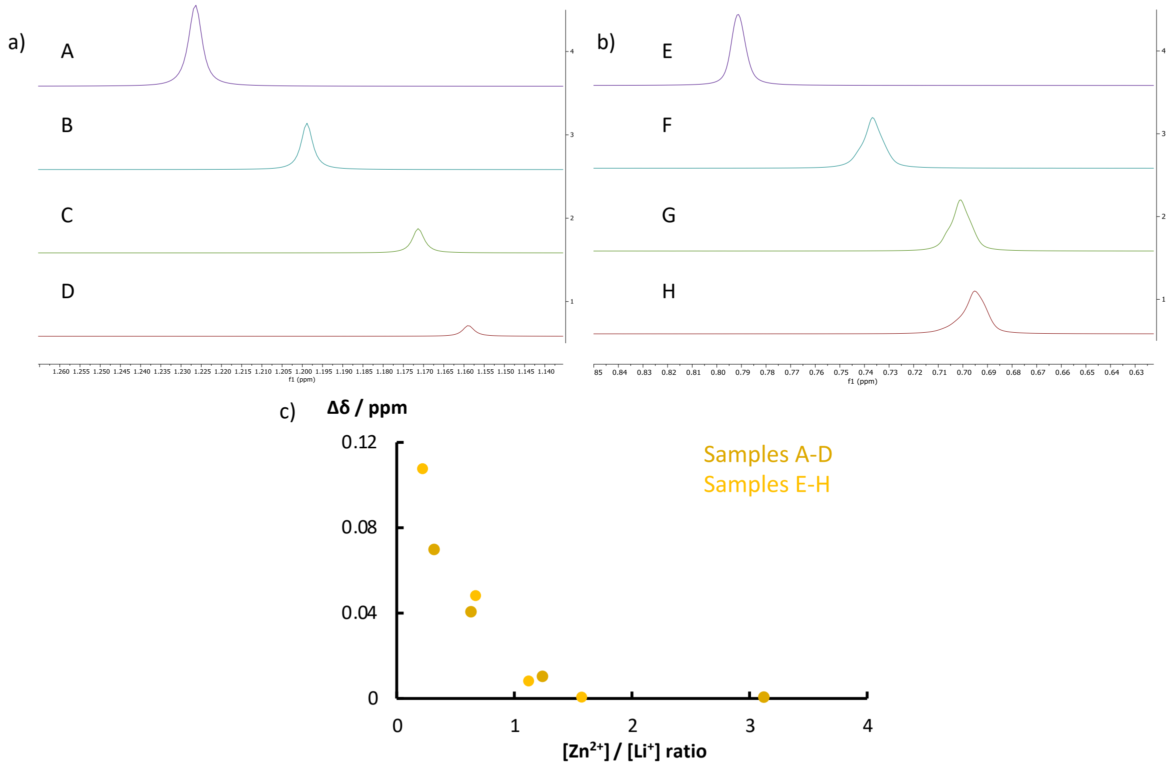

Figure S55: a) ^{7}Li NMR spectra the titration of $ZnCl_2$ with LiCl. b) ^{7}Li NMR spectra of the titration of LiCl with $ZnCl_2$. The signals shifted to higher chemical shift at lower Zn^{2+}/Li^{+} ratios; the asymmetric peak shape is due to due poor shimming. Because there was no internal reference in the ^{7}Li spectra, the spectrometer settings were kept constant between runs to allow direct comparison. c) The change in ^{7}Li NMR chemical shift as a function of the Zn/Li ratio, with samples D and H set to 0.

S5.3 Low Temperature ^{31}P NMR Spectroscopic Studies

*S5.3.1.1 ArCl (**1**) stock solution*

A stock solution of ArCl (**1**, 120 µL, 1.13 mmol) and 1-fluoronaphthalene (IS) (110 µL, 852 µmol) was prepared with DMF in a volumetric flask (2 mL).

S5.3.1.2 Run A

$ZnCl_2$ (29.3 mg, 215 µmol), PPh_3(192 mg, 730 µmol) and DMF (1.5 mL) were added to a flask. A 0.5 mL sample of this solution was transferred to an NMR tube, sealed with a J Young valve, and analysed by ^{31}P VT-NMR spectroscopy.

S5.3.1.3 Run B

[$NiCl_2$(glyme)] (33.1 mg, 151 µmol), PPh_3 (194 mg, 739 µmol), $ZnCl_2$ (24.3 mg, 178 µmol) and DMF (1.5 mL) were added to a flask. A 0.5 mL sample of this solution was transferred to an NMR tube, sealed with a J Young valve, and analysed by ^{31}P VT-NMR spectroscopy.

S5.3.1.4 Run C

A stock solution of [$NiCl_2$(glyme)] (26.9 mg, 122 µmol) and PPh_3 (284 mg, 1.1 mmol) were made in DMF in a volumetric flask. The solution was then transferred into a Schlenk flask and Zn powder (51.2 mg, 783 µmol) added. The flask was transferred out of the glovebox and connected to the vacuum line. The mixture was stirred and heated to 60 °C for 30 minutes. After cooling back to ambient temperature, the flask was transferred back into the glovebox and a 0.5 mL sample taken, transferred to an NMR tube, sealed with a J Young valve, and analysed by ^{31}P VT-NMR spectroscopy.

S5.3.1.5 Run D

A sample of the activated catalyst solution (0.1 mL) from Run C was taken and transferred to an NMR tube, with DMF (0.3 mL) and ArCl (**1**) stock solution (0.1 mL), then sealed with a J Young valve, and analysed by ^{31}P VT-NMR spectroscopy.

S5.3.1.6 Shimming and Tuning of the Spectrometer

The spectrometer was shimmed and tuned to each sample.

S5.3.1.7 Spectrometer Settings

Nucleus: ^{31}P
Pulse sequence: zg30
Number of scans: 64
Temperature: varied
Relaxation Delay: 2 s

^{31}P spectra of all samples were acquired at 300 K (27 °C), 293 K (20 °C), 283 K (10 °C), 273 K (0 °C), 263 K (-10 °C), 253 K (-20 °C) and 233 K (-40 °C) on a Bruker Avance III 400 MHz spectrometer with a BBFO+ probe, and at 313 K (40 °C), 323 K (50 °C) and 333 K (60 °C) on a Bruker Avance III HD 400 MHz spectrometer with a 5 mm BBO CryoProbe Prodigy (LN2).

Table S48: Concentration of reactants samples analysed at low temperature.

All concentration are given in mM

Run	[Ni]	[PAr_3]	[$ZnCl_2$]	[1]	[IS]	[Zn]
A	0.0	486.9	143.5	0.0	0.0	0.0
B	100.3	492.9	118.8	0.0	0.0	0.0
C	61.2	541.8	0.0	0.0	0.0	391.3
D	12.2	108.4	0.0	112.7	85.2	78.3

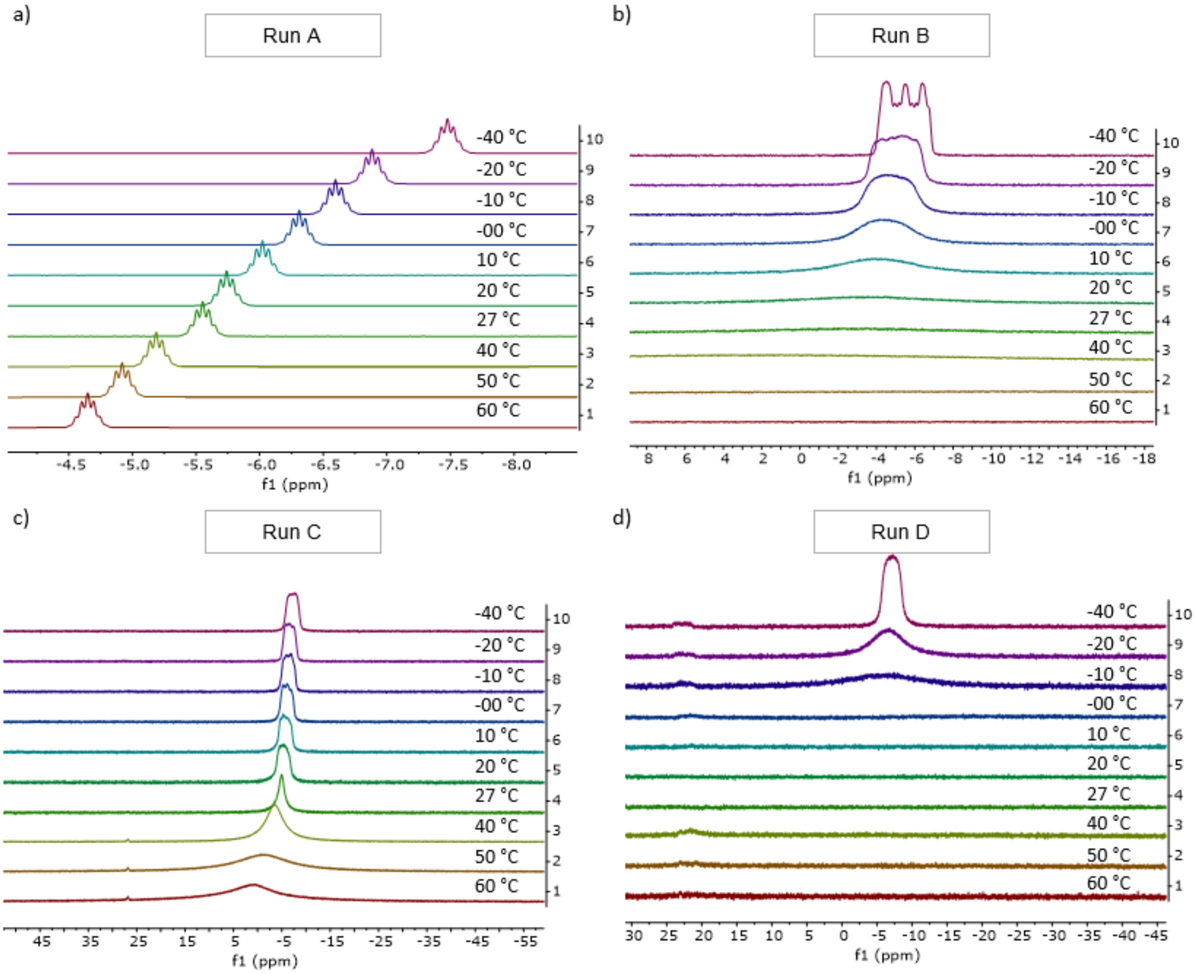

*Figure S56: a) ^{31}P NMR signals of mixtures of $ZnCl_2$ and PPh_3 at various temperatures. The changes in chemical shift are caused by the change in temperature, not by $ZnCl_2$ complexation. b) ^{31}P NMR signals of the PPh_3 ligand in Run B, indicating that there is a rapid exchange between free and Ni^{II} complexed species, accompanied by some changes in spin state at low temperatures c) ^{31}P NMR signals of the activated catalyst specie(s) $[(PPh_3)_nNi(DMF)_m]$; where n, and m, are undefined, and changes as the temperature is raised. d) ^{31}P NMR signal of the activated catalyst after addition of ArCl (**1**) to induce semicatalytic homocoupling via $L_2NiArCl$ (**6**). The spectra have some similarity to those in run B which contains a Ni^{II} species, PAr_3 and $ZnCl_2$.*

S6 UV-Vis Spectroscopic Analyses

S6.1 UV-Vis Analysis of the Mixed Metallate 21_{DMF}

$$NiCl_2 + ZnCl_2 \xrightarrow[DMF]{} [Ni(DMF)_6][Zn(DMF)Cl_3]_2$$

21

Scheme S38: Reaction of $NiCl_2$·glyme with $ZnCl_2$.

S6.1.1.1 [$NiCl_2$(glyme)] stock solution

A stock solution of [$NiCl_2$(glyme)] (80.5 mg, 366 µmol) was prepared in DMF in a volumetric flask (10 mL).

S6.1.1.2 $ZnCl_2$ stock solution

A stock solution of $ZnCl_2$ (102 mg, 746 µmol) was prepared in DMF in a volumetric flask (10 mL).

*S6.1.1.3 Complex **21**$_{DMF}$ stock solution*

A stock solution of mixed metallate **21**$_{DMF}$ (45.3 mg, 45.8 µmol) was prepared in DMF in a volumetric flask (1 mL).

S6.1.1.4 Reaction mixtures

$ZnCl_2$ stock solution (0 mL - 1.2 mL), [$NiCl_2$(glyme)] stock solution (0 µL - 600 µL), DMF (0 mL- 1.5 mL) and mixed metallate **21**$_{DMF}$ stock solution (0 µL - 500 µL) were added to vials. Each vial contained 2 mL of solution. Concentrations are shown in Table S49. Each solution was filtered through a 20-micron disposable syringe filter and then analysed by UV-Vis spectroscopy.

S6.1.2 UV-VIS Measurements

The spectra were acquired from 250-600 nm. The background was measured using a blank sample of DMF (2 mL).

*Table S49: Concentrations of reactants used in the UV-Vis analysis of complex **21**$_{DMF}$. Run K contains a reference sample of the mixed metallate complex **21**$_{DMF}$.*

All concentrations are in mM

Run	$[Ni]_0$	$[ZnCl_2]_0$	[21]
A	11.0	1.9	0
B	11.0	3.7	0
C	11.0	7.5	0
D	11.0	11.2	0
E	11.0	14.9	0
F	11.0	18.7	0
G	11.0	22.4	0
H	11.0	29.8	0
I	11.0	37.3	0
J	11.0	44.8	0
K	0	0	11.5

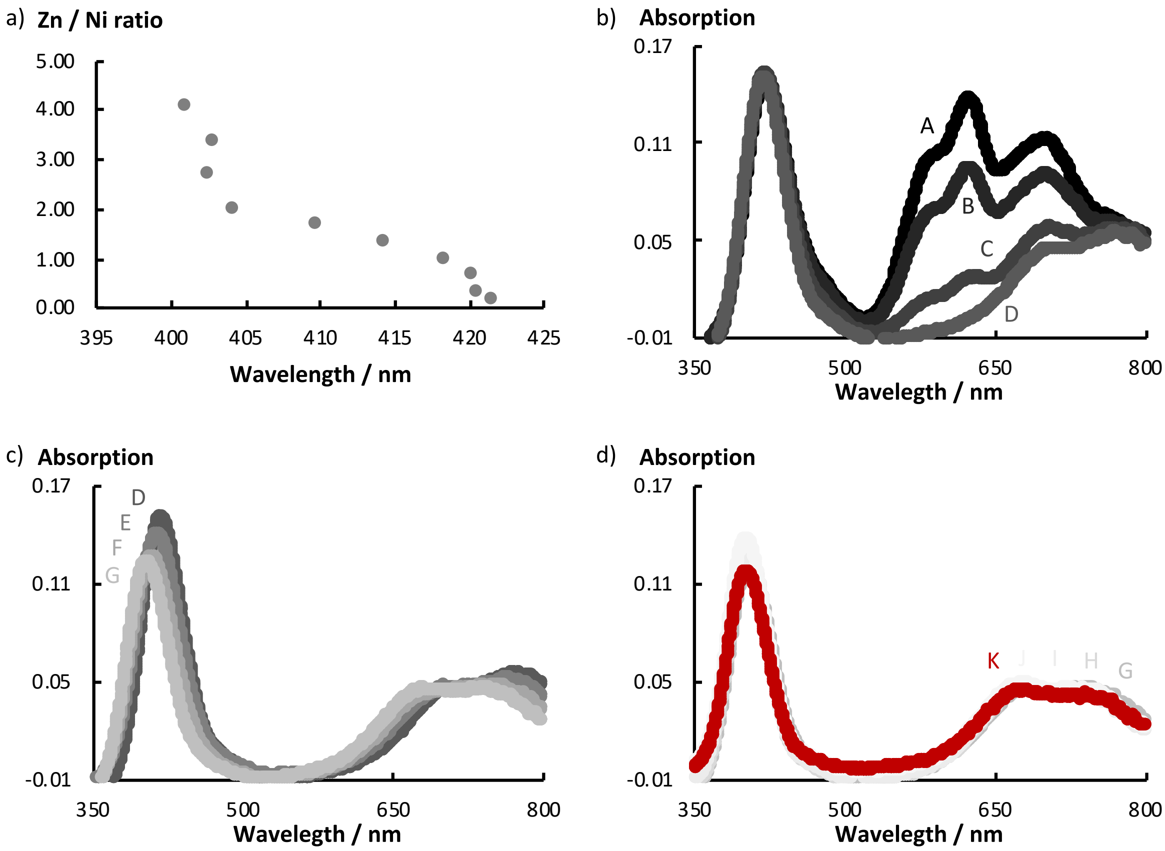

Figure S57: a) Zn/Ni ratio versus against the maximum wavelength of maximum absorbance. Samples with a Zn/Ni ratios over two had maximum in absorbance at wavelengths between 400 nm and 405 nm. Samples with Zn/Ni ratios under 1 had an absorbance maximum at around 420 nm. The samples with ratios between 1 and 2 have maxima between 405 nm and 420 nm. b) UV-Vis spectra of Runs A-D. The spectra show a distinctive absorbance between 550 nm and 800 nm which is also observed in [$NiCl_2$(glyme)]. This absorbance band disappears when Zn/Ni ratio > 1. c) UV-VIS spectra of runs D-G. The maximum absorbance between 400 nm and 430 nm shifts to lower wavelength as the Zn/Ni ratio is raised.. d) UV-Vis spectra of runs G-J. The UV-vis spectra change slightly at these higher Zn/Ni ratios. The spectrum of the reference sample ***21***$_{DMF}$ *sample is shown in red. The spectrum confirms that for samples G to J, the Zn/Ni ≥ 2.*

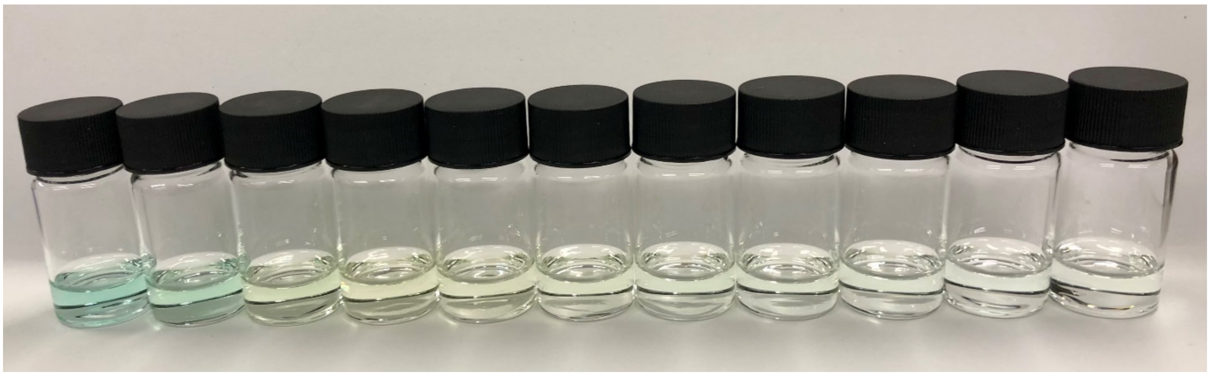

Figure S58: Solutions from Runs A to K (left to right) - analysed by UV-Vis spectroscopy. As the Zn/Ni ratio is raised, the colour of the solutions change from blue to yellow to colourless.

S6.2 Estimation of the limiting solubility of $ZnCl_2$ in DMF and DMAc.

$$n\,[\mathrm{OH}]^- + [\mathrm{ZnCl_2}] + [Salic] \rightleftarrows [Salic(\mathrm{ZnCl_2})]^{n-}\ (detected\ by\ UV\text{–}Vis)$$

Scheme S39: Coordination of the (di)anion generated from N-(2-hydroxy-5-methylphenyl)salicylideneimine ('Salic') to $ZnCl_2$.

The procedure was adapted from literature. [S9]

S6.2.1.1 NaOH stock solution

A stock solution of NaOH (10.1 g, 253 mmol) was prepared in water (250 mL). A 100 µL volume sample was taken and diluted with water in a 100 mL volumetric flask.

S6.2.2 Solubility of $ZnCl_2$ in DMF

S6.2.2.1 Ligand stock solution

A stock solution of the *N*-(2-hydroxy-5-methylphenyl)salicylideneimine (44.8 mg, 197 µmol) in DMF (25 mL) was made. 700 µL was taken from this solution and diluted with DMF in a volumetric flask (100 mL).

S6.2.2.2 $ZnCl_2$ stock solution

A stock solution of $ZnCl_2$ (53.8 mg, 394.8 µmol) was prepared with water (2 mL). 1 mL of this stock solution was taken and diluted with water in a volumetric flask (25 mL). 10 mL of the resulting solution were taken and diluted with water in a volumetric flask (25 mL).

S6.2.2.3 Sample solution

An excess of $ZnCl_2$ was added to DMF in the filtration device shown in Figure S59, and the solution stirred at room temperature for approximately 30 minutes, ensuring that some solid remained undissolved, i.e. that a saturated solution had been generated. An aliquot (1 mL) was taken by inserting the filter tube and allowing the solution to reach the mark, then closing the valve. The tube was removed, carefully wiped with a paper towel, and the contents then washed into a volumetric flask (250 mL) and made up to the mark. A sample of 800 µL was taken and diluted with water in another volumetric flask (25 mL).

The above process was conducted 3 times at room temperature and 3 times at 60 °C. All six samples were diluted at room temperature.

S6.2.3 Solubility of $ZnCl_2$ in DMAc

S6.2.3.1 Ligand stock solution

A stock solution of the *N*-(2-hydroxy-5-methylphenyl)salicylideneimine (44.8 mg, 197 µmol) in DMF (25 mL) was made. 700 µL was taken from this solution and diluted with DMF in a volumetric flask (100 mL).

S6.2.3.2 $ZnCl_2$ stock solution

A stock solution of $ZnCl_2$ (53.8 mg, 394.8 µmol) was prepared with water (2 mL). 1 mL of this stock solution was taken and diluted with water in a volumetric flask (25 mL). 10 mL of the resulting solution were taken and diluted with water in a volumetric flask (25 mL).

S6.2.3.3 Sampling saturated solutions

An excess of $ZnCl_2$ was added to DMAc in the filtration device shown in Figure S59, and the solution stirred at room temperature for approximately 30 minutes, ensuring that some solid remained undissolved, i.e. that a saturated solution had been generated. An aliquot (1 mL) was taken by

inserting the filter tube and allowing the solution to reach the mark, then closing the valve. The tube was removed, carefully wiped with a paper towel, and the contents then washed into a volumetric flask (250 mL) and made up to the mark. A sample of 800 µL was taken and diluted with water in another volumetric flask (25 mL).

The above process was conducted 3 times at room temperature and 3 times at 60 °C. All six samples were diluted at room temperature.

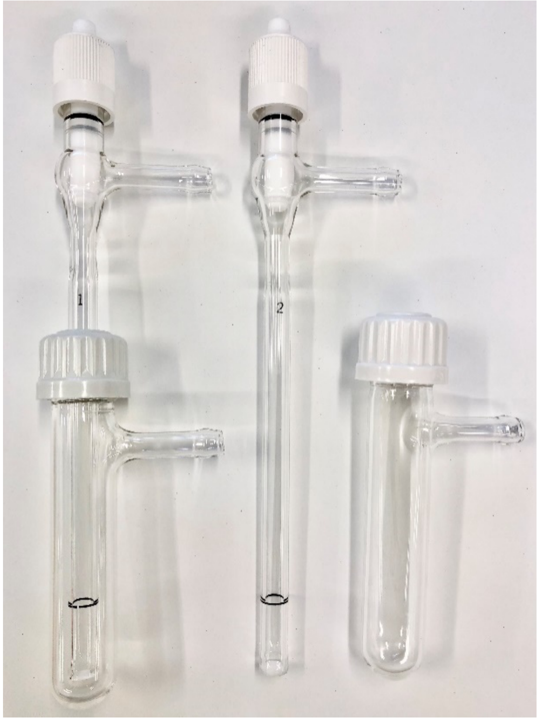

Figure S59: The filtration device used to determine the solubility of $ZnCl_2$ in DMF and in DMAc. The device consists of a long glass tube with a sintered glass filter at the bottom and a J Young valve at the top. This inserts into a Schlenk tube and is held in place with a compression fitting. The Schlenk tube contains the saturated solution, approximately half filled, and is retained in the heating bath if the measurement is made above ambient temperature. The black line on the tube is a calibration mark indicating a 1.00 mL volume within the lower section of the glass tube, including the liquid retained in the sinter-glass filter section. After preparation of the saturated solution, the glass tube is lowered into the solution and the solution allowed to enter the tube through the filter and rise above the mark. A 1.00 mL sample is then collected by closing the J. Young valve and partially withdrawing the tube, then slight opening / closing of the valve until the liquid level is at the mark. The tube is then fully withdrawn, and after briefly wiping the outside with a paper towel, inserted into the top of a volumetric flask. The J Young valve is opened and full removed, and the 1.00 mL contents of the tube rinsed into the volumetric flask with a large volume of diluting solvent, in this case water, and then the volumetric flask made up to the mark.

S6.2.4 UV-VIS Measurements

UV-Vis spectra were acquired between 250-600 nm. The background was measured using a sample containing water (400 µL) and DMF (3 mL) or water (400 µL) and DMAc (3 mL) as appropriate.

Calibration samples containing ligand stock solution (3 mL), NaOH stock solution (300 µL), $ZnCl_2$ stock solution (10 µl -40 µl) and water (60-100 µl) were measured for both solvents. The total volume of each sample was 3.4 mL. The concentrations are shown in Table S50.

The $ZnCl_2$ samples containing ligand stock solution (3 mL), NaOH stock solution (300 µL) and 100 µL sample solution were measured. This process was repeated for all samples.

Table S50: Concentrations of the calibration solutions for the solubility measurements of $ZnCl_2$.

All concentrations are in mol/L			
DMF - calibration		DMAc - calibration	
$[ZnCl_2]$	[Ligand]	$[ZnCl_2]$	[Ligand]
0.00E+00	4.87E-05	0.00E+00	4.80E-05
9.28E-06	4.87E-05	9.34E-06	4.80E-05
1.86E-05	4.87E-05	1.87E-05	4.80E-05
2.78E-05	4.87E-05	2.80E-05	4.80E-05
3.71E-05	4.87E-05	3.74E-05	4.80E-05

S6.2.5 Calculation and Plots

Absorbance values at 363 nm, 438 nm and 393 nm (an isosbestic point) were recorded. The absorbance ratios were calculated by diving the absorbance at two wavelengths (368 nm and 439 nm) by the absorbance at the isosbestic point (393 nm). The absorbance ratios of the calibration samples were then plotted against the $ZnCl_2$ concentration. The concentrations of $ZnCl_2$ in the samples generated from the saturated solutions in DMF and DMAc were calculated using the calibration plots.

solubility of $ZnCl_2$ in DMF:
rt: 220.2 ± 16.7 g/L
60 °C: 311.1 ± 6.4 g/L

solubility of ZnCl2 in DMAc:
rt: 74.8 ± 10.5 g/L
60 °C: 191.8 ± 3.9 g/L

DMF

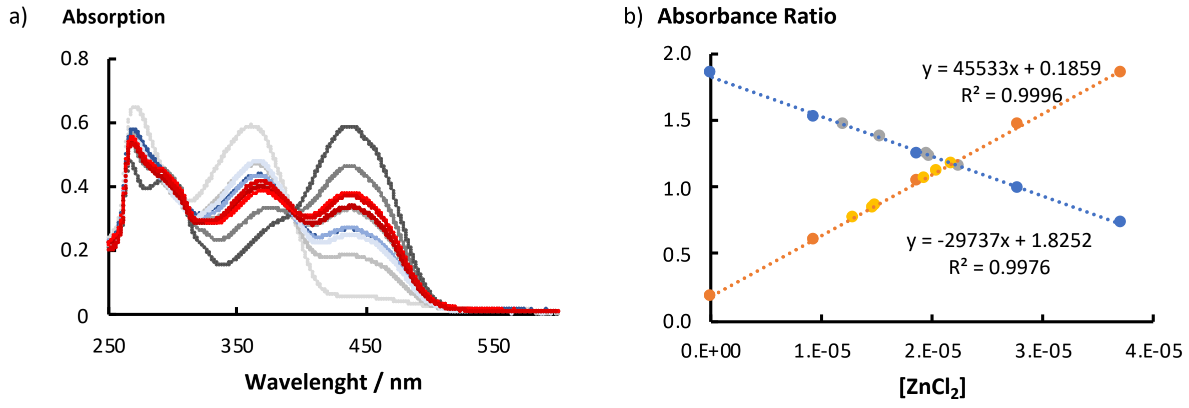

DMAc

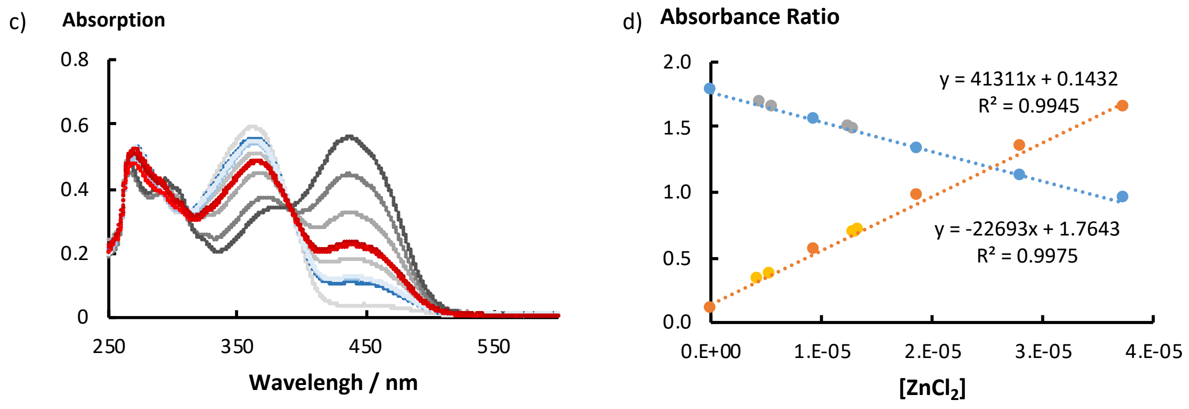

Figure S60: a) UV-Vis measurements of the calibration and sample in DMF. Calibration curves were shown in grey. $ZnCl_2$ solubility measurements at 60 °C were shown in red. $ZnCl_2$ solubility measurements at room temperature were shown in blue. b) Absorbance ratio of the calibration curve in DMF at 368 nm were shown in blue and absorbances of the calibration curve at 439 nm were shown in orange. Both datasets gave a linear trend when plotted against the $ZnCl_2$ concentration. The absorbances of the samples at 368 nm were plotted in grey and the absorbances of the samples at 439 nm were shown in yellow. c) UV-Vis measurements of the calibration and sample in DMAc. Calibration curves were shown in grey. $ZnCl_2$ solubility measurements at 60 °C were shown in red. $ZnCl_2$ solubility measurements at room temperature were shown in blue. d) Absorbance of the calibration curve in DMAc at 368 nm were shown in blue and absorbances of the calibration curve at 439 nm were shown in orange. Both datasets gave a linear trend when plotted against the $ZnCl_2$ concentration. The absorbances of the samples at 368 nm were plotted in grey and the absorbances of the samples at 439 nm were shown in yellow.

S7 EPR Spectroscopic Analysis of Ni Speciation.

All reactions were prepared in the glovebox.

S7.1.1.1 $ZnCl_2$ stock solution

A stock solution of $ZnCl_2$ (140 mg, 1.0 mmol) was prepared in DMF (2 mL).

S7.1.2 Reaction 1: Stoichiometric Homocoupling of $L_2NiArCl$ (6) without $ZnCl_2$

$L_2NiArCl$ (**6**, 21.4 mg, 26.0 µmol) and PAr_3 (67 mg, 212 µmol) were added to a small vial. DMF (2 mL) was added, and the mixture was shaken vigorously to dissolve $L_2NiArCl$ (**6**). The solution was heated to 60 °C and stirred for 30 minutes, cooled to ambient temperature, a sample transferred into an EPR tube, and the contents rapidly cooled in liquid N_2 to generate a glass.

S7.1.3 Reaction 2: Stoichiometric Homocoupling of $L_2NiArCl$ (6) with $ZnCl_2$

$L_2NiArCl$ (**6**, 22 mg, 26.8 µmol) and PAr_3 (115 mg, 364 µmol) were added to the vial. DMF (2 mL) was added, and the mixture was shaken vigorously to dissolve $L_2NiArCl$ (**6**). The solution was heated to 60 °C with stirring and after 5 minutes at 60 °C, the $ZnCl_2$ stock solution (200 µL) was added. After 3 minutes the reaction was cooled to ambient temperature, a sample transferred into an EPR tube, and the contents rapidly cooled in liquid N_2 to generate a glass.

S7.1.4 Reaction 3: [Ni(COD)$_2$] and [NiCl$_2$(glyme)] Comproportionation without $ZnCl_2$

[Ni(COD)$_2$] 4 mg, 14.5 µmol), [NiCl$_2$(glyme)] (3.2 mg, 14.6 µmol) and PAr_3 (15 mg, 364 µmol) were placed in a vial with a stirring bar. DMF (2 mL) was added and the sample was heated to 60 °C for 30 minutes. After cooling to ambient temperature, a sample was transferred into an EPR tube, and the contents rapidly cooled in liquid N_2 to generate a glass.

S7.1.5 Reaction 4: [Ni(COD)$_2$] and [NiCl$_2$(glyme)] Comproportionation with $ZnCl_2$

Ni(COD)$_2$] 4 mg, 14.5 µmol), [NiCl$_2$(glyme)] (3.2 mg, 14.6 µmol) and PAr_3 (15 mg, 364 µmol) were placed in a vial with a stirring bar. DMF (2 mL) was added, followed by $ZnCl_2$ stock solution (200 µL). The sample was heated to 60 °C for 30 minutes, cooled to ambient temperature, a sample transferred into an EPR tube, and the contents rapidly cooled in liquid N_2 to generate a glass.

Table S51: Concentrations of reactants used to generate the samples for EPR spectroscopy

All concentrations are in mM

Reaction	$[6]_0$	[PAr$_3$]	[ZnCl$_2$]	[Ni(COD)$_2$]	[NiCl$_2$·glyme]
1	13.0	105.9	0	0	0
2	12.2	165.2	46.7	0	0
3	0	165.2	0	6.6	6.6
4	0	165.2	46.7	6.6	6.6

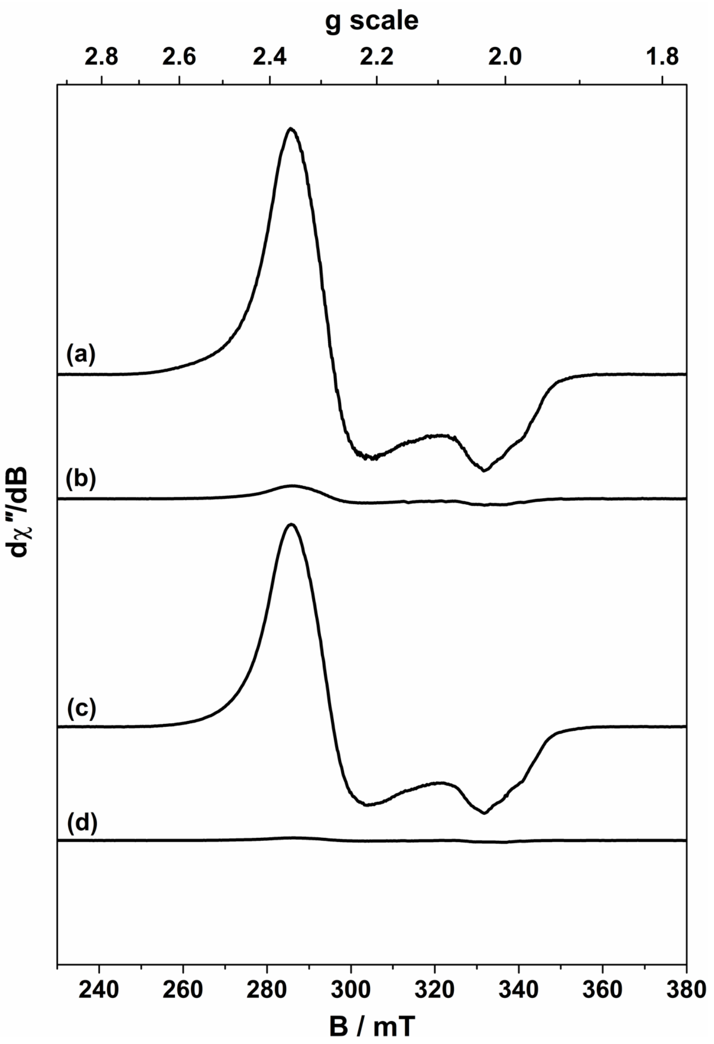

Figure S61: Comparison of the X-band EPR spectra of a) Reaction 1, b) Reaction 2, c) Reaction 3 and d) Reaction 4 in DMF (experimental conditions: frequency, 9.4273 GHz; power, 0.63 mW; modulation, 0.5 mT).

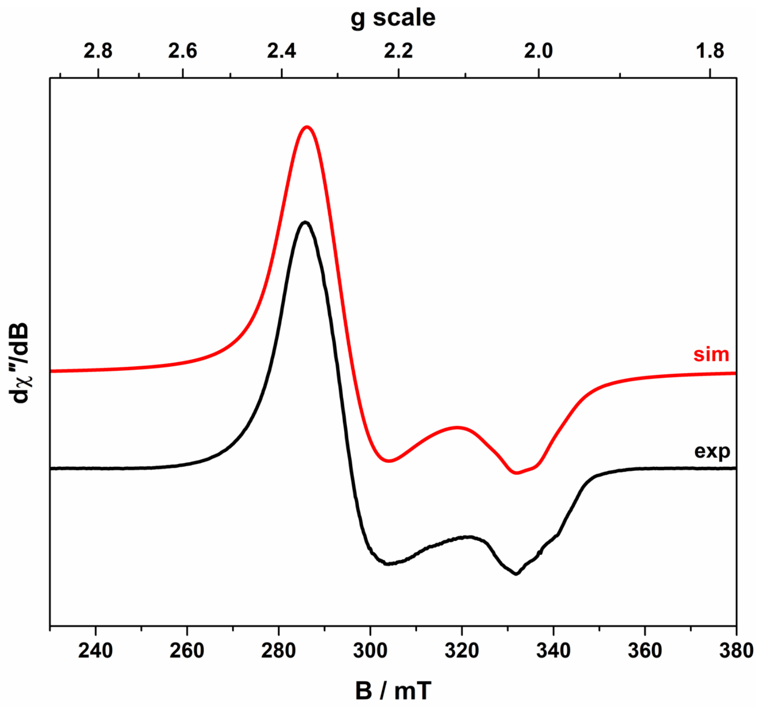

Figure S62: X-band EPR spectrum of Reaction 3 recorded in DMF solution at 77 K (experimental conditions: frequency, 9.4273 GHz; power, 0.63 mW; modulation, 0.5 mT). Experimental data are shown by the black line, and simulation by the dashed red trace: g = (2.352, 2.278, 2.017); A{^{31}P} = (40, 40, 50) × 10^{-4} cm^{-1} (3); W = (20, 40, 15) × 10^{-4} cm^{-1}; σg = (0.010, 0.011, 0.009); σA = (0, 10, -10).

A simulation is proffered though does not represent a unique solution. The only resolved hyperfine splitting is on g_z at ca. 330 mT. The broad line obscures nearly all the hyperfine detail, and the simulation above was achieved by including coupling from three equivalent ^{31}P nuclei, in light of the vast excess of PAr_3. There are other combinations that could be more chemically reasonable for this system, such as two inequivalent phosphines about a tetrahedral Ni(I) centre. A study published in 2006[S10] published several Ni(I)-phosphine combinations, and the spectrum of [Ni(PPh_3)$_3$(OAc)] matches the profile above both with the g-anisotropy ($g_\perp > g_\parallel > g_e$), the Lorentzian line shape and partially resolved hyperfine around $g_\parallel$.

S8 Analyses of kinetic data.

S8.1 Direct homocoupling of Ar-Ni complex 6, second-order steady state rate equation with excess ligand (L); equations 3 and 4 in main manuscript.

The derivation below is based on [L_2Ni(Ar)Cl] complex **6** being in rapid equilibrium ($1/K_L$) with [LNi(Ar)Cl] complex **8**, and the latter undergoing associative equilibrium (K_A) to generate dinuclear complex, [$L_2Ni_2(Ar)_2Cl_2$] **9**, then irreversible Ar/Cl internuclear metathesis (k_m). Equation S8 is the approximation given as equations 3 and 4 in main manuscript.

$$K_L = \frac{[\mathbf{8}]\,[L]}{[\mathbf{6}]}\text{; and thus } [\mathbf{8}] = \frac{[K_L]\,[\mathbf{6}]}{[L]} \qquad \text{(Eq S1)}$$

$$[\mathbf{6}]_{\mathrm{TOT}} - [\mathbf{8}] - 2[\mathbf{9}] = [\mathbf{6}]\text{; and thus } [\mathbf{8}] = \frac{K_L\,[\mathbf{6}]_{\mathrm{TOT}}}{[L]} - \frac{K_L[\mathbf{8}]}{[L]} - \frac{2K_L\,[\mathbf{9}]}{[L]} \qquad \text{(Eq S2)}$$

$$K_A = \frac{[\mathbf{9}]}{[\mathbf{8}][\mathbf{8}]}\text{; and thus } [\mathbf{9}] = K_A[\mathbf{8}]^2 \qquad \text{(Eq S3)}$$

$$[\mathbf{8}]\left(1 + \frac{K_L}{[L]}\left(1 + \frac{2[\mathbf{8}]}{K_A}\right)\right) = \frac{K_L\,[\mathbf{6}]_{\mathrm{TOT}}}{[L]} \qquad \text{(Eq S4)}$$

$$[\mathbf{8}] = \frac{K_L\,[[\mathbf{6}]_{\mathrm{TOT}}}{[L]}\frac{1}{\left(1+\frac{K_L}{[L]}\left(1+\frac{2[\mathbf{8}]}{K_A}\right)\right)} = \frac{[\mathbf{6}]_{\mathrm{TOT}}}{\left(\frac{[L]}{K_L}+1+\frac{2[\mathbf{8}]}{K_A}\right)} \qquad \text{(Eq S5)}$$

$$[\mathbf{9}] = \frac{K_A[\mathbf{6}]_{\mathrm{TOT}}{}^2}{\left(\frac{[L]}{K_L}+1+\frac{2[\mathbf{8}]}{K_A}\right)\left(\frac{[L]}{K_L}+1+\frac{2[\mathbf{8}]}{K_A}\right)} = \frac{[\mathbf{6}]_{\mathrm{TOT}}{}^2}{\frac{[L]}{K_LK_A}\left(\frac{[L]}{K_L}+2+\frac{4\,[\mathbf{8}]}{K_A}+\frac{K_L}{[L]}+4[\mathbf{8}]\frac{K_L}{[L]}(1+[\mathbf{8}])\right)} \qquad \text{(Eq S6)}$$

$$2\frac{d[\mathbf{2}]}{dt} = \frac{k_m\,[\mathbf{6}]_{\mathrm{TOT}}{}^2}{\frac{[L]}{K_LK_A}\left(\frac{[L]}{K_L}+2+\frac{4\,[\mathbf{8}]}{K_A}+\frac{K_L}{[L]}+4[\mathbf{8}]\frac{K_L}{[L]}(1+[\mathbf{8}])\right)} \qquad \text{(Eq S7)}$$

$$when\ [L]_{exo} >> [L]_{endo};\ and\ [L]_{exo} >> K_L;\ and\ K_A \geq 4[\mathbf{8}]\ then:$$

$$2\frac{d[\mathbf{2}]}{dt} \approx k_D\,[\mathbf{6}]_{\mathrm{TOT}}{}^2\ where\ k_D \approx \frac{(K_L)^2K_Ak_m}{[L]_{exo}^2} \qquad \text{(Eq S8)}$$

S8.2 Derivation of integrated rate equation for analysis of mixed order kinetics, *n*, where 1 < *n* < 2; equation 11 in main manuscript.

The derivation below (equations S9 to S25) is based on the simplification of the situation where the kinetics of the reaction of complex **6**, which phenomenologically proceed as -d[**6**]/dt = 2[**2**]/dt = k[**6**]^n (where *n* varies both within and between runs) is approximated by a pair of competing first and second order processes, -d[**6**]/dt = 2[**2**]/dt = *a*[**6**]^1 + b[**6**]^2. The integrated form of this approximation (equation S25; equation 11 in main manuscript) allows fitting of the full temporal evolution of each run, to extract the empirical constants *a* and *b*. With these constants in hand, the rates can be normalised to a standard concentration of complex **6**, in this work, [**6**] = 0.01 M, using rate = *a*[**6**]^1 + b[**6**]^2.

$$rate = \frac{0.5\ d[\mathbf{2}]}{dt} = \frac{-d[\mathbf{6}]}{dt} = a[\mathbf{6}]^1 + b[\mathbf{6}]^2 \qquad \text{(Eq S9)}$$

$$\frac{-d[\mathbf{6}]}{dt} = \frac{a^2[\mathbf{6}]^1 + ab[\mathbf{6}]^2}{a} = \frac{a^2[\mathbf{6}]^1 + ab[\mathbf{6}]^2}{a + b[\mathbf{6}] - b[\mathbf{6}]} \qquad \text{(Eq S10)}$$

$$\frac{-d[\mathbf{6}]}{dt} = \frac{(a+b\,[\mathbf{6}])(a[\mathbf{6}])}{(a + b[\mathbf{6}]) - b[\mathbf{6}]} = \frac{a[\mathbf{6}]}{1 - \frac{b[\mathbf{6}]}{a + b[\mathbf{6}]}} = \frac{a}{\frac{1}{[\mathbf{6}]} - \frac{b}{(a + b[\mathbf{6}])}} \qquad \text{(Eq S11)}$$

$$-d[\mathbf{6}]\frac{1}{[\mathbf{6}]} + d[\mathbf{6}]\frac{b}{(a + b[\mathbf{6}])} = adt \qquad \text{(Eq S12)}$$

$$\int_{[\mathbf{6}]_0}^{[\mathbf{6}]_t} d[\mathbf{6}]\frac{1}{[\mathbf{6}]} - b\int_{[\mathbf{6}]_0}^{[\mathbf{6}]_t} d[\mathbf{6}]\frac{1}{(a + b[\mathbf{6}])} = -a\int_o^t dt \qquad \text{(Eq S13)}$$

$$ln[\mathbf{6}]_t - ln[\mathbf{6}]_0 - \frac{b}{b}ln(a + b[\mathbf{6}]_t) + \frac{b}{b}ln(a + b[\mathbf{6}]_0) = -at \qquad \text{(Eq S14)}$$

$$ln\frac{[\mathbf{6}]_t}{[\mathbf{6}]_0} + ln(\frac{a + b[\mathbf{6}]_0}{a + b[\mathbf{6}]_t}) = ln\left(\frac{[\mathbf{6}]_t}{[\mathbf{6}]_0}\,\frac{a + b[\mathbf{6}]_0}{a + b[\mathbf{6}]_t}\right) = -at \qquad \text{(Eq S15)}$$

$$\frac{[\mathbf{6}]_t}{[\mathbf{6}]_0}\,\frac{a + b[\mathbf{6}]_0}{a + b[\mathbf{6}]_t} = e^{-at} \qquad \text{(Eq S16)}$$

$$[\mathbf{6}]_t\,\frac{a + b[\mathbf{6}]_0}{a + b[\mathbf{6}]_t} = [\mathbf{6}]_0 e^{-at} \qquad \text{(Eq S17)}$$

$$[\mathbf{6}]_t(a + b[\mathbf{6}]_0) = (a + b[\mathbf{6}]_t)[\mathbf{6}]_0 e^{-at} \qquad \text{(Eq S18)}$$

$$[\mathbf{6}]_t(a + b[\mathbf{6}]_0) = a[\mathbf{6}]_0 e^{-at} + b[\mathbf{6}]_t[\mathbf{6}]_0 e^{-at} \qquad \text{(Eq S19)}$$

$$(a + b[\mathbf{6}]_0) = \frac{a[\mathbf{6}]_0}{[\mathbf{6}]_t}e^{-at} + b[\mathbf{6}]_0 e^{-at} \qquad \text{(Eq S20)}$$

$$(a + b[\mathbf{6}]_0 - b[\mathbf{6}]_0 e^{-at}) = \frac{a[\mathbf{6}]_0}{[\mathbf{6}]_t}e^{-at} \qquad \text{(Eq S21)}$$

$$[\mathbf{6}]_t = \frac{a[\mathbf{6}]_0 e^{-at}}{(a + b[\mathbf{6}]_0 - b[\mathbf{6}]_0 e^{-at})}$$ (Eq S22)

$$[\mathbf{2}]_t = 0.5[\mathbf{6}]_0 - \frac{0.5\, a[\mathbf{6}]_0 e^{-at}}{(a + b[\mathbf{6}]_0 - b[\mathbf{6}]_0 e^{-at})}$$ (Eq S23)

$$[\mathbf{2}]_t = 0.5[\mathbf{6}]_0 - \frac{0.5}{\frac{a}{a[\mathbf{6}]_0 e^{-at}} + \frac{b[\mathbf{6}]_0}{a[\mathbf{6}]_0 e^{-at}} - \frac{b[\mathbf{6}]_0 e^{-at}}{a[\mathbf{6}]_0 e^{-at}}}$$ (Eq S24)

$$[\mathbf{2}]_t = 0.5[\mathbf{6}]_0 - \frac{0.5}{e^{at}\left(\frac{1}{[\mathbf{6}]_0} + \frac{b}{a}\right) - \frac{b}{a}}$$; where $rate = a[\mathbf{6}]^1 + b[\mathbf{6}]^2$ (Eq S25)

S8.3 Steady state rate approximations for direct, chloride accelerated, and $ZnCl_2$-accelerated homocoupling of Ar-Ni complex 6; with excess ligand (L); equation 14 in main manuscript.

The approximation below is based on [L_2Ni(Ar)Cl] complex **6** being in rapid equilibrium ($K_{\text{-L}}$) with [LNi(Ar)Cl] complex **8**, see equation S5, with the concentration of any associated dinuclear complex being vanishingly small. Under these conditions, equation S5 can be simplified to equation S26; the exact (standard quadratic) solution to this approximation is equation S27. The condition that $[L]_{exo}$ >> $[\mathbf{6}]_0$ applies throughout the analyses of the kinetic data. The effects of chloride and $ZnCl_2$, when both are present, are mutually antagonistic, equation S28. Only the species in excess (equations S29 and S30) is employed in the final holistic equation S43. The latter is developed by combining a direct ($K_A k_m$) and chloride ion catalyzed ($K'_A k'_m[Cl_-]_f$) homocoupling of [LNi(Ar)Cl] complex **8**, with a reversible Ni-to-Zn transmetallation (k_{TM1}, $k_{TM\text{-}1}$) coupled to irreversible Zn-to-Ni transmetallation (k_{TM2}); equations S31-33. Exogenous $NiCl_2$ generates [**17**] which acts as an inhibitor for this process (accelerating the competing Zn-to-Ni transmetallation, $k_{TM\text{-}1}$). The ligation state of $NiCl_2$ is not accounted for in this empirical analysis. The concentration of active $[ZnCl_2]_f$ takes into account mixed metallate generation (equation S39); the latter also reduces the availability of the inhibitor, [*exog*-**17**]$_f$. Endogenous [*endo*-**17**]$_f$ is primarily consumed in comproportionation with nascent Ni^0, equation S34; however, to allow solution to equation S37, [*endo*-**17**] is set to 0.5 mM. Equation S43 can display kinetic order in $[\mathbf{6}]^n$ spanning $1 \leq n \leq 2$; see limiting equations S45 and S46.

$$[\mathbf{8}] = \frac{K_L[\mathbf{6}]}{[L]} \approx \frac{[\mathbf{6}]_0 - 2[\mathbf{2}]}{\left(1 + \frac{[L]}{K_L}\right)}$$ (Eq S26)

$$[\mathbf{8}] \approx \frac{\sqrt{(K_L + [L]_{exo})^2 + 4K_L[\mathbf{6}]} - (K_L + [L]_{exo})}{2}$$ (Eq S27)

$$Cl^- + ZnCl_2 \rightleftarrows ZnCl_3{}^- ;\ where\ K_{Cl} = \frac{[ZnCl_3(DMF)^-]}{[Cl^-]_f[ZnCl_2]_f} \geq 2 \times 10^5\ \mathrm{M}^{-1}$$ (Eq S28)

$$[Cl^-]_f \approx [Cl^-]_0 - [ZnCl_2]_0\ when\ \frac{[Cl^-]_0}{[ZnCl_2]_0} > 1$$ (Eq S29)

$$[ZnCl_2]_f \approx [ZnCl_2]_0 - [Cl^-]_0\ when\ \frac{[ZnCl_2]_0}{[Cl^-]_0} > 1$$ (Eq S30)

$$[\mathbf{8}] + ZnCl_2 \rightarrow ArZnCl\,(\mathbf{16}) + NiCl_2\,(\mathbf{17});\ \ Ni\ to\ Zn\ transmet.(k_{TM1}) \quad \text{(Eq S31)}$$

$$[\mathbf{8}] + ArZnCl(\mathbf{16}) \rightarrow Ni^0(L)_1 + Ar\text{–}Ar(\mathbf{2}) + ZnCl_2;\ Zn\ to\ Ni\ transmet.(k_{TM2}) \quad \text{(Eq S32)}$$

$$ArZnCl\,(\mathbf{16}) + NiCl_2\,(\mathbf{17}) \rightarrow [\mathbf{8}] + ZnCl_2;\ \ Zn\ to\ Ni\ transmet.(k_{TM-1}) \quad \text{(Eq S33)}$$

$$Ni^0(L)_n + (L)_2NiCl_2\,(\mathbf{17}) + L \rightarrow 2\,(L)_3NiCl\,(\mathbf{7});\ K_{CD}\ large \quad \text{(Eq S34)}$$

$$[Cl^-]_f \approx [Cl^-]_0 - [ZnCl_2]_0\ when\ \frac{[Cl^-]_0}{[ZnCl_2]_0} > 1 \quad \text{(Eq S35)}$$

$$[ZnCl_2]_f \approx [ZnCl_2]_0 - [Cl^-]_0\ when\ \frac{[ZnCl_2]_0}{[Cl^-]_0} > 1 \quad \text{(Eq S36)}$$

$$ZnCl_2 + [\mathbf{17}] \rightleftarrows [ZnCl_3(DMF)][(DMF)_5NiCl]\,(\mathbf{20}_{\mathrm{DMF}}) \quad \text{(Eq S37)}$$

$$K'_{\mathrm{M1}} = \frac{[\mathbf{20}_{\mathrm{DMF}}]}{[ZnCl_2][\mathbf{17}]} \quad \text{(Eq S38)}$$

$$[exog\mathbf{17}]_f \approx \frac{\sqrt{(K'_{-M1}+[ZnCl_2]_0-[NiCl_2]_0)^2+4K'_{-M1}[NiCl_2]_0}-(K'_{-M1}+[ZnCl_2]_0-[NiCl_2]_0)}{2} \quad \text{(Eq S39)}$$

$$rate = \frac{-d[\mathbf{6}]}{dt} \approx (K_A k_m + (K'_A k'_m [Cl^-]_f)[\mathbf{8}]^2\ when\ \frac{[Cl^-]_0}{[ZnCl_2]_0} > 1 \quad \text{(Eq S40)}$$

$$\frac{-d[\mathbf{6}]}{dt} \approx \frac{k_{TM2}[\mathbf{8}]k_{TM1}[\mathbf{8}][ZnCl_2]_f}{k_{TM2}[\mathbf{8}] + k_{TM-1}[\mathbf{17}]_f} when\ \frac{[ZnCl_2]_0}{[Cl^-]_0} > 1 \quad \text{(Eq S41)}$$

$$\frac{-d[\mathbf{6}]}{dt} \approx [\mathbf{8}]^2 \frac{[ZnCl_2]_f}{\frac{k_{TM1}[\mathbf{8}]}{k_{TM-1}} + \frac{k_{TM-1}[\mathbf{17}]_f}{k_{TM1}k_{TM2}}};\ when\ \frac{[ZnCl_2]_0}{[Cl^-]_0} > 1 \quad \text{(Eq S42)}$$

$$\frac{-d[\mathbf{6}]}{dt} \approx [\mathbf{8}]^2 \left[(K_A k_m + K'_A k'_m [Cl^-]_f)\ or \frac{[ZnCl_2]_f}{\frac{[\mathbf{8}]}{k_{TM2}} + \frac{k_{TM-1}[\mathbf{17}]_f}{k_{TM1}k_{TM2}}} \right] exclusive \quad \text{(Eq S43)}$$

$$\frac{-d[\mathbf{6}]}{dt} \approx \frac{[\mathbf{6}]^2}{\left(1+\frac{2[L]}{K_L}+\frac{[L]^2}{(K_L)^2}\right)} \left[\frac{[ZnCl_2]_f}{\frac{\frac{[\mathbf{6}]}{\left(1+\frac{[L]}{K_L}\right)}}{k_{TM2}} + \frac{k_{TM-1}[\mathbf{17}]_f}{k_{TM1}k_{TM2}}} \right];\ when\ \frac{[ZnCl_2]_0}{[Cl^-]_0} > 1 \quad \text{(Eq S44)}$$

$$as\ [L]\ and\ [exog\mathbf{17}]_f\ raised; \frac{-d[\mathbf{6}]}{dt} \rightarrow \frac{[\mathbf{6}]^2}{\left(1+\frac{2[L]}{K_L}+\frac{[L]^2}{(K_L)^2}\right)} \left[\frac{[ZnCl_2]_f}{\frac{[\mathbf{17}]_f}{K_{TM1}k_{TM2}}} \right] when\ \frac{[ZnCl_2]_0}{[Cl^-]_0} > 1 \quad \text{(Eq S45)}$$

$$as\ [L]\ and\ [exog\mathbf{17}]_f\ reduced; \frac{-d[\mathbf{6}]}{dt} \rightarrow \frac{[\mathbf{6}]}{\left(1+\frac{[L]}{K_L}\right)} \left[\frac{[ZnCl_2]_f}{\frac{1}{k_{TM2}}} \right] when\ \frac{[ZnCl_2]_0}{[Cl^-]_0} > 1 \quad \text{(Eq S46)}$$

S8.4 Kinetic data and analysis.

Table S52 - Stoichiometric homocoupling of $[(L)_2Ni(Ar)Cl]$ (**6**) experimental concentrations, calculated concentrations, empirical first and second order fitting constants for primary data, and rates calculated at [**6**] = 0.01 M from equations S25 and S34, Figure 31 in main manuscript.

entry	$[ZnCl_2]_0$ mM	$[NiCl_2]_0$ mM	$[Cl^-]_0$ mM	$[L]_f$ mM	$[ZnCl_2]_f$[i] mM	[**17**]$_f$[ii] mM	$[Cl^-]_f$[iii] mM	[**8**]$_{calc}$[iv] μM	a[v] s^{-1} /10^{-3}	b[vi] Ms^{-1} /10^{-2}	rate$_{obs.}$[vii] Ms^{-1} /10^{-5}	rate $_{calc.}$[viii] Ms^{-1} /10^{-5}
1	30.4	0.0	0.0	**108**	29.7	0.08	0.0	18.5	8.1	10.0	9.1	9.1
2	30.4	0.0	0.0	**109**	29.7	0.08	0.0	18.3	8.0	9.0	8.9	8.9
3	30.4	0.0	0.0	**162**	29.7	0.08	0.0	12.3	4.4	13.2	5.7	5.7
4	30.4	0.0	0.0	**216**	29.7	0.08	0.0	9.3	2.0	19.3	3.9	3.9
5	30.4	0.0	0.0	**316**	29.7	0.08	0.0	6.3	1.3	13.7	2.7	2.4
6	30.4	0.0	0.0	**555**	29.7	0.08	0.0	3.6	0.3	12.0	1.5	1.0
7	30.4	**0.0**	0.0	162	29.7	0.08	0.0	12.3	4.4	13.2	5.7	5.7
8	30.4	**3.6**	0.0	158	26.6	0.51	0.0	12.3	1.0	10.0	2.0	2.0
9	30.4	**7.2**	0.0	155	23.5	1.03	0.0	12.3	0.5	6.0	1.1	1.1
10	30.4	**10.8**	0.0	151	20.6	1.68	0.0	12.3	0.5	0.7	0.6	0.6
11	30.4	**14.4**	0.0	148	17.8	2.49	0.0	12.3	0.14	1.7	0.3	0.4
12	31.1	**17.5**	0.0	144	16.1	3.25	0.0	12.3	.03	3.8	0.4	0.3
13	**3.1**	0.0	0.0	162	2.8	0.42	0.0	12.3	--[ix]	3.3	0.3	0.3
14	**6.2**	0.0	0.0	162	5.7	0.28	0.0	12.3	--[ix]	7.9	0.8	0.7
15	**15.4**	0.0	0.0	162	14.8	0.14	0.0	12.3	1.0	20.0	3.0	2.5
16	**30.8**	0.0	0.0	162	30.1	0.08	0.0	12.3	6.0	--[x]	6.0	5.9
17	**46.7**	0.0	0.0	162	46.0	0.05	0.0	12.3	9.6	--[x]	9.6	9.5
18	**61.6**	0.0	0.0	162	60.9	0.04	0.0	12.3	13.2	--[x]	13.2	13.0
19	0.0	0.0	**4.5**	158	0.6	0.63	4.5	12.6	--[ix]	47	4.7	4.5
20	0.0	0.0	**13.5**	158	0.6	0.63	13.5	12.6	--[ix]	137	13.7	13.3
21	0.0	0.0	**22.5**	158	0.6	0.63	22.5	12.6	--[ix]	221	22.1	22.3
22	**30.4**	0.0	**4.5**	158	25.2	0.09	0.0	12.6	3.4	5.0	3.9	4.7
23	**30.4**	0.0	**13.5**	158	16.3	0.13	0.0	12.6	0.9	15.6	2.5	2.9
24	**30.4**	0.0	**22.5**	158	7.4	0.24	0.0	12.6	0.2	8.9	1.0	1.0
25	0.0	0.0	0.0	**554**	0.0	0.00	0.0	3.6	--[ix]	1.0	0.1	0.1
26	0.0	0.0	0.0	**319**	0.0	0.00	0.0	6.3	--[ix]	2.0	0.2	0.2
27	0.0	0.0	0.0	**216**	0.0	0.00	0.0	9.3	--[ix]	4.4	0.4	0.5
28	0.0	0.0	0.0	**160**	0.0	0.00	0.0	12.5	--[ix]	7.7	0.8	0.8
29	0.0	0.0	0.0	**106**	0.0	0.00	0.0	18.8	--[ix]	20.2	2.0	1.9

i. The free $ZnCl_2$ concentration calculated from equations S29, S30 , S39. ii. Free $NiCl_2$ concentration calculated from equation S39, with endogeneous $[NiCl_2]$ set to 0.5 mM. iii. Chloride concentration calculated from equations S29, S30. iv. Concentration of [LNi(Ar)Cl] (**8**) calculated using equation S27. v. Empirical first order fitting constant, a, used in isolation (i.e. > 95 % first order if no b value cited) or in combination with b, via equation S9. vi. The empirical second order fitting constant, b, used in isolation (i.e. > 95 % second order if no a value cited) or in combination with a, via equation S9. vii. Rate calculated from one or both of a and b, when [**6**] = 0.01 M. viii. Rate calculated from equation S43 when [**6**] = 0.01 M. ix. Second order kinetics dominant. x. First order kinetics dominant.

The following parameters collectively give a satisfactory correlation between observed and calculated rates, Figure 31 in main manuscript, but are not unique solutions and should not be used in isolation.

$K_L = 2 \times 10^{-4}\ M^{-1}$ $(equation\ S27)$; $k_{TM2} = 2 \times 10^{2}\ \mathrm{M}^{-1} s^{-1}$; $\frac{1}{K_{TM1}k_{TM2}}\ 3 \times 10^{-4}\ \mathrm{Ms}$; $K_A k_m = 5 \times 10^{4}\ \mathrm{M}^{-1} s^{-1}$; $K'_A k'_m = 6 \times 10^{7}\ \mathrm{M}^{-1} s^{-1}$ $(equation\ S43)$; $K'_{-M1} = 2.9 \times 10^{2}\ \mathrm{M}$; $(equation\ S39)$

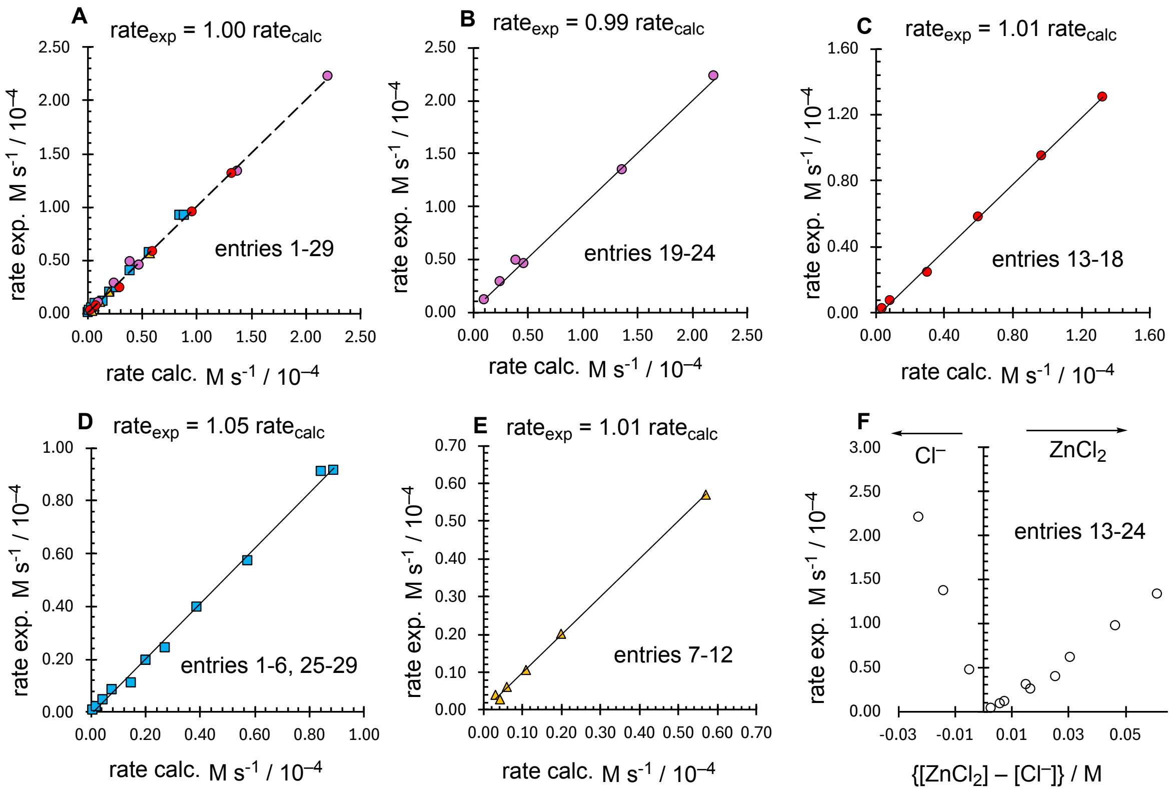

Figure S63 - Selected linear correlations of experimental versus calculated rate data from Table S52. **A**. All entries (1-2) combined. **B**. Variation in chloride ion (entries 19-24). **C**. Variation in $ZnCl_2$ (entries 13-18). **D**. Variation in excess phosphine ligand (entries 1-6 with $ZnCl_2$ and 25-29 without $ZnCl_2$). **E**. Variation in exogeneous [L_2NiCl_2] (**17**) (entries 7-12). **F** mutual antagonism of effects of chloride ion and [$ZnCl_2$] (entries 13-24), negative *x*-axis values are chloride excess, positive *x*-axis values are $ZnCl_2$ excess.

S9 IR Spectra

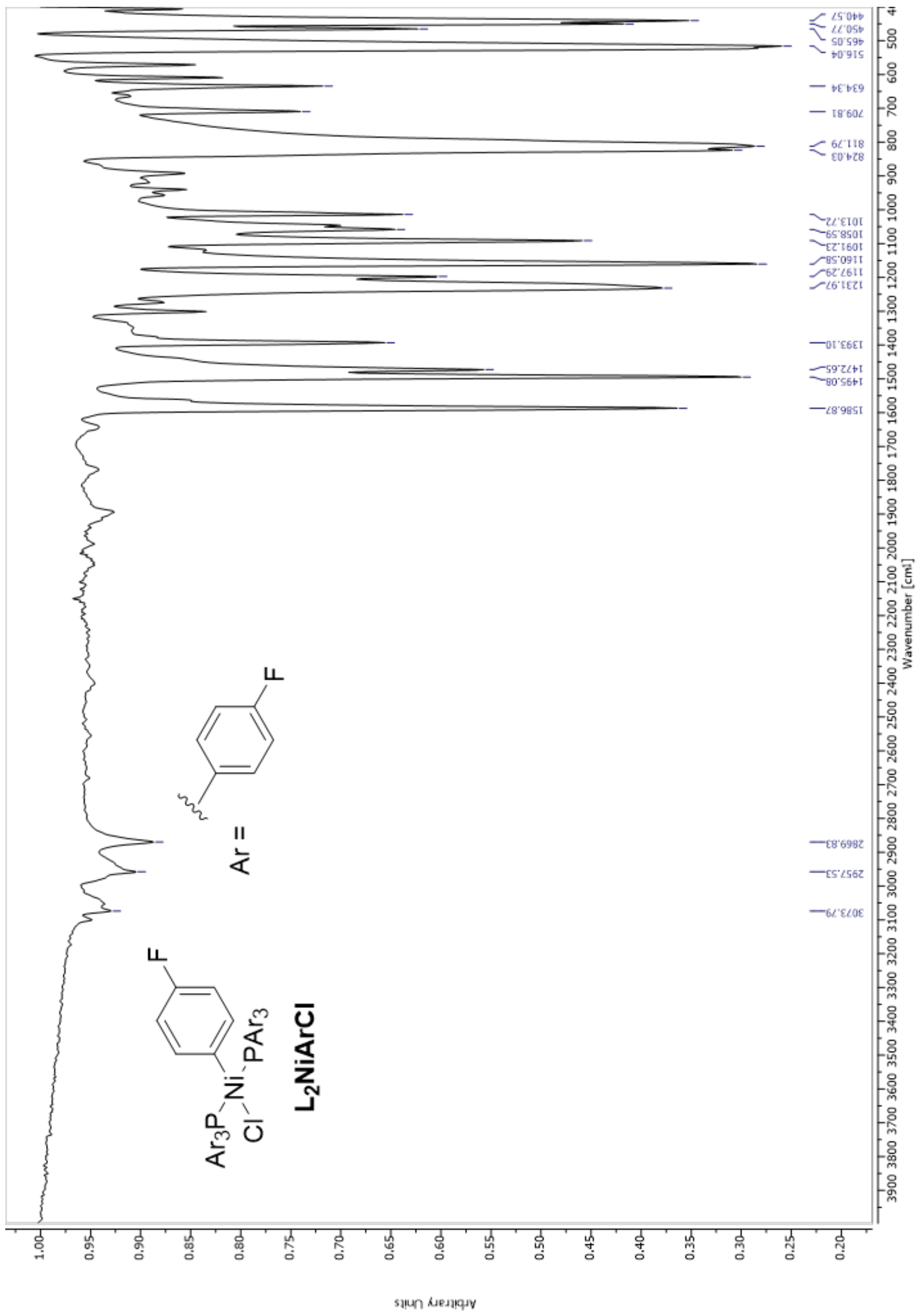

Ar3P Ni PAr3 Cl
F
L2NiArCl
Ar =
F
3073.79
2957.53
2869.83
1586.87
1495.08
1472.65
1393.10
1231.97
1197.29
1160.58
1091.23
1058.59
1013.72
824.03
811.79
709.81
634.34
516.04
465.05
450.77
440.57
Wavenumber [cml]
Arbitrary Units

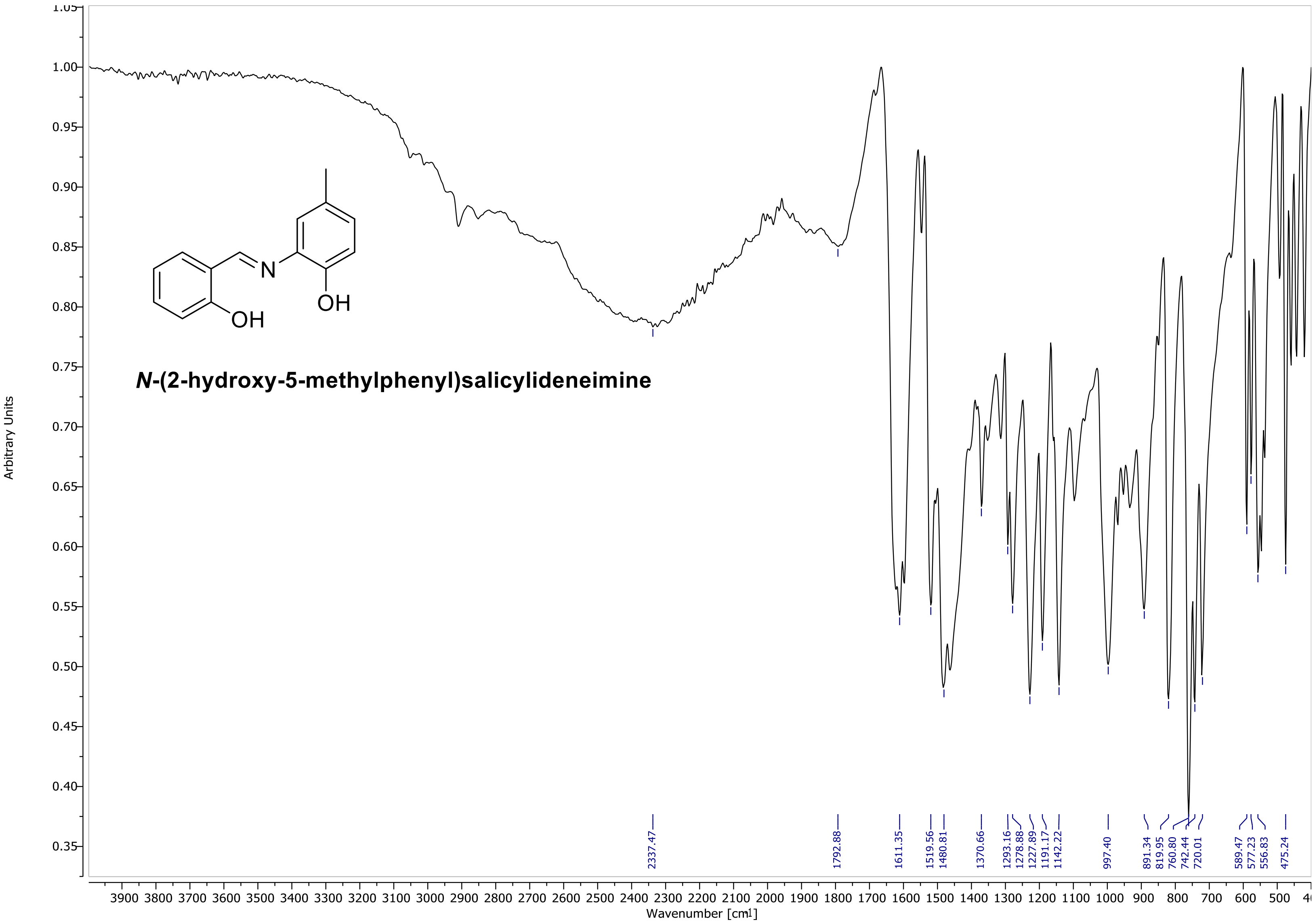

N-(2-hydroxy-5-methylphenyl)salicylideneimine
OH
N
OH
Arbitrary Units
Wavenumber [cm-1]
2337.47
1792.88
1611.35
1519.56
1480.81
1370.66
1293.16
1278.88
1227.89
1191.17
1142.22
997.40
891.34
819.95
760.80
742.44
720.01
589.47
577.23
556.83
475.24

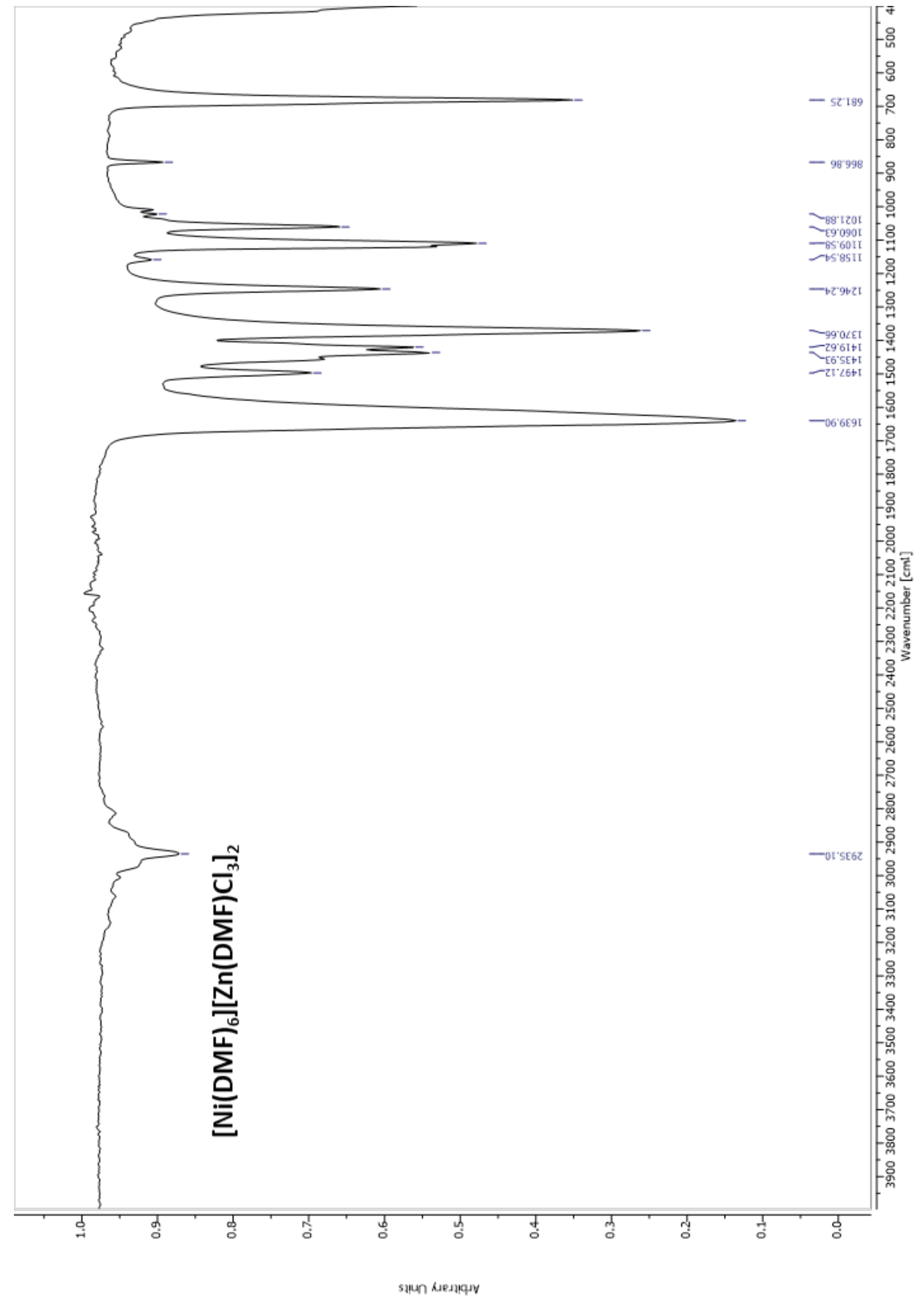

[Ni(DMF)$_6$][Zn(DMF)Cl$_3$]$_2$
681.25
866.86
1021.88
1060.63
1109.58
1158.54
1246.24
1370.66
1419.62
1435.93
1497.12
1639.90
2935.10
Wavenumber [cm-1]
Arbitrary Units

S10 NMR Spectra

The NMR spectra for $L_2NiArCl$ (**6**) were acquired in d_7-DMF in the presence of excess PAr_3.

^{1}H NMR (400 MHz, d_7-DMF, 300 K) with excess PAr_3 in solution

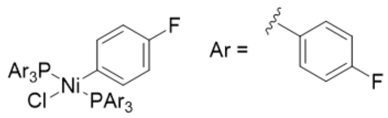

$L_2NiArCl$

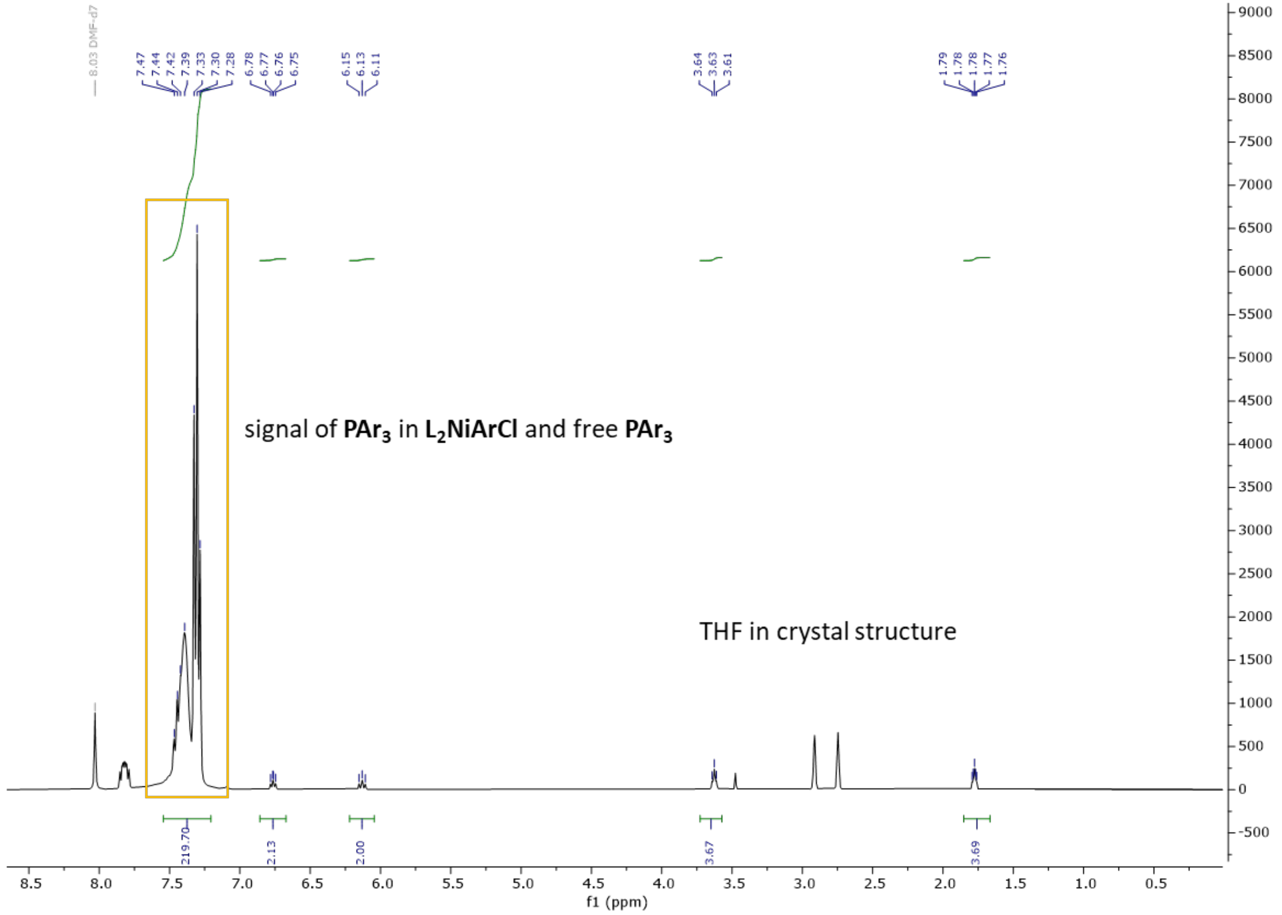

^{13}C {1H} NMR (101 MHz, d_7-DMF, 300 K) with excess PAr_3 in solution

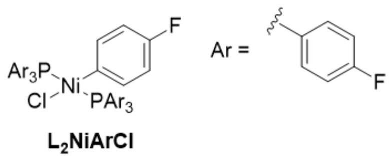

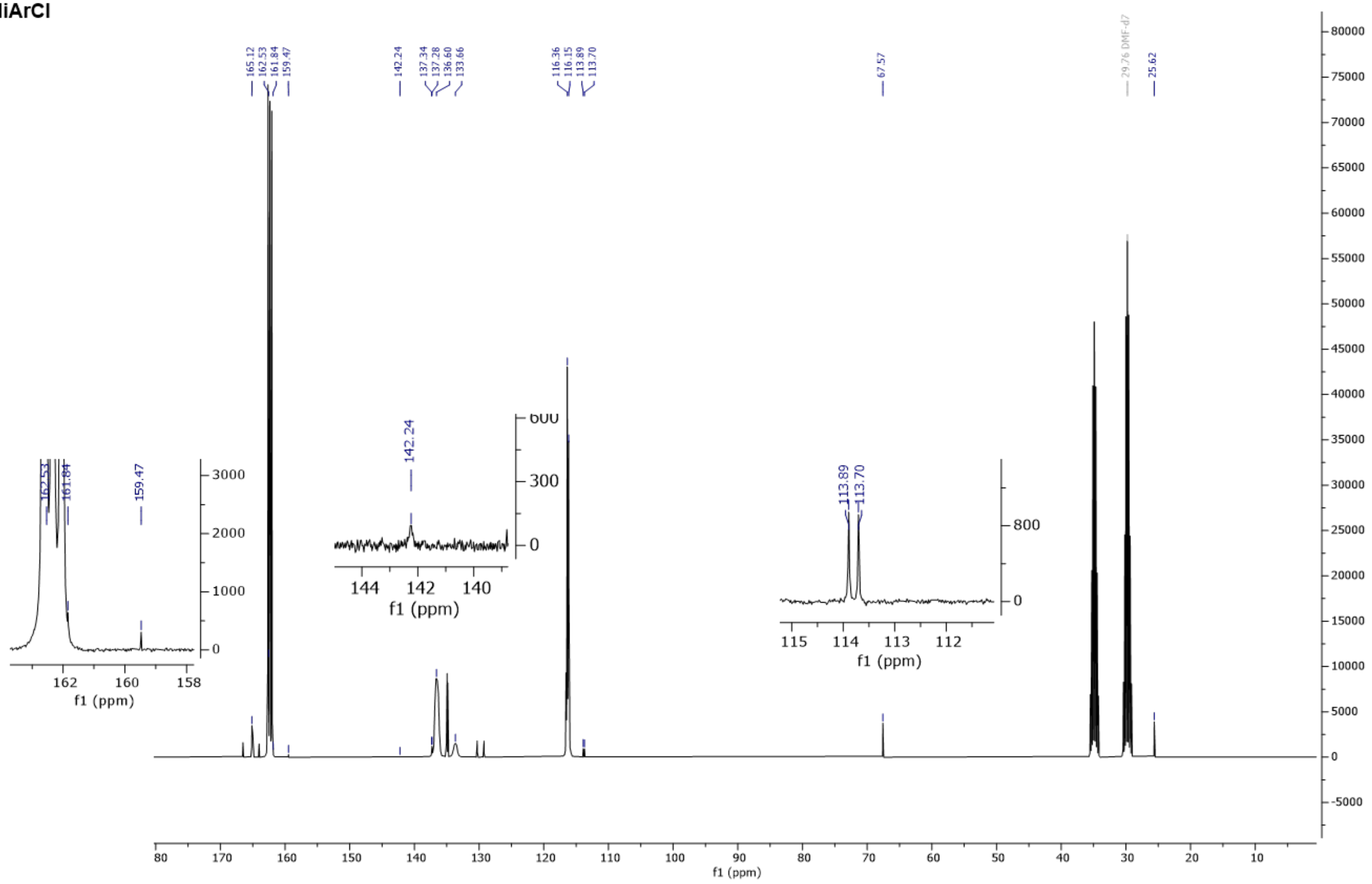

^{19}F NMR (377 MHz, d_7-DMF, 300 K) with excess PAr_3 in solution

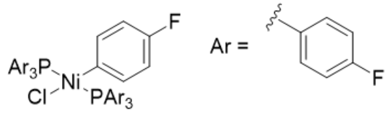

$L_2NiArCl$

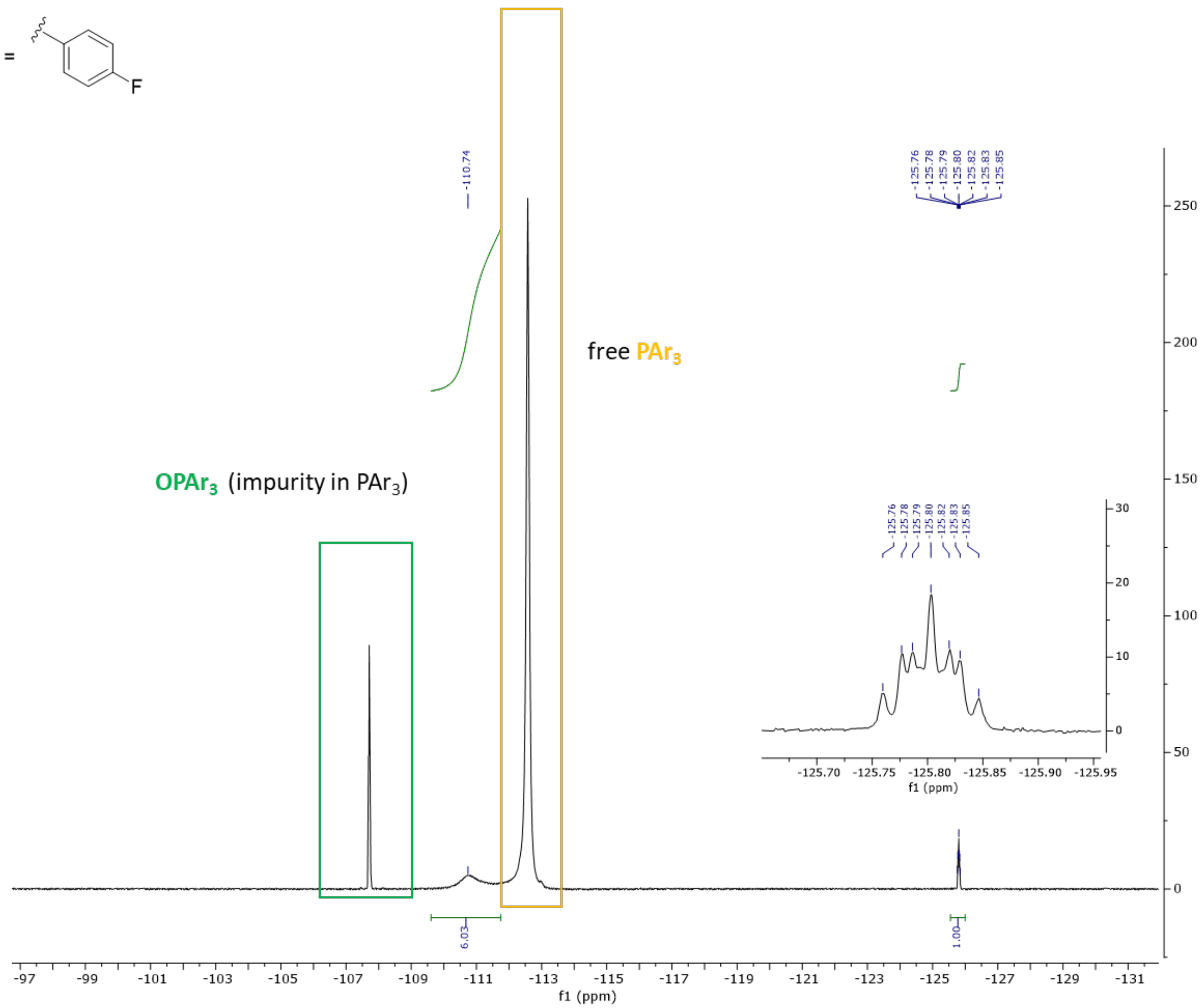

^{31}P {1H} NMR (162 MHz, d_7-DMF, 300 K) with excess PAr_3 in solution

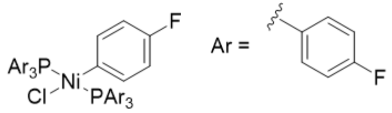

$L_2NiArCl$

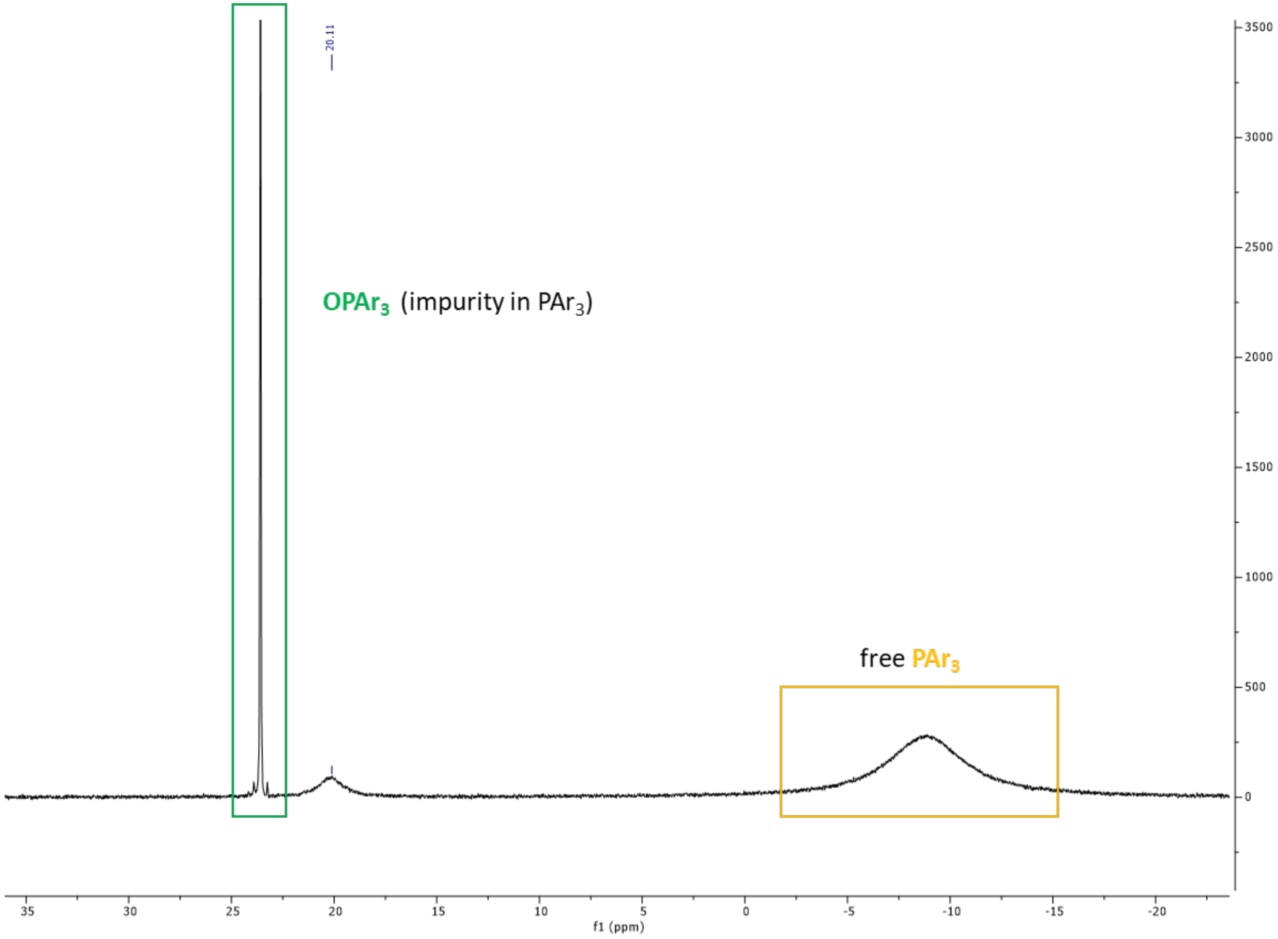

COSY (400 MHz, d_7-DMF, 300 K) with excess PAr_3 in solution

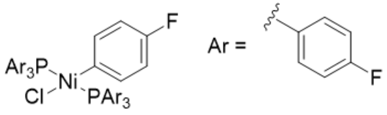

L_2NiArCl

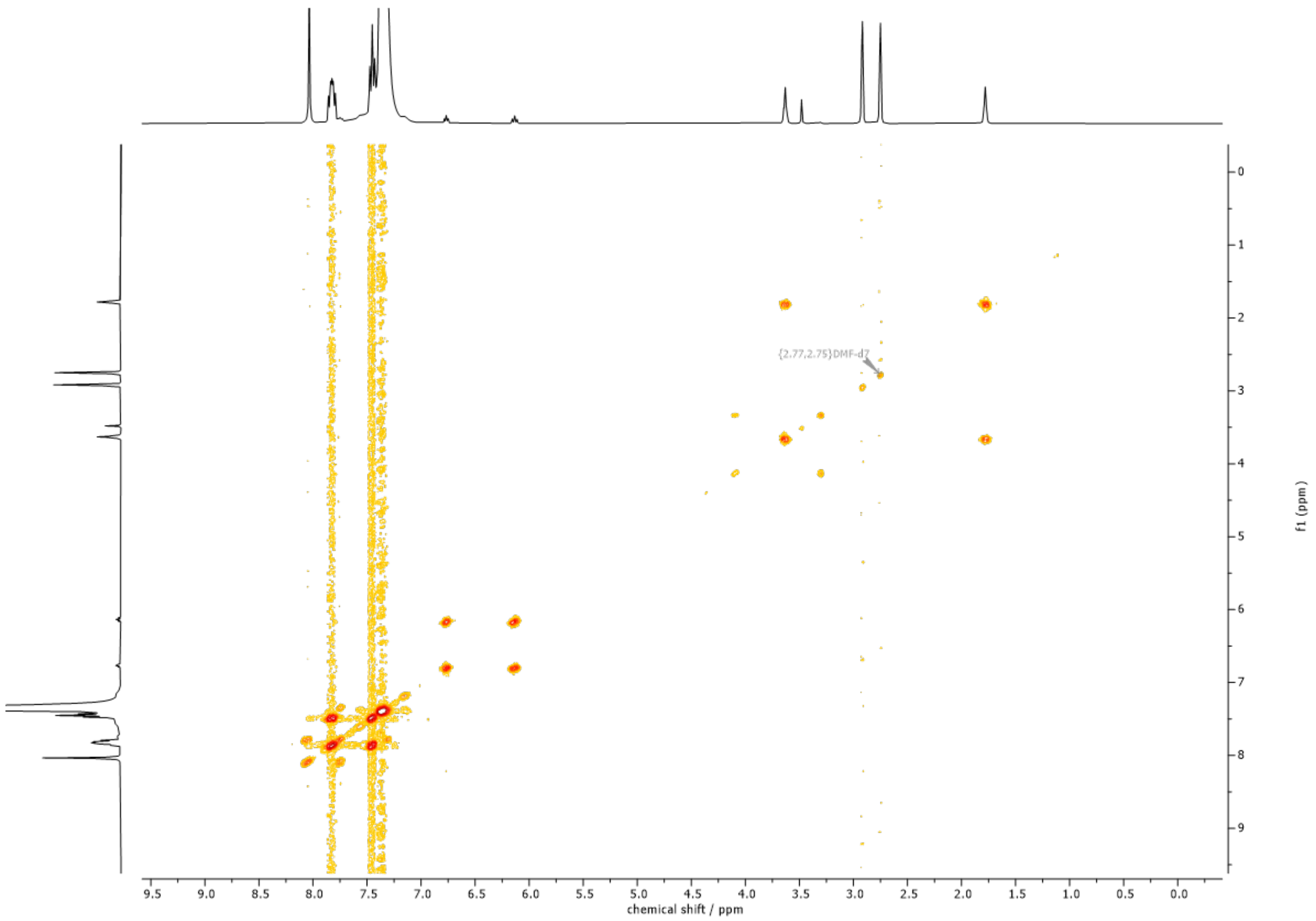

HSQC (400 MHz, d_7-DMF, 300 K) with excess PAr_3 in solution

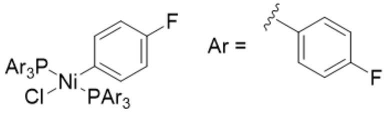

$L_2NiArCl$

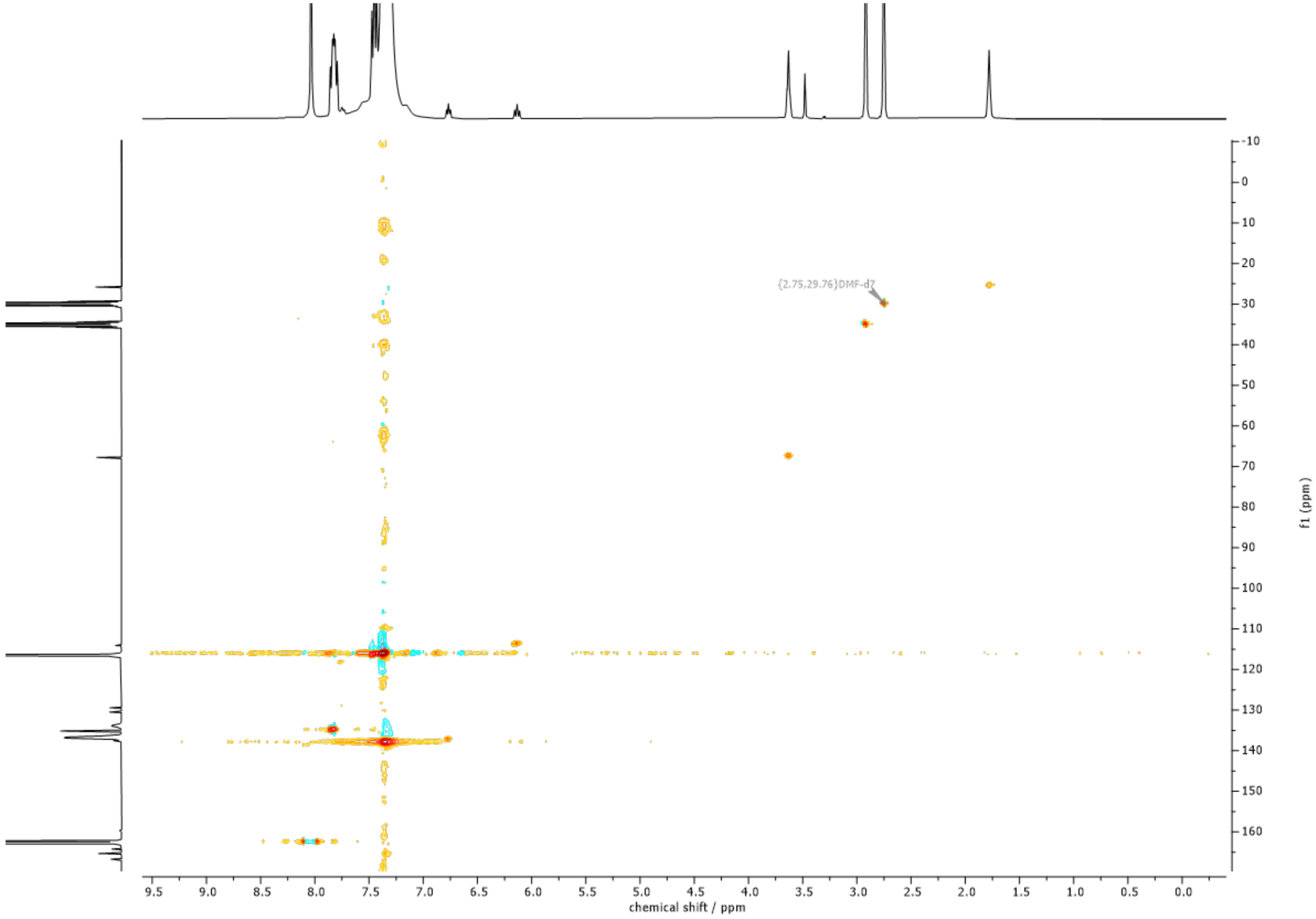

HMBC (400 MHz, d_7-DMF, 300 K) with excess PAr_3 in solution

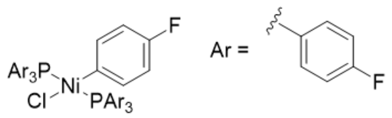

$L_2NiArCl$

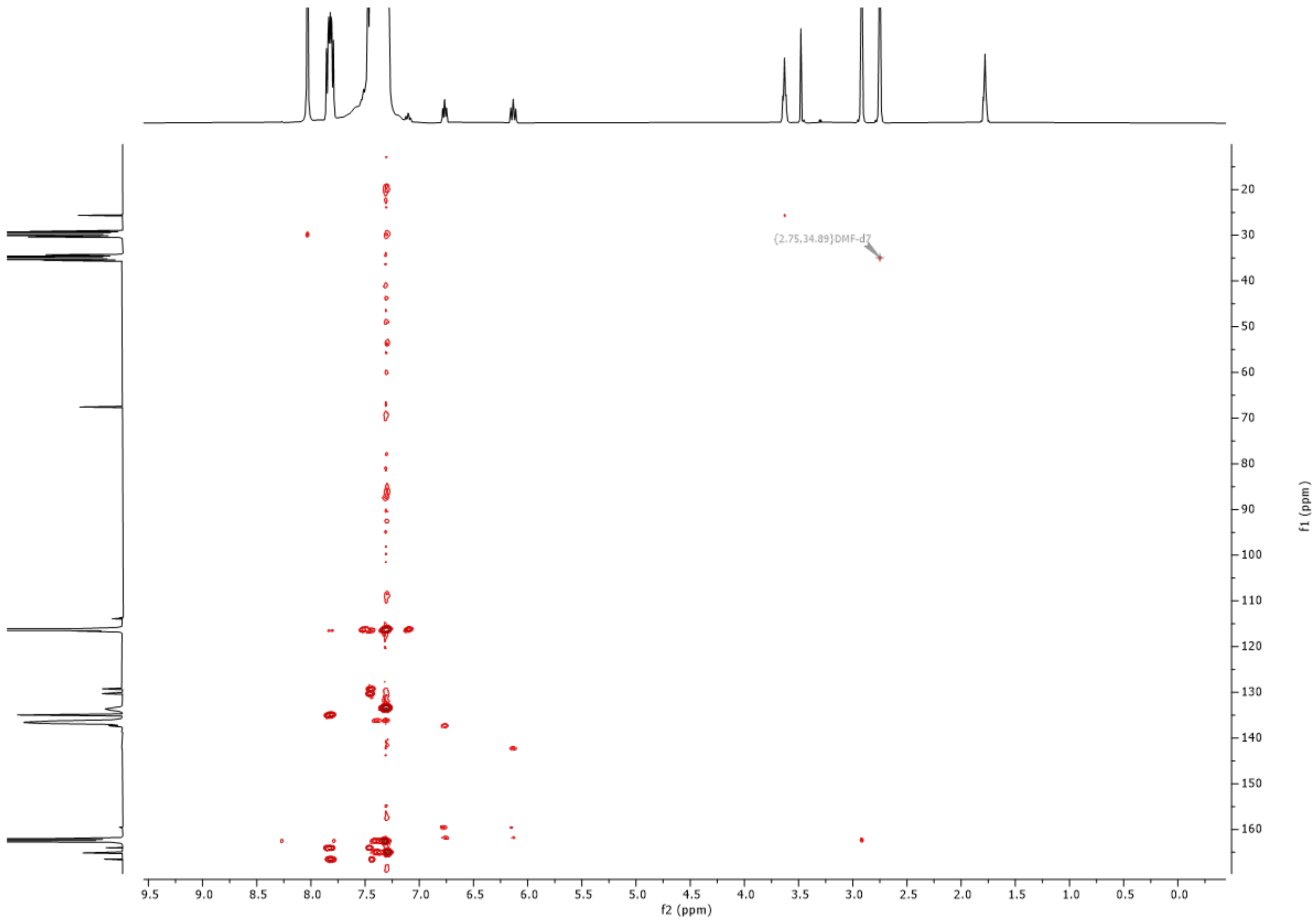

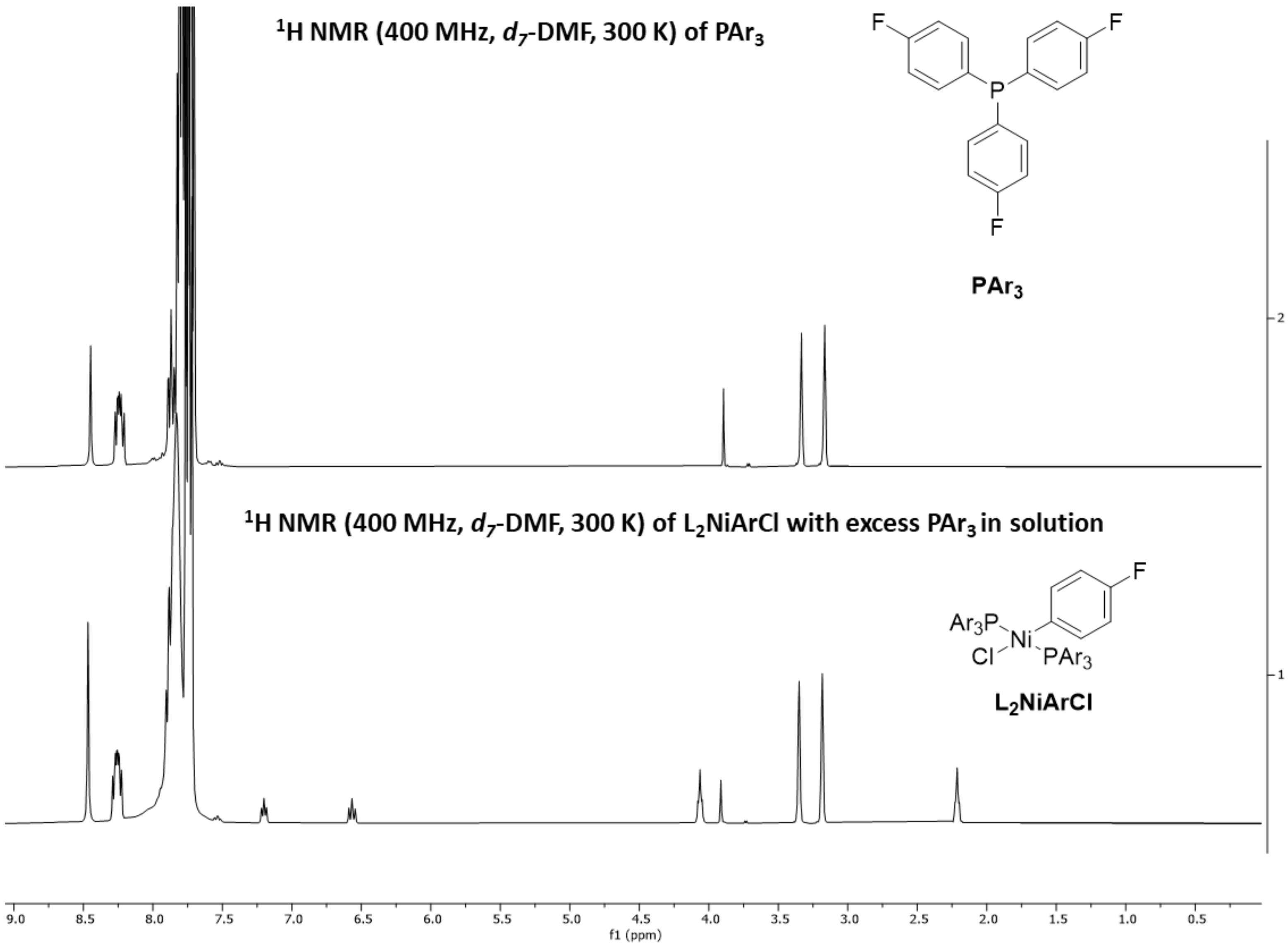

¹H NMR (400 MHz, d₇-DMF, 300 K) of PAr₃
PAr₃
¹H NMR (400 MHz, d₇-DMF, 300 K) of L₂NiArCl with excess PAr₃ in solution
L₂NiArCl
f1 (ppm)

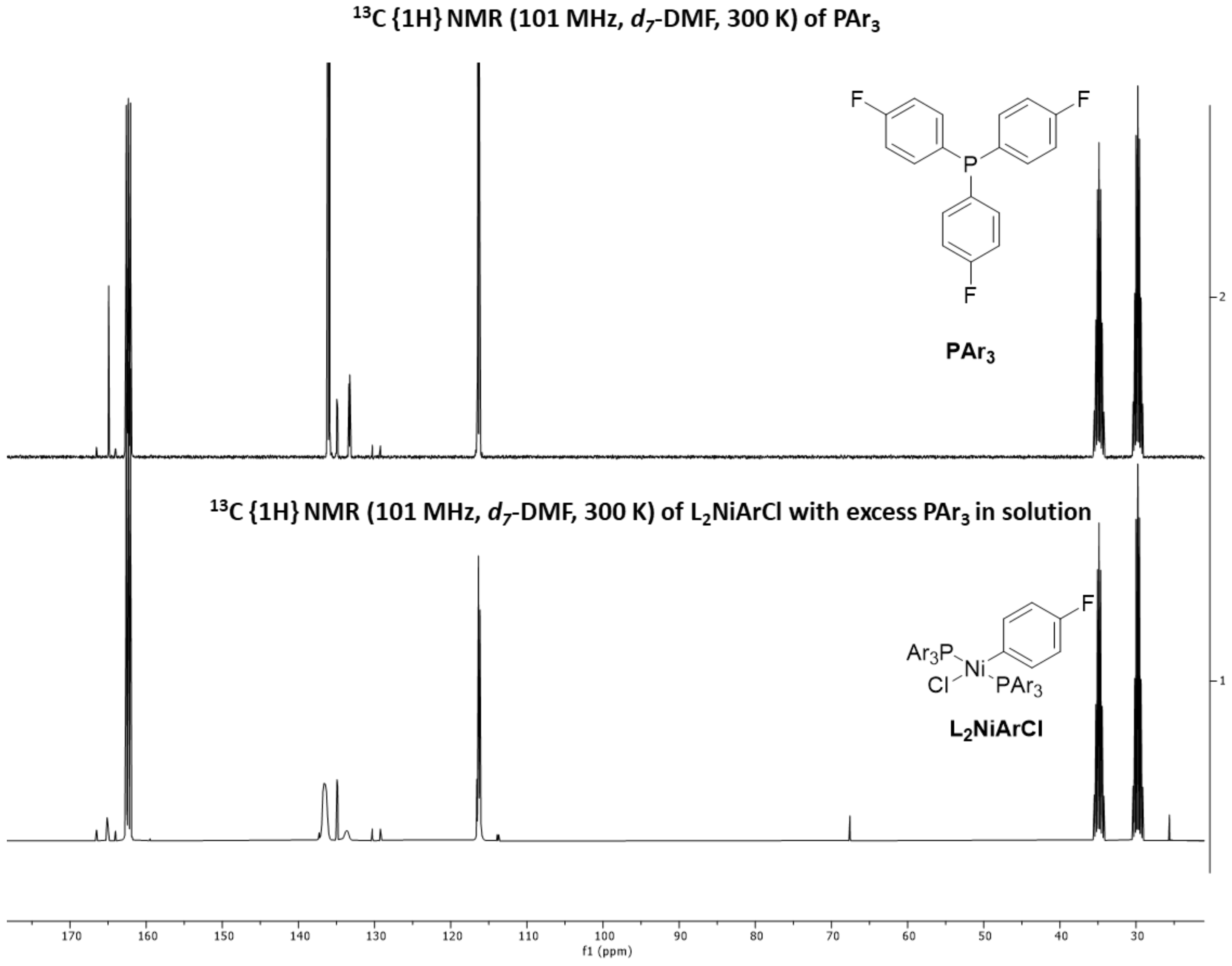
^{13}C {1H} NMR (101 MHz, d_7-DMF, 300 K) of PAr_3
PAr_3
^{13}C {1H} NMR (101 MHz, d_7-DMF, 300 K) of $L_2NiArCl$ with excess PAr_3 in solution
$L_2NiArCl$
f1 (ppm)

^{19}F NMR (377 MHz, d_7-DMF, 300 K) of PAr_3

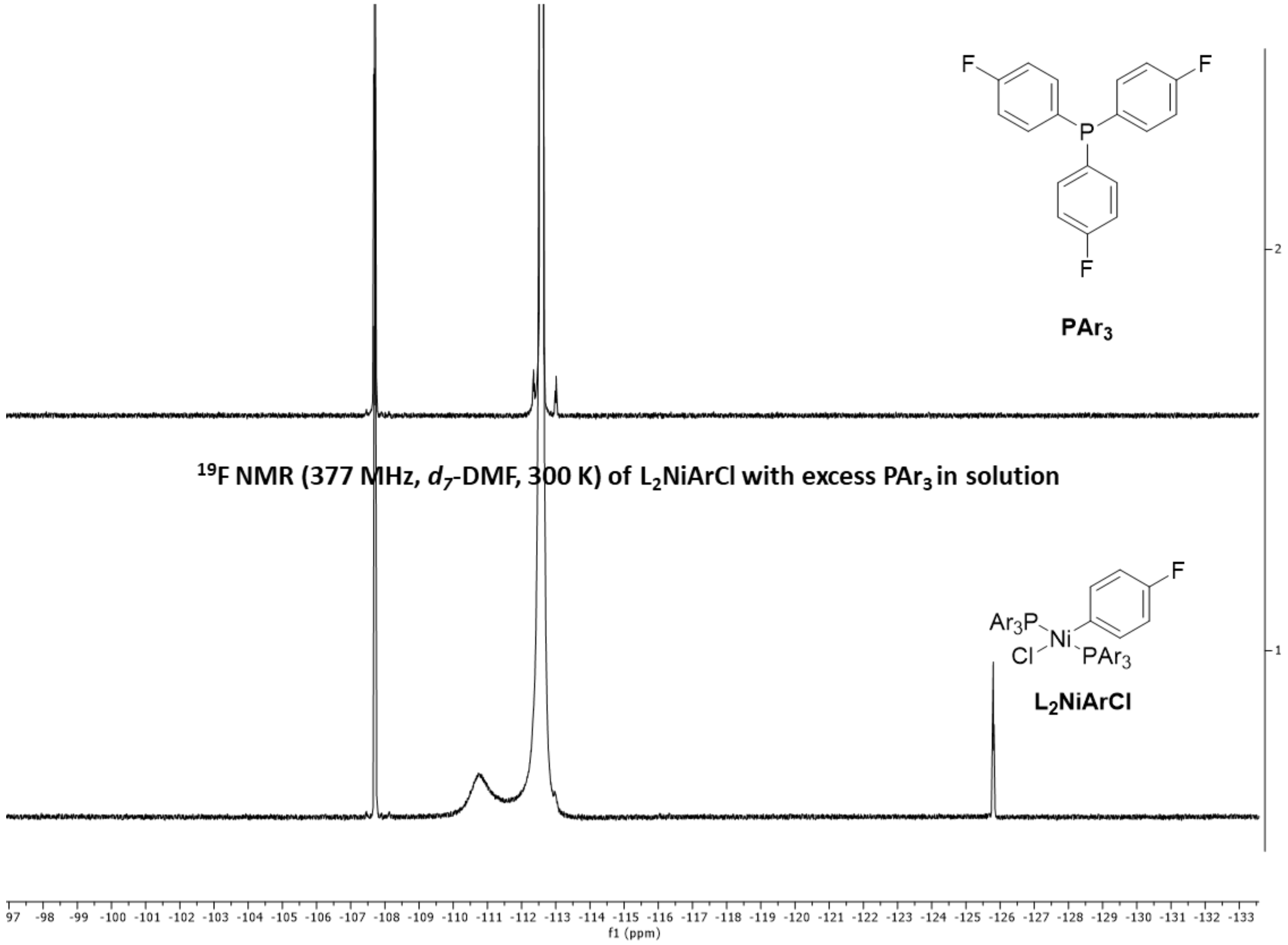

^{19}F NMR (377 MHz, d_7-DMF, 300 K) of $L_2NiArCl$ with excess PAr_3 in solution

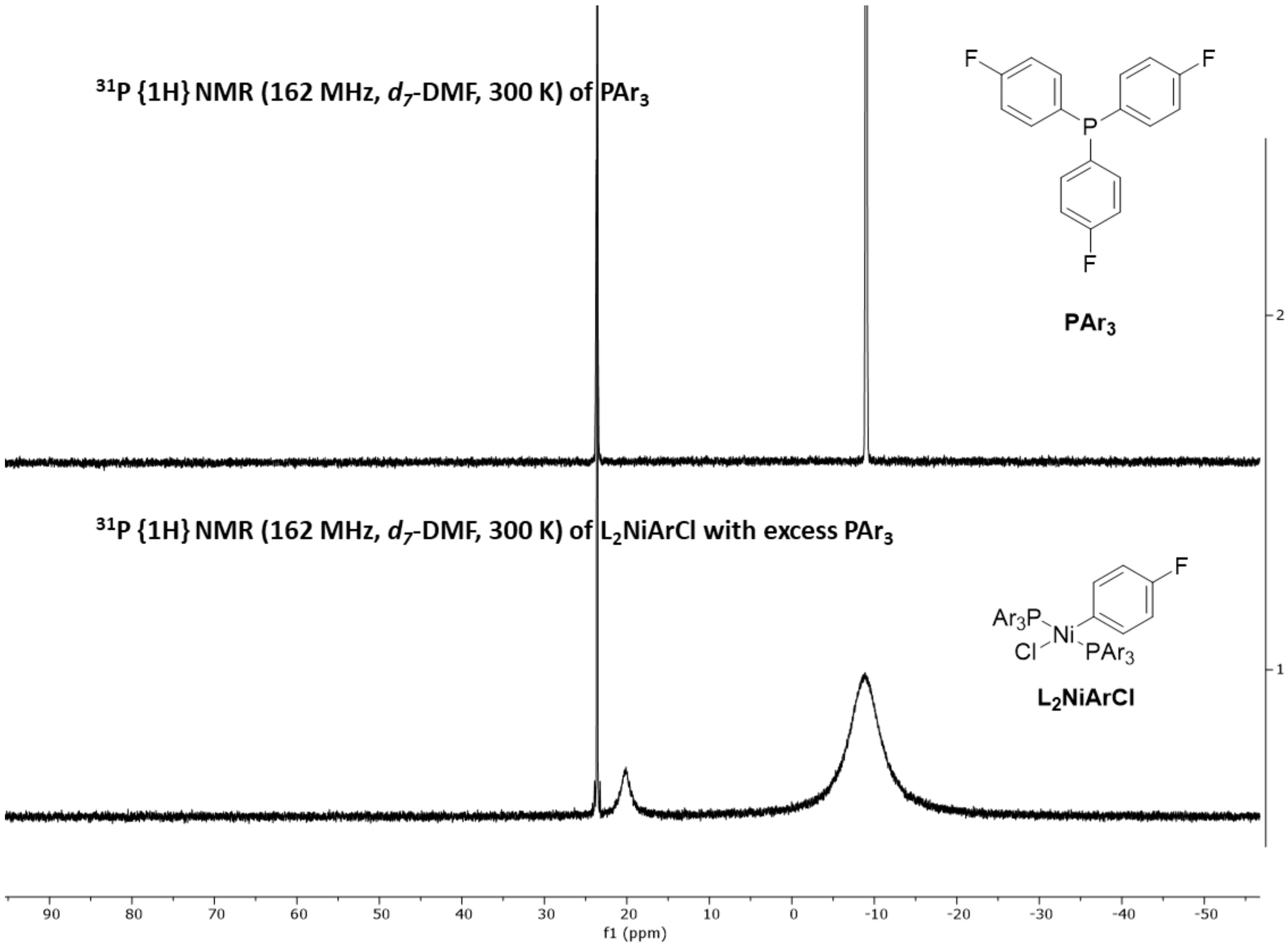
^{31}P {1H} NMR (162 MHz, d_7-DMF, 300 K) of PAr$_3$
PAr$_3$
^{31}P {1H} NMR (162 MHz, d_7-DMF, 300 K) of L$_2$NiArCl with excess PAr$_3$
L$_2$NiArCl
f1 (ppm)

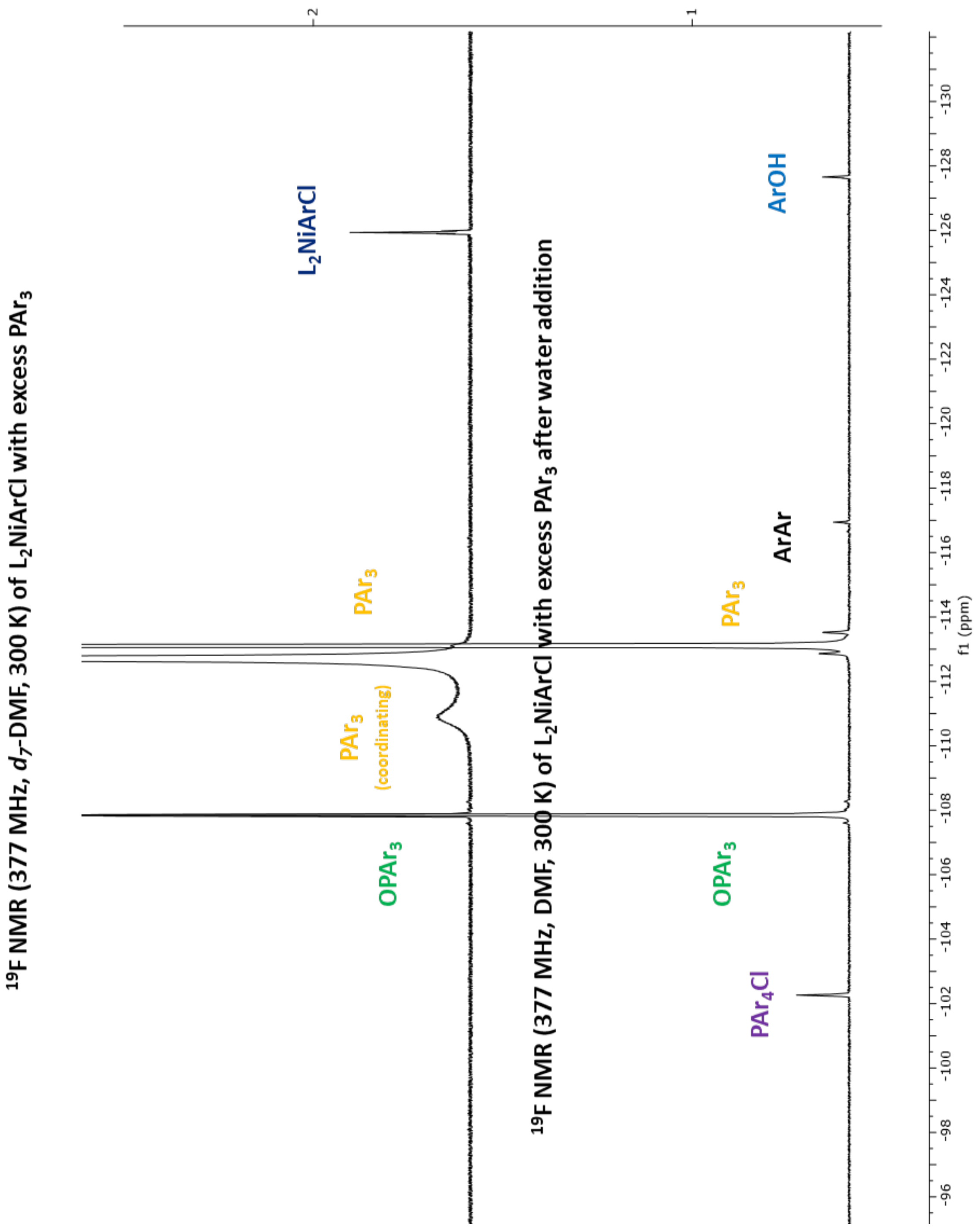

19F NMR (377 MHz, d7-DMF, 300 K) of L2NiArCl with excess PAr3
19F NMR (377 MHz, DMF, 300 K) of L2NiArCl with excess PAr3 after water addition
L2NiArCl
PAr3
PAr3 (coordinating)
OPAr3
ArOH
ArAr
PAr3
OPAr3
PAr4Cl
f1 (ppm)

^{31}P {1H} NMR (162 MHz, DMF, 300 K) of $L_2NiArCl$ with excess PAr_3 after water addition

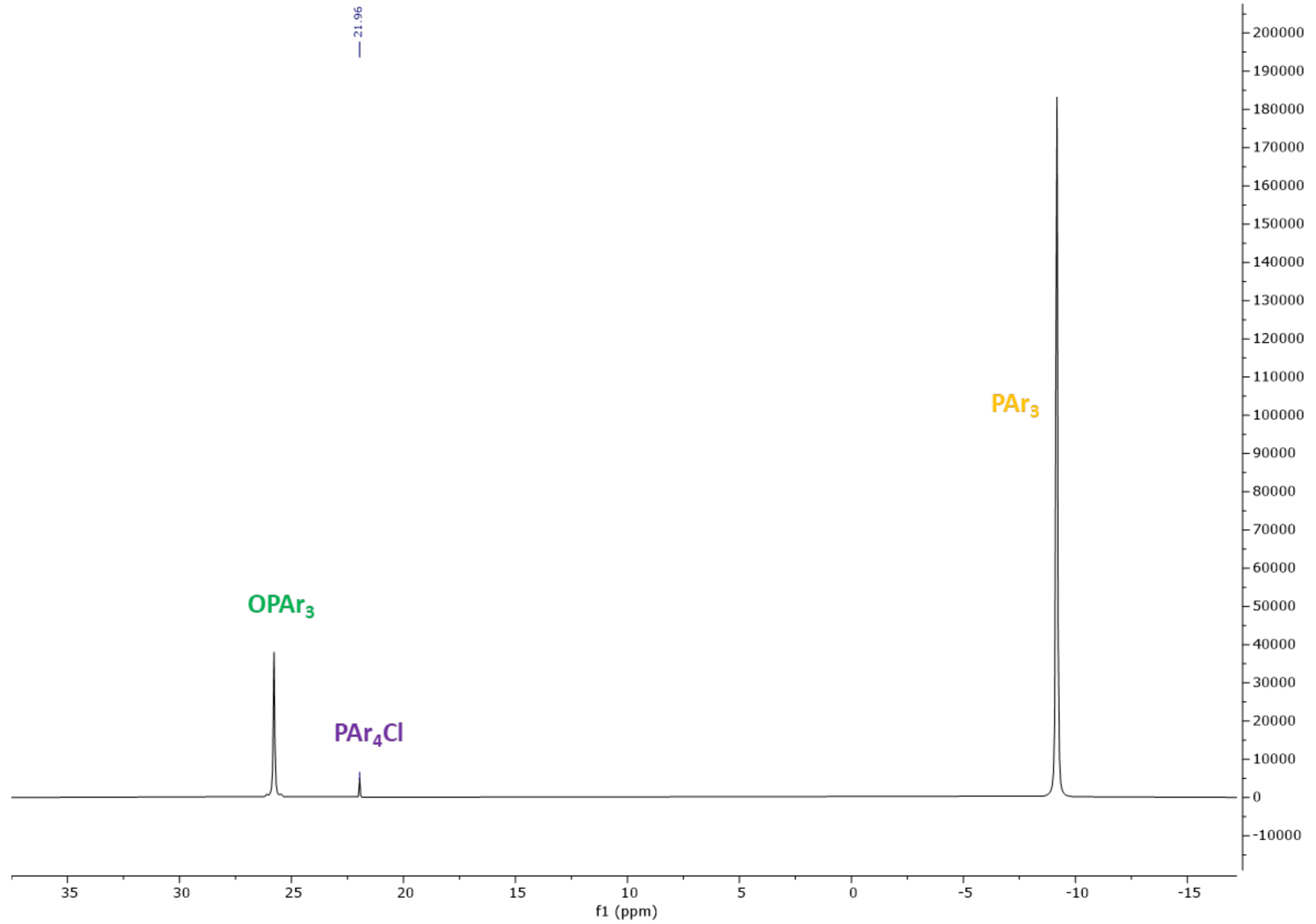

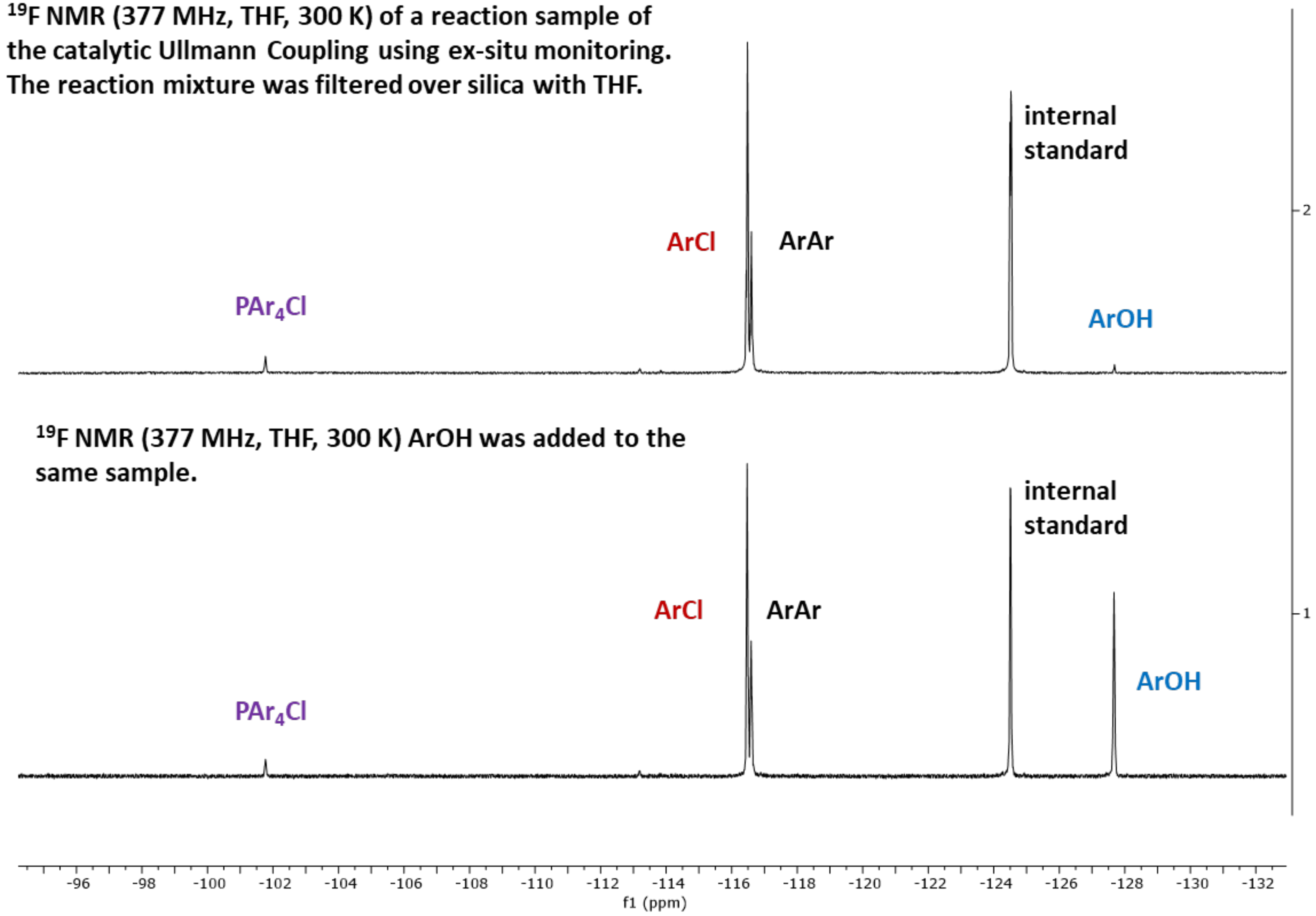
^{19}F NMR (377 MHz, THF, 300 K) of a reaction sample of the catalytic Ullmann Coupling using ex-situ monitoring. The reaction mixture was filtered over silica with THF.
PAr_4Cl
ArCl
ArAr
internal standard
ArOH
^{19}F NMR (377 MHz, THF, 300 K) ArOH was added to the same sample.
PAr_4Cl
ArCl
ArAr
internal standard
ArOH
2
1
-96
-98
-100
-102
-104
-106
-108
-110
-112
-114
-116
-118
-120
-122
-124
-126
-128
-130
-132
f1 (ppm)

^1H NMR (400 MHz, $CDCl_3$, 300 K)

$[Ni(DMF)_6][Zn(DMF)Cl_3]_2$

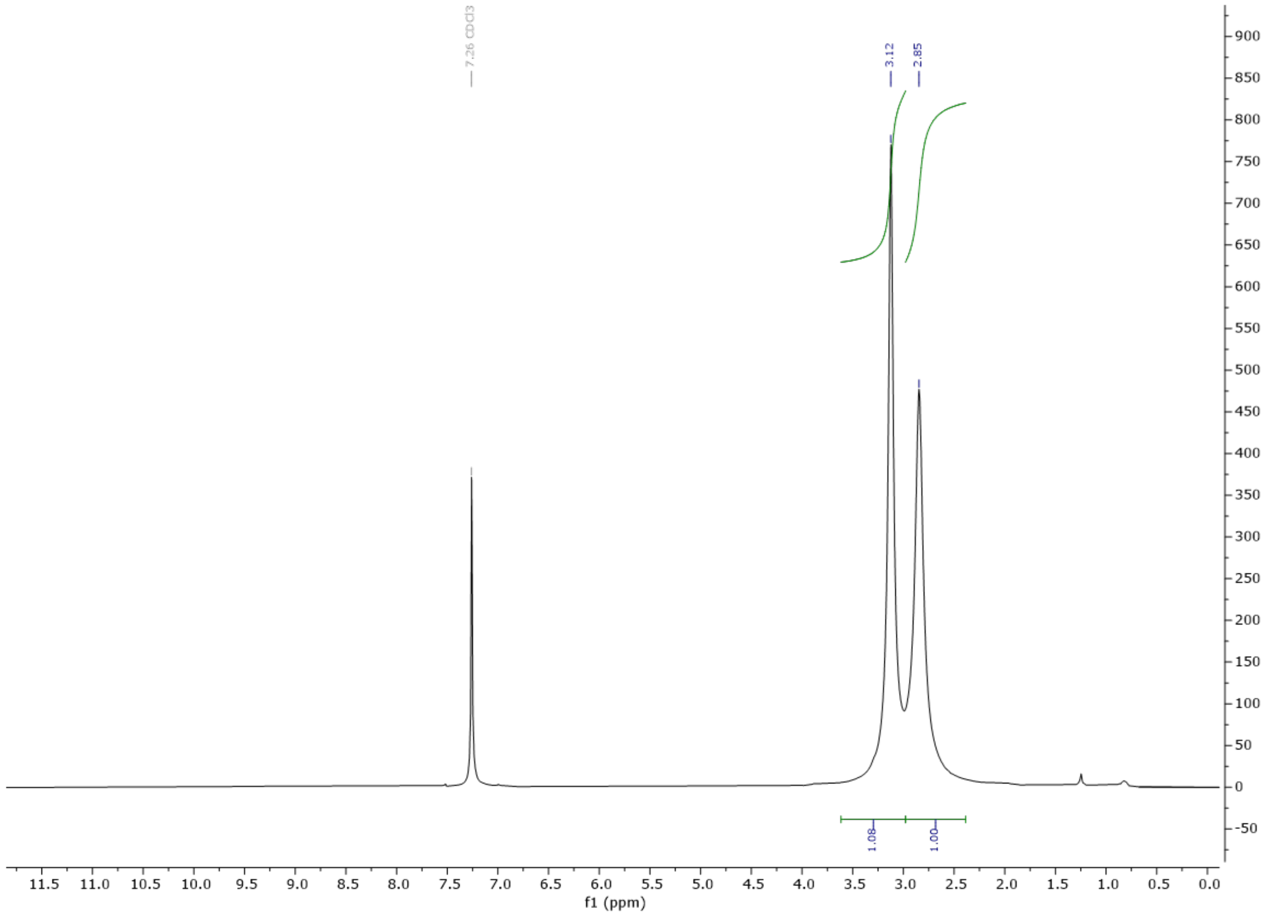

^{1}H NMR (400 MHz, $CDCl_3$, 278 K)

$[Ni(DMF)_6][Zn(DMF)Cl_3]_2$

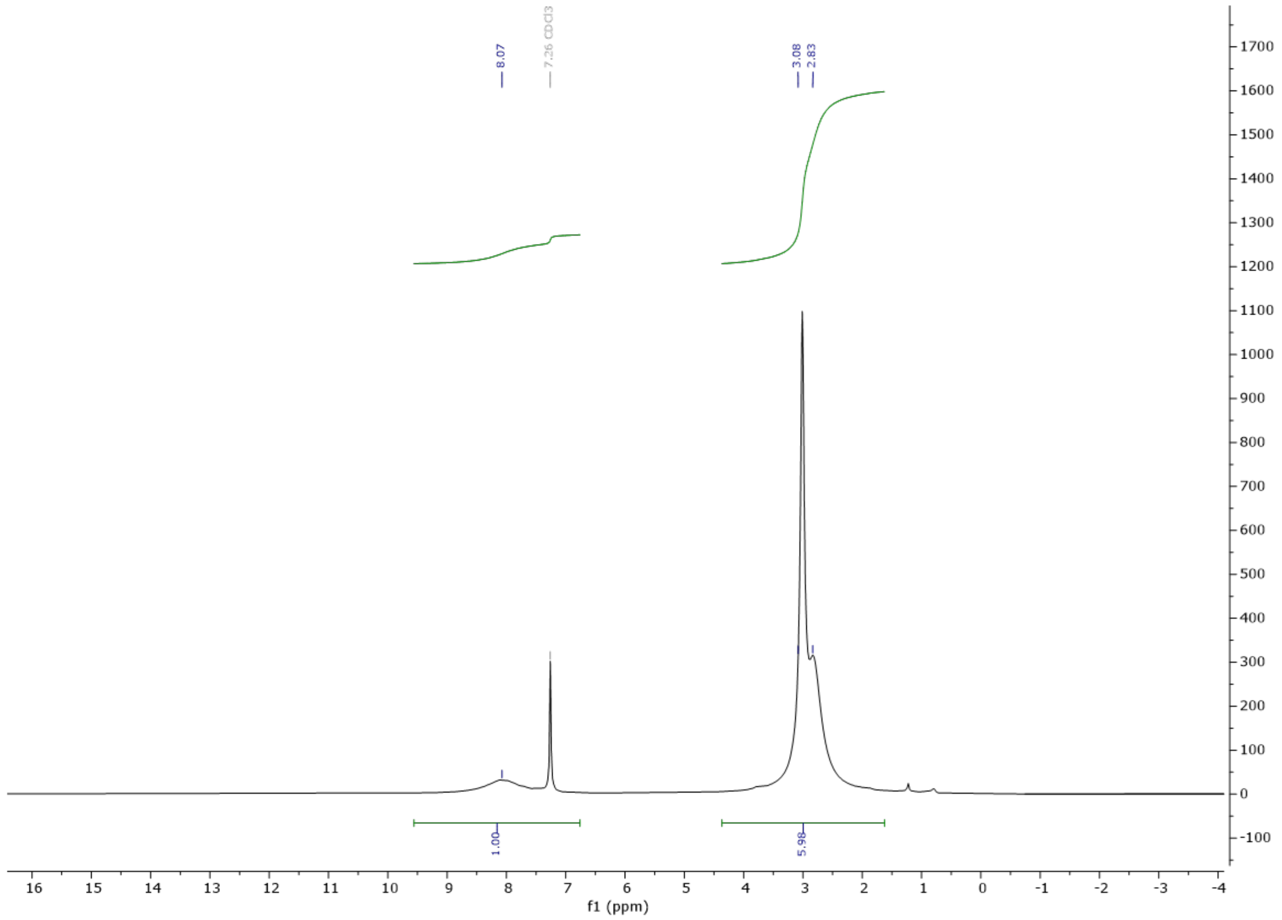

S11 MALDI

$L_2NiArCl$ was measured using MALDI but decomposed to give PAr_4Cl during the measurement.

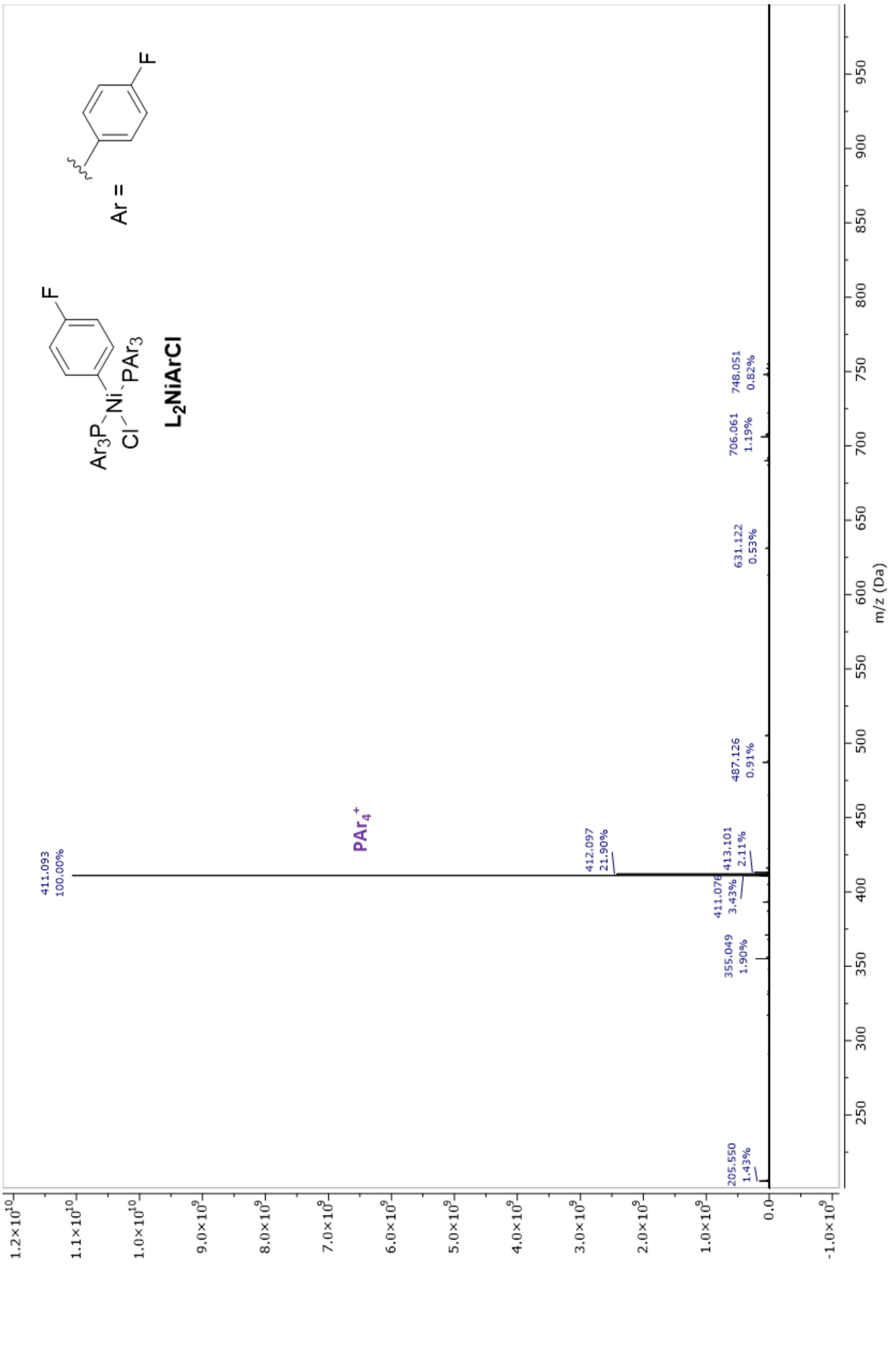

Ar3P
Ni
Cl
PAr3
F
L2NiArCl
Ar =
F
PAr4+
411.093
100.00%
412.097
21.90%
413.101
2.11%
411.076
3.43%
355.049
1.90%
205.550
1.43%
487.126
0.91%
631.122
0.53%
706.061
1.19%
748.051
0.82%
m/z (Da)
250
300
350
400
450
500
550
600
650
700
750
800
850
900
950
1.2×10^10
1.1×10^10
1.0×10^10
9.0×10^9
8.0×10^9
7.0×10^9
6.0×10^9
5.0×10^9
4.0×10^9
3.0×10^9
2.0×10^9
1.0×10^9
0.0
-1.0×10^9

S12 Single Crystal X-ray Diffraction

Mixed Metallate (**20**$_{DMF}$; CCDC 2373480) Data and Experimental

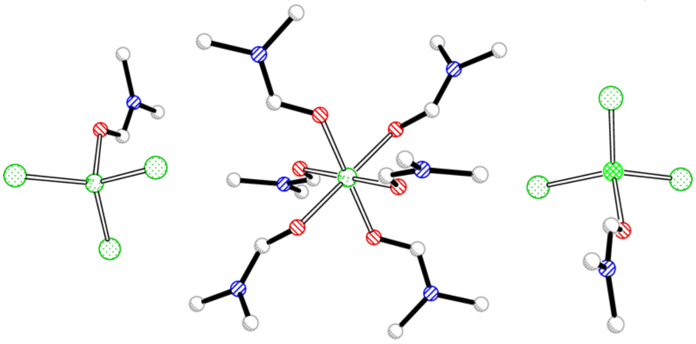

Experimental. Single green block-shaped crystals of **20$_{DMF}$** recrystallised from DMF by slow cooling. A suitable crystal with dimensions 0.38 × 0.37 × 0.33 mm^3 was selected and mounted on a MITIGEN holder NVH oil on a Bruker D8 VENTURE diffractometer. The crystal was kept at a steady T = 100.00 K during data collection. The structure was solved with the ShelXT 2018/2 (Sheldrick, 2018) solution program using dual methods and by using Olex2 1.5-beta (Dolomanov et al., 2009) as the graphical interface. The model was refined with ShelXL 2018/3 (Sheldrick, 2015) using full matrix least squares minimisation on $\boldsymbol{F^2}$.

Crystal Data. $C_{24}H_{56}Cl_6N_8Ni_{1.03}O_8Zn_{1.97}$, M_r = 986.71, triclinic, P-1 (No. 2), a = 8.6836(7) Å, b = 9.2120(7) Å, c = 14.8648(12) Å, α = 71.950(3)°, β□= 73.853(3)°, γ = 85.621(4)°, V = 1085.90(15) Å^3, T = 100.00 K, Z = 1, Z' = 0.5, μ(MoK$_\alpha$) = 1.938, 75143 reflections measured, 10496 unique (R_{int} = 0.0465) which were used in all calculations. The final wR_2 was 0.0704 (all data) and R_1 was 0.0332 (I≥2 σ(I)).

**Compound**	**20**$_{DMF}$ (GL24002 CCDC 2373480)
Formula	$C_{24}H_{56}Cl_6N_8Ni_{1.03}O_8Zn_{1.97}$
$D_{calc.}$/ g cm^{-3}	1.509
μ/mm^{-1}	1.938
Formula Weight	986.71
Colour	green
Shape	block-shaped
Size/mm^3	0.38×0.37×0.33
T/K	100.00
Crystal System	triclinic
Space Group	P-1
a/Å	8.6836(7)
b/Å	9.2120(7)
c/Å	14.8648(12)
α/°	71.950(3)
β/°	73.853(3)
γ/°	85.621(4)
V/Å^3	1085.90(15)
Z	1
Z'	0.5
Wavelength/Å	0.71073
Radiation type	MoK$_\alpha$
Θ_{min}/°	2.325
Θ_{max}/°	36.377
Measured Refl's.	75143
Indep't Refl's	10496
Refl's I≥2 σ(I)	9054
R_{int}	0.0465
Parameters	337
Restraints	0
Largest Peak	1.030
Deepest Hole	-0.936
GooF	1.110
wR_2 (all data)	0.0704
wR_2	0.0685
R_1 (all data)	0.0399
R_1	0.0332

(PAr$_3$Ni(Ar)Cl] complex **6** (CCDC 2373479) Data and Experimental

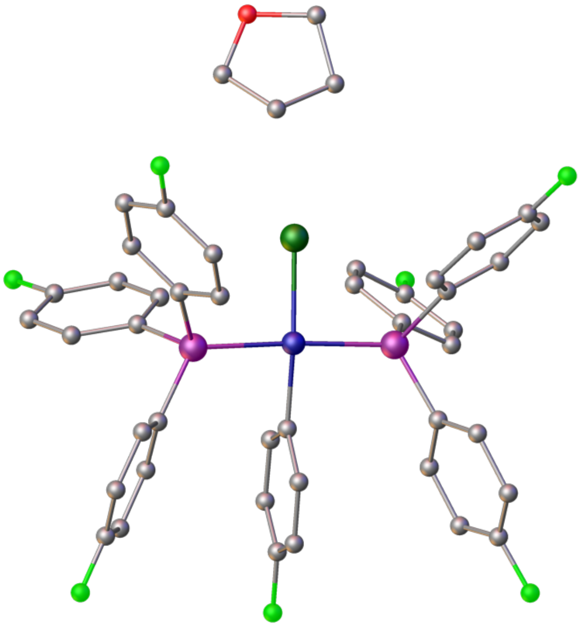

Experimental. Single yellow block-shaped crystals of **6** (GL23004 CCDC-2373479) recrystallised from a mixture of THF and hexane by solvent layering. A suitable crystal with dimensions 0.25 × 0.20 × 0.19 mm^3 was selected and mounted on a MITIGEN holder in Paratone oil. on a Bruker D8 VENTURE diffractometer. The crystal was kept at a steady T = 100.00 K during data collection. The structure was solved with the ShelXS (Sheldrick, 2008) solution program using direct methods and by using Olex2 1.5-beta (Dolomanov et al., 2009) as the graphical interface. The model was refined with olex2.refine 1.5-beta (Bourhis et al., 2015) using full matrix least squares minimisation on $\boldsymbol{F^2}$.

Crystal Data. $C_{46}H_{36}ClF_7NiOP_2$, M_r = 893.880, triclinic, $P1$ (No. 1), a = 9.231(1) Å, b = 10.0997(10) Å, c = 11.9430(13) Å, α = 87.161(4)°, β = 78.126(4)°, γ = 65.214(3)°, V = 988.40(18) Å^3, T = 100.00 K, Z = 1, Z' = 1, μ(Mo K$_\alpha$) = 0.709, 98366 reflections measured, 16547 unique (R_{int} = 0.0275) which were used in all calculations. The final wR_2 was 0.0460 (all data) and R_1 was 0.0220 (I≥2 σ(I)).

**Compound**	**6** (GL23004 CCDC 2373479)
Formula	$C_{46}H_{36}ClF_7NiOP_2$
$D_{calc.}$/ g cm^{-3}	1.502
μ/mm^{-1}	0.709
Formula Weight	893.880
Colour	yellow
Shape	block-shaped
Size/mm^3	0.25×0.20×0.19
T/K	100.00
Crystal System	triclinic
Flack Parameter	0.0042(13)
Hooft Parameter	0.0042(13)
Space Group	P1
a/Å	9.231(1)
b/Å	10.0997(10)
c/Å	11.9430(13)
α/°	87.161(4)
β/°	78.126(4)
γ/°	65.214(3)
V/Å^3	988.40(18)
Z	1
Z'	1
Wavelength/Å	0.71073
Radiation type	Mo K_α
Θ_{min}/°	2.77
Θ_{max}/°	36.24
Measured Refl's.	98366
Indep't Refl's	16547
Refl's I≥2 σ(I)	15714
R_{int}	0.0275
Parameters	847
Restraints	18
Largest Peak	0.3522
Deepest Hole	-0.4710
GooF	1.0587
wR_2 (all data)	0.0460
wR_2	0.0448
R_1 (all data)	0.0247
R_1	0.0220

(PAr$_3$Ni(Ar)Cl] complex **5** (CCDC 2373478) Data and Experimental

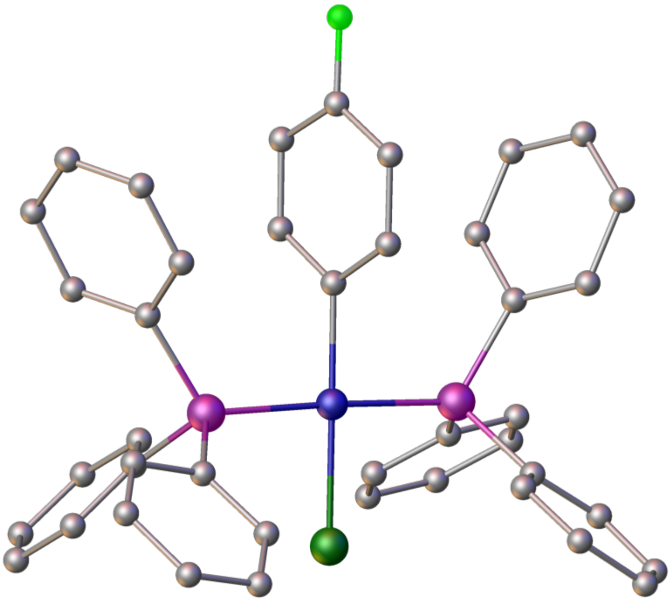

Experimental. Single orange prism-shaped crystals of **5** (GL23001 CCDC-2373478) recrystallised from a mixture of THF and hexane by solvent layering. A suitable crystal with dimensions 0.37 × 0.27 × 0.24 mm^3 was selected and mounted on a mitegen tip in Paratone oil on a Bruker D8 VENTURE diffractometer. The crystal was kept at a steady T = 100.00 K during data collection. The structure was solved with the **ShelXT** 2018/2 (Sheldrick, 2018) solution program using dual methods and by using **Olex2** 1.5-beta (Dolomanov et al., 2009) as the graphical interface. The model was refined with **olex2.refine** 1.5-beta (Bourhis et al., 2015) using full matrix least squares minimisation on $\boldsymbol{F^2}$.

Crystal Data. $C_{42}H_{34.31}ClF_{0.69}NiP_2$, M_r = 708.185, orthorhombic, *Pbca* (No. 61), a = 11.6316(14) Å, b = 23.444(3) Å, c = 25.269(3) Å, $\alpha = \beta = \gamma = 90^{\circ}$, V = 6890.4(15) Å^3, T = 100.00 K, Z = 8, Z' = 1, μ(Mo K$_\alpha$) = 0.767, 230769 reflections measured, 12880 unique (R_{int} = 0.0428) which were used in all calculations. The final wR_2 was 0.0350 (all data) and R_1 was 0.0203 (I≥2 σ(I)).

**Compound**	**5** (GL23001 CCDC 2373478)
Formula	$C_{42}H_{34.31}ClF_{0.69}NiP_2$
$D_{calc.}$/ g cm^{-3}	1.365
μ/mm^{-1}	0.767
Formula Weight	708.185
Colour	orange
Shape	prism-shaped
Size/mm^3	0.37×0.27×0.24
T/K	100.00
Crystal System	orthorhombic
Space Group	*Pbca*
a/Å	11.6316(14)
b/Å	23.444(3)
c/Å	25.269(3)
α/°	90
β/°	90
γ/°	90
V/Å^3	6890.4(15)
Z	8
Z'	1
Wavelength/Å	0.71073
Radiation type	Mo K_α
Θ_{min}/°	2.37
Θ_{max}/°	33.14
Measured Refl's.	230769
Indep't Refl's	12880
Refl's I≥2 σ(I)	10611
R_{int}	0.0428
Parameters	834
Restraints	192
Largest Peak	0.4319
Deepest Hole	-0.5345
GooF	1.1114
wR_2 (all data)	0.0350
wR_2	0.0301
R_1 (all data)	0.0345
R_1	0.0203

S13 References

S1. Hanson, G. R.; Gates, K. E.; Noble, C. J.; Griffin, M.; Mitchell, A.; Benson, S. XSophe-Sophe-XeprView®. A Computer Simulation Software Suite (v. 1.1.3) for the Analysis of Continuous Wave EPR Spectra. *J. Inorg. Biochem.* **2004**, *98*, 903–916. https://doi.org/10.1016/j.jinorgbio.2004.02.003.

S2. Figueira, C. A.; Lopes, P. S.; Gomes, C. S. B.; Gomes, J. C. S.; Lemos, F.; Gomes, P. T. New Phenyl–Nickel Complexes of Bulky 2-Iminopyrrolyl Chelates: Synthesis, Characterisation and Application as Aluminium-Free Catalysts for the Production of Hyperbranched Polyethylene. *Dalton Trans.* **2018**, *47*, 15857–15872. https://doi.org/10.1039/C8DT02824A.

S3. Hernán-Gómez, A.; Orr, S. A.; Uzelac, M.; Kennedy, A. R.; Barroso, S.; Jusseau, X.; Lemaire, S.; Farina, V.; Hevia, E. Exploiting Synergistic Effects in Organozinc Chemistry for Direct Stereoselective C-Glycosylation Reactions at Room Temperature. *Angew. Chem. Int. Ed.* **2018**, *57*, 10630–10634. https://doi.org/10.1002/anie.201805758.

S4. Le Hiress, M.; Akagah, B.; Bernadat, G.; Tu, L.; Thuillet, R.; Huertas, A.; Phan, C.; Fadel, E.; Simonneau, G.; Humbert, M.; Jalce, G.; Guignabert, C. Design, Synthesis, and Biological Activity of New N-(Phenylmethyl)-Benzoxazol-2-Thiones as Macrophage Migration Inhibitory Factor (MIF) Antagonists: Efficacies in Experimental Pulmonary Hypertension. *J. Med. Chem.* **2018**, *61*, 2725–2736. https://doi.org/10.1021/acs.jmedchem.7b01312.

S5. Öztürk, B. Ö.; Bucak, E.; Karabulut, S. In Situ Modification of the Grubbs First Generation Catalyst: A Highly Controllable Metathesis Catalyst Bearing Tridentate Schiff Base Ligands. *J. Mol. Catal. Chem.* **2013**, *376*, 53–62. https://doi.org/10.1016/j.molcata.2013.04.010.

S6. Gao, Y.; Hall, A. M. R.; Fohn, N. A.; King, E. J.; Mitchell, L. A. L.; Steedman, G. A.; Lloyd-Jones, G. C. A Simple Device for Automated Mixing of Heterogeneous Solid-Liquid Reactions During In-Situ Monitoring by NMR Spectroscopy. *Eur. J. Org. Chem.* **2024**, e202400095. https://doi.org/10.1002/ejoc.202400095.

S7. Fearnley, A. F.; An, J.; Jackson, M.; Lindovska, P.; Denton, R. M. Synthesis of Quaternary Aryl Phosphonium Salts: Photoredox-Mediated Phosphine Arylation. *Chem. Commun.* **2016**, *52*, 4987–4990. https://doi.org/10.1039/C6CC00556J.

S8. Colon, I.; Kelsey, D. R. Coupling of Aryl Chlorides by Nickel and Reducing Metals. *J. Org. Chem.* **1986**, *51*, 2627–2637. https://doi.org/10.1021/jo00364a002.

S9. Maheswari, P. U.; Renuga, D.; Henry, L. J. K.; Ruckmani, K. A Novel Biphenolic Ligand for Selective Mg2+ and Zn2+ Ions Sensing Followed by Colorimetric, Spectroscopic and Cell Imaging Methods. *Eur. J. Pharm. Sci.* **2018**, *116*, 61–69. https://doi.org/10.1016/j.ejps.2018.01.025.

S10. Saraev, V. V.; Kraikivskii, P. B.; Matveev, D. A.; Zelinskii, S. N.; Lammertsma, K. EPR Study of the Oxidation Reaction of Nickel(0) Phosphine Complexes with Lewis and Brønsted Acids. *Inorg. Chim. Acta* **2006**, *359*, 2314–2320. https://doi.org/10.1016/j.ica.2006.01.028.
